# Nature of the heavy formal double bonds As=Ch, Sb=Ch and Bi=Ch (Ch = S, Se, Te) in NCN-pincer supported arsinidene, stibinidene and bismuthinidene chalcogenides†

**DOI:** 10.1039/d5sc03320a

**Published:** 2025-07-18

**Authors:** Fabio Meyer, Arina Siumbeli, Libor Dostál, Emanuel Hupf, Jens Beckmann

**Affiliations:** a Institute for Inorganic Chemistry and Crystallography, University of Bremen Germany emanuel.hupf@uni-bremen.de j.beckmann@uni-bremen.de; b Department of General and Inorganic Chemistry, Faculty of Chemical Technology, University of Pardubice Studentska, 573 Pardubice 532 10 Czech Republic libor.dostal@upce.cz

## Abstract

The synthesis of arylpnictinidenes 2,6-(Ph_2_PNMes)_2_C_6_H_3_Pn based upon a novel bis(phosphine imine) NCN-pincer ligand is reported (Pn = As, Sb, Bi). The oxidation of 2,6-(Ph_2_PNMes)_2_C_6_H_3_Pn with sulfur, selenium and tellurium, respectively, afforded arylarsinidene chalcogenides 2,6-(Ph_2_PNMes)_2_C_6_H_3_AsCh, arylstibinidene chalcogenides 2,6-(Ph_2_PNMes)_2_C_6_H_3_SbCh and arylbismuthinidene chalcogenides 2,6-(Ph_2_PNMes)_2_C_6_H_3_BiCh, which can be formulated as containing terminal As=Ch, Sb=Ch and Bi=Ch double bonds. Based on the complementary bonding analysis, the bonding situation is best described in terms of bipolar ^+^Pn–Ch^−^ single bonds (Ch = S, Se, Te).

## Abstract 2

Oxidation of the arylpnictinidenes 2,6-(Ph_2_PNMes)_2_C_6_H_3_Pn (Pn = As, Sb, Bi) with the heavier chalcogens (Ch = S, Se, Te) led to the formation of arylpnictinidene chalcogenides (Ph_2_PNMes)_2_C_6_H_3_PnCh formally containing terminal double bonds, which are best described as bipolar ^+^Pn–Ch^−^ single bonds.

## Graphical abstract

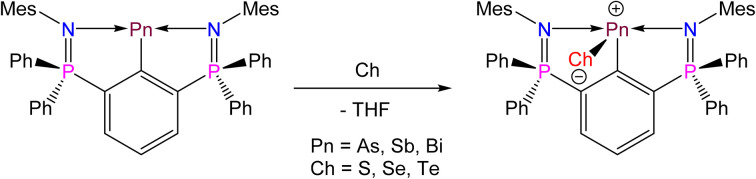

## Introduction

**Scheme 1:**
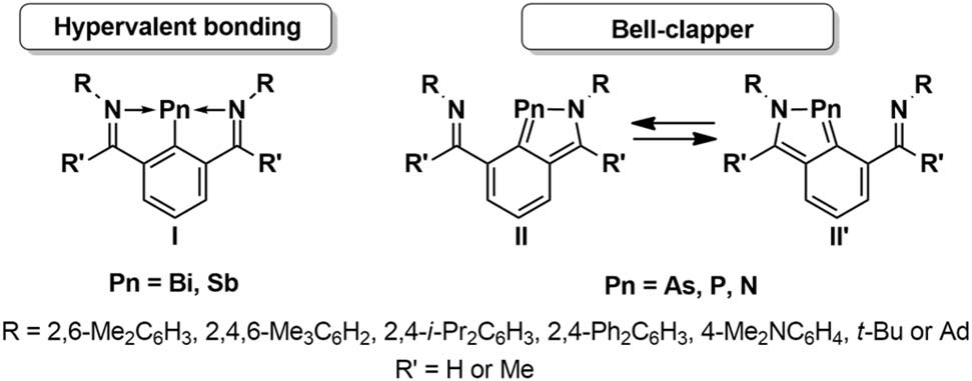
Electronically stabilized arylpnictinidenes containing NCN-pincer ligands.

Arylpnictinidenes RPn (Pn = N, P, As, Sb, Bi; R = aryl) are neutral carbene analogues of group 15^1,2^ that have received tremendous attention in recent years owing to their low valency and arising opportunities for main group driven bond activation and catalysis.^3,4^ In their native form, arylpnictinidenes, are fiercely reactive species due to their electron deficiency as well as their electronic triplet ground states and only very recently, it has been discovered that kinetic stabilization of triplet arylpnictinidenes can be achieved using extremely bulky and rigid M^S^Fluind substituents that effectively shield the pnictogen atoms.^5–9^ Prior to this discovery, singlet arylpnictinidenes were obtained by electronic stabilization using intramolecularly coordinating aryl substituents comprising N-donor atoms, which are able to compensate the electron deficiency of the pnictogen atoms. In 2010, the first isolable arylstibinidene and arylbismuthinidene, 2,6-[RNC(R′)]_2_C_6_H_3_Pn (I: Pn = Sb, Bi; R = 2,6-Me_3_C_6_H_3_, R′ = Me) were reported using an adjustable bis(aldimine) and bis(ketimine)-based NCN-pincer ligand system (Scheme 1).^10^ Structural elucidation revealed that both N atoms are involved in the coordination of the pnictogens giving rise to formal hypervalent bonding. Since then, closely related arylstibinidenes and arylbismuthinidenes I have been reported with the same NCN-pincer ligand system, at which the positions of the organic substituents R and R′ were varied with the aim of fine tuning the reactivity.^11–14^

The lighter group 15 analogues, namely arylnitrenes, -phosphinidenes and -arsinidenes II were reported with the same bis(imine)-based NCN-pincer ligand system.^14–19^ Notably, within the lighter arylpnictindenes, one imine-group preferentially coordinates to the pnictogens due to the smaller size and aromatic stabilization of the resulting five-membered heterocycles, while a fluxional exchange (“bell-cappers”) was observed in solution. Asymmetric coordination modes may be locked in upon modification of the NCN-pincer system.^11,14,20,21^

The arylpnictinidenes I and II were subject to extensive reactivity studies. The heavier arylpnictinidenes 2,6-[RNC(H)]_2_C_6_H_3_Pn (Pn = As, Sb, Bi) were used as donor ligands for the preparation of numerous metal carbonyl complexes of Cr, Mo, W, Fe, Mn and Co^12,16^ as well as Au^22^ or in Diels–Alder type cycloadditions with alkynes.^23,24^ The stibinidene 2,6-[*t*-BuNC(H)]_2_C_6_H_3_Sb was utilized as an effective main group redox catalyst in the hydroboration of disulfides^25^ as well as in reversible addition reactions with *N*-alkyl/arylmaleimides.^26^ The bismuthinidene 2,6-[RNC(H)]_2_C_6_H_3_Bi undergoes (light assisted) oxidative addition with aryl iodides,^27,28^ stemming from single-electron transfer (SET) processes.^29^ Activation of dinitrogen oxide provided an asymmetric oxidation product,^13^ which was able to oxidize HBpin to HOBpin. Reaction of the bismuthinidene 2,6-[2′,6′-Ph_2_C_6_H_3_NC(Me)]_2_C_6_H_3_Bi with the 2,4,6-tri-*tert*-butylphenoxy radical (2,4,6-TTBP) gave rise to a Bi(iii) species featuring an extremely labile Bi–O bond, which could be readily transformed into an arylbismuth(ii) species able to activate protic species, such as water, ammonia, phenol and aniline.^30^ Furthermore, upon single electron oxidation with [Cp_2_Fe][BAr^F^_4_], the arylbismuthinidene could be converted into the related radical cations.^31^ The utility of the arylbismuthinidene 2,6-[*t*-BuNC(H)]_2_C_6_H_3_Bi as a catalyst was showcased for transfer hydrogenation reactions of azobenzenes,^32^ which occur without metal–ligand cooperativity,^20^ the hydrodefluorination of polyfluorinated aromatics,^33^ and for the degradation of sulfur hexafluoride and phenylsulfur pentafluoride.^34^ Most recent applications in catalysis include light-induced trifluoromethylation of heteroarenes,^35^ light-induced reductive cyclopropanation of alkenes with diiodomethane and manganese powder,^36^ and the intramolecular aminocyclization affording cyclic carbamates.^37^

Given the rich diversity in the reactivity of stibinidenes and bismuthinidenes as well as the non-innocent character of the bis(aldimine) and bis(ketimine)-based NCN-pincer ligand system, we envisaged a novel bis(phosphine imine)-based NCN-pincer scaffold, derived from the 2,6-[bis(diphenylphosphinophenyl)]phenyl substituent, recently introduced by us and applied for the synthesis of transition metal complexes.^38–40^ This novel bis(phosphine imine)-based NCN-pincer scaffold based upon PNMes groups allowed the preparation of a novel arsinidene, stibinidene and bismuthinidene, the oxidation of which with chalcogens led to the formation of electronically stabilized stibinidene chalcogenides and bismuthinidene chalcogenides comprising formal As=Ch, Sb=Ch and Bi=Ch double bonds (Ch = S, Se, Te) including the first structurally authenticated Bi=Te double bond. These compounds nicely complement the kinetically stabilized stibinidene chalcogenides reported very recently.^41^

## Results & discussion

### Synthetic aspects

**Scheme 2:**
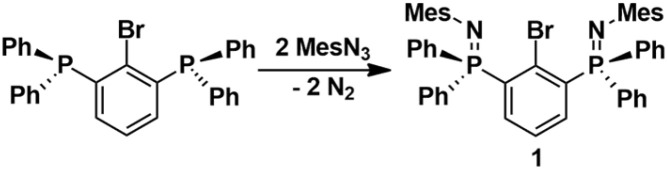
Synthesis of 1.

The ligand precursor was synthesized by a Staudinger reaction of 2,6-(Ph_2_P)_2_C_6_H_3_Br^42^ with two equivalents of mesityl azide to provide 2,6-(Ph_2_PNMes)_2_C_6_H_3_Br (1) as colourless crystals in 84% yield (Scheme 2). The ^31^P NMR spectrum of 1 consists of a singlet at *δ* = −8.9 ppm, which is more deshielded compared to related pincer complexes reflecting the relative coordination strengths of the N atoms, *vide infra*.

**Scheme 3:**
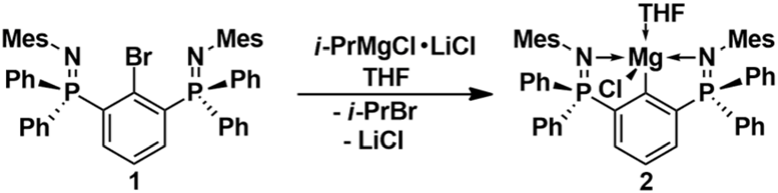
Synthesis of 2.

The metal halide exchange reaction of 1 with the Turbo-Grignard-reagent, *i*-PrMgCl·LiCl, produced the arylmagnesium chloride 2,6-(Ph_2_PNMes)_2_C_6_H_3_MgCl·THF·Et_2_O (2) as pale-yellow crystals in 90% yield (Scheme 3).

**Fig. 1:**
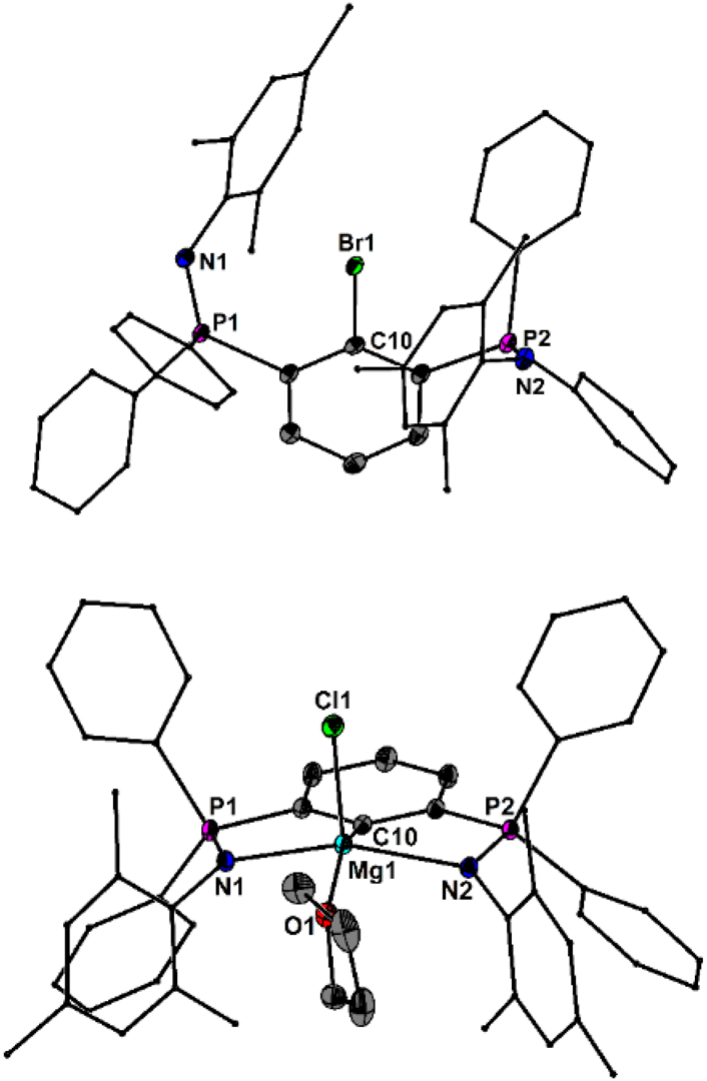
Molecular structures of 1 and 2 showing 50% probability ellipsoids and the essential atomic numbering scheme of the core region. Substituents are shown as wireframes for clarity. Selected bond lengths [Å] for 1: P1–N1 1.546(2), P2–N2 1.548(2); for 2: P1–N1 1.596(2), P2–N2 1.598(2), Mg1–N1 2.222(2), Mg1–N2 2.248(2), Mg1–C10 2.130(2), Mg1–Cl1 2.381(1), Mg1–O1 2.073(2).

The molecular structures of 1 and 2 are shown in Fig. 1. As anticipated, the N atoms of 1 are not involved in any interactions with the Br atom. The short, unaltered P–N bond lengths of 1 (1.546(2), 1.548(2) Å) may be regarded as reference values for the discussion of the relative coordination strengths in related pincer complexes, *vide infra*. In 2, both N atoms firmly coordinate to the Mg atom, leading to a tetrahedral spatial arrangement of the Mg atom in 2, defined by a CN_2_O donor set. The Mg–N bond lengths (2.222(2), 2.248(2) Å) are marginally unequal. As the result of this coordination, the P–N bond lengths of 2 (1.596(2), 1.598(2) Å) are slightly longer than in 1. Additionally, 2 shows a ^31^P-NMR chemical shift of 10.0 ppm.

**Scheme 4:**
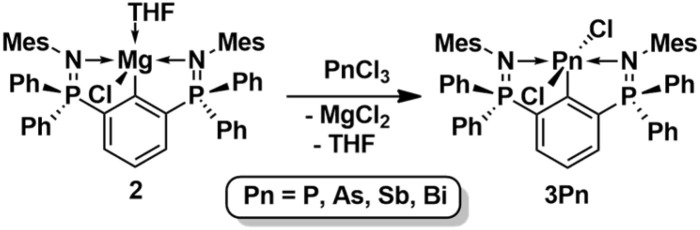
Synthesis of 3Pn (*Pn* = P, As, Sb, Bi).

The salt metathesis reaction of 2 with pnictogen trichlorides, PnCl_3_ (Pn = P, As, Sb, Bi) afforded the aryldichloropnictogens, 2,6-(Ph_2_PNMes)_2_C_6_H_3_PnCl_2_ (3Pn) as colourless crystalline solids in 61–94% yield (Scheme 4). Unlike 3As, 3Sb and 3Bi that are indefinitely stable when kept under inert conditions, 3P decomposes within a few days into ill-defined products even when stored as a solid under argon.

**Fig. 2:**
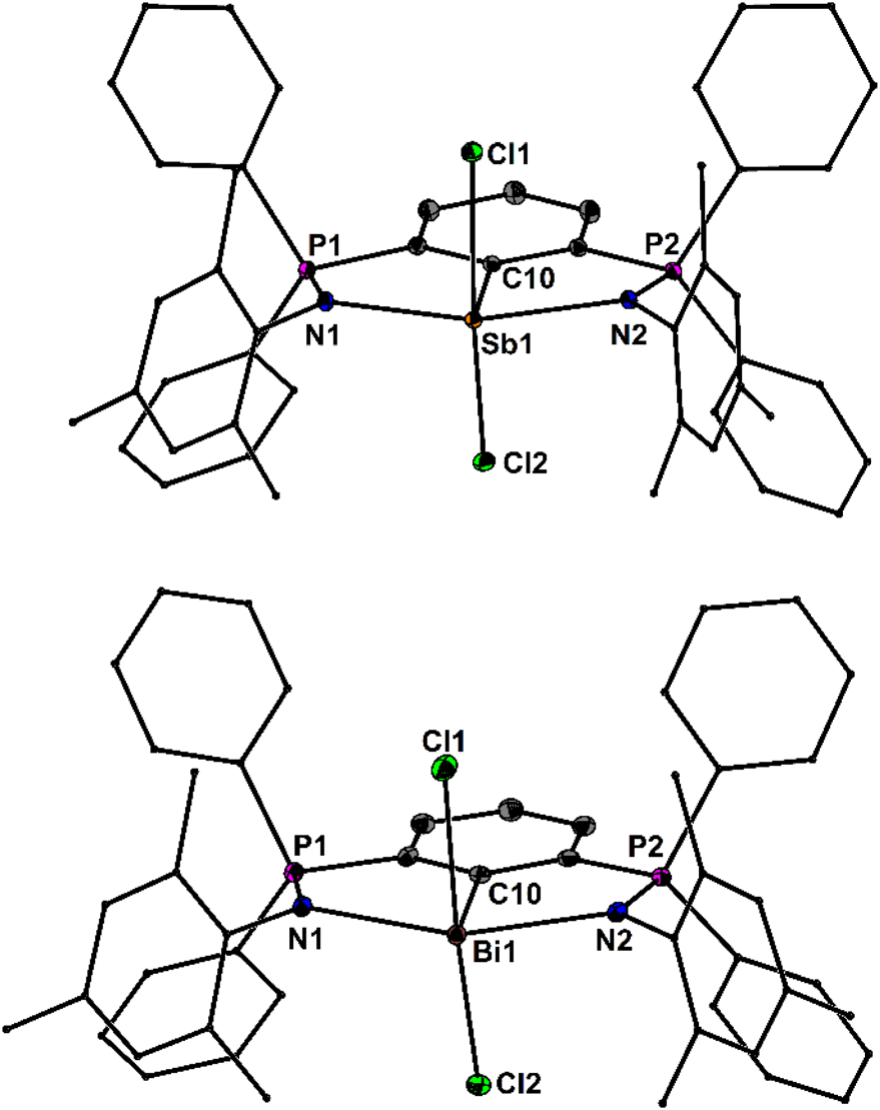
Molecular structures of 3Sb and 3Bi showing 50% probability ellipsoids and the essential atomic numbering scheme of the core region. Substituents are shown as wireframes for clarity. Selected bond lengths [Å] for 3Sb: P1–N1 1.604(2), P2–N2 1.600(1), Sb1–N1 2.286(1), Sb1–N2 2.326(1), Sb1–C10 2.129(2), Sb1–Cl1 2.605(1), Sb1–Cl2 2.693(1); for 3Bi: P1–N1 1.595(2), P2–N2 1.596(2), Bi1–N1 2.438(2), Bi1–N2 2.395(2), Bi1–C10 2.219(3), Bi1–Cl1 2.626(1), Bi1–Cl2 2.849(1).

The molecular structures of 3Sb and 3Bi are shown in Fig. 2. The spatial arrangement of the Sb and Bi atoms is distorted square pyramidal due to the stereochemically active lone pairs and defined by CN_2_Cl_2_ donors sets. The Sb–N and Bi–N bond lengths of 3Sb (2.286(1), 2.326(1) Å) and 3Bi (2.395(2), 2.438(2) Å) are slightly unequal and significantly shorter than those of the arylantimony- and -bismuth dichlorides 2,6-(*t*-BuNCH)_2_C_6_H_3_PnCl_2_ (Sb: 2.415(2), 2.399(2) Å, Bi: 2.470(7), 2.499(6) Å).^43^ In turn, the Sb–Cl and Bi–Cl bond lengths of 3Sb (2.605(1), 2.693(1) Å) and 3Bi (2.626(1), 2.849(1) Å) are slightly longer than those of 2,6-(*t*-BuNCH)_2_C_6_H_3_PnCl_2_ (Sb: 2.597(1), 2.583(1) Å; Bi: 2.662(2), 2.689(2) Å).^43^ Based upon the comparison of the P–N bond lengths, the N-coordination in 3Sb (1.604(2), 1.600(1) Å) is somewhat stronger than in 3Bi (1.595(2), 1.596(2) Å), which reflects the greater Lewis acidity of the Sb compound. The phosphine imide moieties give rise to ^31^P NMR chemical shifts of *δ* = 19.8 (3P), 21.1 (3As), 22.9 (3Sb) and 37.6 ppm (3Bi), respectively. The central P-atom of 3P revealed a triplet centred at *δ* = 100.0 ppm with a *J*(^31^P–^31^P) coupling of 46 Hz. Despite arduous efforts, we failed to obtain single crystals of 3P and 3As. On one occasion, we obtained a small crop of crystals that was identified as the arylchloroarsenium ion, [2,6-(Ph_2_PNMes)_2_C_6_H_3_AsCl][As_2_OCl_5_] [4As][As_2_OCl_5_], which serendipitously formed from 3As, excess AsCl_3_ and adventitious moisture.

**Scheme 5:**
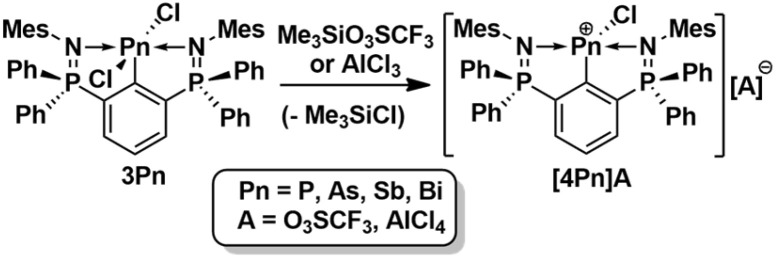
Synthesis of [4Pn][A] (Pn = P, As, Sb, Bi; A = O_3_SCF_3_, AlCl_4_).

In an effort to deliberately prepare arylchloropnictogenium ions, we reacted the aryldichloropnictogens 3Pn (Pn = P, As, Sb, Bi) with trimethylsilyl triflate, Me_3_SiO_3_SCF_3_, or aluminium trichloride, AlCl_3_ (Scheme 5). Both Lewis acids abstracted a chloride leading to the formation of the desired arylchloropnictogenium ions [2,6-(Ph_2_PNMes)_2_C_6_H_3_PnCl][A] ([4Pn]A, Pn = P, As, Sb, Bi; A^–^ = O_3_SCF_3_^−^, AlCl_4_^−^) that were isolated as colourless crystalline solids in quantitative yields.

**Fig. 3:**
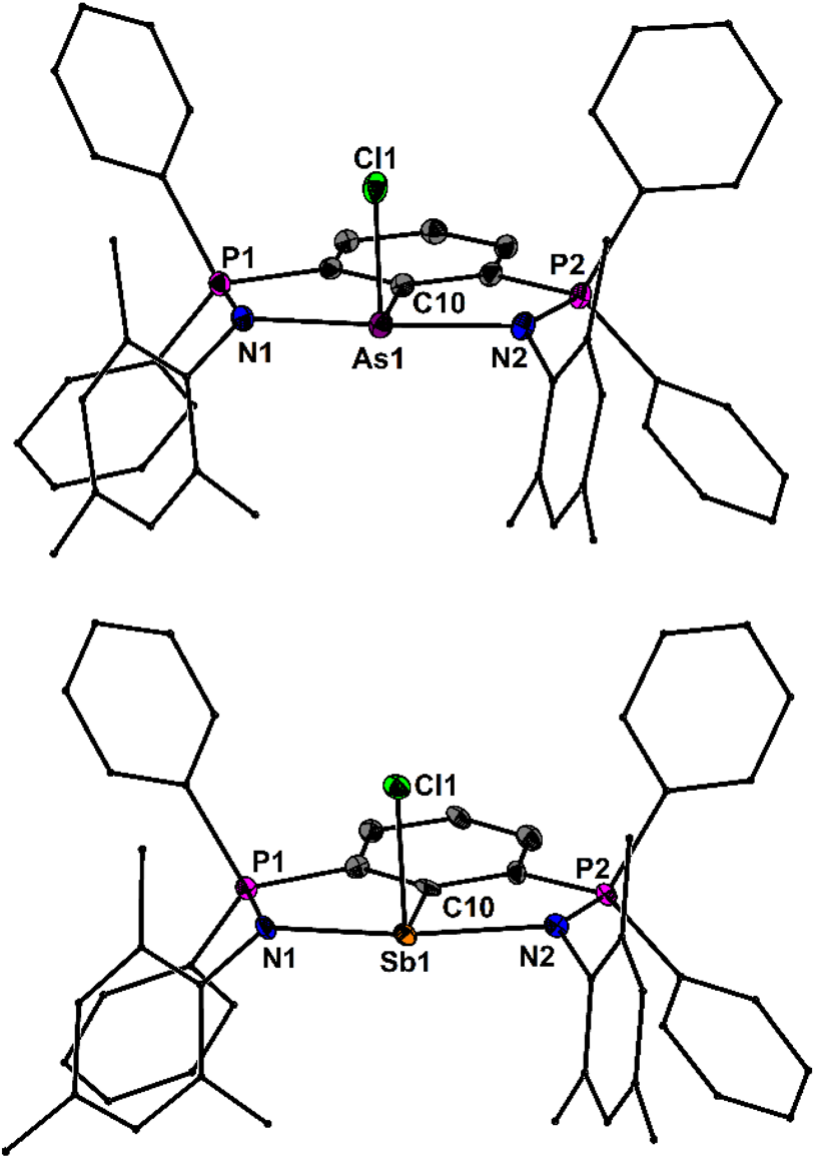
Molecular structures of [4As]^+^ and [4Sb]^+^ showing 50% probability ellipsoids and the essential atomic numbering scheme of the core region. Substituents are shown as wireframes for clarity. Selected bond lengths [Å] for [4As]^+^: P1–N1 1.605(3), P2–N2 1.601(3), As1–N1 2.122(3), As1–N2 2.114(3), As1–C10 1.967(3), As1–Cl1 2.242(1); for [4Sb]^+^: P1–N1 1.601(6), P2–N2 1.597(6), Sb1–N1 2.226(5), Sb1–N2 2.272(6), Sb1–C10 2.158(6), Sb1–Cl1 2.410(2).

The molecular structures of [4As]^+^ and [4Sb]^+^ are depicted in Fig. 3. The spatial arrangement of the As and Sb atoms is distorted disphenoidal due to the stereochemically active lone pairs and defined by CN_2_Cl donor sets. The Sb–N bond lengths of [4Sb]^+^ (2.226(5), 2.272(6) Å) are slightly shorter than those of 3Sb (2.286(1), 2.326(1) Å) and the remaining Sb–Cl bond length [4Sb]^+^ (2.410(2) Å) is shorter than those in 3Sb (2.605(1), 2.693(1) Å), but longer than in the chlorostibenium cations [2,6-(*t*-BuNCH)_2_C_6_H_3_SbCl]^+^ (2.360(1) Å) and [2,6-(2′,6′-Me_2_C_6_H_4_NCH)_2_C_6_H_3_SbCl]^+^ (2.361(1) Å).^44^ Based upon the P–N bond lengths of [4As]^+^ (1.597(6), 1.601(6) Å) and [4Sb]^+^ (1.600(1), 1.601(6) Å), the N-coordination within these two cations is very similar, but slightly stronger than in 3Sb and 3Bi, *vide supra*. Interestingly, the two mesityl groups and the two P atoms of the complete series [4Pn]^+^ (Pn = P, As, Sb, Bi) are magnetically inequivalent in solution. The ^31^P NMR spectrum of [4P]^+^ gives rise to three equally intense signals at *δ* = 129.4 59.3 and 33.0 ppm. The first two signals comprise doublets with identical *J*(^31^P–^31^P) couplings of 62 Hz, while the last signal is a singlet, which suggest an unsymmetrical coordination mode in solution. Consistently, the remaining compounds show pairs of ^31^P NMR signals at *δ* = 60.0/33.7 ([4As]^+^), 60.0/30.0 ([4Sb]^+^) and 78.2/37.5 ppm for ([4Bi]^+^), respectively.

**Scheme 6:**
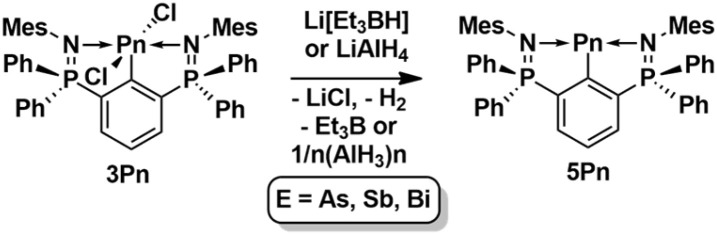
Synthesis of 5Pn (Pn = As, Sb, Bi).

**Scheme 7:**
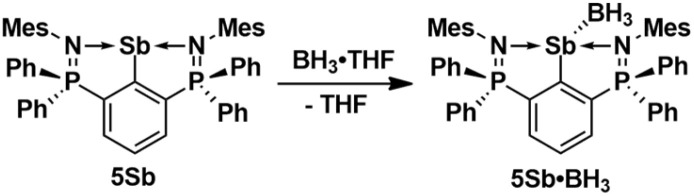
Synthesis of 5Sb·BH_3_.

The reduction of the heavier aryldichloropnictogens 3Pn (Pn = As, Sb, Bi), with Li[Et_3_BH] provided the arylpnictinidenes 2,6-(Ph_2_PNMes)_2_C_6_H_3_Pn (5Pn) as red solids in quantitative yields (Scheme 6). Alternatively, the arylstibinidene 2,6-(Ph_2_PNMes)_2_C_6_H_3_Sb (5Sb) was also obtained by the reduction of 3Sb with LiAlH_4_ and isolated as 5Sb·(AlCl_3_·THF)·THF. Notably, neither the donor acceptor complex AlCl_3_·THF nor the free THF showed any coordination to the Sb atom, but facilitated crystallization. Unfortunately, the arylpnictinidenes are thermally unstable and start to decompose within a few hours under argon. While attempting to grow single crystals of the arylbismuthinidene 2,6-(Ph_2_PNMes)_2_C_6_H_3_Bi (5Bi), a small crop of a crystalline decomposition product was obtained, namely the arylalkylbismuthenium ion [2,6-(Ph_2_PNMes)_2_C_6_H_3_BiEt][Et_4_B] (Fig. S128†), which formed *via* oxidative addition of the ethylboron species and ethyl group scrambling. Interestingly, all attempts to prepare 5Sb using NaBH_4_ as the reducing agent gave the donor acceptor complex 5Sb·BH_3_ as unstable red crystals. The reaction of 5Sb with BH_3_·THF provided the same complex 5Sb·BH_3_ that was isolated quantitatively, while 5Bi showed no reactivity towards BH_3_·THF (Scheme 7). So far, all attempts to reduce 3P with various reducing agents, such as KC_8_, lithium, potassium, Li[Et_3_BH] or Cp_2_Co, have resulted only in ill-defined mixtures of products.

**Fig. 4:**
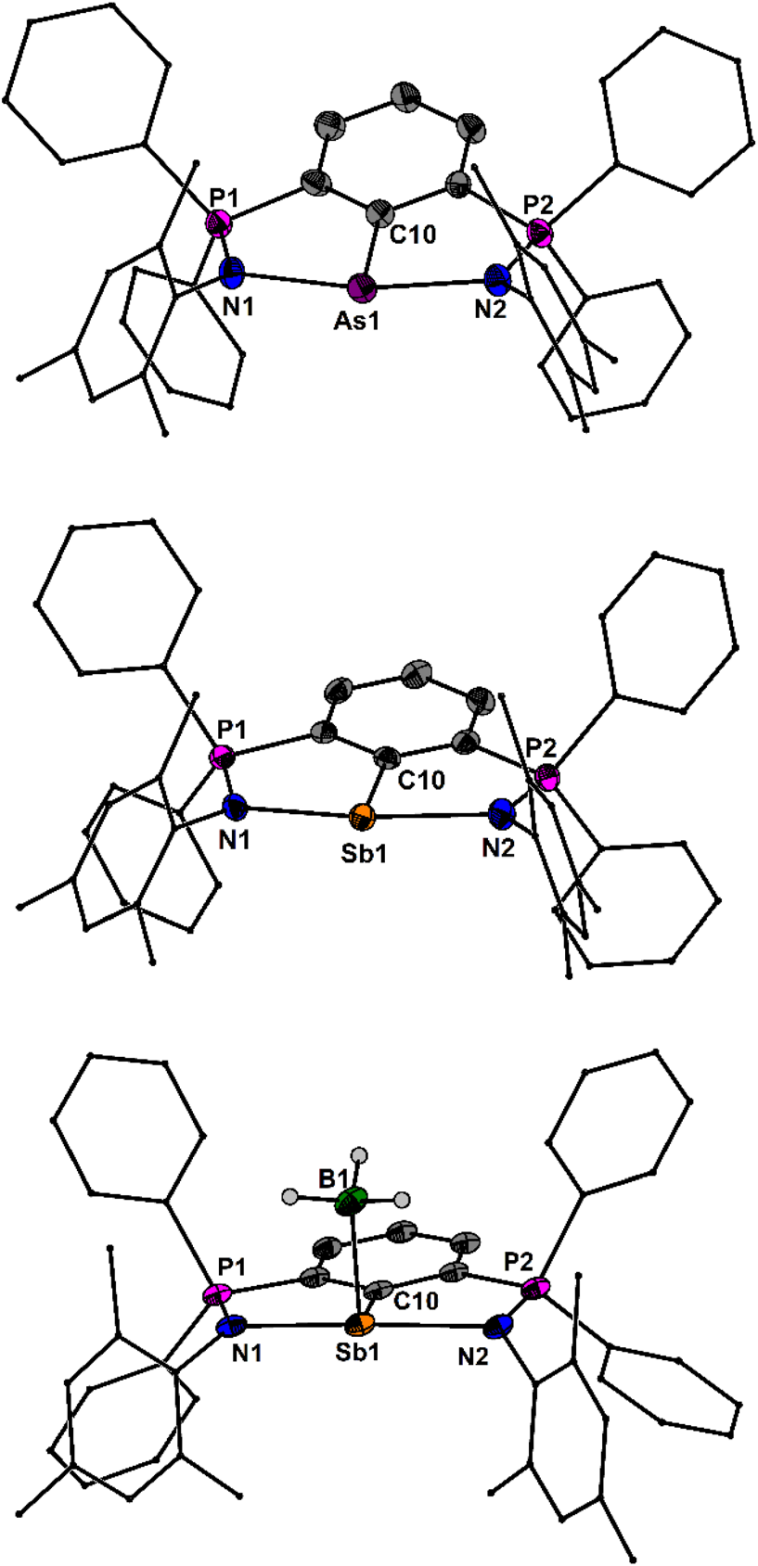
Molecular structures of 5As, 5Sb and 5Sb·BH_3_ showing 50% probability ellipsoids and the essential atomic numbering scheme. Selected bond lengths [Å] for 5As: P1–N1 1.592(3), P2–N2 1.586(3), As1–N1 2.242(3), As1–N2 2.271(3), As1–C10 1.900(4). Selected bond lengths [Å] for 5Sb: P1–N1 1.610(3), P2–N2 1.600(3), Sb1–N1 2.273(3), Sb1–N2 2.312(3), Sb1–C10 2.155(3). Selected bond lengths [Å] for 5Sb·BH_3_: P1–N1 1.590(3), P2–N2 1.585(3), Sb1–N1 2.337(3), Sb1–N2 2.361(3), Sb1–C10 2.163(3), Sb1–B1 2.293(4).

The molecular structures of 5As, 5Sb and 5Sb·BH_3_ are shown in Fig. 4. The As–N bond lengths of 5As (2.242(3), 2.271(3) Å) are longer than those of the arylchloroarsenium ion [4As]^+^ (2.122(3), 2.114(3) Å). The Sb–N bond lengths of the arylstibinidene 5Sb (2.273, 2.272(6) Å) are between those of the arylantimony dichloride 3Sb (2.286(1), 2.326(1) Å) and the arylchlorostibenium ion [4Sb]^+^ (2.226(5), 2.272(6) Å). The Sb–N bond lengths of the donor acceptor complex 5Sb·BH_3_ (2.337(3), 2.361(3) Å) are substantially longer, which suggests a lower Lewis acidity than in the aforementioned antimony compounds. This is also reflected in the short P–N bond lengths of 5Sb·BH_3_ (1.585(3), 1.590(3) Å). Notably, the Sb–N bond lengths of the 2,6-bis[N-2′,6'-(dimethylphenyl)ketimino]stibinidene (2.352(3), 2.346(3) Å) are in between those of 5Sb and 5Sb·BH_3_. The donor acceptor Sb–B bond of 5Sb·BH_3_ (2.293(4) Å) compares well with the sum of covalence radii. The ^31^P NMR resonances of 5Sb, 5Sb·BH_3_ and 5Bi show little variance at *δ* = 10.8, 16.0 and 8.8 ppm.

**Scheme 8:**
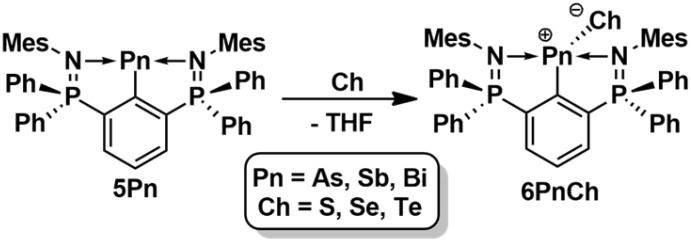
Synthesis of 6PnCh (Pn = As, Sb, Bi; Ch = S, Se, Te).

The reaction of the arylarsinidene 5As, arylstibinidene 5Sb and the arylbismuthinidene 5Bi with sulfur, selenium and tellurium led to the formation of arylarsinidene chalcogenides, 2,6-(Ph_2_PNMes)_2_C_6_H_3_AsCh (6AsCh; Ch = S, Se), arylstibinidene chalcogenides, 2,6-(Ph_2_PNMes)_2_C_6_H_3_SbCh (6SbCh; Ch = S, Se, Te), and arylbismuthinidene chalcogenides, (Ph_2_PNMes)_2_C_6_H_3_BiCh (6BiCh; Ch = S, Se, Te), which were isolated as yellow (6AsS, 6AsSe, 6SbS, 6SbSe and 6BiS, 6BiSe) and red (6SbTe and 6BiTe) crystalline needles in nearly quantitative yield based on ^31^P-NMR (Scheme 8).

**Fig. 5:**
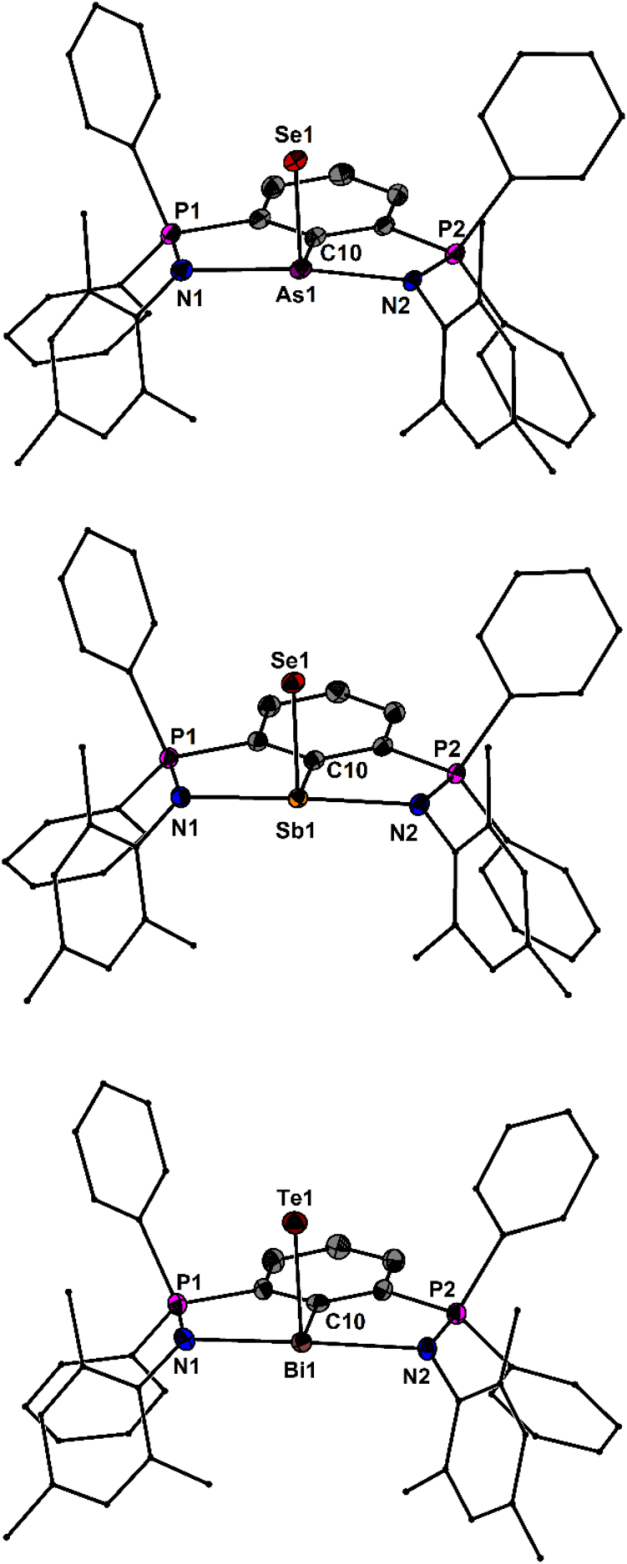
Molecular structures of 6AsSe, 6SbSe and 6BiTe showing 50% probability ellipsoids and the essential atomic numbering scheme of the core region. Substituents are shown as wireframes for clarity. Selected bond lengths [Å] for 6AsSe: P1–N1 1.569(2), P2–N2 1.605(2), As1–N1 2.376(2), As1–N2 2.126(2), As1–C10 2.000(2), Se1–As1 2.310(1). Selected bond lengths [Å] for 6SbSe: P1–N1 1.584(1), P2–N2 1.597(1), Sb1–N1 2.386(1), Sb1–N2 2.307(1), Sb1–C10 2.189(1), Sb1–Se1 2.4661(2). Selected bond lengths [Å] for 6BiTe: P1–N1 1.582(4), P2–N2 1.586(4), Bi1–N1 2.499(4), Bi1–N2 2.488(3), Bi1–C10 2.306(4), Bi1–Te1 2.7614(4).

The molecular structures of 6AsSe, 6SbSe and 6BiTe are shown in Fig. 5. The As–Se bond length of 6AsSe (2.310(1) Å) is marginally smaller than that of 2,6-(Me_2_NCH_2_)_2_C_6_H_3_AsSe (2.351(3) Å).^45^ The Sb–Se bond length of 6SbSe (2.466(1) Å) is marginally larger those in the electronically stabilized species 2-[(2′,4′-*i*-Pr_2_C_6_H_4_)NC(H)]-6-[(2′′,4′′-*i*-Pr_2_C_6_H_4_)NHC(H)]C_6_H_3_SbSe (2.437(1) Å),^14^, [2,6-(Me_2_NCH_2_)C_6_H_3_]SbSe (2.440(1) Å) and 2,6-[2′,6′-Ph_2_C_6_H_3_NC(Me)]_2_C_6_H_3_SbSe (2.433(1) Å)^46^ and substantially longer than in the kinetically stabilized M^S^Fluind*SbSe (2.372(1) Å).^41^ The Bi–Te bond length of 6BiTe (2.7614(4) Å) is the longest formal double bond between different elements ever reported. It is considerably shorter than the single bond lengths of Et_2_BiTeEt (2.912(1) Å)^47^ and 2-[(2′,4′-*i*-Pr_2_C_6_H_4_)NC(H)]C_6_H_3_Bi(TePh)_2_ (2.895(1) Å).^48^ The ^31^P NMR chemical shifts of 6AsCh, 6SbCh and 6BiCh are in the range from *δ* = 12.9 to 13.2 ppm, from *δ* = 14.6 to 15.6 ppm and from *δ* = 18.2 to 22.8 ppm, respectively. Despite all efforts, no ^77^Se NMR signals were found for the selenides 6AsSe, 6SbSe and 6BiSe after scanning the range from −3000 to +6000 ppm for several hours. The ^125^Te NMR spectra of the tellurides 6SbTe and 6BiTe gave rise to singlets at *δ* = −274.8 and −193.1 ppm.

### DFT computations

In an effort to unravel the nature of the formal heavy As=Ch, Sb=Ch and Bi=Ch double bonds in the arylpnictinidene chalcogenides, 2,6-(Ph_2_PNMes)_2_C_6_H_3_AsCh (6AsCh; Ch = S, Se, Te), 2,6-(Ph_2_PNMes)_2_C_6_H_3_SbCh (6SbCh; Ch = S, Se, Te), (Ph_2_PNMes)_2_C_6_H_3_BiCh (6BiCh; Ch = S, Se, Te) we performed complementary bonding analyses.^49^

**Fig. 6:**
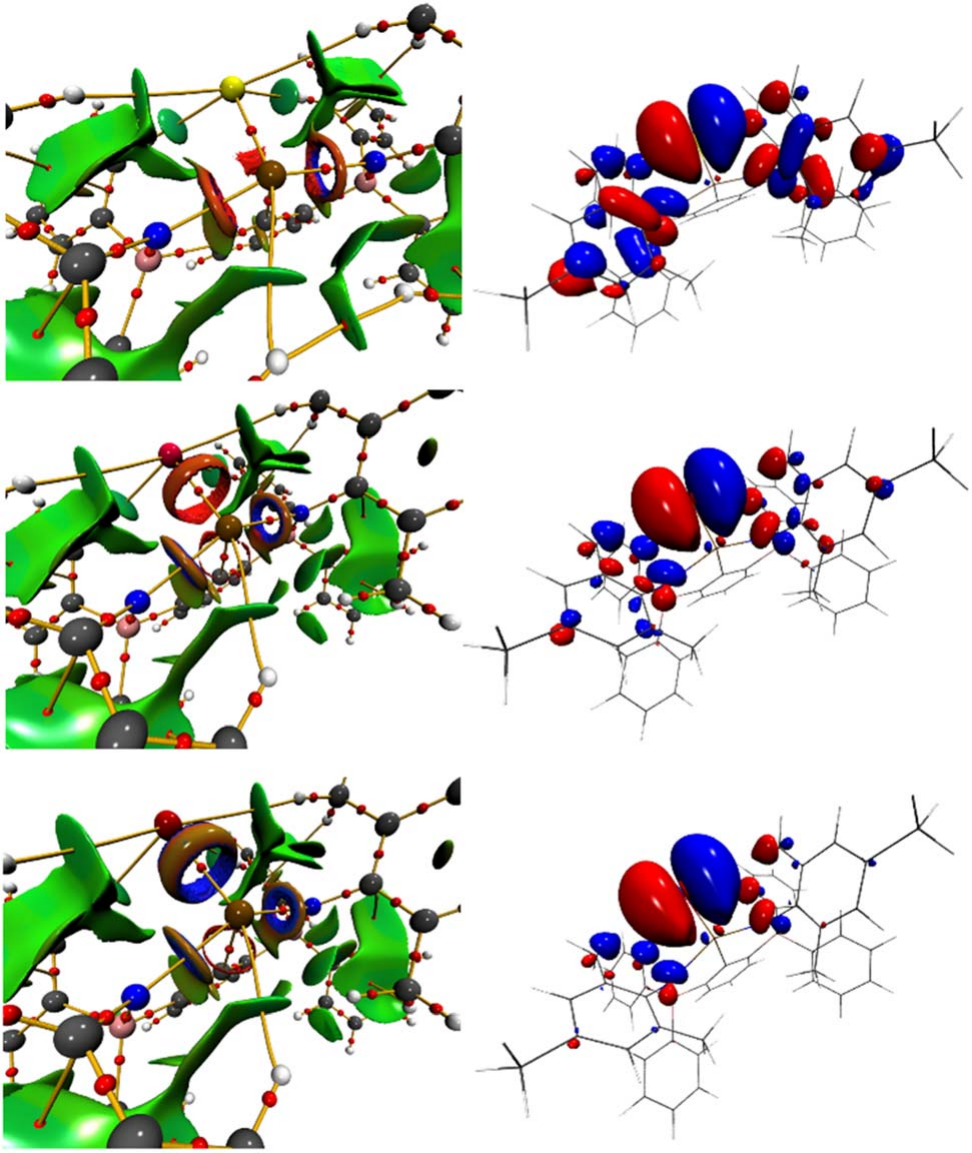
AIM molecular graphs of 6AsS (top left), 6SbSe (middle left) and 6BiTe (bottom left) with bond critical points as red spheres and bond paths in orange as well as NCI *iso*-surfaces at s(*r*) = 0.5 colour coded with sign(λ2)*ρ* in a. u. Blue surfaces refer to attractive forces and red to repulsive forces. Green indicates weak interactions. Respective HOMO–1 of 6AsS (top right), 6SbSe (middle right) and 6BiTe (bottom right) at *iso*-surfaces at s(*r*) = +/− 0.02 (blue/red).

The electron density-based Atoms-In-Molecule (AIM)^50^ approach reveals decreasing electron density values (*ρ*_bcp_) at the bond critical point with increasing size of the chalcogen of 0.86/0.70/0.65 e Å^−3^ (AsS/SbS/BiS), 0.72/0.61/0.57 e Å^−3^ (AsSe/SbSe/BiSe) and 0.56/0.50/0.47 e Å^−3^ (AsTe/SbTe/BiTe) with slightly higher values obtained for 6AsCh. Interestingly, the Laplacians (∇^2^*ρ*_bcp_) are increasing from AsS to AsTe, while a steady decrease is observed for the heavier congeners SbS to SbTe as well as from BiS to BiTe indicating an increase in ionic contributions for 6AsCh and a decrease within 6SbCh and 6BiCh. However, the kinetic energy over electron density ratios (*G*/*ρ*_bcp_) are slightly steadily decreasing also from AsS to AsTe indicating a slight decrease in ionic bonding contributions also for 6AsCh (Tables S2–S4†). On the other side, the total energy over electron density values (H/*ρ*_bcp_) are getting closer to zero from S to Te, which in turn indicates a simultaneous slight increase in polarity/iconicity also for the heavier 6SbCh and 6BiCh. Such behavior has also been described before for Si–O bonds.^51^ Despite the rather low values of AIM derived parameters, the delocalization indices range from 1.19 to 1.26 for AsCh, 1.24 to 1.26 for SbCh and from 1.29 to 1.30 for BiCh, supporting the formulation of formal Ch=Pn double bonds (Table S5†). The Wiberg Bond indices (WBI, AsCh: 1.11–1.23, SbCh: 1.19–1.21; BiCh: 1.21–1.25) are lower compared to the recently reported kinetically stabilized stibinidene chalcogenides M^S^Flunid*SbCh (ranging from 1.69 to 1.82)^41^ presumably owing to the electronic stabilization of the N-atoms in 6PnCh. Inspection of the Non-covalent interaction (NCI) index clearly indicates the covalent bonding contributions along the AsCh, SbCh and BiCh bond axis, which are visibly more pronounced within the AsS interactions already indicated by the AIM parameters (Fig. 6 and S129–S131†).^52^ Furthermore, the trend of the AIM parameters for the series 6PnCh augment the values reported for the heaviest members of SbO and BiO bonds being prepared only recently.^53,54^ Following the trend, the SbO and BiO bcps show the highest values of *ρ*_bcp_ and ∇^2^*ρ*_bcp_ indicating a higher degree of polarity in the oxygen based SbO and BiO double bonds. Extending the analysis of *ρ*_bcp_, ∇^2^*ρ*_bcp_ and the ellipticity (*ε*) along the entire PnCh bond lengths reveals a substantial difference between the PnS bonds and the heavier PnSe and PnTe bonds. The electron densities along the AsS, SbS and BiS bonds are asymmetric (Fig. S132†), reminiscent of the reported AsO, SbO and SbO bonds,^53^ whereas the distribution of *ρ* becomes more symmetric for the heavier chalcogens Se and Te (Fig. S132†). The Laplacians of *ρ* show a clear minimum near the sulfur in all PnS bonds, again similar to the respective heavier PnO bonds,^53^ which is absent in the PnSe and PnTe bonds. However, slight differences are observed between the AsSe and AsTe bonds compared to the heavier analogues. For AsSe, ∇^2^*ρ*_bcp_ is almost symmetrically distributed, whereas a small minimum is formed in the location of the selenium for SbSe and BiSe. For AsTe, ∇^2^*ρ*_bcp_ a small minimum is observed in the location of the arsenic, whereas for SbTe the distribution is rather symmetric and for BiTe a small minimum is observed in the direction of the tellurium (Fig. S132†). For the ellipticity, the highest values are observed close to the arsenic in the AsCh bonds, which decrease towards the bond critical point and reach a minimum after the bcp before increasing towards the chalcogens. For the SbCh and BiCh bonds, the overall ellipticities are substantially smaller, with the SbCh bonds showing the same trend as the AsCh bonds. For the BiCh bonds, the ellipticities reach a minimum before the bcp (closer to the bismuth) and increase towards the chalcogen (Fig. S132†).

NBO/NLMO analyses of the AsCh, SbCh and BiCh bonds revealed in each case only one As–Ch, Sb–Ch and Bi–Ch bonding orbital in line with the formulation of bipolar ^+^As–Ch^–^, ^+^Sb–Ch^–^ and ^+^Bi–Ch^−^ bonds (Tables S7, S9 and S11†).^55^ Second-order perturbation theory shows LP(Ch) → LV(As/Sb/Bi), LP(Ch) → LV(C_ipso_) and LP(Ch) → σ*(As/Sb/Bi–C) donor–acceptor interactions summing up to a total of E2 = 63 to 73 kcal mol^−1^ (6AsCh), E2 = 48 to 53 kcal mol^−1^ (6SbCh) and E2 = 41 to 46 kcal mol^−1^ (6BiCh), which are higher compared to values reported for the SbO and BiO bonds.^54^ The NLMO analysis revealed that two LP_Ch NLMOs show reduced percentages of the parent NBO, indicating that the respective lone pairs are partially delocalized and make up 12.4 to 17.4% (6AsCh), 15.4 to 20.1% (6SbCh) and 20.8 to 25.5% (6BiCh) of the total AsCh/SbCh/BiCh NLMO bond orders (Tables S5, S8, S10 and S12†). The partial π-backdonation from the chalcogen is also visible in the respective HOMO–1 molecular orbitals (Fig. 6 and S136–S138†).

Additionally, EDA-NOCV^56^ analyses of the two experimentally obtained molecular structures of 6SbSe and 6BiTe were carried out considering two different bonding situations: (i) for the interaction of the formal Sb=Se and Bi=Te double bond, two neutral fragments in their respective triplet states have been applied. (ii) For the bipolar bonding situation ^+^Sb–Se^–^ and ^+^Bi–Te^–^, charged fragments (RSb^+^/RBi^+^ and Se^−^/Te^−^) in their respective doublet state have been used. The energy terms derived from the EDA-NOCV analysis are summarized in Table S13.† The bipolar bonding situation with a Sb–Se and Bi–Te single bond gives rise to Δ*E*_orb_ values which are closer to zero compared to the Δ*E*_orb_ values obtained for the double bond situation, indicating that the heavy formal double bonds are better described with a Lewis structure containing bipolar ^+^Pn–Ch^−^ bonds. As a last point, the *iso*-surface of the localized orbital locator^57^ of the π-orbitals (LOL-π), exemplified for 6BiCh, shows the same trend (Fig. S140†), clearly indicating a charge separated formulation with π-electron residing at the chalcogens, respectively.

## Conclusions

A novel NCN-pincer ligand based upon a bis(phosphine imine) substituted phenyl substituent was introduced and applied for the preparation of an electronically stabilized arylarsinidene 2,6-(Ph_2_PNMes)_2_C_6_H_3_As (5As), arylstibinidene 2,6-(Ph_2_PNMes)_2_C_6_H_3_Sb (5Sb) and arylbismuthinidene 2,6-(Ph_2_PNMes)_2_C_6_H_3_Bi (5Bi). The oxidation of 5As, 5Sb and 5Bi with sulfur, selenium and tellurium, respectively, produced the arylarsinidene chalcogenides 2,6-(Ph_2_PNMes)_2_C_6_H_3_AsCh (6AsCh, Ch = S, Se), arylstibinidene chalcogenides 2,6-(Ph_2_PNMes)_2_C_6_H_3_SbCh (6SbCh) and arylbismuthinidene chalcogenides 2,6-(Ph_2_PNMes)_2_C_6_H_3_BiCh (6BiCh) formally containing terminal As=Ch, Sb=Ch and Bi=Ch double bonds (Ch = S, Se, Te). The formal Bi=Te double bond of the arylbismuthinidene telluride 6BiTe (bond length 2.7614(4) Å) comprises the heteroatomic combination of the two heaviest main group elements excluding strongly radioactive ones. A bond analysis suggests these formal double bonds are best described as bipolar ^+^As–Ch^−^, ^+^Sb–Ch^−^ and ^+^Bi–Ch^−^ single bonds (Ch = S, Se, Te).

## Author contributions

F. M. synthesized and isolated the compounds 1, 2, [4]A (A = AlCl_4_, O_3_SCF_3_), 5Pn, 6PnCh and performed NMR and UV-vis measurements. A. S. synthesized and performed NMR and UV-vis measurements on 3Pn and [4As][As_2_OCl_5_]. J. B. conducted the X-ray diffraction measurements and structure refinement. J. B. and L. D. designed the project. E. H. performed all theoretical computations. Writing of the manuscript was done by F. M., L. D., J. B. and E. H.

## Conflicts of interest

There are no conflicts to declare.

## Supplementary Material

SC-OLF-D5SC03320A-s001

SC-OLF-D5SC03320A-s002

SC-OLF-D5SC03320A-s003

## Data Availability

Figures of NMR spectra as well as crystal and refinement data are given in the ESI.† Crystallographic information files (CIF) have been deposited with the Cambridge Crystallographic Data Centre, no. 2446710–2446720, 2457652 and 2457653. Additional results from quantum chemical calculations are given in the ESI.† The raw data that support the findings of this study are available from the corresponding authors upon reasonable request.

## References

[cit1] Dostal L. (2017). Coord. Chem. Rev..

[cit2] He M., Hu C., Wei R., Wang X.-F., Liu L. L. (2024). Chem. Soc. Rev..

[cit3] Lipshultz J. M., Li G., Radesevich A. T. (2021). J. Am. Chem. Soc..

[cit4] Moon H. W., Cornella J. (2022). ACS Catal..

[cit5] Pang Y., Nöthling N., Leutzsch M., Kang L., Bill E., van Gastel M., Reijerse E., Goddard R., Wagner L., SantaLucia D., DeBeer S., Neese F., Cornella J. (2023). Science.

[cit6] Wu M., Li H., Chen W., Wang D., Chen Y., Ye S., Tan G. (2023). Nat. Sci. Rev..

[cit7] Wu M., Li H., Chen W., Wang D., He Y., Xu L., Ye S., Tan G. (2023). Chem.

[cit8] Janssen M., Frederichs T., Olaru M., Lork E., Hupf E., Beckmann J. (2024). Science.

[cit9] Wang D., Chen W., Chen H., Chen Y., Ye S., Tan G. (2025). Nat. Chem..

[cit10] Šimon P., de Proft F., Jambor R., Růžička A., Dostál L. (2010). Angew. Chem., Int. Ed..

[cit11] Vránová I., Alonso M., Lo R., Sedlák R., Jambor R., Růžička A., de Proft F., Hobza P., Dostál L. (2015). Chem.–Eur. J..

[cit12] Vránová I., Alonso M., Jambor R., Růžička A., Erben M., Dostál L. (2016). Chem.–Eur. J..

[cit13] Pang Y., Leutzsch M., Nöthling N., Cornella J. (2020). J. Am. Chem. Soc..

[cit14] Zechovský J., Kertész E., Kremláček V., Hejda M., Mikysek T., Erben M., Růžička A., Jambor R., Benkő Z., Dostál L. (2022). Organometallics.

[cit15] Hyvl J., Yoshida W. Y., Rheingold A. L., Hughes R. P., Cain M. F. (2016). Chem.–Eur. J..

[cit16] Vránová I., Kremláček V., Erben M., Turek J., Jambor R., Růžička A., Alonso M., Dostál L. (2017). Dalton Trans..

[cit17] Hyvl J., Yoshida W. Y., Moore C. E., Rheingold A. L., Hughes R. P., Cain M. F. (2018). Polyhedron.

[cit18] Kremláček V., Hyvl J., Yoshida W. Y., Růžička A., Rheingold A. L., Turek J., Hughes R. P., Dostál L., Cain M. F. (2018). Organometallics.

[cit19] Nguyen M. T., Gabidullin B., Nikonov G. I. (2018). Dalton Trans..

[cit20] W Moon H., Wang F., Bhattacharyya K., Planas O., Leutzsch M., Nöthling N., Auer A. A., Cornella J. (2023). Angew. Chem., Int. Ed..

[cit21] Vránová I., Alonso M., Jambor R., Růžička A., Turek J., Dostál L. (2017). Chem.–Eur. J..

[cit22] Kořenkova M., Kremláček V., Erben M., Jirásko R., de Proft F., Turek J., Jambor R., Růžička A., Cisařová I., Dostál L. (2018). Dalton Trans..

[cit23] Zechovský J., Kremláček V., Erben M., Hejda M., Rychagova E., Jambor R., Růžička A., Ketkov S., Dostál L. (2022). Dalton Trans..

[cit24] Greb L., Ebner F., Ginzburg Y., Sigmund L. M. (2020). Eur. J. Inorg. Chem..

[cit25] Huang M., Li K., Zhang Z., Zhou J. (2024). Antimony Redox Catalysis. J. Am. Chem. Soc..

[cit26] Kořenkova M., Hejda M., Erben M., Jirásko R., Jambor R., Růžička A., Rychagova E., Ketkov S., Dostál L. (2019). Chem.–Eur. J..

[cit27] Hejda M., Jirásko R., Růžička A., Jambor R., Dostál L. (2020). Organometallics.

[cit28] Mato M., Bruzzese P. C., Takahashi F., Leutzsch M., Reijerse E. J., Schnegg A., Cornella J. (2023). J. Am. Chem. Soc..

[cit29] Mato M., Spinnato D., Leutzsch M., Moon H. W., Reijerse E. J., Cornella J. (2023). Nature Chem..

[cit30] Yang X., Reijerse E. J., Bhattacharyya K., Leutzsch M., Kochius M., Nöthing N., Busch J., Schnegg A., Auer A. A., Cornella J. (2022). J. Am. Chem. Soc..

[cit31] Yang X., Reijerse E. J., Nöthing N., SantaLucia D. J., Leutzsch M., Schnegg A., Cornella J. (2023). J. Am. Chem. Soc..

[cit32] Wang F., Planas O., Cornella J. (2019). J. Am. Chem. Soc..

[cit33] Pang Y., Leutzsch M., Nöthling N., Katzenburg F., Cornella J. (2021). J. Am. Chem. Soc..

[cit34] Béland V. A., Nöthing N., Leutzsch M., Cornella J. (2024). J. Am. Chem. Soc..

[cit35] Tsuruta T., Spinnato D., Moon H. W., Lentzsch M., Cornella J. (2023). J. Am. Chem. Soc..

[cit36] Ni S., Spinnato D., Cornella J. (2024). J. Am. Chem. Soc..

[cit37] Mato M., Wang F., Cornella J. (2024). Adv. Synth. Catal..

[cit38] Olaru M., Kögel J. F., Lork E., Mebs S., Vogt M., Beckmann J. (2020). Chem.–Eur. J..

[cit39] Olaru M., Rychagova E., Ketkov S., Shynkarenko Y., Yakunin S., Kovalenko M. V., Yablonsiky A., Andreev B., Beckmann J., Vogt M. (2020). J. Am. Chem. Soc..

[cit40] Meyer F., Puylaert P., Duvinage D., Hupf E., Beckmann J. (2024). Chem. Commun..

[cit41] Li X., Chen Y., Dong S., Wang D., Xu L., Zhu J., Tan G. (2025). J. Am. Chem. Soc..

[cit42] Meyer F., Kuzmera T., Lork E., Vogt M., Beckmann J. (2021). Z. Anorg. Allg. Chem..

[cit43] Vránová I., Jambor R., Růžička A., Jirásko R., Dostál L. (2015). Organometallics.

[cit44] Vránová I., Erben M., Jambor R., Růžička A., Jirásko R., Dostál L. (2016). Z. Anorg. Allgem. Chem..

[cit45] Vrána J., Jambor R., Růžička A., Lyčka A., De Proft F., Dostál L. (2013). J. Organomet. Chem..

[cit46] Jambor R., Růžička A., Lyčka A., Brus J., de Proft F., Dostál L. (2008). Organometallics.

[cit47] Heimann S., Kuczkowski A., Bläser D., Wölper C., Haack R., Jansen G., Schulz S. (2014). Eur. J. Inorg. Chem..

[cit48] Šimon P., Jambor R., Růžička A., Dostál L. (2013). Organometallics.

[cit49] Complementary Bonding Analysis, ed. Grabowsky, S., de Gruyter, Berlin, 2021

[cit50] Bader R. W. F. , Atoms in Molecules. A Quantum Theory, Cambridge University Press, Oxford U.K., 1991

[cit51] Feige F., Malaspina L. A., Kleemiss F., Kögel J. F., Ketkov S., Hupf E., Grabowsky S., Beckmann J. (2023). Dalton Trans..

[cit52] Johnson E.
R., Keinan S., Mori-Sánchez P., Contreras-García J., Cohen A. J., Yang W. (2010). J. Am. Chem. Soc..

[cit53] Lindquist-Kleissler B., Wenger J. S., Johnstone T. C. (2021). Inorg. Chem..

[cit54] Wenger J. S., Weng M., George G. N., Johnstone T. C. (2023). Nat. Chem..

[cit55] Glendening E. D., Landis C. R., Weinhold F. (2012). Wiley Interdiscip. Rev.: Comput. Mol. Sci..

[cit56] Mitoraj M. P., Michalak A., Ziegler T. (2009). J. Chem. Theory Comput..

[cit57] Schmider H. L., Becke A. D. (2000). J. Mol. Struct..

