# Supplementary material for: Nature of the heavy formal double bonds As[double bond, length as m-dash]Ch, Sb[double bond, length as m-dash]Ch and Bi[double bond, length as m-dash]Ch (Ch = S, Se, Te) in NCN-pincer supported arsinidene, stibinidene and bismuthinidene chalcogenides
Source: Chem Sci. 2025 Jul 18;16(35):16232–9. doi: 10.1039/d5sc03320a (PMC12352694; doi:10.1039/d5sc03320a)
Supplement: SC-016-D5SC03320A-s002 [file SC-016-D5SC03320A-s002.pdf]

Supplementary Information (SI) for Chemical Science.
This journal is © The Royal Society of Chemistry 2025

Supporting Information

Experimental Section 2
NMR Spectra 18
UV-vis Spectra 69
X-ray crystallography 82
DFT Computations 89

Experimental Section

General information

Chemicals and solvents were obtained commercially (e.g. Sigma Aldrich) and were used unchanged. Dry solvents, such as tetrahydrofuran (THF), toluene, dichloromethane (DCM) and *n*-hexane, were collected from a SPS800 MBRAUN solvent purification system, degassed and stored over 3 Å molecular sieves before usage. Et_2O was freshly distilled over sodium/benzophenone under argon and stored over 3 Å molecular sieves. 1,2-difluorobenzene (1,2-DFB), were degassed and dried directly over 3 Å molecular sieves. Deuterated solvents for NMR measurements were degased and dried directly over 3 Å molecular sieves. The corresponding ^{1}H,^{11}B, ^{13}C, ^{31}P and ^{125}Te NMR measurements were executed (if not stated otherwise) at room temperature by using a Bruker Avance Neo 600 spectrometer. These were referenced to tetramethylsilane (^{1}H, ^{13}C), BF_3-Et_2O (15% in $CDCl_3$, ^{11}B), phosphoric acid (85 % in water, ^{31}P) and dimethyltelluride (^{125}Te) respectively. Chemical shifts are illustrated in parts per million (ppm). The assignments of the ^{1}H and ^{13}C signals were made in accordance to 2D NMR methods (COSY, HSQC, HMBC). High resolution mass spectroscopy was performed with a BRUKER IMPACT II spectrometer at a flow rate of 3 µL·min−1 in stated solvent solutions. UV-vis absorption spectra were recorded in stated concentrations in THF on a VWR UV-1600PC spectrophotometer.

Synthesis of 2,6-($Ph_2PNMes)_2C_6H_3Br$ (1). Mesityl azide (1.23 g, 7.61 mmol, 2 eq.) was added to a solution of 2,6-bis(diphenylphosphino)-1-bromobenzene (2.00 g, 3.81 mmol, 1 eq.) in dry toluene (20 mL) and was stirred for 1 h at room temperature and for 3 days at 100 °C. The solvent was removed under reduced pressure and the residue was washed with cold *n*-pentane (3 × 20 mL). The precipitate was separated from the organic phase via filtration and dried under vacuum. To optimize the yield the washing solution was stored at –40 °C for 24 h to crystallize the remaining desired product from the collected organic phases. The precipitates were

combined and recrystallized from *n*-pentane, EtOH or MeOH at –40 °C. The desired product was obtained as an air and moisture stable pale brownish solid. Suitable crystals for X-ray diffraction measurements were obtained by recrystallization from hot *n*-hexane.

Yield: 2.53 g (3.20 mmol, 84 %). **H NMR (600 MHz, CD_2Cl_2):** δ = 7.64 (dd, $^3J(^{31}P\text{-}^1H)$ = 11.9 Hz, $^3J(^1H\text{-}^1H)$ = 7.6 Hz, 8H, H_{11}), 7.55 (t, $^3J(^1H\text{-}^1H)$ = 7.5 Hz, 4H, H_{13}), 7.45 (td, $^4J(^{31}P\text{-}^1H)$ = 7.7 Hz, $^3J(^1H\text{-}^1H)$ = 3.0 Hz, 8H, H_{12}), 7.40 (d, $^3J(^1H\text{-}^1H)$ = 11.5 Hz, 2H, H_3), 7.29 (t, $^3J(^1H\text{-}^1H)$ = 7.7 Hz, 1H, H_4), 6.72 (s, 4H, H_{22}), 2.23 (s, 6H, H_{25}), 1.90 (s, 12H, H_{24}) ppm. **$^{13}C\{^1H\}$ NMR (150.9 MHz, CD_2Cl_2):** δ = 144.3 (s, C_{20}), 138.1 – 137.8 (m, C_3), 137.3 (d, $^1J(^{31}P\text{-}^{13}C)$ = 4.9 Hz, C_2), 133.3 (s, C_{23}), 132.6 (s, C_1), 132.0 (d, $^2J(^{31}P\text{-}^{13}C)$ = 9.6 Hz, C_{11}), 131.6 (d, $^2J(^{31}P\text{-}^{13}C)$ = 6.9 Hz, C_{21}), 131.1 (s, C_{13}), 128.4 (s, C_{22}), 128.4 (s, C_{12}), 128.3 (s, C_{10}), 127.3 – 126.9 (m, C_4), 20.6 (s, C_{24}), 20.4 (s, C_{25}) ppm.**$^{31}P\{^1H\}$ NMR (243 MHz, CD_2Cl_2):** δ = –8.9 (s) ppm. **Mp**: 149 °C. **HRMS (ESI, positive mode, MeOH):** *m/z* 791.23141 $[M+H]^+$, Err. 0.40 mDa, 396.11918 $[M+2H]^{2+}$, Err. 0.17 mDa.

Synthesis of 2,6-($Ph_2PNMes)_2C_6H_3MgCl\cdot THF$ (2). *i*Pr-MgCl·LiCl (27.5 mg, 0.189 mmol, 1.3 M in THF, 1 eq.) was added to a solution of **1** (150 mg, 0.189 mmol, 1 eq.) in dry and degassed Et_2O or THF (10 mL). The reaction mixture was stirred for 5 min at room temperature. The desired product **2** is labile towards extended time of vacuum therefore drying was immediately stopped after the solvent was evaporated. Even adding higher boiling solvents like dioxane for stabilizing coordination purposes were not successful. Suitable crystals for single crystal X-ray diffraction measurements were obtained by recrystallization from THF/*n*-hexane.

^1H NMR (600 MHz, CD_2Cl_2): δ = 7.82 – 7.65 (m, 6H), 7.47 (t, $J(^1H\text{-}^1H)$ = 7.6 Hz, 4H), 7.40 – 7.36 (m, 12H), 7.35 (td, $J(^1H\text{-}^1H)$ = 7.7, $J(^1H\text{-}^1H)$ = 2.3 Hz, 12H), 7.24 – 7.15 (m, 1H), 6.51 (s, 4H), 2.07 (s, 6H), 1.80 (s, overlapped with THF signal, 12H) ppm. **$^{13}C\{^1H\}$ NMR**

(150.9 MHz, CD_2Cl_2): δ = 193.6 (t, $^3J(^{31}P-^{13}C)$ = 38.1 Hz), 145.1 (d, $J(^{31}P-^{13}C)$ = 5.3 Hz), 136.5 (d, $J(^{31}P-^{13}C)$ = 21.2 Hz), 135.8 (s), 135.6 (s), 134.0 (d, $J(^{31}P-^{13}C)$ = 8.3 Hz), 133.0 (d, $J(^{31}P-^{13}C)$ = 23.6 Hz), 131.5 (s), 130.4 (s), 128.8 (s), 128.4 (d, $J(^{31}P-^{13}C)$ = 11.1 Hz), 126.0 (t, $J(^{31}P-^{13}C)$ = 13.1 Hz), 26.0 (s, overlapped with THF signal), 20.8 (s) ppm. **$^{31}P\{^1H\}$ NMR (243 MHz, CD_2Cl_2):** δ = 10.0 (s) ppm. **HRMS (ESI, positive mode, DCM/MeCN 1:10):** No mass cluster observed, presumably due to the sensitivity in vacuum.

Synthesis of 2,6-$(Ph_2PNMes)_2C_6H_3PCl_2$ (3P). *i*Pr-MgCl·LiCl (0.485 mL, 0.631 mmol, 1.3 M in THF, 1 eq.) was added to a solution of **1** (500 mg, 0.631 mmol, 1 eq.) in dry and degassed Et_2O (10 mL). The reaction mixture was stirred for 5 min at room temperature. PCl_3 (26.0 mg, 0.189 mmol, 1 eq.) was added to the reaction solution at –78 °C. The reaction mixture was allowed to warm up to room temperature within 30 min. The solvent was decanted off. The residue was washed with dry and degassed Et_2O (2 × 15 mL) and *n*-hexane (2 × 15 mL) to obtain a pale yellow solid. The desired product is labile to vacuum. Even adding high boiling coordinating solvents like dioxane for stabilizing coordination purposes were not successful. For this reason, drying was carefully performed until a pale solid was obtained. The compound decomposes in solution unselectively within a few days. Even when stored as a solid under inert conditions the compound decomposes unselectively over a few weeks.

Yield: 328.9 mg (0.404 mmol, 64 %). **^1H NMR (600 MHz, CD_2Cl_2):** δ = 8.28 (s, 1H, H_4), 8.15 – 8.08 (m, 2H, H_3), 7.87 – 7.80 (m, 4H, H_{11a}), 7.74 (t, $^3J(^1H-^1H)$ = 7.5 Hz, 2H, H_{13b}), 7.69 (t, $^3J(^1H-^1H)$ = 7.5 Hz, 2H, H_{13a}), 7.60 – 7.51 (m, 8H, H_{12a}+H_{12b}), 7.44 – 7.38 (m, 4H, H_{11b}), 6.68 (s, 4H, H_{22}), 2.14 (s, 6H, H_{25}), 1.89 – 1.82 (m, 12H, H_{24}) ppm. **$^{13}C\{^1H\}$ NMR (150.9 MHz, CD_2Cl_2):** δ = 156.2 – 154.7 (dt, $^1J(^{31}P-^{13}C)$ = 39.4 Hz, $^2J(^{31}P-^{13}C)$ = 10.6 Hz, C_1), 138.6 (d, $^2J(^{31}P-^{13}C)$ = 14.3 Hz, C_3), 137.2 (s, C_{23}), 136.06 (s, C_{21}), 135.0 (s, C_4), 134.9 (s, C_{11a}), 134.7 (s, C_{13a}+C_{13b}), 134.0 (d, $^1J(^{31}P-^{13}C)$ = 8.2 Hz, C_{20}), 132.9 (d, $^2J(^{31}P-^{13}C)$ = 9.5 Hz, C_{11b}), 131.9

(dd, $^{1}J(^{31}P\text{-}^{13}C)$ = 118.9 Hz, $^{3}J(^{31}P\text{-}^{13}C)$ = 11.8 Hz, C_2), 130.2 (s, C_{22}), 130.1 (d, $^{3}J(^{31}P\text{-}^{13}C)$ = 7.3 Hz, C_{12a}), 130.1 (d, $^{3}J(^{31}P\text{-}^{13}C)$ = 7.9 Hz, C_{12b}), 128.3 (dd, $^{1}J(^{31}P\text{-}^{13}C)$ = 89.9, $^{4}J(^{31}P\text{-}^{13}C)$ = 6.0 Hz, C_{10}), 121.6 (d, $^{1}J(^{31}P\text{-}^{13}C)$ = 90.9 Hz, C_{10b}), 25.9 (s, C_{24}), 21.0 (s, C_{25}) ppm. **$^{31}P\{^{1}H\}$ NMR (243 MHz, CD_2Cl_2):** δ = 100.0 (t, $^{2,3}J(^{31}P\text{-}^{31}P)$ = 46.1 Hz), 19.8 (t, $^{2,3}J(^{31}P\text{-}^{31}P)$ = 46.1 Hz) ppm. **HRMS (ESI, positive mode, DCM/MeCN):** *m/z* 777.28988 [M-2Cl+OH+H_2O]$^+$, Err. 0.63 mDa, *m/z* 759.27994 [M-2Cl+OH]$^+$, Err. 1.81 mDa.

Synthesis of 2,6-($Ph_2PNMes)_2C_6H_3AsCl_2$ (3As). *i*Pr-MgCl·LiCl (0.485 mL, 0.631 mmol, 1.3 M in THF, 1 eq.) was added to a solution of **1** (500 mg, 0.631 mmol, 1 eq.) in dry and degassed Et_2O (10 mL). The reaction mixture was stirred for 5 min at room temperature. $AsCl_3$ (34.34 mg, 0.189 mmol, 1 eq.) and $AlCl_3$ (25.2 mg, 0.189 mmol, 1 eq.) were added to the reaction mixture at room temperature. The solvent was decanted off. The residue was washed with dry and degassed Et_2O (2 × 15 mL) and *n*-hexane (2 × 15 mL) to obtain a beige solid. The desired product is stable towards moisture and oxygen, but should be stored under inert conditions to prevent minimal decomposition. Suitable crystals for single crystal x-ray diffraction measurements were obtained by recrystallization from THF/*n*-hexane.

Yield: 509.1 mg (0.593 mmol, 94 %). **^{1}H NMR (600 MHz, $CDCl_3$):** δ = 8.53 (m, 1H, H_4), 8.12 (dd, $^{2}J(^{31}P\text{-}^{1}H)$ = 11.2 Hz, $^{4}J(^{1}H\text{-}^{1}H)$ = 7.6 Hz, 2H, H_3), 7.71 – 7.66 (m, 4H, H_{13}), 7.67 – 7.60 (m, 8H, H_{11}), 7.55 (td, $^{3}J(^{1}H\text{-}^{1}H)$ = 7.8 Hz, $^{3}J(^{1}H\text{-}^{1}H)$ = 3.4 Hz, 8H, H_{12}), 6.68 (s, 4H, H_{22}), 2.16 (s, 6H, H_{25}), 1.69 (s, 12H, H_{24}) ppm. **$^{13}C\{^{1}H\}$ NMR (150.9 MHz, $CDCl_3$):** δ = 158.2 (t, $^{2}J(^{31}P\text{-}^{13}C)$ = 10.4 Hz, C_1), 138.6 (dd, $^{2}J(^{31}P\text{-}^{13}C)$ = 14.7 Hz, $^{4}J(^{31}P\text{-}^{13}C)$ = 3.1 Hz, C_3), 136.6 (s, $C_{q\text{-}Mes}$), 135.4 (s, $C_{q\text{-}Mes}$), 135.1 (t, $^{3}J(^{31}P\text{-}^{13}C)$ = 12.5 Hz, C_4), 134.2 (s, $C_{q\text{-}Mes/20?}$), 134.1 (s, C_{13}), 133.4 (d, $^{2}J(^{31}P\text{-}^{13}C)$ = 12.3 Hz, C_{11}), 132.7 (d, $^{1}J(^{31}P\text{-}^{13}C)$ = 12.2 Hz, C_2), 129.9 (s, C_{22}), 129.8 (d, $^{3}J(^{31}P\text{-}^{13}C)$ = 12.8 Hz, C_{12}), 20.8 (s, C_{24}), 20.0 (s, C_{25}) ppm. **$^{31}P\{^{1}H\}$ NMR (243 MHz,**

$CDCl_3$): δ = 21.1 (s) ppm. **Mp.:** 147 °C. **HRMS (ESI, positive mode, MeCN):** *m/z* 821.19414 $[M\text{-}Cl]^+$, Err. 1.56 mDa.

Synthesis of 2,6-($Ph_2PNMes)_2C_6H_3SbCl_2$ (3Sb). *i*Pr-MgCl·LiCl (0.485 mL, 0.631 mmol, 1.3 M in THF, 1 eq.) was added to a solution of **1** (500 mg, 0.631 mmol, 1 eq.) in dry and degassed Et_2O (10 mL). The reaction mixture was stirred for 5 min at room temperature. $SbCl_3$ (64.8 mg, 0.284 mmol, 1.5 eq.) was added to the reaction mixture at room temperature. The solvent was decanted off. The residue was washed with dry and degassed Et_2O (2 × 15 mL) and *n*-hexane (2 × 15 mL) to obtain an almost colorless solid. The desired product is stable towards moisture and oxygen, but should be stored under inert conditions to prevent minimal decomposition. Suitable crystals for single crystal x-ray diffraction measurements were obtained by recrystallization from 1,2-difluorobenzene/*n*-hexane or acetone/*n*-hexane.

Yield: 519.8 mg (0.574 mmol, 91 %). **^{1}H NMR (600 MHz, $CDCl_3$):** δ = 8.43 (s, 1H, H_4), 7.95 (dd, $^3J(^{31}P\text{-}^1H)$ = 11.4 Hz, $^3J(^1H\text{-}^1H)$ = 7.4 Hz, 2H, H_3), 7.74 – 7.61 (m, 12H, H_{11}+H_{13}), 7.53 (td, $^3J(^1H\text{-}^1H)$ = 7.8 Hz, $^5J(^1H\text{-}^1H)$ = 3.2 Hz, 8H, H_{12}), 6.67 (s, 4H, H_{22}), 2.15 (s, 6H, H_{25}), 1.76 (s, 12H, H_{24}) ppm. **$^{13}C\{^1H\}$ NMR (150.9 MHz, $CDCl_3$):** δ = 165.8 (t, $^2J(^{31}P\text{-}^{13}C)$ = 12.2 Hz, C_1), 139.0 (d, $^2J(^{31}P\text{-}^{13}C)$ = 14.9 Hz, C_3), 136.7 (s, C_{20}), 135.0 (s, C_{21}), 134.9 (s, C_{23}), 134.3 (d, $^1J(^{31}P\text{-}^{13}C)$ = 13.5 Hz, C_2), 133.7 (s, C_4), 133.6 (s, C_{11}+C_{13}), 130.0 (s, C_{22}), 129.5 (d, $^3J(^{31}P\text{-}^{13}C)$ = 12.4 Hz, C_{12}), 126.1 (d, $^1J(^{31}P\text{-}^{13}C)$ = 90.9 Hz, C_{10}), 20.7 (s, C_{25}), 20.4 (s, C_{24}) ppm. **$^{31}P\{^1H\}$ NMR (243 MHz, $CDCl_3$):** δ = 22.9 (s) ppm. **Mp**: Decomposition at 254 °C. **HRMS (ESI, positive mode, DCM/MeCN 1:10):** *m/z* 867.17790 $[M\text{-}Cl]^+$, Err. 0.02 mDa.

Synthesis of 2,6-($Ph_2PNMes)_2C_6H_3BiCl_2$ (3Bi). *i*Pr-MgCl·LiCl (0.485 mL, 0.631 mmol, 1.3 M in THF, 1 eq.) was added to a solution of **1** (500 mg, 0.631 mmol, 1 eq.) in dry and degassed Et_2O (10 mL). The reaction mixture was stirred for 5 min at room temperature. $BiCl_3$

(89.6 mg, 0.284 mmol, 1.5 eq.) was added to the reaction mixture at room temperature. The solvent was decantated off. The residue was washed with dry and degassed Et_2O (2 × 15 mL) and *n*-hexane (2 × 15 mL) to obtain an almost colorless solid. The desired product is stable towards moisture and oxygen, but should be stored under inert conditions to prevent minimal decomposition. Suitable crystals for single crystal x-ray diffraction measurements were obtained by recrystallization from DCM/*n*-hexane.

Yield: 382.0 mg, 0.385 mmol, 61 %. **^{1}H NMR (600 MHz, $CDCl_3$):** δ = 7.88 (dd, $^{3}J(^{31}P-^{1}H)$ = 11.5 Hz, $^{3}J(^{1}H-^{1}H)$ = 7.9 Hz, 8H, H_{11}), 7.75 (dd, $^{3}J(^{31}P-^{1}H)$ = 11.8 Hz, $^{3}J(^{1}H-^{1}H)$ = 7.6 Hz, 2H, H_3), 7.67 – 7.61 (m, 1H, H_4), 7.57 (t, $^{3}J(^{1}H-^{1}H)$ = 7.5 Hz, 4H, H_{13}), 7.46 (td, $^{3}J(^{1}H-^{1}H)$ = 7.7 Hz, $^{4}J(^{31}P-^{1}H)$ = 3.0 Hz, 8H, H_{12}), 6.62 (s, 4H, H_{22}), 2.14 (s, 6H, H_{25}), 2.00 (s, 12H, H_{24}) ppm. **$^{13}C\{^{1}H\}$ NMR (150.9 MHz, $CDCl_3$):** δ = 224.1 – 223.3 (m, C_1), 140.9 (dd, $^{1}J(^{31}P-^{13}C)$ = 127.7 Hz, $^{3}J(^{31}P-^{13}C)$ = 13.8 Hz, C_2), 140.9 (dd, $^{2}J(^{31}P-^{13}C)$ = 17.0 Hz, $^{4}J(^{31}P-^{13}C)$ = 2.6 Hz, C_3), 137.9 (d, $^{1}J(^{31}P-^{13}C)$ = 5.2 Hz, C_{20}), 137.2 (d, $^{4}J(^{31}P-^{13}C)$ = 5.3 Hz, C_{23}), 134.2 (d, $^{2}J(^{31}P-^{13}C)$ = 10.1 Hz, C_{11}), 133.0 (d, $^{2}J(^{31}P-^{13}C)$ = 3.3 Hz, C_{21}), 132.3 (d, $^{4}J(^{31}P-^{13}C)$ = 2.8 Hz, C_{13}), 130.5 (d, $^{1}J(^{31}P-^{13}C)$ = 87.8 Hz, C_{10}), 129.6 (d, $^{3}J(^{31}P-^{13}C)$ = 2.7 Hz, C_{22}), 128.8 (d, $^{3}J(^{31}P-^{13}C)$ = 12.1 Hz, C_{12}), 126.8 (t, $^{3}J(^{31}P-^{13}C)$ = 3.4 Hz, C_4), 21.5 (s, C_{24}), 20.5 (s, C_{25}) ppm. **$^{31}P\{^{1}H\}$ NMR (243 MHz, $CDCl_3$):** δ = 37.6 (s) ppm. **Mp:** Decomposition at 224 °C. **HRMS (ESI, positive mode, MeCN):** *m/z* 955.25496 $[M-Cl]^{+}$, Err. -0.46 mDa.

Synthesis of [2,6-($Ph_2PNMes)_2C_6H_3PnCl$][OTf] ([4Pn]OTf). The general procedure to synthesize [**4Pn**][OTf] was analogue with every compound from the group 15 elements.

Method A (preferred option): Trimethylsilyl trifluoromethanesulfonate (1 eq.) was added to a solution of **3Pn** (75 mg, 1 eq.) in 3 mL anhydrous and degassed DCM at 0 °C. The reaction was finished instantly at room temperature. The solvent was removed under vacuum and the residue was washed with anhydrous *n*-pentane (4 × 2 mL). The solid was dried under vacuum.

Method B: $AlCl_3$ (1 eq.) was added to a solution of **3Pn** (15 mg, 1 eq.) in 0.5 mL anhydrous and degassed CD_2Cl_2 in a *J*-Young NMR tube at room temperature. The reaction mixture was placed in a sonication bath several times. Conversion was examined by ^{31}P NMR. The reaction mixture was filtered through a PTFE syringe filter. The solvent was removed under vacuum and the residue was washed with anhydrous *n*-pentane (4 × 1 mL) and dried under vacuum.

[2,6-(Ph_2PNMes)$_2$$C_6H_3$PCl]OTf (**[4P]**OTf).

Yield: 63.0 mg (68.2 μmol, 74 %). **^{1}H NMR (600 MHz, CD_2Cl_2)**: δ = 9.58 (ddd, $^3J(^1H-^1H)$ = 12.5 Hz, $^3J(^1H-^1H)$ = 7.8 Hz, $^3J(^1H-^1H)$ = 3.9 Hz, 1H), 8.56 (tdd, $^3J(^1H-^1H)$ = 7.9 Hz, $^3J(^1H-^1H)$ = 4.2 Hz, $^3J(^1H-^1H)$ = 1.7 Hz, 1H), 8.32 (t, $^3J(^1H-^1H)$ = 9.0 Hz, 1H), 8.18 (dd, $^3J(^1H-^1H)$ = 13.6 Hz, $^3J(^1H-^1H)$ = 7.8 Hz, 2H), 8.00 – 7.87 (m, 5H), 7.86 – 7.80 (m, 1H), 7.78 (td, $^3J(^1H-^1H)$ = 8.2 Hz, $^3J(^1H-^1H)$ = 4.2 Hz, 2H), 7.68 – 7.57 (m, 7H), 7.42 (dd, $^3J(^1H-^1H)$ = 12.7 Hz, $^3J(^1H-^1H)$ = 7.4 Hz, 2H), 7.31 (dd, $^3J(^1H-^1H)$ = 12.9 Hz, $^3J(^1H-^1H)$ = 7.3 Hz, 2H), 6.82 (s, 2H), 6.78 (d, $^3J(^1H-^1H)$ = 112.4 Hz, 2H), 2.28 (s, 3H), 2.25 (s, 3H), 1.90 (s, 6H), 1.82 (s, 3H), 1.12 (s, 3H) ppm. **$^{13}C\{^1H\}$ NMR (151 MHz, CD_2Cl_2)**: δ = 155.2 (ddd, $^1J(^{31}P-^{13}C)$ = 48.9 Hz, $^2J(^{31}P-^{13}C)$ = 21.6 Hz, $J(^{31}P-^{13}C)$ = 16.9 Hz, C_1), 144.9 (d, $J(^{31}P-^{13}C)$ = 6.1 Hz), 141.2 (s), 139.3 (d, $J(^{31}P-^{13}C)$ = 16.1 Hz), 139.1 (s), 138.4 (d, $J(^{31}P-^{13}C)$ = 3.3 Hz), 138.0 (d, $J(^{31}P-^{13}C)$ = 2.1 Hz), 137.2 (d, $J(^{31}P-^{13}C)$ = 3.0 Hz), 136.2 – 136.1 (m), 136.0 (s), 135.7 – 135.5 (m), 135.4 – 135.3 (m), 134.6 (d, $J(^{31}P-^{13}C)$ = 11.5 Hz), 130.7 (s), 130.5 (s), 130.4 (d, $J(^{31}P-^{13}C)$ = 4.4 Hz), 129.7 (d, $J(^{31}P-^{13}C)$ = 4.3 Hz), 129.4 (d, $J(^{31}P-^{13}C)$ = 13.4 Hz), 128.6 (d, $J(^{31}P-^{13}C)$ = 13.3 Hz), 128.2 – 128.0 (m), 128.0 (s), 127.9 (s), 127.4 (dd, $J(^{31}P-^{13}C)$ = 16.1 Hz, $J(^{31}P-^{13}C)$ = 12.4 Hz), 121.1 (d, $J(^{19}F-^{13}C)$ = 320.8 Hz), 21.1 (s), 21.03 (s), 20.3 (s), 20.1 (s), 18.8 (d, $J(^{31}P-^{13}C)$ = 10.5 Hz) ppm. **$^{31}P\{^1H\}$ NMR (243 MHz, CD_2Cl_2):** δ = 129.4 (d, $^2J(^{31}P-^{31}P)$ = 61.8 Hz), 59.3 (d, $^2J(^{31}P-^{31}P)$ = 60.6 Hz), 33.0 (s) ppm. **Mp**: 256 °C. **HRMS (ESI, positive mode, DCM):** *m/z* 777.29115 $[M-Cl+OH^-+H_2O]^+$, Err. 1.16 mDa, 759.28115 $[M-Cl+OH^-]$, Err. 0.60 mDa.

[2,6-(Ph_2PNMes)$_2$$C_6H_3$AsCl]OTf (**[4As]**OTf).

Yield: 69.2 mg (71.7 µmol, 82 %). **^{1}H NMR (600 MHz, CD_2Cl_2)**: δ = 9.47 (dd, ^3J(^{1}H-^{1}H) = 12.8 Hz, ^3J(^{1}H-^{1}H) = 7.9 Hz, 1H), 8.47 (qd, ^3J(^{1}H-^{1}H) = 6.9 Hz, ^3J(^{1}H-^{1}H) = 6.0 Hz, ^3J(^{1}H-^{1}H) = 2.1 Hz, 1H), 8.23 (dd, ^3J(^{1}H-^{1}H) = 13.2 Hz, ^3J(^{1}H-^{1}H) = 7.7 Hz, 2H), 8.18 (t, ^3J(^{1}H-^{1}H) = 9.1 Hz, 1H), 7.88 (dd, ^3J(^{1}H-^{1}H) = 14.0 Hz, ^3J(^{1}H-^{1}H) = 7.8 Hz, 6H), 7.74 – 7.67 (m, 4H), 7.57 (qd, ^3J(^{1}H-^{1}H) = 8.0 Hz, ^3J(^{1}H-^{1}H) = 4.0 Hz, 4H), 7.39 (dd, ^3J(^{1}H-^{1}H) = 12.6 Hz, ^3J(^{1}H-^{1}H) = 7.8 Hz, 2H), 7.23 (dd, ^3J(^{1}H-^{1}H) = 13.0 Hz, ^3J(^{1}H-^{1}H) = 7.7 Hz, 2H), 6.80 (s, 2H), 6.74 (d, ^3J(^{1}H-^{1}H) = 107.1 Hz, 2H), 2.25 (s, 3H), 2.20 (s, 3H), 1.88 (s, 6H), 1.84 (s, 3H), 1.19 (s, 3H) ppm. **^{13}C{^{1}H} NMR (151 MHz, CD_2Cl_2)**: δ = 160.0 (t, J(^{31}P-^{13}C) = 19.1 Hz), 144.4 (d, J(^{31}P-^{13}C) = 7.6 Hz), 140.7 (s), 140.4 (d, J(^{31}P-^{13}C) = 16.7 Hz), 139.4 (s), 138.5 (s), 138.4 (d, J(^{31}P-^{13}C) = 2.9 Hz), 138.2 (s), 137.8 (d, J(^{31}P-^{13}C) = 11.8 Hz), 137.6 (s), 137.3 (s), 137.1 (s), 136.9 (s), 135.9 (d, J(^{31}P-^{13}C) = 13.0 Hz), 135.1 (d, J(^{31}P-^{13}C) = 10.5 Hz), 135.1 (d, J(^{31}P-^{13}C) = 10.5 Hz), 134.5 (d, J(^{31}P-^{13}C) = 11.0 Hz), 134.4 (d, J(^{31}P-^{13}C) = 10.7 Hz), 132.9 (d, J(^{31}P-^{13}C) = 14.7 Hz), 132.2 (d, J(^{31}P-^{13}C) = 12.7 Hz), 132.1 (d, J(^{31}P-^{13}C) = 13.7 Hz), 131.7 (s), 131.2 (d, J(^{31}P-^{13}C) = 13.8 Hz), 130.9 – 130.9 (m), 130.9 (s), 130.7 (d, J(^{31}P-^{13}C) = 13.2 Hz), 130.2 (d, J(^{31}P-^{13}C) = 13.3 Hz), 129.4 (d, J(^{31}P-^{13}C) = 4.5 Hz), 128.9 (s), 120.4 (q, J(^{19}F-^{13}C) = 318.5 Hz), 21.1 (s), 20.5 (s), 20.3 (s), 18.8 (s) ppm. **^{31}P{^{1}H} NMR (243 MHz, CD_2Cl_2):** δ = 60.1 (s), 33.7 (s) ppm. **Mp**: 237 °C. **HRMS (ESI, positive mode, MeCN):** *m/z* 821.19398 [M-Cl]$^+$, Err. 1.40 mDa.

[2,6-(Ph_2PNMes)$_2$$C_6H_3$SbCl]OTf (**[4Sb]**OTf).

Yield: 65.1 mg (63.85 µmol, 77 %). **^{1}H NMR (600 MHz, CD_2Cl_2)**: δ = 9.25 (dd, ^3J(^{1}H-^{1}H) = 12.6 Hz, ^3J(^{1}H-^{1}H) = 7.9 Hz, 1H), 8.32 (tdd, ^3J(^{1}H-^{1}H) = 7.8 Hz, ^3J(^{1}H-^{1}H) = 3.8 Hz, ^3J(^{1}H-^{1}H) = 2.3 Hz, 1H), 8.20 (dd, ^3J(^{1}H-^{1}H) = 12.4 Hz, ^3J(^{1}H-^{1}H) = 7.4 Hz, 2H), 8.03 (t, ^3J(^{1}H-^{1}H) = 9.3 Hz, 1H), 7.96 (t, ^3J(^{1}H-^{1}H) = 7.4 Hz, 1H), 7.93 – 7.77 (m, 8H), 7.67 (td, ^3J(^{1}H-^{1}H) = 7.7 Hz, ^3J(^{1}H-^{1}H) = 3.7 Hz, 2H), 7.64 – 7.49 (m, 7H), 7.40 (dd, ^3J(^{1}H-^{1}H) = 12.1 Hz, ^3J(^{1}H-^{1}H) = 7.6 Hz, 2H), 7.26 (dd, ^3J(^{1}H-^{1}H) = 13.0 Hz, ^3J(^{1}H-^{1}H) = 7.8 Hz, 2H), 6.83 (s, 3H), 6.67 (s, 1H),

2.26 (s, 3H), 2.19 (s, 3H), 1.90 (s, 6H), 1.84 (s, 3H), 1.33 (s, 3H) ppm. **^{13}C{^{1}H} NMR (151 MHz, CD_2Cl_2)**: δ = 169.3 (dd, J(^{31}P-^{13}C) = 23.5 Hz, J(^{31}P-^{13}C) = 19.7 Hz), 143.5 (dd, J(^{31}P-^{13}C) = 10.3 Hz, J(^{31}P-^{13}C) = 2.7 Hz), 141.2 (dd, J(^{31}P-^{13}C) = 17.7 Hz, J(^{31}P-^{13}C) = 3.2 Hz), 139.6 (dd, J(^{31}P-^{13}C) = 8.7 Hz, J(^{31}P-^{13}C) = 2.5 Hz), 139.1 (d, J(^{31}P-^{13}C) = 3.0 Hz), 138.4 (d, J(^{31}P-^{13}C) = 11.3 Hz), 138.2 (d, J(^{31}P-^{13}C) = 3.4 Hz), 138.0 (d, J(^{31}P-^{13}C) = 3.1 Hz), 137.3 (d, J(^{31}P-^{13}C) = 3.4 Hz), 137.0 (d, J(^{31}P-^{13}C) = 3.0 Hz), 136.4 (d, J(^{31}P-^{13}C) = 3.1 Hz), 135.6 (d, J(^{31}P-^{13}C) = 12.1 Hz), 135.2 (d, J(^{31}P-^{13}C) = 10.5 Hz), 134.5 (d, J(^{31}P-^{13}C) = 11.4 Hz), 134.2 (d, J(^{31}P-^{13}C) = 10.1 Hz), 133.8 – 133.4 (m), 132.6 (d, J(^{31}P-^{13}C) = 12.2 Hz), 131.7 (s), 131.1 (d, J(^{31}P-^{13}C) = 2.1 Hz), 130.9 (d, J(^{31}P-^{13}C) = 5.8 Hz), 130.9 (d, J(^{31}P-^{13}C) = 6.1 Hz), 130.5 (s), 130.5 (d, J(^{31}P-^{13}C) = 10.0 Hz), 130.4 (s), 120.0 (q, J(^{19}F-^{13}C) = 318.6 Hz), 21.1 (s), 21.0 (s), 20.9 (s), 20.5 (s), 19.3 (s) ppm. **^{31}P{^{1}H} NMR (243 MHz, CD_2Cl_2):** δ = 60.0 (s), 36.1 (s) ppm. **Mp:** 273 °C. **HRMS (ESI, positive mode, DCM):** *m/z* 867.17792 [M]$^{+}$, Err. 1.07 mDa.

[2,6-(Ph_2PNMes)$_2C_6H_3$BiCl]OTf (**[4Bi]**OTf).

Yield: 45.9 mg (42.35 µmol, 56 %). **^{1}H NMR (600 MHz, CD_2Cl_2)**: δ = 12.21 (s, 3H), 9.22 (t, ^{3}J(^{1}H-^{1}H) = 10.1 Hz, 1H), 8.35 (t, ^{3}J(^{1}H-^{1}H) = 7.9 Hz, 1H), 8.25 (t, ^{3}J(^{1}H-^{1}H) = 9.5 Hz, 1H), 7.93 (t, ^{3}J(^{1}H-^{1}H) = 7.8 Hz, 2H), 7.84 – 7.68 (m, 8H), 7.60 (q, ^{3}J(^{1}H-^{1}H) = 7.3 Hz, 6H), 7.50 (dd, ^{3}J(^{1}H-^{1}H) = 13.3 Hz, ^{3}J(^{1}H-^{1}H) = 7.8 Hz, 1H), 7.26 (d, ^{3}J(^{1}H-^{1}H) = 8.2 Hz, 1H), 6.82 (s, 2H), 6.71 (d, ^{3}J(^{1}H-^{1}H) = 28.0 Hz, 2H), 2.25 (s, 3H), 2.21 (s, 3H), 2.03 (d, ^{3}J(^{1}H-^{1}H) = 147.5 Hz, 3H), 1.88 (s, 6H), 1.72 (s, 3H) ppm. **^{13}C{^{1}H} NMR (151 MHz, CD_2Cl_2)**: δ = 146.1 (d, J(^{31}P-^{13}C) = 17.1 Hz), 145.5 (dd, J(^{31}P-^{13}C) = 18.0 Hz, J(^{31}P-^{13}C) = 3.3 Hz), 145.3 (d, J(^{31}P-^{13}C) = 17.5 Hz), 145.4 – 145.2 (m), 139.5 (d, J(^{31}P-^{13}C) = 2.6 Hz), 139.0 (s), 138.8 (d, J(^{31}P-^{13}C) = 2.6 Hz), 138.6 (s), 138.0 (d, J(^{31}P-^{13}C) = 3.3 Hz), 137.9 (d, J(^{31}P-^{13}C) = 2.8 Hz), 136.3 (s), 135.6 (s), 134.5 (d, J(^{31}P-^{13}C) = 11.5 Hz), 133.7 (d, J(^{31}P-^{13}C) = 12.3 Hz), 132.2 (d, J(^{31}P-^{13}C) = 4.6 Hz), 131.5 (t, J(^{31}P-^{13}C) = 12.2 Hz), 131.0 (d, J(^{31}P-^{13}C) = 2.1 Hz), 130.5 (s), 130.4 (s), 129.5 (d, J(^{31}P-^{13}C) = 5.3 Hz), 119.8 (q, J(^{19}F-^{13}C) = 318.2 Hz), 21.1 (s), 20.9 (s), 21.0 –

20.3 (m), 20.4 (s), 20.1 (s) ppm. **$^{31}P\{^1H\}$ NMR (243 MHz, CD_2Cl_2):** δ = 78.2 (s), 37.5 (s) ppm. **Mp**: 288 °C. **HRMS (ESI, positive mode, DCM):** *m/z* 955.25333 $[M]^+$, Err. 1.17 mDa.

Synthesis of 5Pn (Pn = As, Sb, Bi): $Li(Et_3BH)$ (1M in THF, 5 eq.) was added to a solution of **3Pn** (50.0 mg, 1 eq.) in THF (0.5 mL) at 0 °C. The reaction instantly turned dark red and was stirred for further 5 min. Quantitative conversion was confirmed by ^{31}P NMR. The desired products **5Pn** are labile towards extended time of vacuum therefore drying was immediately stopped after the solvent was evaporated. **5As, 5Sb** and **5Bi** start to decompose under inert conditions within the first hours at room temperature. The lifetime is improved under inert conditions at –40 °C to towards around 3 days.

2,6-$(Ph_2PNMes)_2C_6H_3As$ (5As):

Dark red crystals suitable for X-ray single crystal diffraction measurements of **5As** were obtained by the layering of a THF solution with *n*-hexane at –40 °C.

^1H NMR (600 MHz, THF-d_8): δ = 7.66 (dd, *J* = 11.5 Hz, *J* = 7.5 Hz, 8H), 7.50 (t, *J* = 7.3 Hz, 5H), 7.42–7.38 (m, 10H), 6.47 (s, 4H), 2.03 (s, 6H), Mes_{ortho}-CH_3 signal overlapped with THF signal at 1.77 ppm, ppm. **$^{13}C\{^1H\}$ NMR (151 MHz, THF-d_8)**: δ = 184.1 (t, *J* = 15.2 Hz, C_1), 140.0 (s), 136.7 – 135.6 (m), 135.0 (d, *J* = 17.2 Hz), 131.8 (s), 131.8 (s), 131.8 (s), 131.6 (s), 131.2 (s), 130.7 (s), 128.5 (s), 128.5 (s), 128.4 (s), 119.1 (d, *J* = 13.5 Hz), 118.2 (d, *J* = 13.4 Hz), 114.8 (t, *J* = 15.0 Hz), 20.2 (s), 19.9 (s) ppm. Aliphatic area was not assigned due to overlapping signals with THF-H_8. **$^{31}P\{^1H\}$ NMR (243 MHz, THF-d_8):** δ = 11.8 (s) ppm.

2,6-$(Ph_2PNMes)_2C_6H_3Sb$ (5Sb):

Dark red Crystals suitable for X-ray single crystal diffraction measurements of **5Sb** were obtained from THF/*n*-hexane at –40 °C after the reaction of **3Sb** and $LiAlH_4$

^{1}H NMR (600 MHz, THF-d$_8$): 7.67 (dd, J(^{1}H-^{1}H) = 11.8 Hz, J(^{1}H-^{1}H) = 7.5 Hz, 8H), 7.49 – 7.38 (m, 5H), 7.35 – 7.33 (m, 10H), 6.41 (d, J(^{1}H-^{1}H) = 4.2 Hz, 4H), 1.98 (s, 6H) ppm. The remaining Mes$_{ortho}$-CH$_3$ signal overlaps with excess of THF-H$_8$. **^{13}C{^{1}H} NMR (151 MHz, CD$_2$Cl$_2$)**: Not measured due to rapid decomposition. **^{31}P{^{1}H} NMR (243 MHz, THF-d$_8$):** δ = 10.8 (s) ppm.

2,6-(Ph$_2$PNMes)$_2$C$_6$H$_3$Bi (5Bi):

^{1}H NMR (600 MHz, THF-d$_8$): δ = 7.68 (dd, J(^{1}H-^{1}H) = 11.3 Hz, J(^{1}H-^{1}H) = 7.5 Hz, 8H), 7.44 (m, 5H), 7.34 (m, 10H), 6.42 (s, 4H), 2.04 (s, 6H) ppm. The remaining Mes-*ortho*-CH$_3$ signal overlaps with excess of THF-H$_8$. **^{13}C{^{1}H} NMR (151 MHz, THF-d$_8$)**: δ = 184.0 (t, J(^{31}P-^{13}C) = 20.2 Hz, C$_1$), 144.1 (dd, J(^{31}P-^{13}C) = 131.7 Hz, J(^{31}P-^{13}C) = 15.8 Hz), 143.4 (s), 136.4 (s), 136.2 (d, J(^{31}P-^{13}C) = 5.9 Hz), 135.8 (s), 135.2 (d, J(^{31}P-^{13}C) = 20.2 Hz), 134.4 (d, J(^{31}P-^{13}C) = 9.4 Hz), 133.0 (d, J(^{31}P-^{13}C) = 9.9 Hz), 132.4 (d, J = 2.9 Hz), 132.0, 130.7 (d, J(^{31}P-^{13}C) = 3.5 Hz), 129.4 (s), 129.12 (s), 129.1 (s), 127.5 (t, J(^{31}P-^{13}C) = 13.9 Hz) ppm. Aliphatic area was not assigned due to overlapping signals with THF-H$_8$. **^{31}P{^{1}H} NMR (243 MHz, THF-d$_8$):** δ = 8.8 (s) ppm.

Synthesis of 2,6-(Ph$_2$PNMes)$_2$C$_6$H$_3$Sb·BH$_3$ (5Sb·BH$_3$):

Procedure A: NaBH$_4$ (2.09 mg, 55.3 µmol, 1 eq.) was added to a solution of **3Sb** (50.0 mg, 55.3 µmol, 1 eq.) in THF (0.5 mL). The reaction mixture was stirred at 50 °C for 3 d. The reaction mixture was filtered through a PTFE syringe filter. **5Sb**·BH$_3$ is labile towards extended time of vacuum therefore drying was immediately stopped after the solvent was evaporated. Suitable crystals for single crystal x-ray diffraction measurements were obtained by recrystallization from THF/*n*-hexane.

Procedure B: Li(Et$_3$BH) (0.0280 mL, 1M in THF, 0.280 mmol, 5 eq.) was added to a solution of **3Sb** (50.0 mg, 55.3 µmol, 1 eq.) in THF (0.5 mL) at 0 °C. The reaction instantly turned dark

red and was stirred for further 5 min. Quantitative conversion was confirmed by ^{31}P NMR. The reaction mixture was cooled down to –40 °C and BH_3·THF complex (1M in THF, 1 eq.) was added. The reaction mixture was filtered through a PTFE syringe filter. **5Sb**·BH_3 is labile towards extended time of vacuum therefore drying was immediately stopped after the solvent was evaporated.

^{1}H NMR (600 MHz, THF-*d*$_8$): δ = 8.16 – 8.08 (m, 4H), 7.58 (td, J(^{1}H-^{1}H) = 7.4 Hz, J(^{1}H-^{1}H) = 1.5 Hz, 2H, H_3), 7.52 – 7.45 (m, 4H), 7.45 – 7.30 (m, 13H), 6.62 (s, 2H, Mes-CH), 6.38 (s, 2H, Mes-CH), 2.15 (s, 6H, Mes-CH_3), 2.06 (d, J(^{1}H-^{1}H), 6H, Mes-CH_3), 1.47 (s, 6H, Mes-CH_3), 0.11 (s, 3H) ppm. **^{13}C{^{1}H} NMR (151 MHz, THF-*d*$_8$)**: δ = 177.5 (t, J(^{31}P-^{13}C) = 16.4 Hz, C_1), 140.3 (d, J(^{31}P-^{13}C) = 3.6 Hz), 138.0 (d, J(^{31}P-^{13}C) = 4.4 Hz), 137.3 – 137.0 (m), 136.7 – 136.5 (m), 135.4 (d, J(^{31}P-^{13}C) = 8.9 Hz), 135.3 (d, J(^{31}P-^{13}C) = 11.5 Hz), 134.8, 134.2 (d, J(^{31}P-^{13}C) = 15.4 Hz), 133.4 (d, J(^{31}P-^{13}C) = 8.6 Hz), 133.0 (s), 132.7 (s), 132.4 (d, J(^{31}P-^{13}C) = 2.6 Hz), 129.8 (s), 129.6 (s), 129.4 (t, J(^{31}P-^{13}C) = 10.7 Hz), 127.3 (s), 126.7 (d, J(^{31}P-^{13}C) = 8.3 Hz), 22.3 (s), 21.0 (s), 20.8 (s) ppm. **^{31}P{^{1}H} NMR (243 MHz, THF-d$_8$):** δ = 16.0 (s) ppm. **^{11}B NMR (192 MHz, THF):** δ = –0.1 (q, ^{1}J(^{1}H-^{11}B) = 106.8 Hz) ppm.

Synthesis of 6PnCh (Pn = As, Sb, Bi; Ch = S, Se, Te):

Procedure A (preferred method):

3Pn (Pn = As, Sb, Bi) (50.0 mg, 1 eq.) and chalcogen powder (Ch = S, Se, Te, 1eq.) were dissolved in 3 mL anhydrous and degassed THF. Li[Et_3BH] (5 eq.) was added at –40 °C. The solution was allowed to warm up to room temperature within 10 minutes. The precipitate was filtered off *via* PTFE syringe filtration. The filtrate was recrystallized from THF/*n*-hexane. The **6PnS** and **6PnSe** compounds crystallized as yellow or slightly orange crystals, while the **6PnTe** compounds were obtained as red crystals. No conversion was observable in the reaction between **5As** and tellurium powder. **6PnCh** decompose in common organic solvents such as

DCM or MeCN, as well as if exposed to moisture or air. The compounds are labile to extended time of vacuum therefore the drying process of the compounds were immediately stopped after the solvent was evaporated. Crystals suitable for single crystal X-ray diffraction measurements (**6AsSe**, **6SbSe** and **6BiTe**) were obtained by recrystallization from THF/*n*-hexane.

Procedure B:

3Pn (Pn = As, Sb, Bi) (50.0 mg, 1 eq.) was dissolved in 3 mL anhydrous and degassed THF and Li[Et_3BH] (5 eq.) was added at room temperature. After 5 minutes the blood red solution was cooled down to –40 °C. Chalcogen powder (Ch = S, Se, Te, 1 eq.) was added and the solution was allowed to warm up to room temperature within 10 minutes.

Work up was performed analogue to Procedure A.

2,6-(Ph_2PNMes)$_2$$C_6H_3$AsS (6AsS):

^{1}H NMR (600 MHz, THF-d_8): δ = 8.43 (dd, *J* = 12.8 Hz, *J* = 7.6 Hz, 4H), 7.62 – 7.53 (m, 4H), 7.52 – 7.47 (m, 3H), 7.44 (t, *J* = 6.8 Hz, 8H), 7.37 (t, *J* = 9.3 Hz, 4H), 6.51 (s, 4H), 2.05 (s, 6H), 1.83 (s_{br}, 12H) ppm. **^{13}C{^{1}H} NMR (151 MHz, THF-d_8)**: δ =178.3 (t, *J* = 5.1 Hz, C_1), 139.5 (s), 137.1 (s), 137.0 (s), 136.8 (d, *J* = 13.0 Hz), 133.9 (s), 133.3 (s), 133.0 (s), 132.8 (d, *J* = 8.6 Hz), 132.69, 132.1 (s), 129.7 (d, *J* = 11.4 Hz), 129.4 (s), 129.2 (d, *J* = 12.6 Hz), 128.3 (d, *J* = 16.0 Hz), 127.7 (s), 21.3 (s_{br}), 21.0 (s) ppm. **^{31}P{^{1}H} NMR (243 MHz, THF-d_8):** δ = 13.2 (s) ppm.

2,6-(Ph_2PNMes)$_2$$C_6H_3$AsSe (6AsSe):

^{1}H NMR (600 MHz, THF-d_8): δ = 8.50 (dd, *J* = 12.7 Hz, *J* = 7.3 Hz, 4H), 7.59 (t, *J* = 7.4 Hz, 2H), 7.57 – 7.49 (m, 5H), 7.43 (td, *J* = 10.2 Hz, *J* = 2.6 Hz, 8H), 7.37 – 7.27 (m, 4H), 6.51 (s, 4H), 2.05 (s, 6H); 1.86 (s_{br}, 12H) ppm. **^{13}C{^{1}H} NMR (151 MHz, THF-d_8)**: δ = 178.5 (t, *J* = 13.8 Hz), 139.3 (d, *J* = 3.2 Hz), 137.2 (s), 137.1 (s), 137.0 (s), 134.1 (d, *J* = 14.2 Hz), 133.4 (d, *J* = 90.0 Hz), 133.0 (s), 132.8 (s), 132.7 (d, *J* = 8.8 Hz), 132.3 (d, *J* = 3.2 Hz), 129.7 (d, *J* = 11.3

Hz), 129.5 (s), 129.2 (d, *J* = 12.6 Hz), 128.4 (d, *J* = 22.7 Hz), 127.9 (s), 21.3 (s_{br}), 21.0 (s) ppm. **^{31}P{^{1}H} NMR (243 MHz, THF-d$_8$):** δ = 12.9 (s) ppm.

2,6-($Ph_2PNMes)_2C_6H_3SbS$ (6SbS):

^{1}H NMR (600 MHz, THF-*d*$_8$): δ = 8.53 (dd, *J*(^{1}H-^{1}H) = 12.9 Hz, *J*(^{1}H-^{1}H) = 7.6 Hz, 4H), 7.67 – 7.59 (m, 4H), 7.59 – 7.55 (m, 1H, H_4), 7.52 (t, *J*(^{1}H-^{1}H) = 7.3 Hz, 2H, H_3), 7.47 (td, *J*(^{1}H-^{1}H) = 7.8 Hz, *J*(^{1}H-^{1}H) = 2.8 Hz, 8H), 7.44 – 7.34 (m, 4H), 6.69 (s, 2H, Mes-CH), 6.45 (s, 2H, Mes-CH), 2.27 (s, 6H, Mes-CH_3), 2.10 (s, 6H, Mes-CH_3), 1.43 (s, 6H, Mes-CH_3) ppm. **^{13}C{^{1}H} NMR (151 MHz, THF-*d*$_8$)**: δ = 139.5 (s), 138.9 – 138.6 (m), 137.6 (d, *J*(^{31}P-^{13}C) = 20.2 Hz), 136.7 (d, *J*(^{31}P-^{13}C) = 12.7 Hz), 135.9 (d, *J*(^{31}P-^{13}C) = 15.7 Hz), 135.8 (d, *J*(^{31}P-^{13}C) = 4.6 Hz), 133.2 (s), 133.1 (s), 132.7 (s), 129.9 (s), 129.7 (d, *J*(^{31}P-^{13}C) = 11.2 Hz), 129.3 (d, *J*(^{31}P-^{13}C) = 12.2 Hz), 128.7 – 128.3 (m), 127.4 (d, *J*(^{31}P-^{13}C) = 80.7 Hz), 22.7 (s), 21.0 (s), 20.0 (s) ppm. **^{31}P{^{1}H} NMR (243 MHz, THF-*d*$_8$):** δ = 15.6 (s) ppm.

2,6-($Ph_2PNMes)_2C_6H_3SbSe$ (6SbSe):

^{1}H NMR (600 MHz, THF-*d*$_8$): δ = 8.52 (dd, *J*(^{1}H-^{1}H) = 12.9, *J*(^{1}H-^{1}H) = 7.6 Hz, 4H), 7.60 (t, *J*(^{1}H-^{1}H) = 7.7 Hz, 2H), 7.53 (dt, *J*(^{1}H-^{1}H) = 29.3, *J*(^{1}H-^{1}H) = 7.1 Hz, 6H), 7.46 – 7.39 (m, 8H), 7.33 (t, *J*(^{1}H-^{1}H) = 9.3 Hz, 4H), 6.64 (s, 2H), 6.41 (s, 2H), 2.27 (s, 6H), 2.06 (s, 6H), 1.38 (s, 6H). **^{13}C{^{1}H} NMR (151 MHz, THF-*d*$_8$)**: δ = 176.5 (t, *J*(^{31}P-^{13}C) = 16.6 Hz, C_1), 138.3 (d, *J*(^{31}P-^{13}C) = 3.9 Hz), 137.7 (d, *J*(^{31}P-^{13}C) = 4.9 Hz), 136.5 (dd, *J*(^{31}P-^{13}C) = 18.4 Hz, *J*(^{31}P-^{13}C) = 3.5 Hz), 135.8 (d, *J*(^{31}P-^{13}C) = 12.8 Hz), 134.9 (d, *J*(^{31}P-^{13}C) = 15.6 Hz), 134.6 (d, *J*(^{31}P-^{13}C) = 5.3 Hz), 132.4 (d, *J*(^{31}P-^{13}C) = 90.9 Hz), 131.9 (d, *J*(^{31}P-^{13}C) = 2.6 Hz), 131.9 (d, *J*(^{31}P-^{13}C) = 8.7 Hz), 131.6 (d, *J*(^{31}P-^{13}C) = 2.8 Hz), 131.4 (d, *J*(^{31}P-^{13}C) = 3.3 Hz), 128.8 (s), 128.5 (d, *J*(^{31}P-^{13}C) = 11.2 Hz), 128.2 (s), 128.1 (s), 127.2 (t, *J*(^{31}P-^{13}C) = 13.6 Hz), 126.5 (d, *J*(^{31}P-^{13}C) = 82.0 Hz), 21.6 (s), 19.8 (s), 18.7 (s). **^{31}P{^{1}H} NMR (243 MHz, THF-*d*$_8$):** δ = 15.0 (s) ppm.

2,6-($Ph_2PNMes)_2C_6H_3SbTe$ (6SbTe):

^{1}H NMR (600 MHz, THF-*d*$_8$): δ = 8.54 (dd, *J*(^{1}H-^{1}H) = 12.8, *J*(^{1}H-^{1}H) = 7.1 Hz, 4H), 7.60 (t, *J*(^{1}H-^{1}H) = 7.4 Hz, 2H), 7.56 – 7.51 (m, 6H), 7.43 (dtd, *J*(^{1}H-^{1}H) = 15.9, *J*(^{1}H-^{1}H) = 7.8, *J*(^{1}H-^{1}H) = 2.8 Hz, 8H), 7.28 (dd, *J*(^{1}H-^{1}H) = 10.3, *J*(^{1}H-^{1}H) = 7.7 Hz, 4H), 6.64 (s, 2H), 6.41 (s, 2H), 2.31 (s, 6H), 2.06 (s, 6H), 1.36 (s, 6H) ppm. **^{13}C{^{1}H} NMR (151 MHz, THF-*d*$_8$)**: δ = 173.7 (t, *J*(^{31}P-^{13}C) = 15.6 Hz, C_1), 139.1 (dd, *J*(^{31}P-^{13}C) = 57.6 Hz, *J*(^{31}P-^{13}C) = 4.5 Hz), 137.7 (d, *J*(^{31}P-^{13}C) = 18.1 Hz), 137.3 (d, *J*(^{31}P-^{13}C) = 13.1 Hz), 133.0 (d, *J*(^{31}P-^{13}C) = 22.3 Hz), 132.8 (d, *J*(^{31}P-^{13}C) = 8.4 Hz), 132.8 – 132.6 (m), 130.3 (s), 130.2 (s), 129.6 (d, *J*(^{31}P-^{13}C) = 11.2 Hz), 129.5 (s), 129.3 (d, *J*(^{31}P-^{13}C) = 12.5 Hz), 128.6 (t, *J*(^{31}P-^{13}C) = 13.6 Hz), 127.9 (d, *J*(^{31}P-^{13}C) = 82.6 Hz), 23.2 (s) 21.0 (s), 19.7 (s). **^{31}P{^{1}H} NMR (243 MHz, THF-*d*$_8$):** δ = 14.6 (s) ppm. **^{125}Te NMR (189 MHz, THF-d$_8$):** δ = –274.8 (s) ppm.

2,6-($Ph_2PNMes)_2C_6H_3BiS$ (6BiS):

^{1}H NMR (600 MHz, THF-*d*$_8$): δ = 8.50 (t, *J*(^{1}H-^{1}H) = 9.9 Hz, 4H), 7.73 – 7.57 (m, 6H), 7.52 – 7.31 (m, 16H), 6.56 (d, *J* = 127.7 Hz, 4H, Mes-CH), 2.15 (s, 6H, Mes-CH_3), 2.07 (s, 6H), 1.39 (s, 6H) ppm. **^{13}C{^{1}H} NMR (151 MHz, THF-*d*$_8$):** δ = 209.6 (t, ^{1}*J*(^{31}P-^{13}C) = 17.2 Hz, C_1), 142.9 (dd, ^{1}*J*(^{31}P-^{13}C) = 129.8 Hz, ^{3}*J*(^{31}P-^{13}C) = 15.9 Hz, C_2), 140.7 (d, *J*(^{31}P-^{13}C) = 3.4 Hz), 140.6 (d, *J*(^{31}P-^{13}C) = 3.9 Hz), 138.2 – 137.8 (m), 136.2 (d, *J*(^{31}P-^{13}C) = 12.2 Hz), 135.4 – 134.7 (m), 134.4 (s), 133.2 (s), 133.0 (d, *J*(^{31}P-^{13}C) = 8.6 Hz), 132.7 (s), 132.3 (d, *J*(^{31}P-^{13}C) = 3.4 Hz), 130.0 (s), 129.8 (d, *J*(^{31}P-^{13}C) = 11.2 Hz), 129.5 (s), 129.4 (s), 128.1 (s), 127.7 (d, *J*(^{31}P-^{13}C) = 13.7 Hz), 22.5 (s), 20.9 (s), 20.1 (s). **^{31}P{^{1}H} NMR (243 MHz, THF-*d*$_8$):** δ = 22.8 (s) ppm.

2,6-($Ph_2PNMes)_2C_6H_3BiSe$ (6BiSe):

^{1}H NMR (600 MHz, THF-*d*$_8$): δ = 8.53 (dd, *J*(^{1}H-^{1}H) = 12.7 Hz, *J*(^{1}H-^{1}H) = 7.7 Hz, 1H), 7.69 – 7.62 (m, 1H), 7.62 – 7.55 (m, 4H), 7.52 – 7.47 (m, 2H), 7.43 (q, *J*(^{1}H-^{1}H) = 8.2 Hz, 8H), 7.33

(t, $J(^{1}H-^{1}H)$ = 9.3 Hz, 4H), 6.65 (s, 2H, Mes-CH), 6.42 (s, 2H, Mes-CH), 2.20 (s, 6H, Mes-CH_3), 2.06 (s, 6H, Mes-CH_3), 1.35 (s, 6H, Mes-CH_3) ppm. **$^{13}C\{^{1}H\}$ NMR (151 MHz, THF-*d_8*)**: δ = 205.6 (t, $^{1}J(^{31}P-^{13}C)$ = 18.1 Hz, C_1), 143.2 (dd, $J(^{31}P-^{13}C)$ = 130.8 Hz, $J(^{31}P-^{13}C)$ = 16.0 Hz, C_2), 140.7 (s), 140.4 (d, $J(^{31}P-^{13}C)$ = 18.7 Hz), 138.2 (d, $J(^{31}P-^{13}C)$ = 4.6 Hz), 136.6 (d, $J(^{31}P-^{13}C)$ = 12.3 Hz), 135.4 (s), 135.2 – 134.3 (m), 133.0 (s), 132.9 (s), 132.5 (s), 132.0 (s), 130.1 (s), 129.6 (d, $J(^{31}P-^{13}C)$ = 11.0 Hz), 129.5 (s), 129.4 (s), 128.3 (d, $J(^{31}P-^{13}C)$ = 80.6 Hz), 127.2 (t, $J(^{31}P-^{13}C)$ = 13.8 Hz), 22.6 (s), 21.0 (s), 20.0 (s) ppm. **$^{31}P\{^{1}H\}$ NMR (243 MHz, THF-*d_8*):** δ = 20.5 (s) ppm.

2,6-($Ph_2PNMes)_2C_6H_3BiTe$ (6BiTe):

^{1}H NMR (600 MHz, THF-*d_8*): δ = 8.52 (d, $J(^{1}H-^{1}H)$ = 12.6 Hz, $J(^{1}H-^{1}H)$ = 7.3 Hz, 4H), 7.67 (tt, $J(^{1}H-^{1}H)$ = 7.5 Hz, $J(^{1}H-^{1}H)$ = 3.6 Hz, 1H), 7.59 (t, $J(^{1}H-^{1}H)$ = 7.4 Hz, 2H), 7.54 (dd, $J(^{1}H-^{1}H)$ = 9.3 Hz, $J(^{1}H-^{1}H)$ = 7.4 Hz, 4H), 7.45 (td, $J(^{1}H-^{1}H)$ = 7.8 Hz, $J(^{1}H-^{1}H)$ = 3.1 Hz, 4H), 7.41 (td, $J(^{1}H-^{1}H)$ = 7.8 Hz, $J(^{1}H-^{1}H)$ = 2.7 Hz, 4H), 7.31 – 7.22 (m, 4H), 6.64 (s, 2H), 6.42 (s, 2H), 2.22 (s, 6H), 2.06 (s, 6H), 1.33 – 1.30 (m, 6H) ppm. **$^{13}C\{^{1}H\}$ NMR (151 MHz, THF-*d_8*)**: δ = 194.0 (t, J = 17.0 Hz), 142.7 (dd, $J(^{31}P-^{13}C)$ = 130.4 Hz, $J(^{31}P-^{13}C)$ = 16.0 Hz), 139.5 (d, $J(^{31}P-^{13}C)$ = 4.1 Hz), 139.3 (d, $J(^{31}P-^{13}C)$ = 19.8 Hz), 137.2 – 137.0 (m), 135.9 (d, $J(^{31}P-^{13}C)$ = 12.1 Hz), 134.5 (d, $J(^{31}P-^{13}C)$ = 91.8 Hz), 133.7 (d, $J(^{31}P-^{13}C)$ = 4.1 Hz), 132.1 – 131.9 (m), 131.8, 131.6 (d, $J(^{31}P-^{13}C)$ = 8.3 Hz), 131.5 (s), 130.9 (s), 129.1 (s), 128.5 (d, $J(^{31}P-^{13}C)$ = 11.1 Hz), 128.4 (d, $J(^{31}P-^{13}C)$ = 12.0 Hz), 126.9 (d, $J(^{31}P-^{13}C)$ = 80.8 Hz), 126.3 (t, $J(^{31}P-^{13}C)$ = 13.7 Hz), 21.8 (s), 19.8 (s), 18.6 (s) ppm. **$^{31}P\{^{1}H\}$ NMR (243 MHz, THF-d_8):** δ = 18.2 (s) ppm. **^{125}Te NMR (189 MHz, THF-d_8):** δ = –191.1 (s) ppm.

NMR Spectra

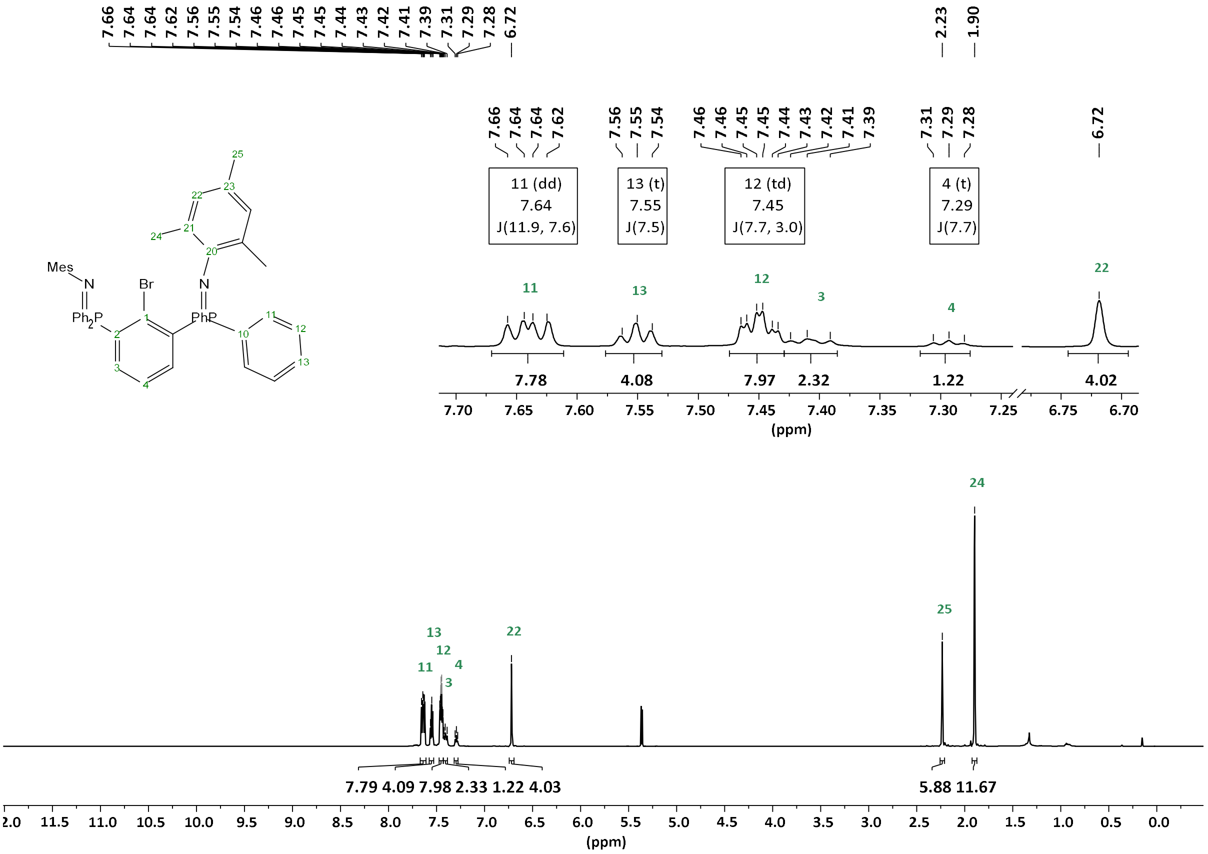

Figure S1: ^{1}H NMR spectrum (CD_2Cl_2, 600 MHz) of **1.**

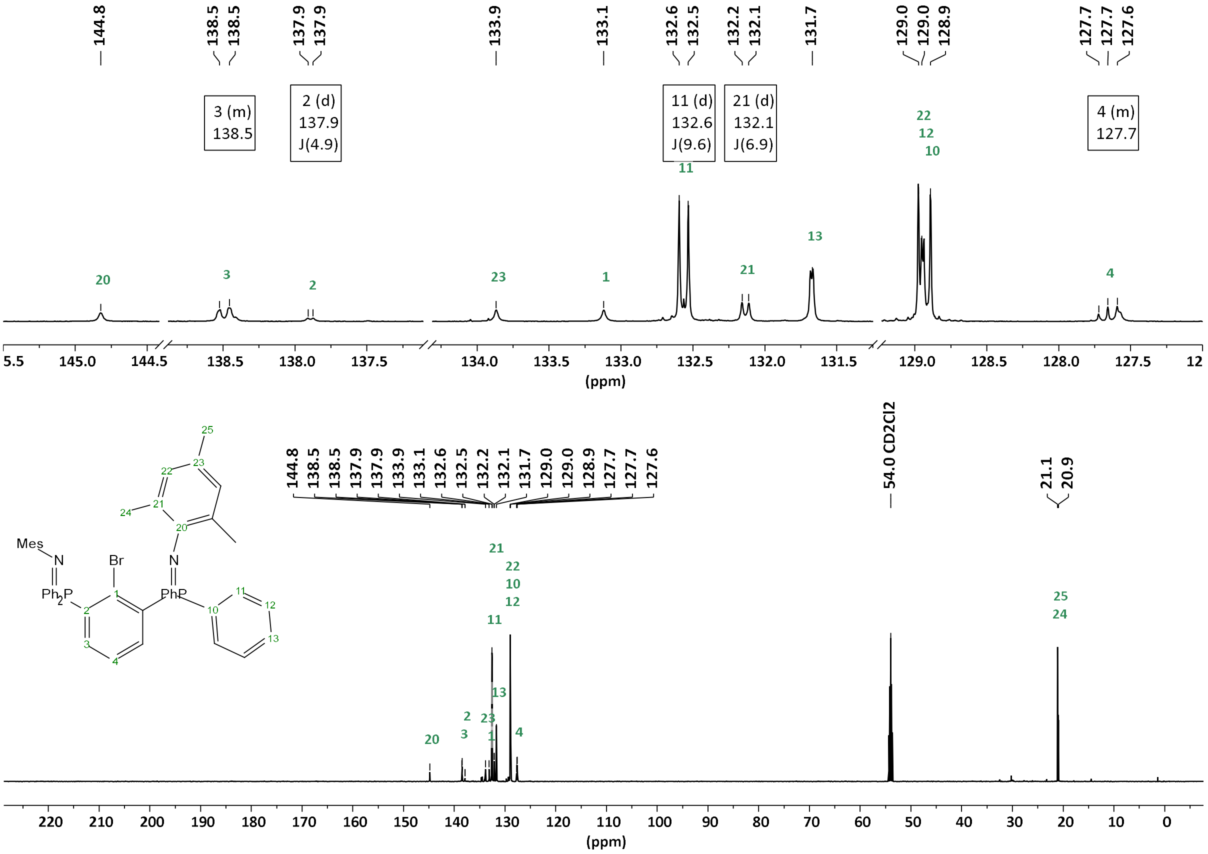

Figure S2: $^{13}C\{^{1}H\}$ NMR spectrum (CD_2Cl_2, 151 MHz) of **1**.

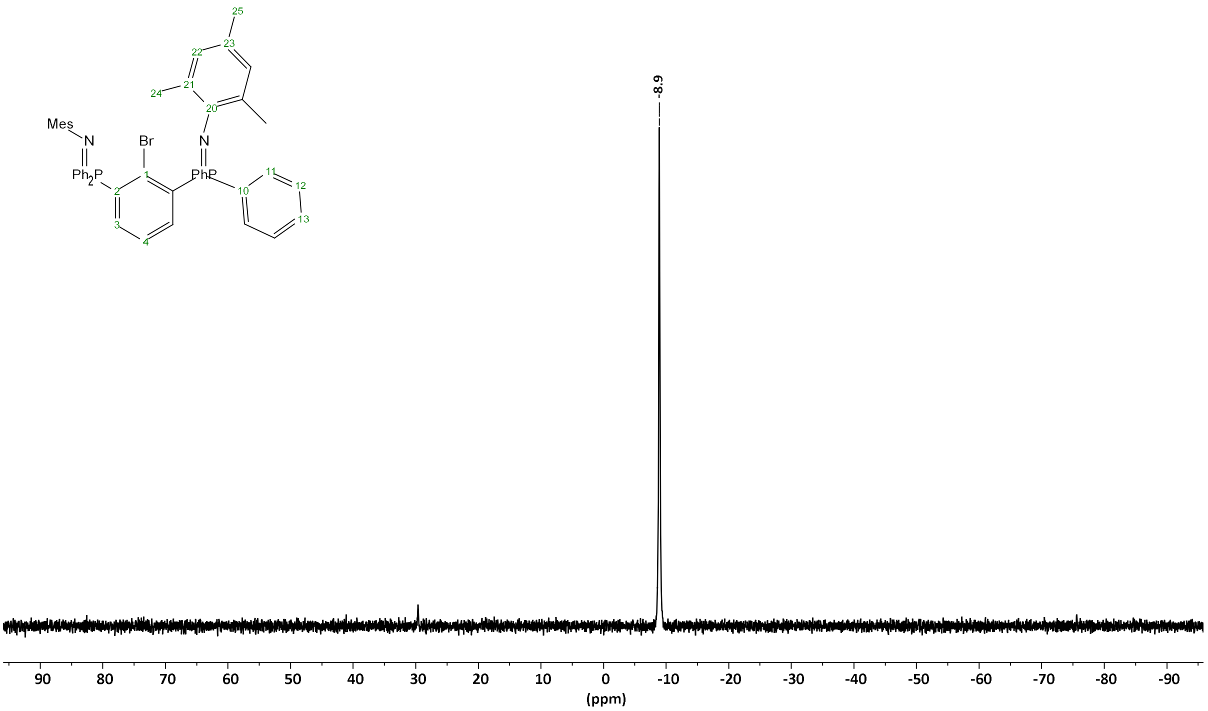

Figure S3: $^{31}P\{1H\}$ NMR spectrum (CD_2Cl_2, 151 MHz) of 1.

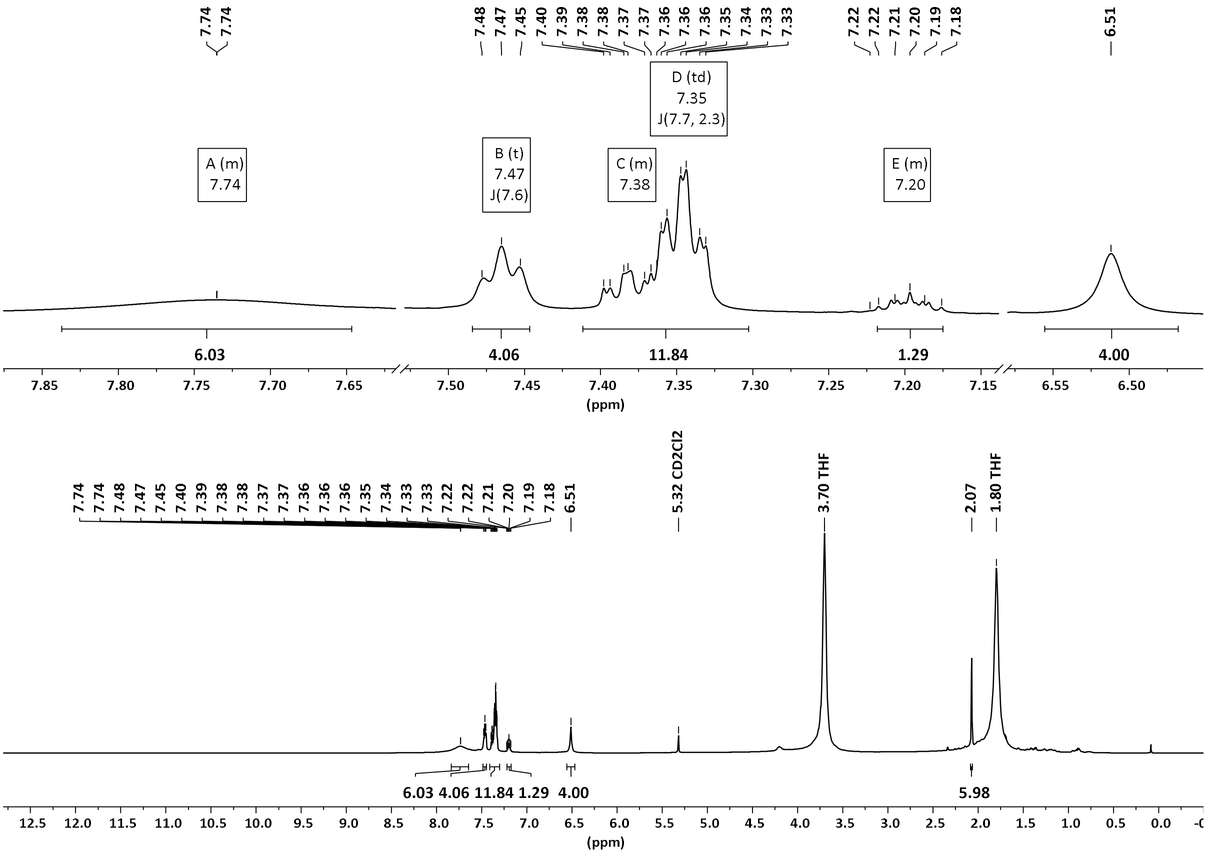

Figure S4: ^{1}H NMR spectrum (CD_2Cl_2, 600 MHz) of **2.**

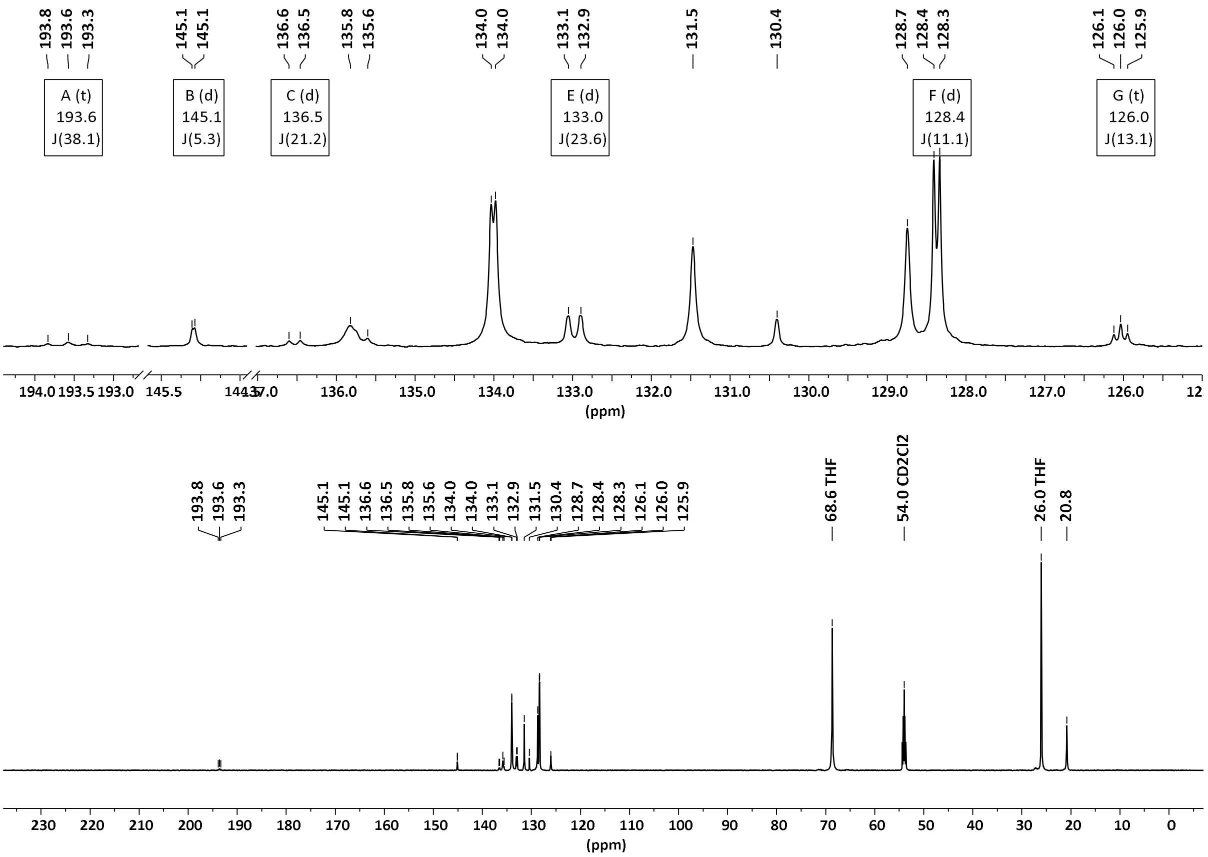

Figure S5: $^{13}C\{^{1}H\}$ NMR spectrum (CD_2Cl_2, 151 MHz) of **2**.

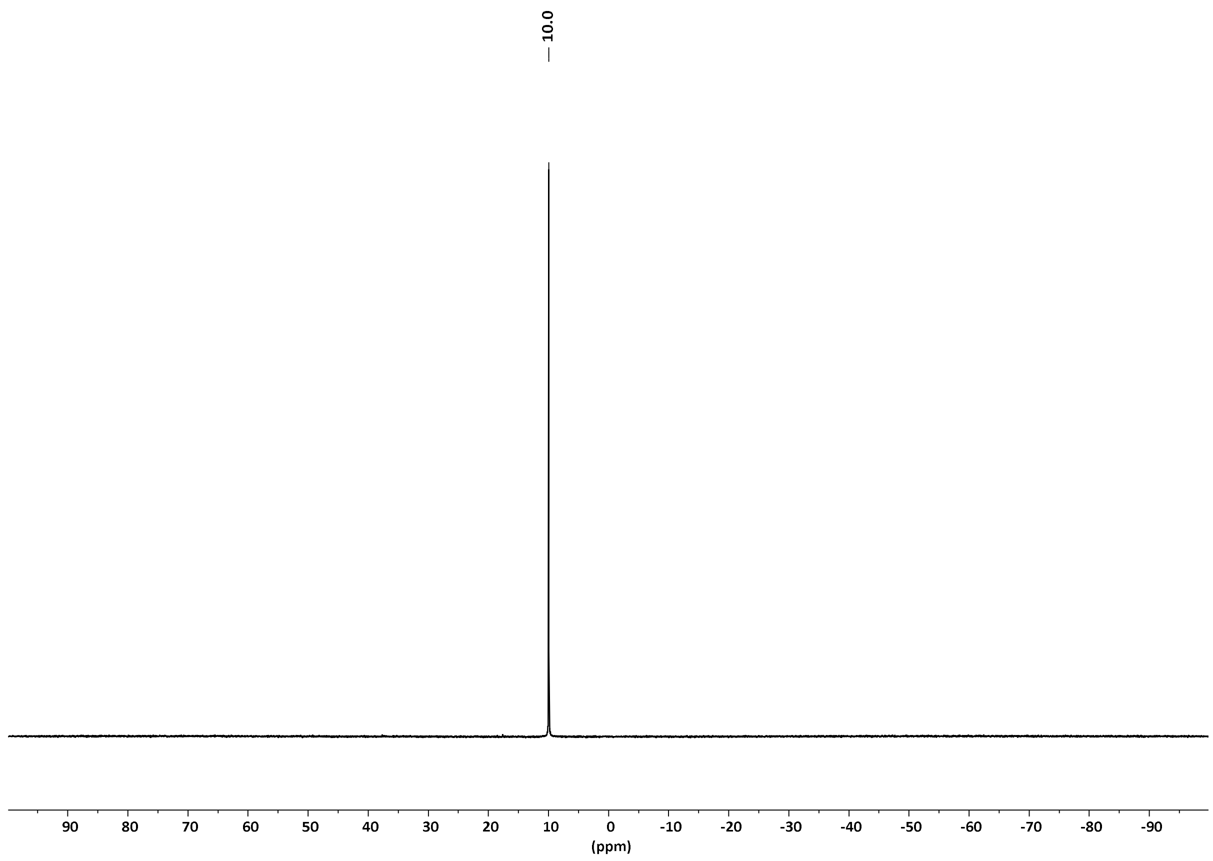

Figure S6: $^{31}P\{^1H\}$ NMR spectrum (CD_2Cl_2, 151 MHz) of **2**.

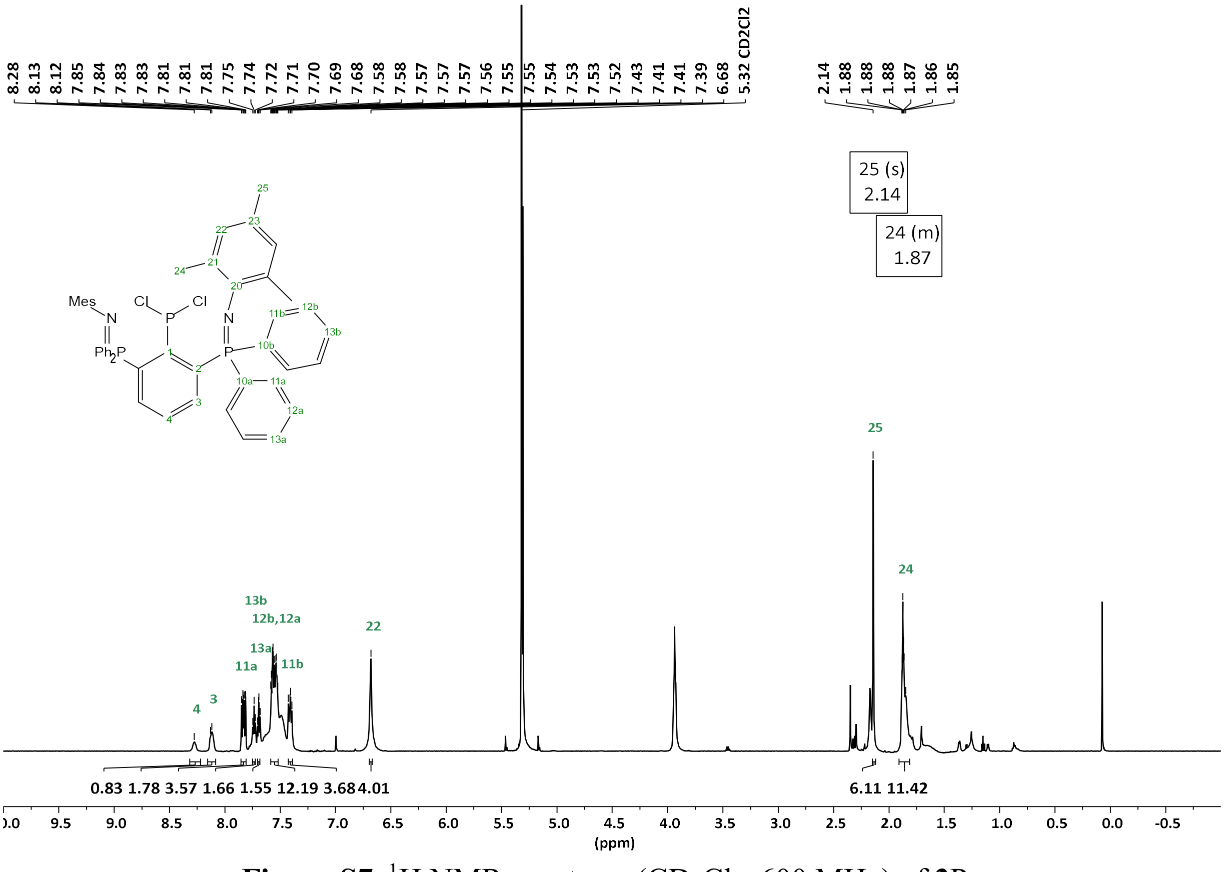

Figure S7: ^1H NMR spectrum (CD_2Cl_2, 600 MHz) of **3P.**

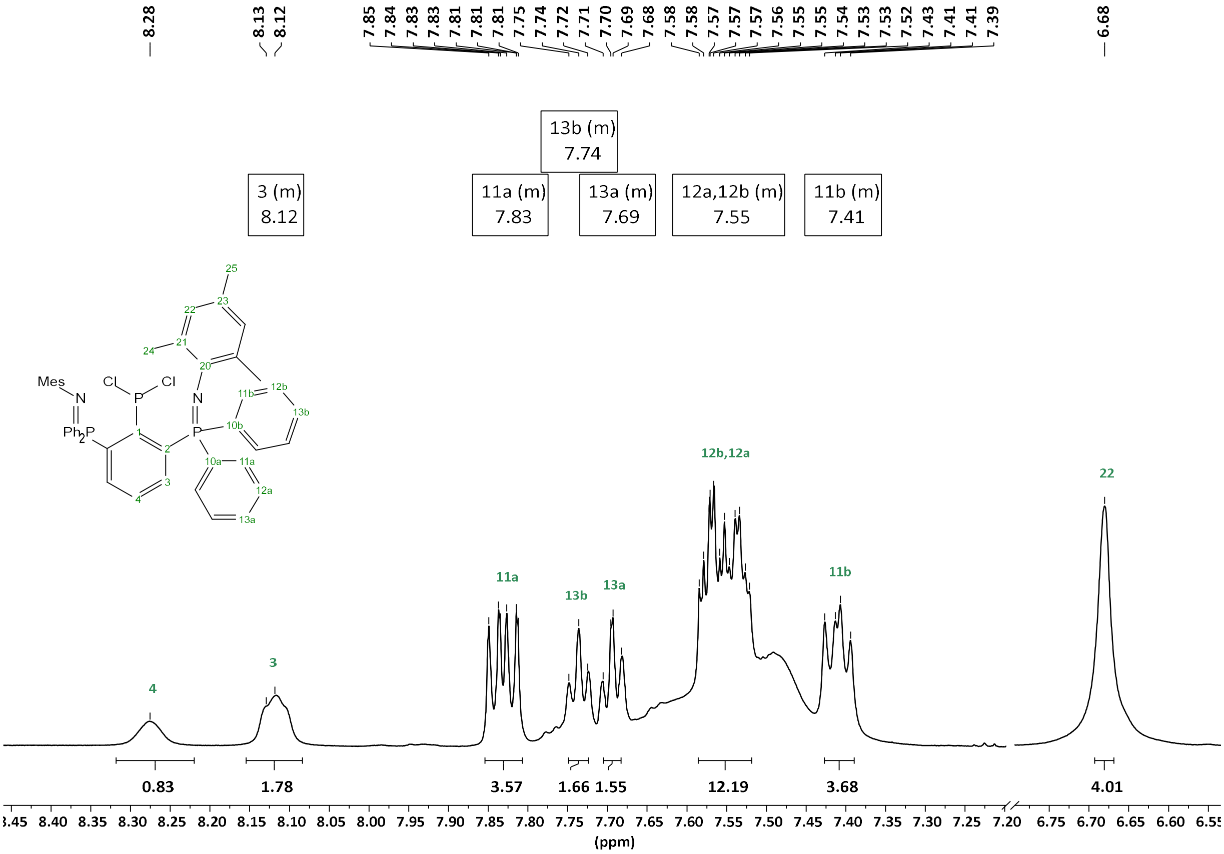

Figure S8: Detailed ^{1}H NMR spectrum (CD_2Cl_2, 600 MHz) of **3P.**

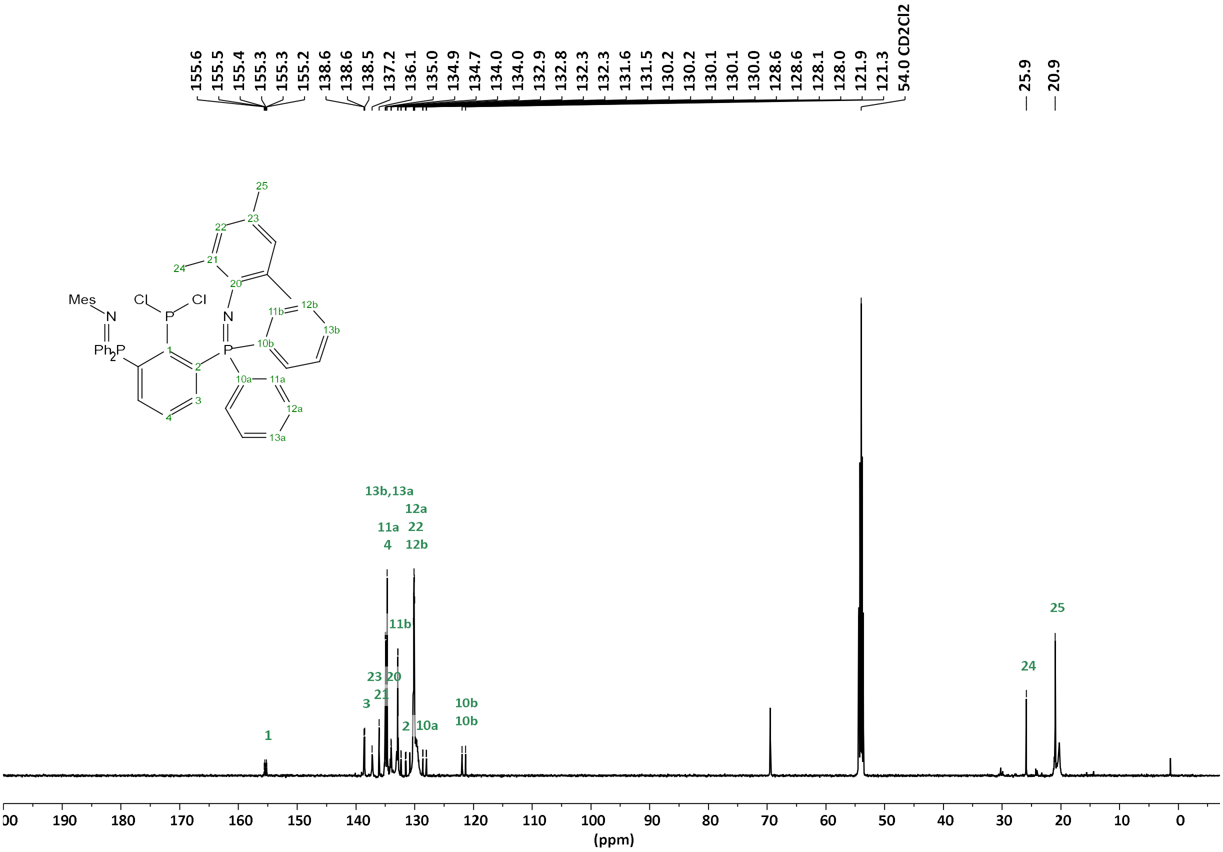

Figure S9: $^{13}C\{^{1}H\}$ NMR spectrum (CD_2Cl_2, 151 MHz) of **3P**.

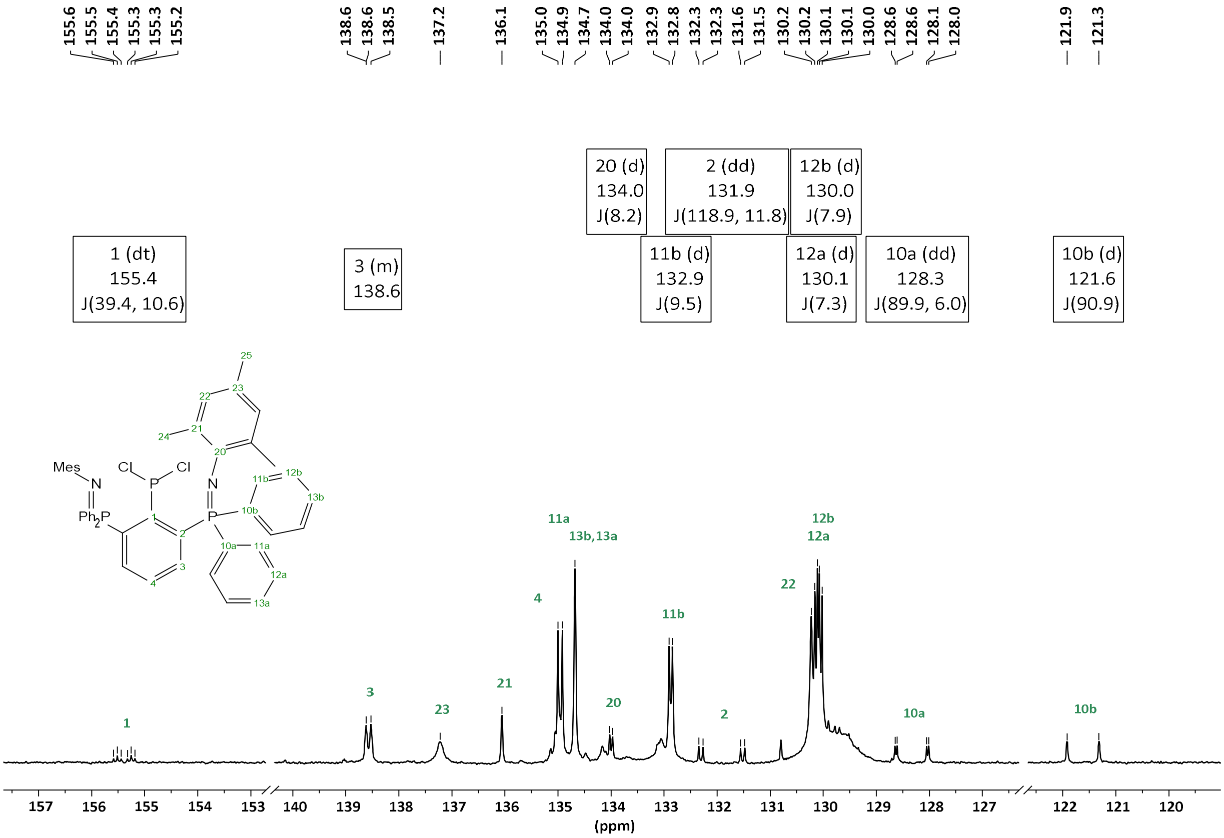

Figure S10: Detailed $^{13}C\{^1H\}$ NMR spectrum (CD_2Cl_2, 151 MHz) of **3P**.

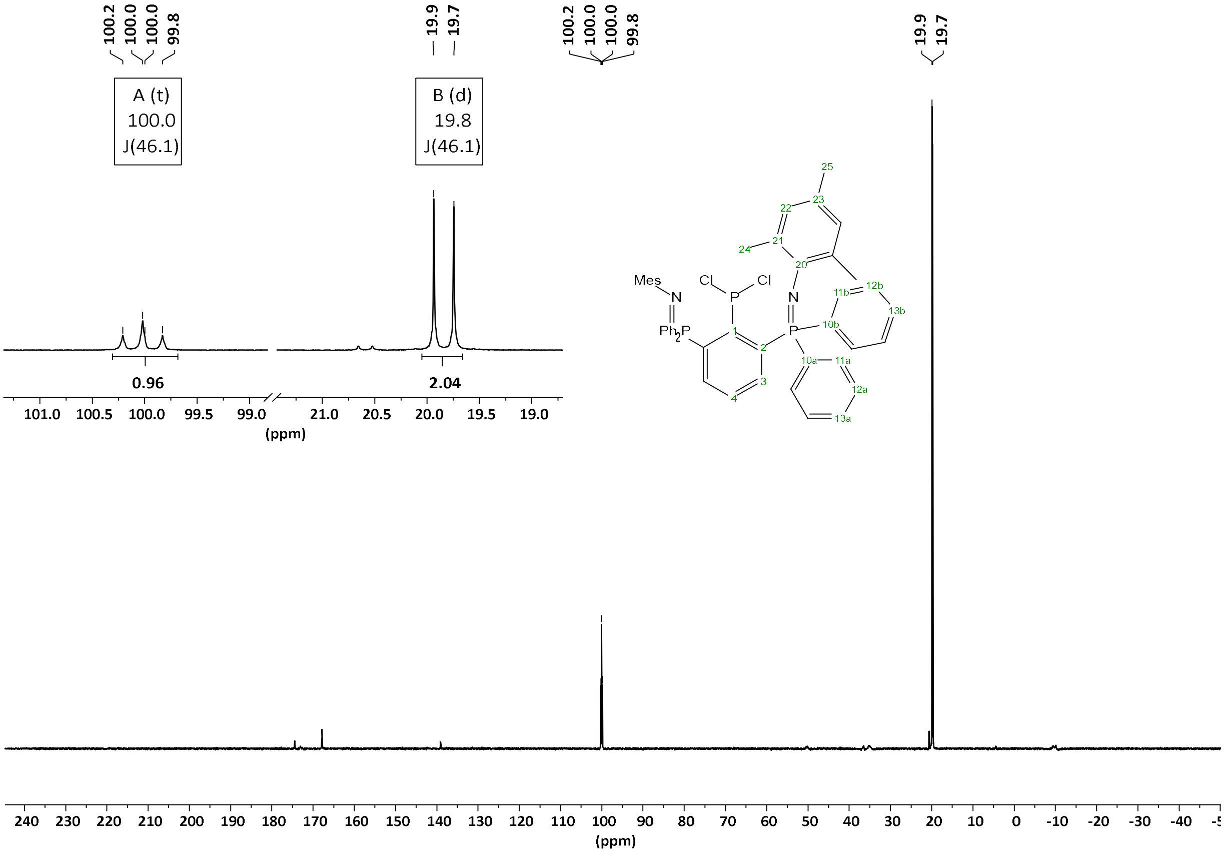

Figure S11: $^{31}P\{^1H\}$ NMR spectrum (CD_2Cl_2, 151 MHz) of **3P**.

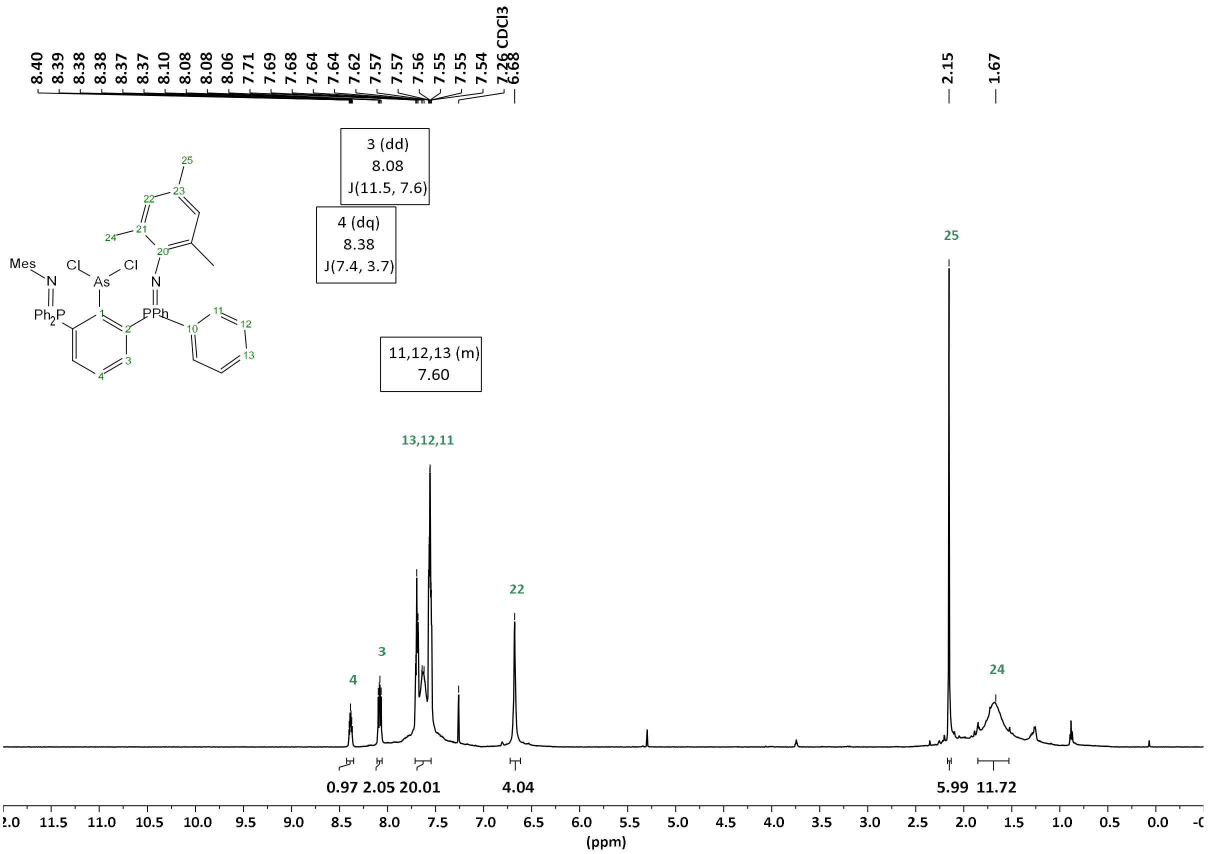

Figure S12: ^{1}H NMR spectrum ($CDCl_3$, 600 MHz) of **3As.**

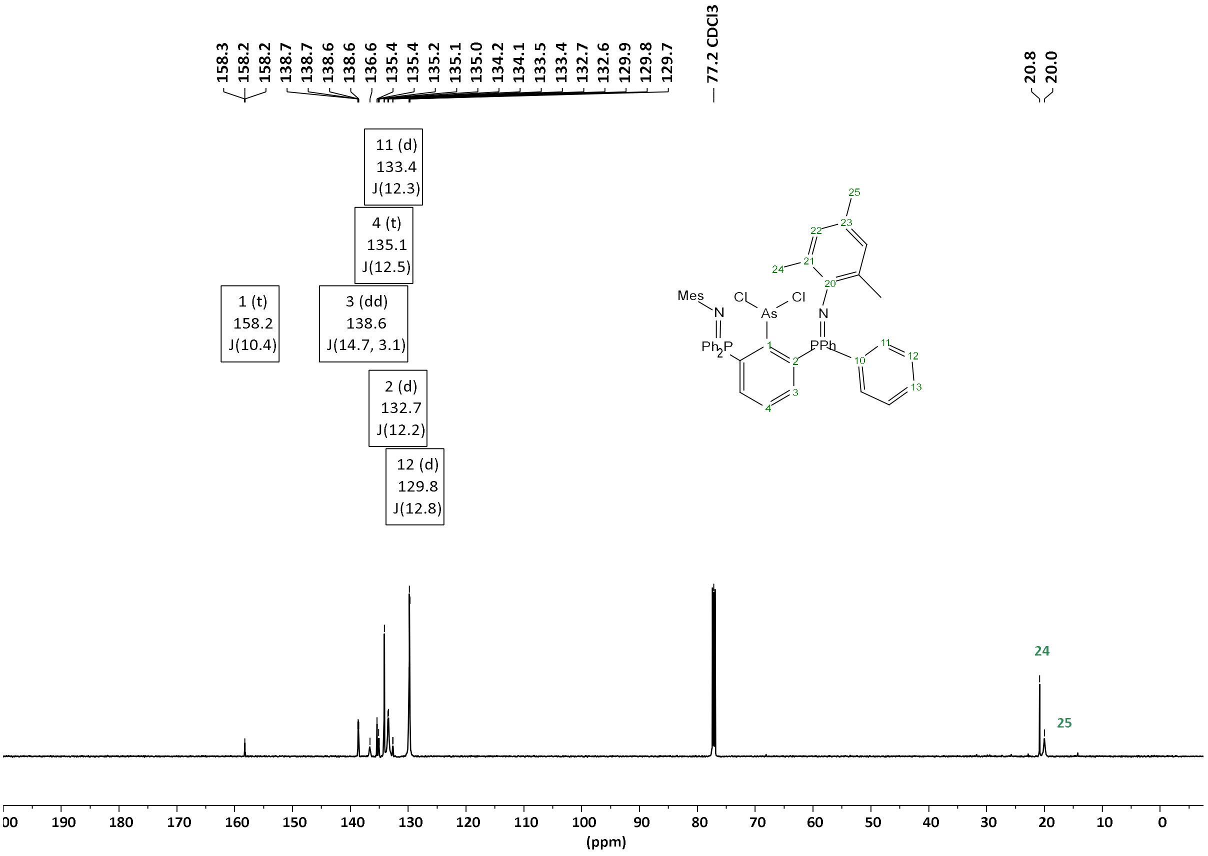

Figure S13: $^{13}C\{^{1}H\}$ NMR spectrum ($CDCl_3$, 151 MHz) of **3As**.

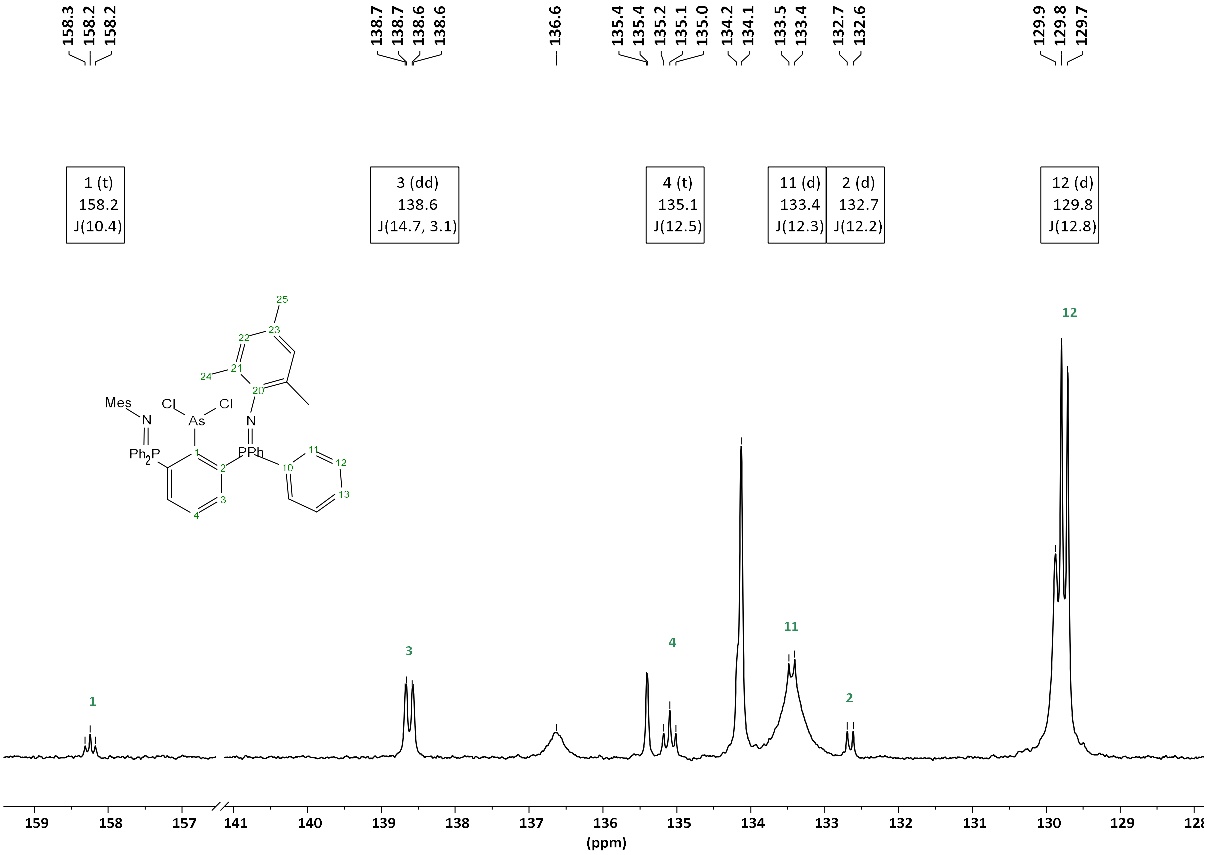

Figure S14: Detailed $^{13}C\{^{1}H\}$ NMR spectrum ($CDCl_3$, 151 MHz) of **3As**.

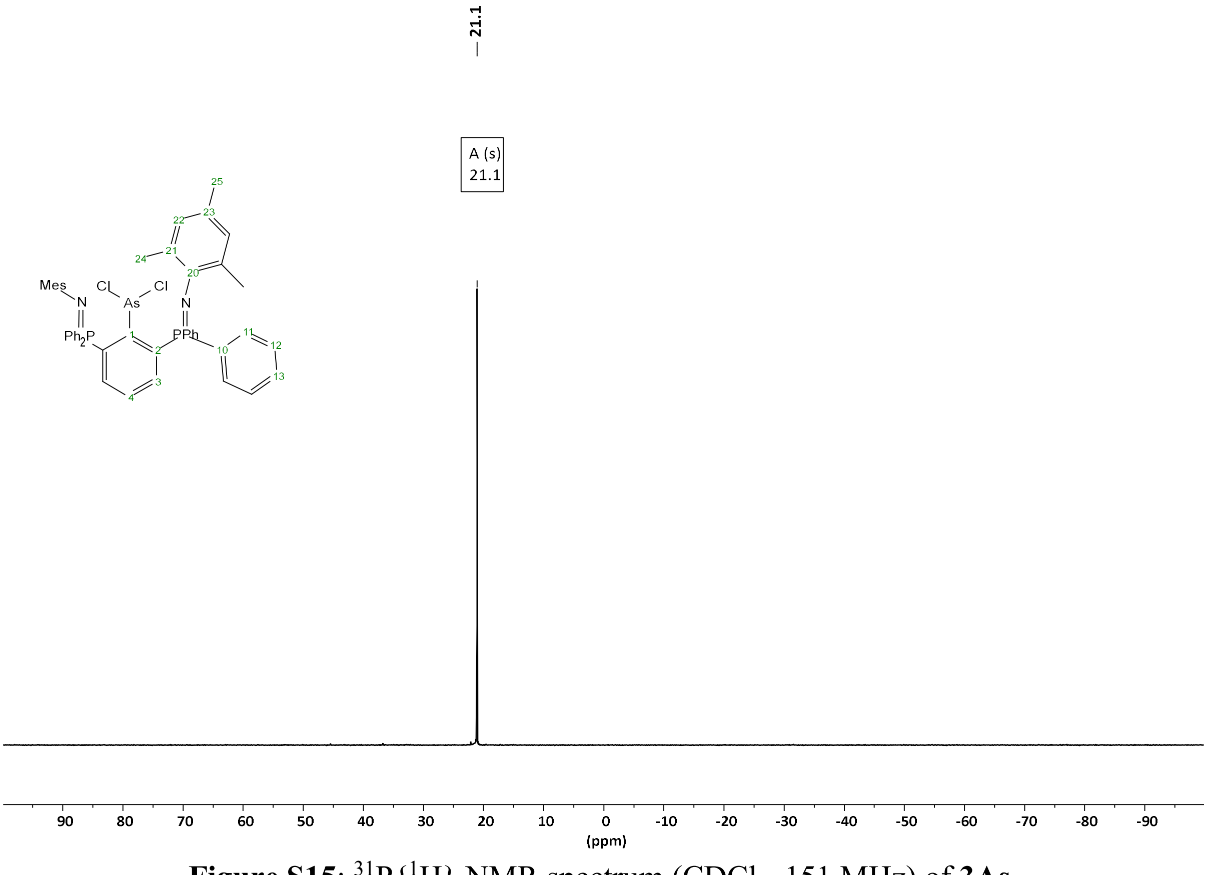

Figure S15: $^{31}P\{^{1}H\}$ NMR spectrum ($CDCl_3$, 151 MHz) of **3As**.

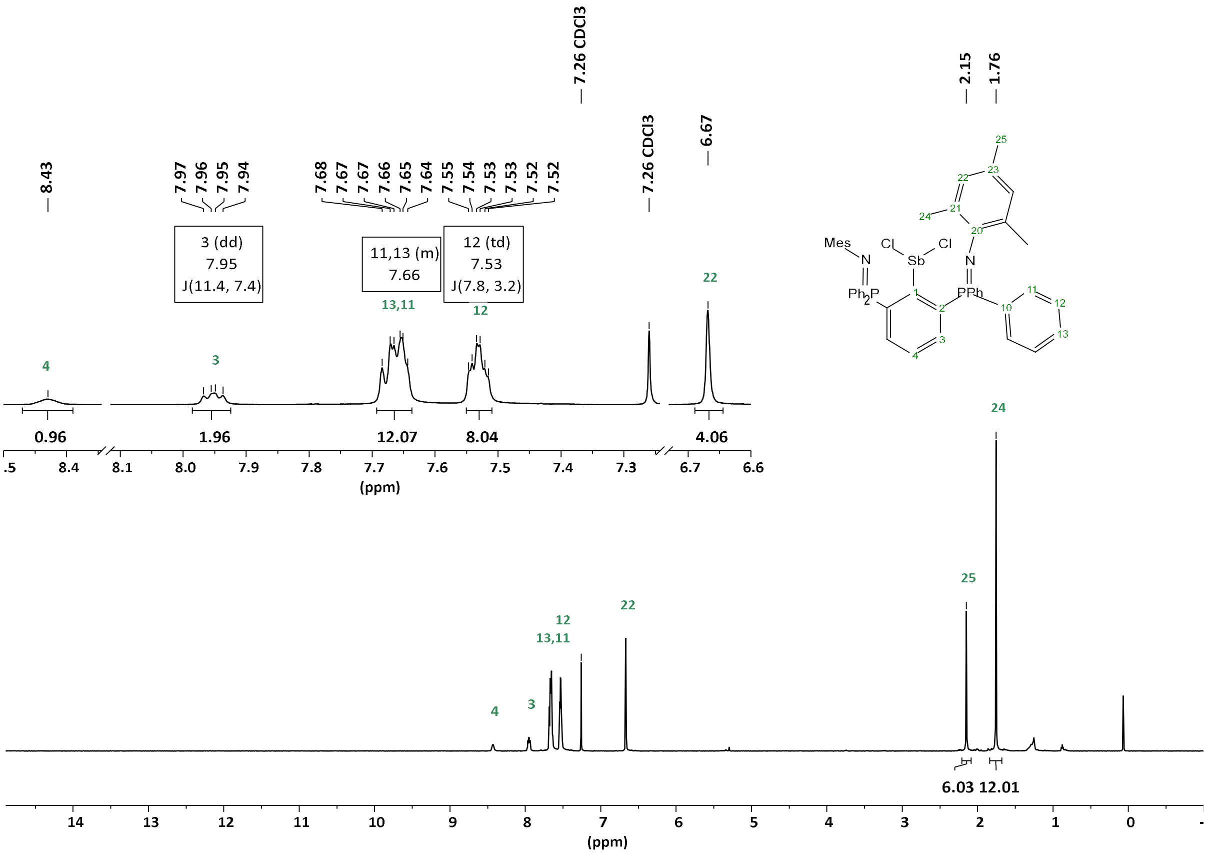

Figure S16: ^{1}H NMR spectrum ($CDCl_3$, 600 MHz) of **3Sb.**

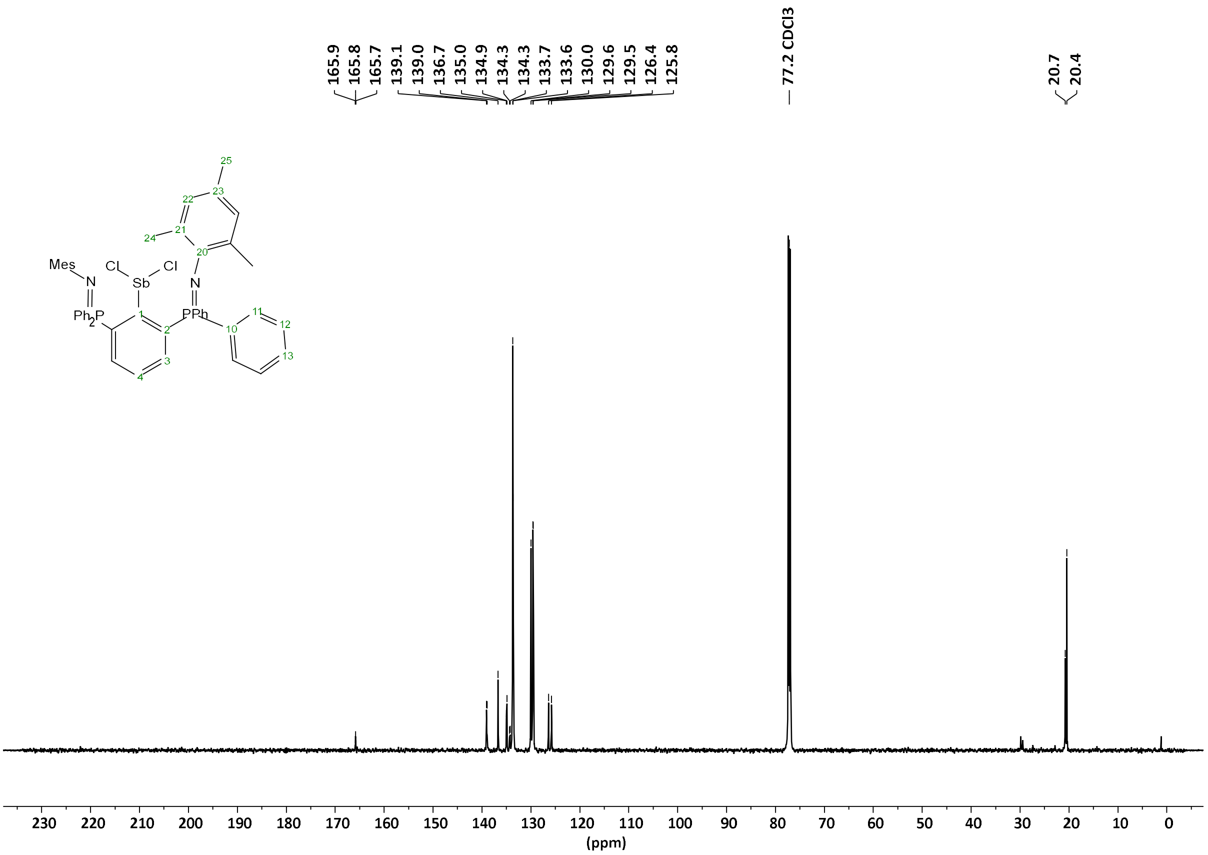

Figure S17: $^{13}C\{^{1}H\}$ NMR spectrum ($CDCl_3$, 151 MHz) of **3Sb**.

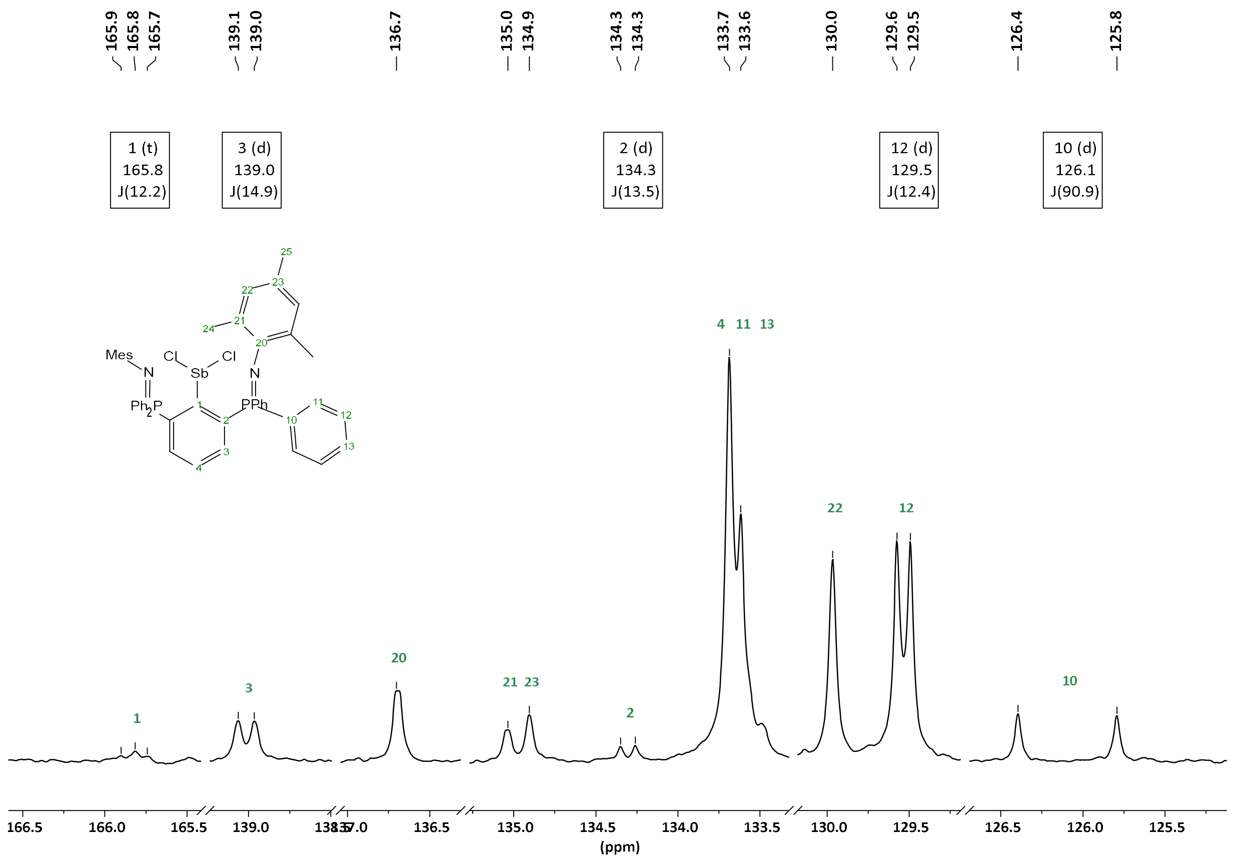

Figure S18: Detailed $^{13}C\{^{1}H\}$ NMR spectrum ($CDCl_3$, 151 MHz) of **3Sb**.

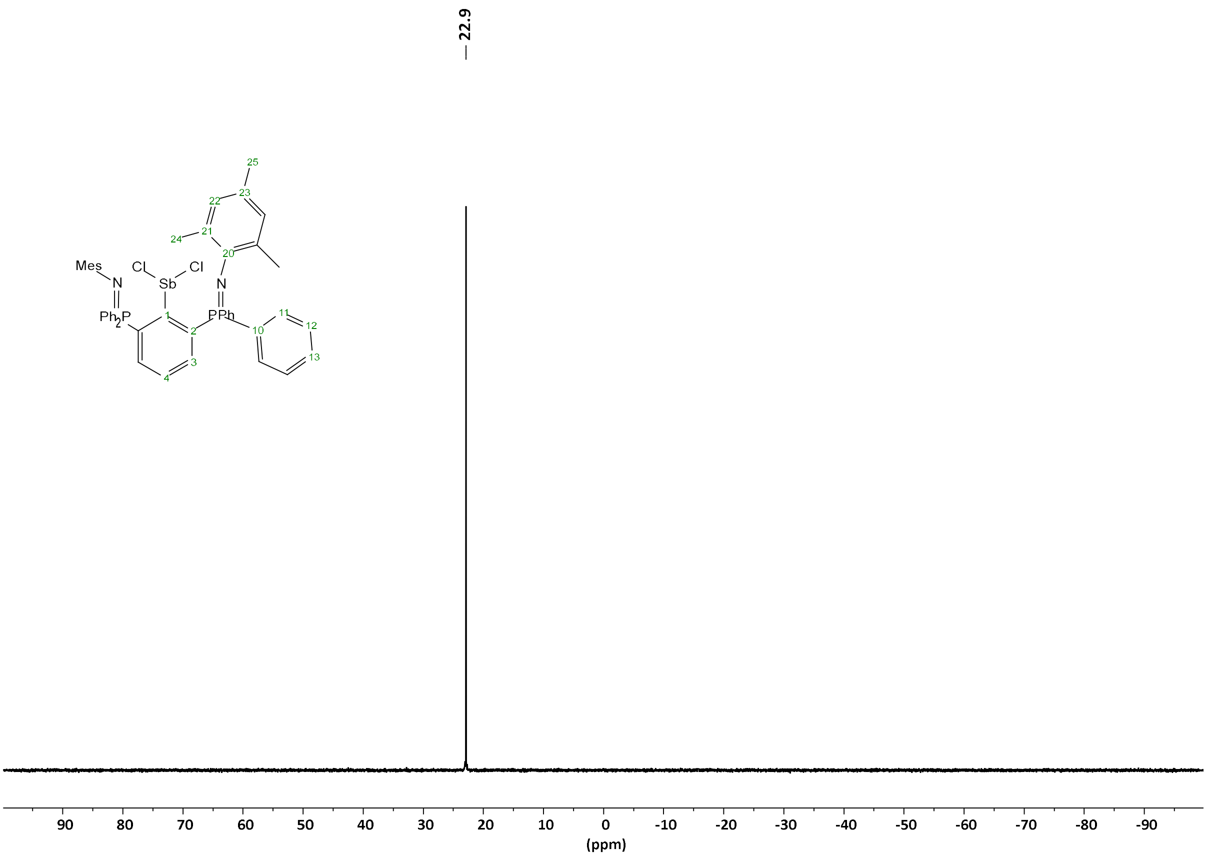

Figure S19: $^{31}P\{^{1}H\}$ NMR spectrum ($CDCl_3$, 151 MHz) of **3Sb**.

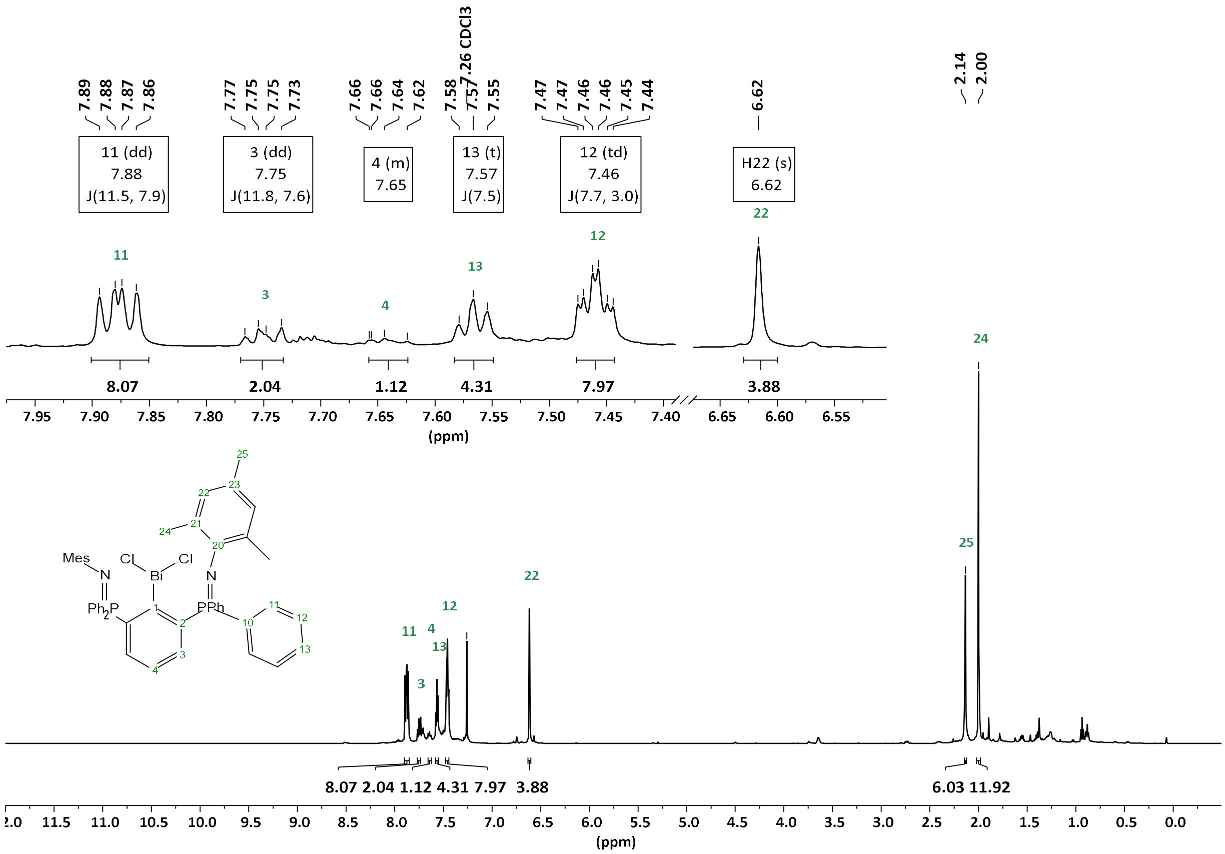

Figure S20: ^{1}H NMR spectrum ($CDCl_3$, 600 MHz) of **3Bi.**

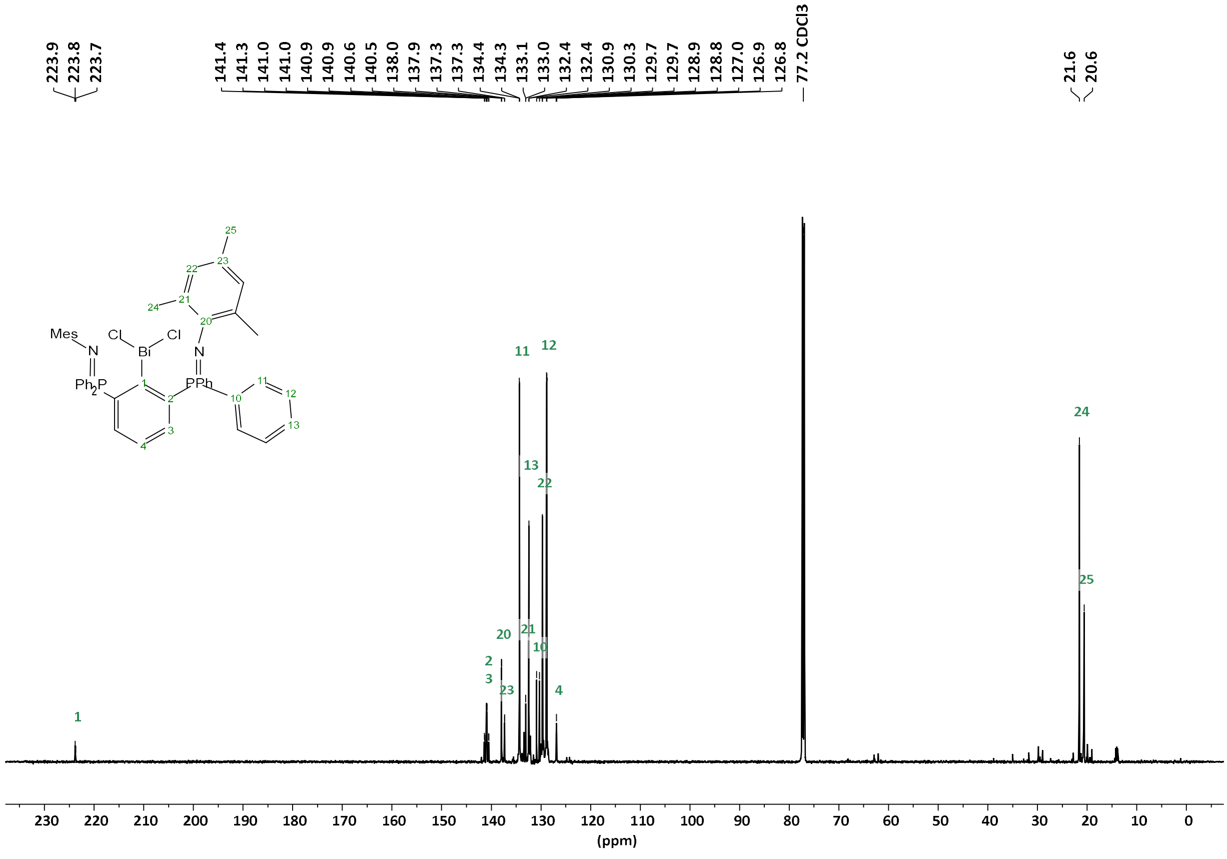

Figure S21: $^{13}C\{^{1}H\}$ NMR spectrum ($CDCl_3$, 151 MHz) of **3Bi**.

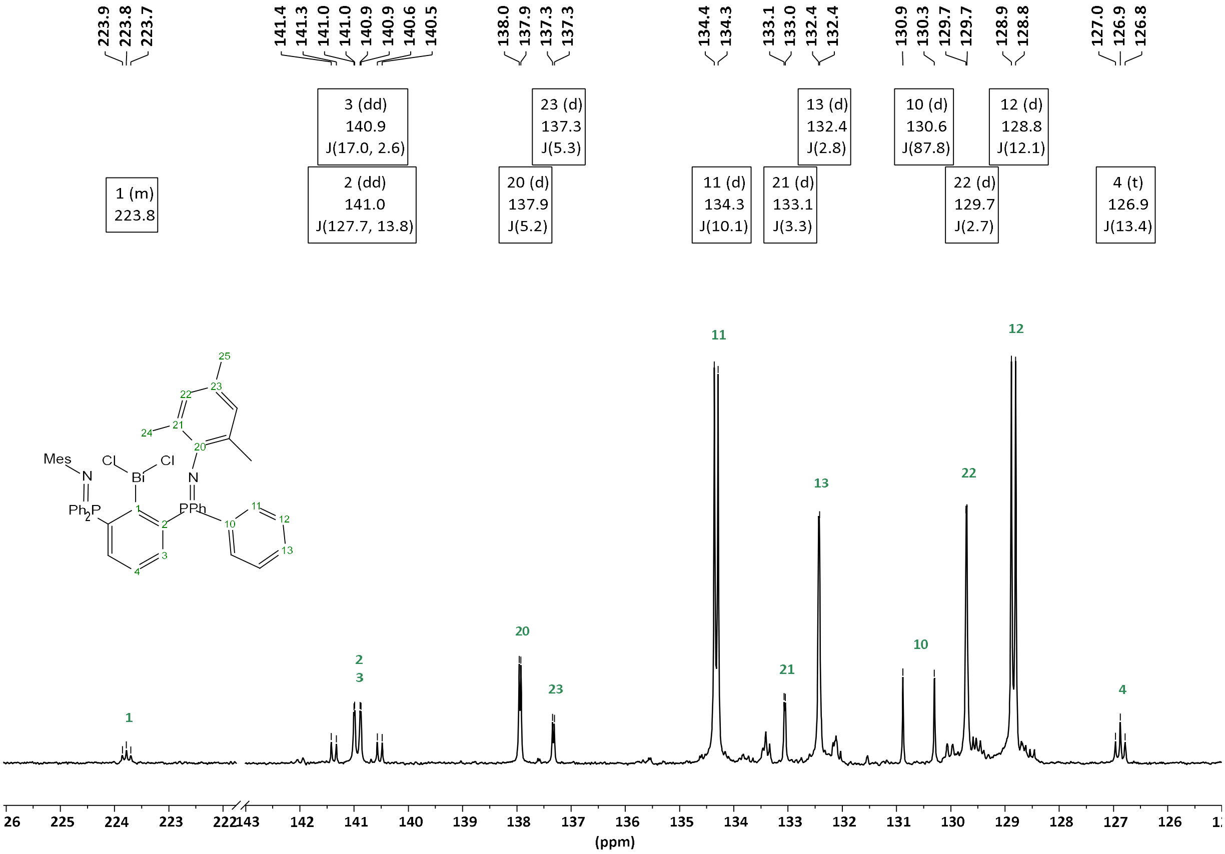

Figure S22: Detailed $^{13}C\{^{1}H\}$ NMR spectrum ($CDCl_3$, 151 MHz) of **3Bi**.

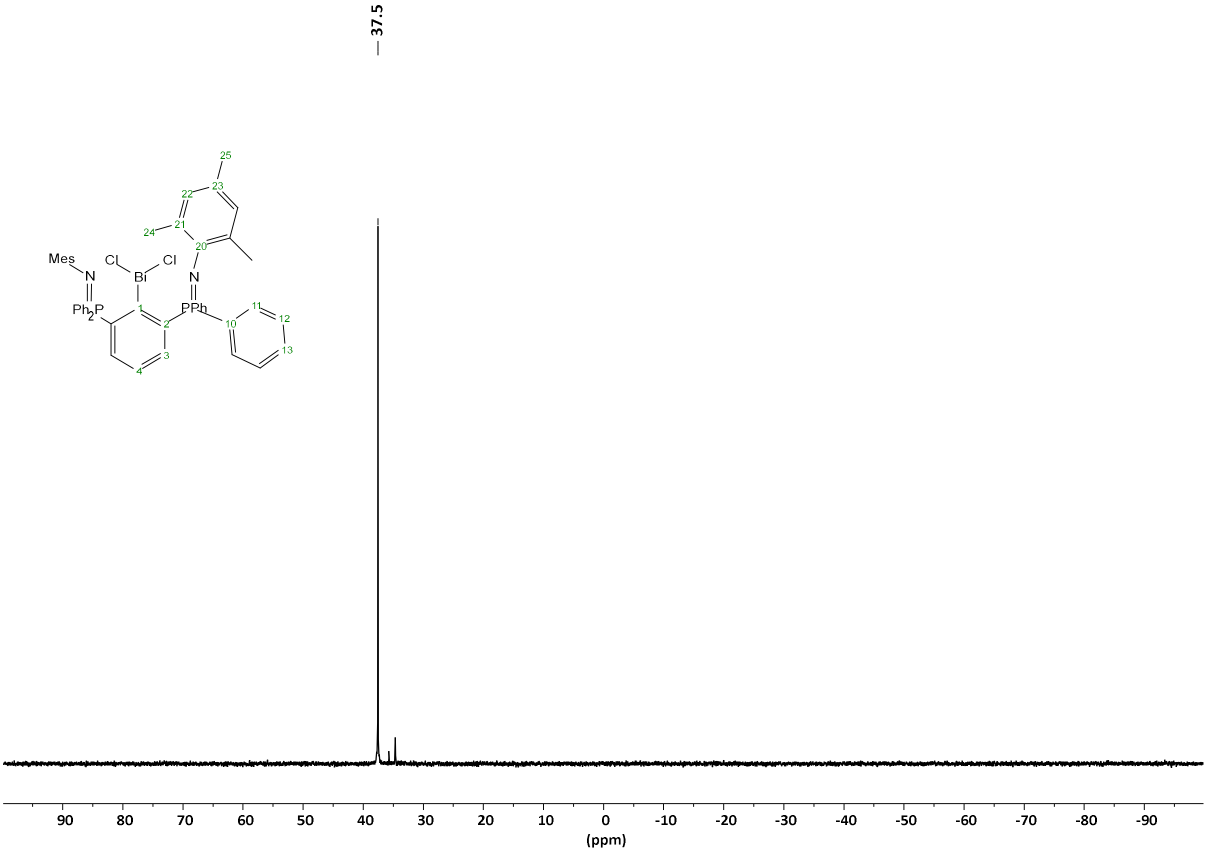

Figure S23: $^{31}P\{^{1}H\}$ NMR spectrum ($CDCl_3$, 151 MHz) of **3Bi**.

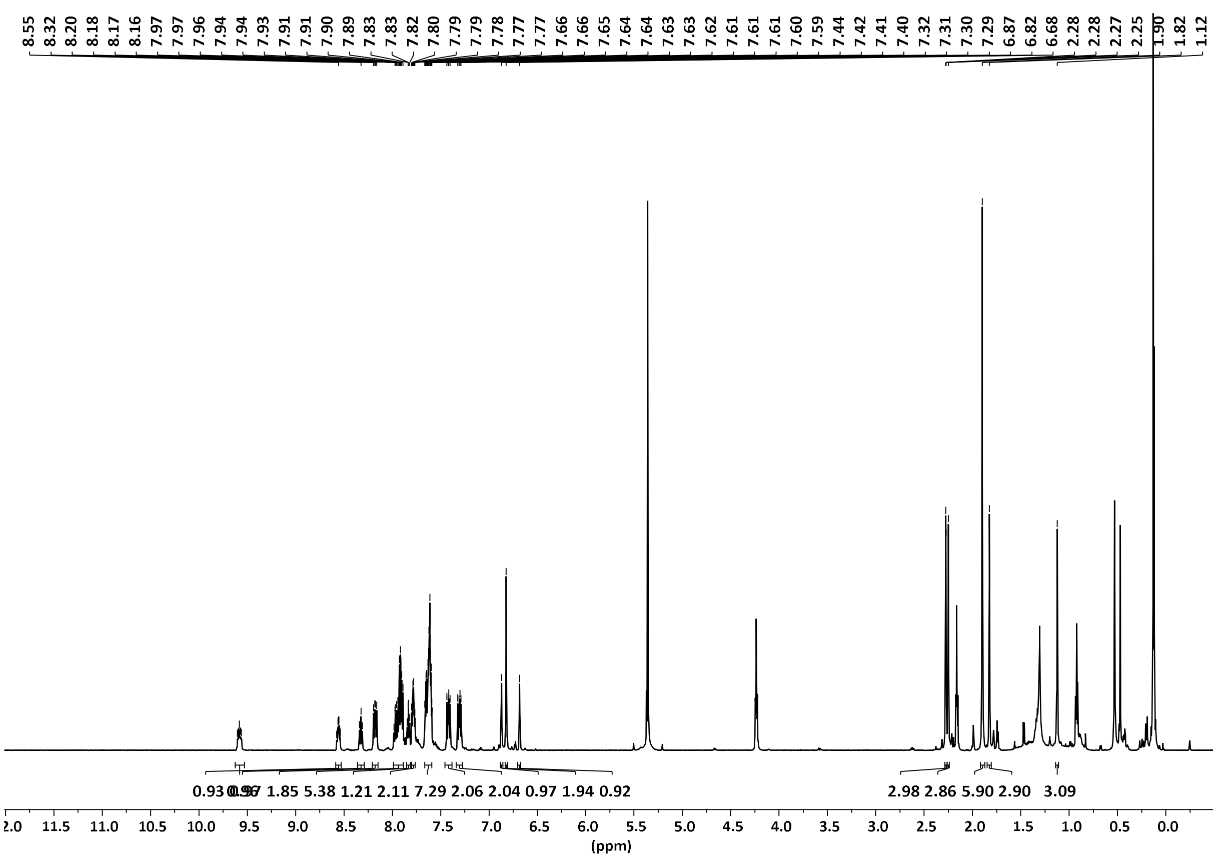

Figure S24: ^{1}H NMR spectrum (CD_2Cl_2, 600 MHz) of [**4P**]OTf.

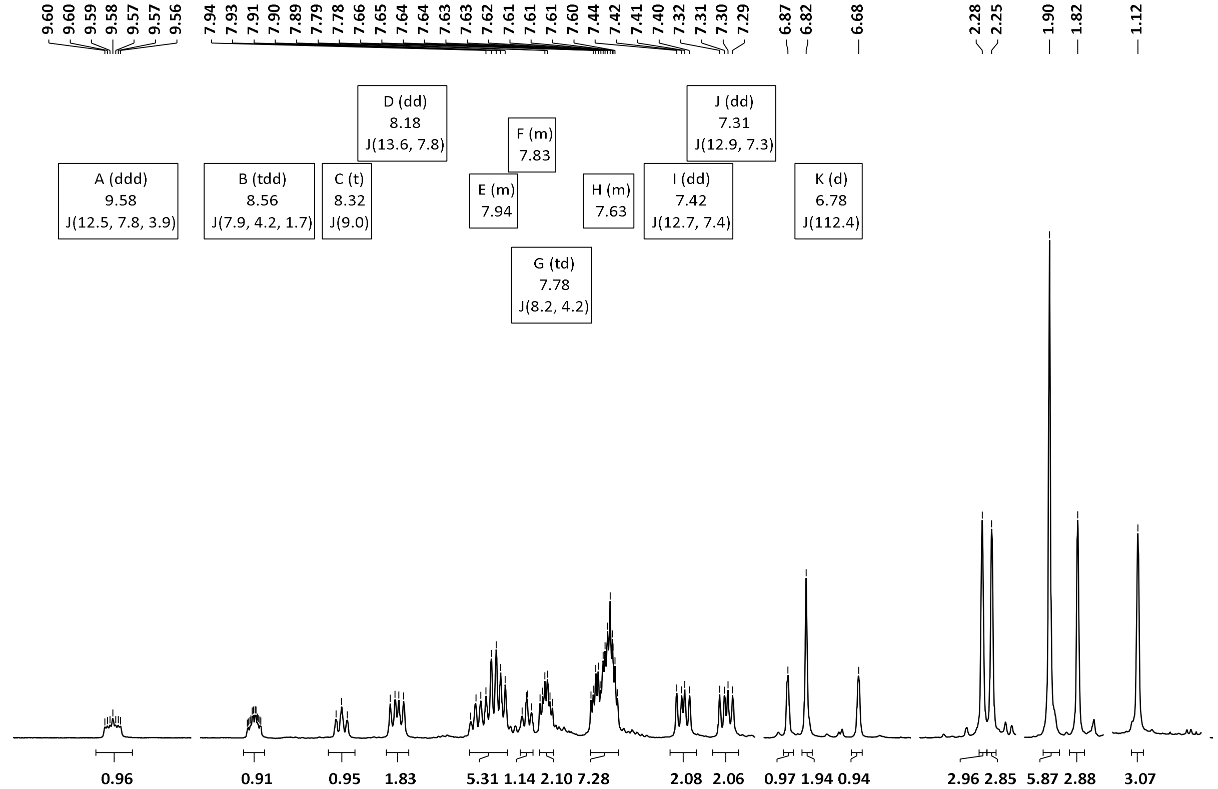

Figure S25: Detailed ^{1}H NMR spectrum (CD_2Cl_2, 600 MHz) of [**4P**]OTf.

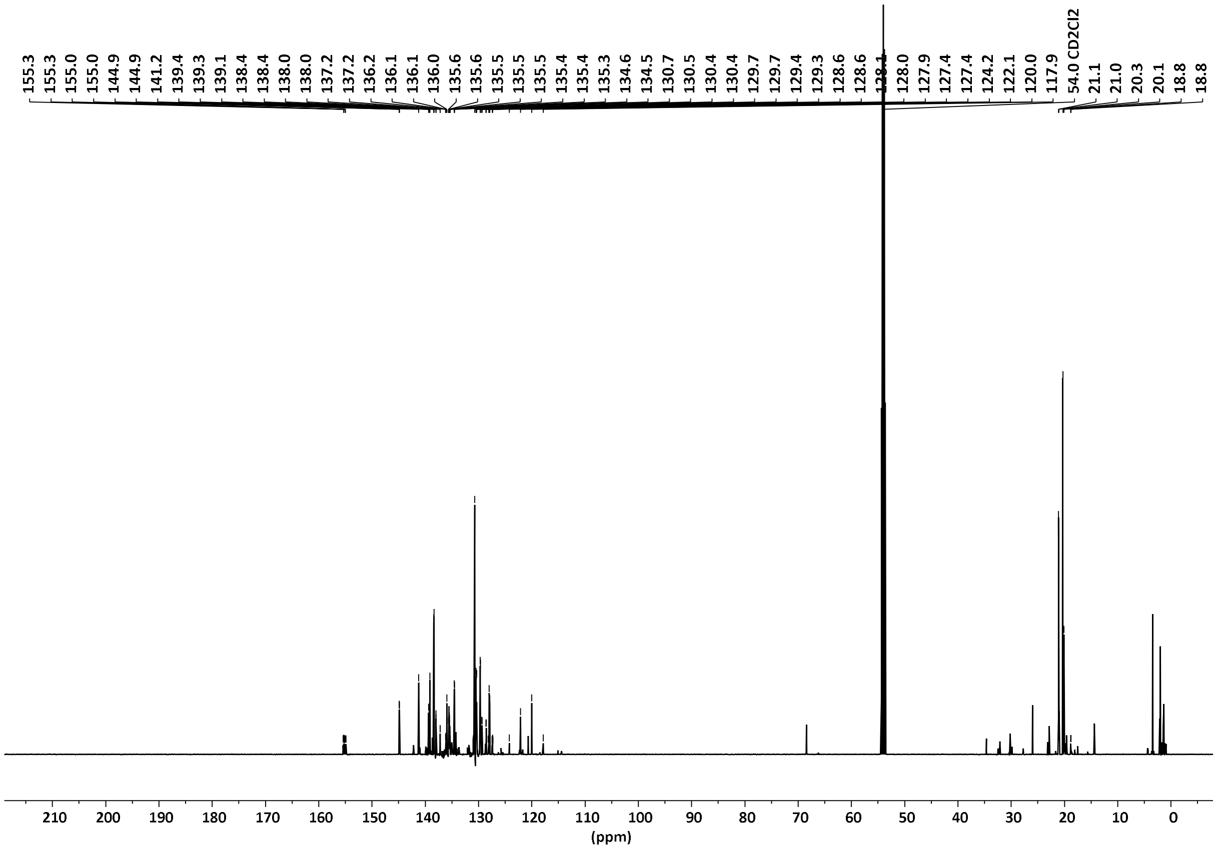

Figure S26: $^{13}C\{^{1}H\}$ NMR spectrum (CD_2Cl_2, 151 MHz) of [**4P**]OTf.

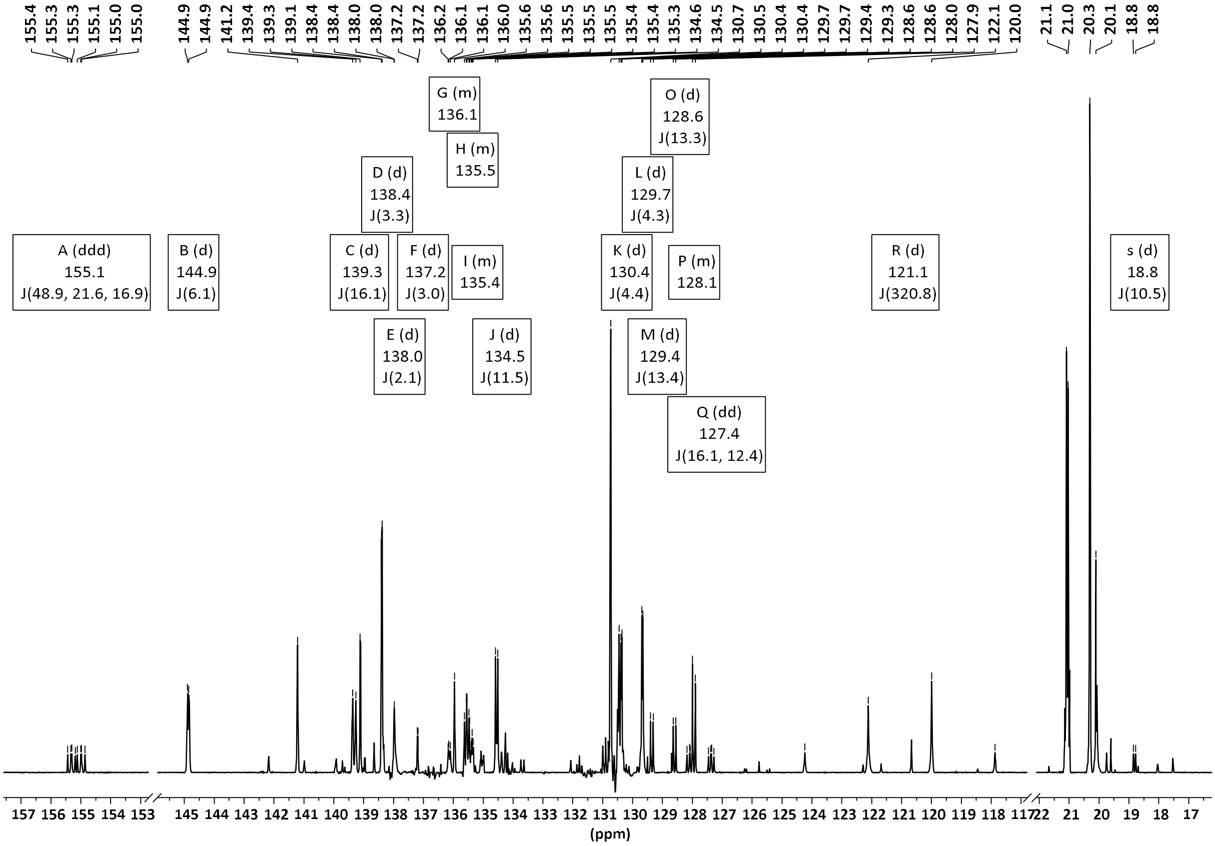

Figure S27: Detailed $^{13}C\{^{1}H\}$ NMR spectrum (CD_2Cl_2, 151 MHz) of [**4P**]OTf.

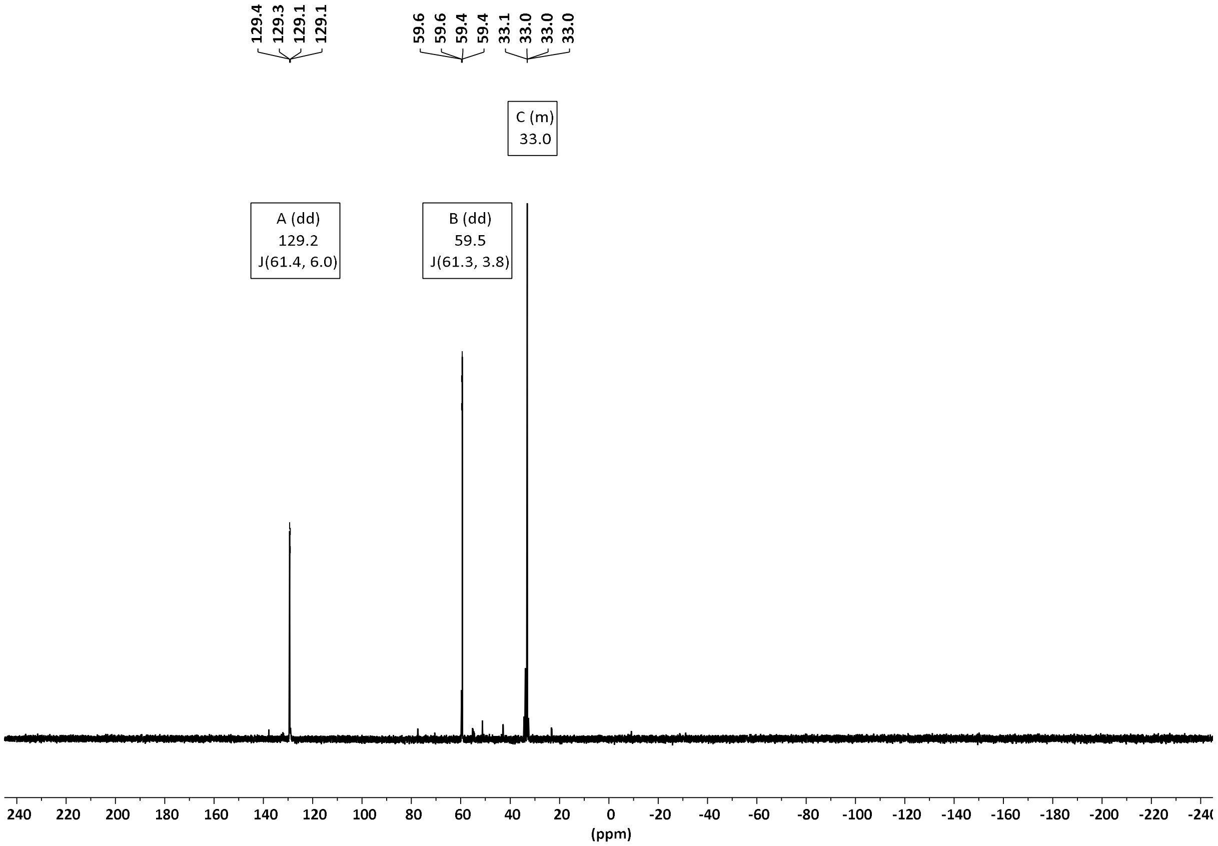

Figure S28: $^{31}P\{^{1}H\}$ NMR spectrum (CD_2Cl_2, 151 MHz) of [**4P**]OTf.

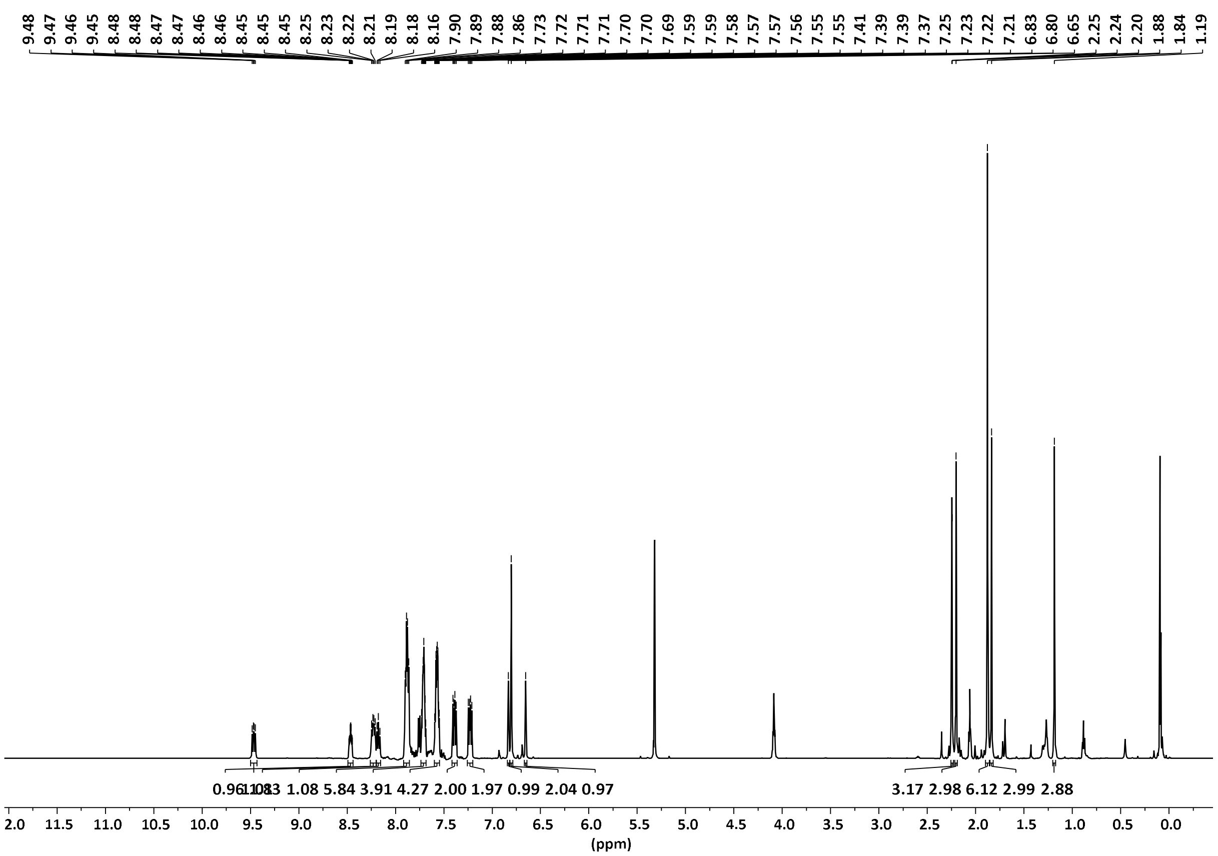

Figure S29: ^{1}H NMR spectrum (CD_2Cl_2, 600 MHz) of [**4As**]OTf.

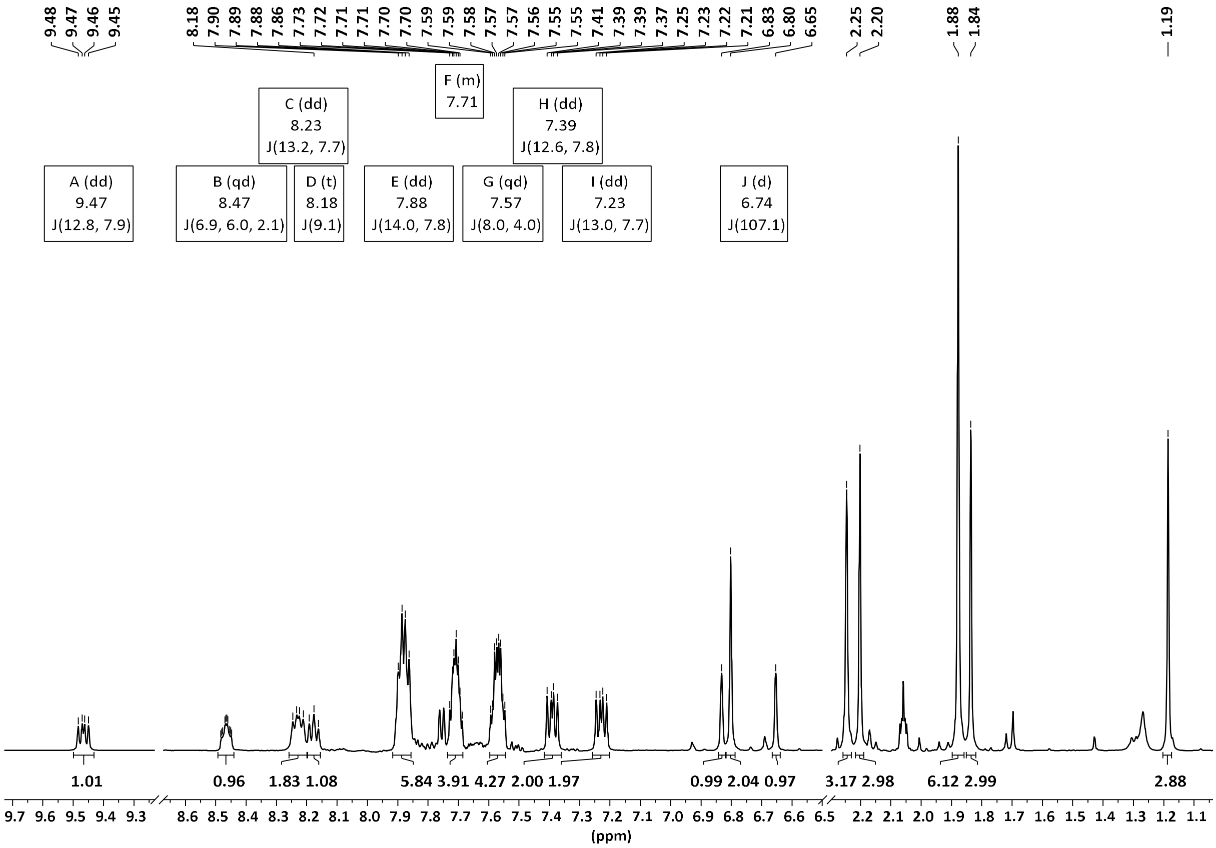

Figure S30: Detailed ^{1}H NMR spectrum (CD_2Cl_2, 600 MHz) of [**4As**]OTf.

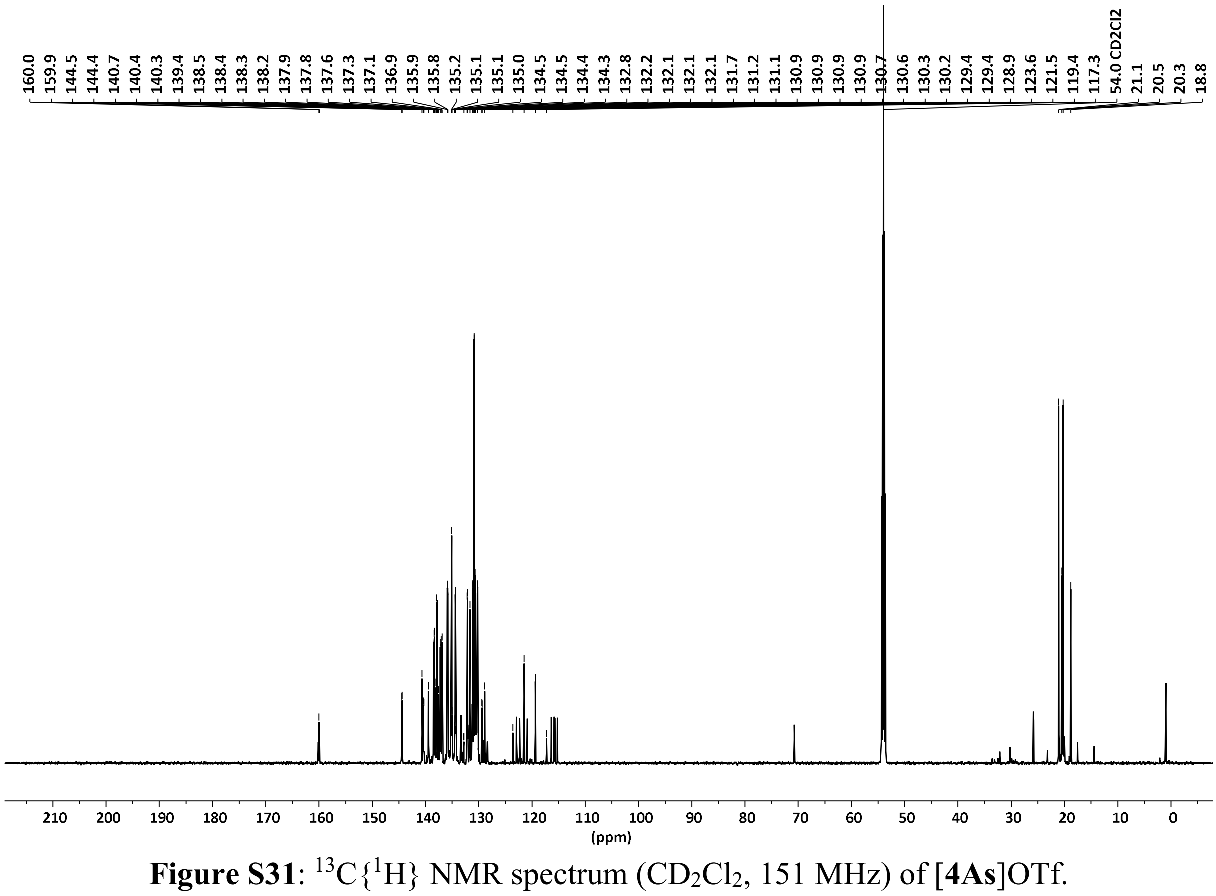

Figure S31: $^{13}C\{^{1}H\}$ NMR spectrum (CD_2Cl_2, 151 MHz) of [**4As**]OTf.

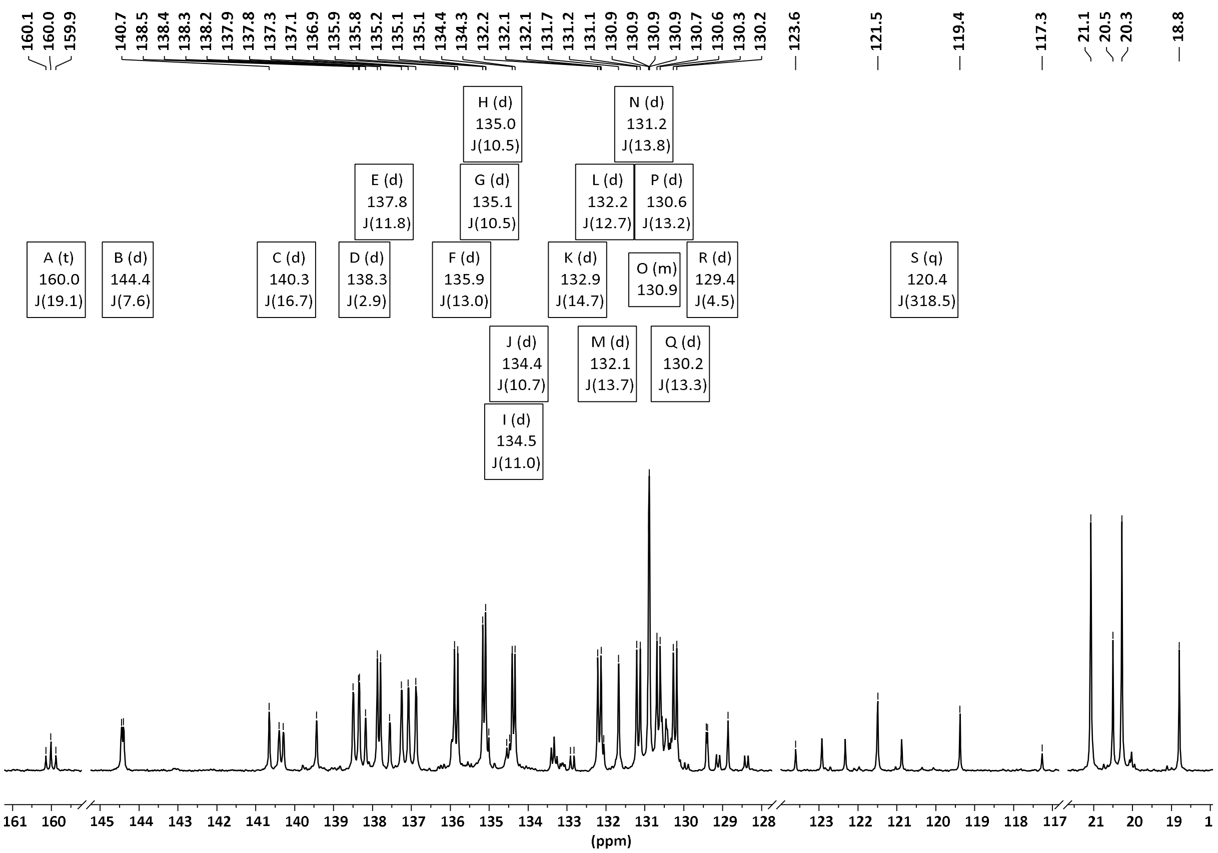

Figure S32: Detailed $^{13}C\{^{1}H\}$ NMR spectrum (CD_2Cl_2, 151 MHz) of [**4As**]OTf.

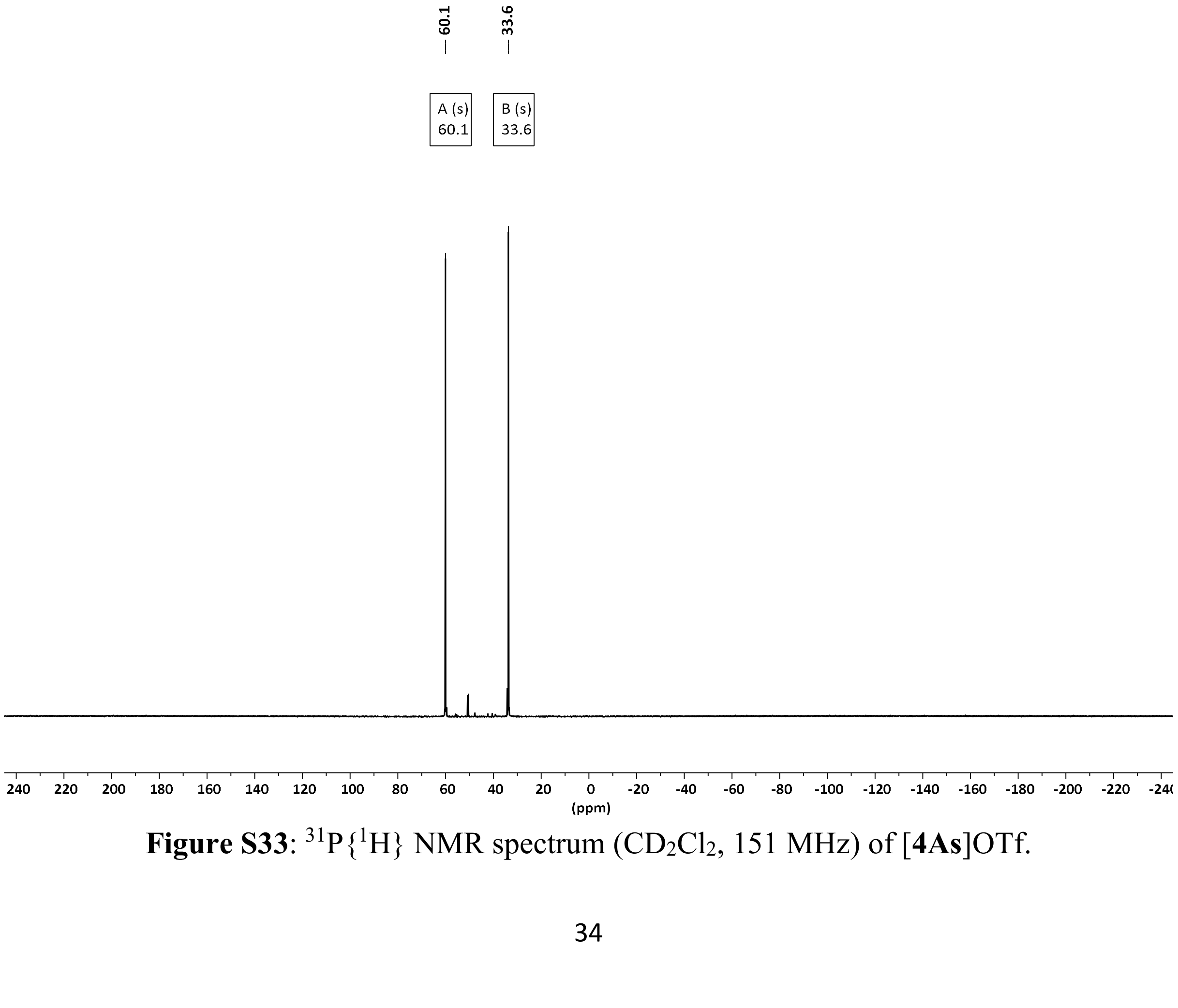

Figure S33: $^{31}P\{^{1}H\}$ NMR spectrum (CD_2Cl_2, 151 MHz) of [**4As**]OTf.

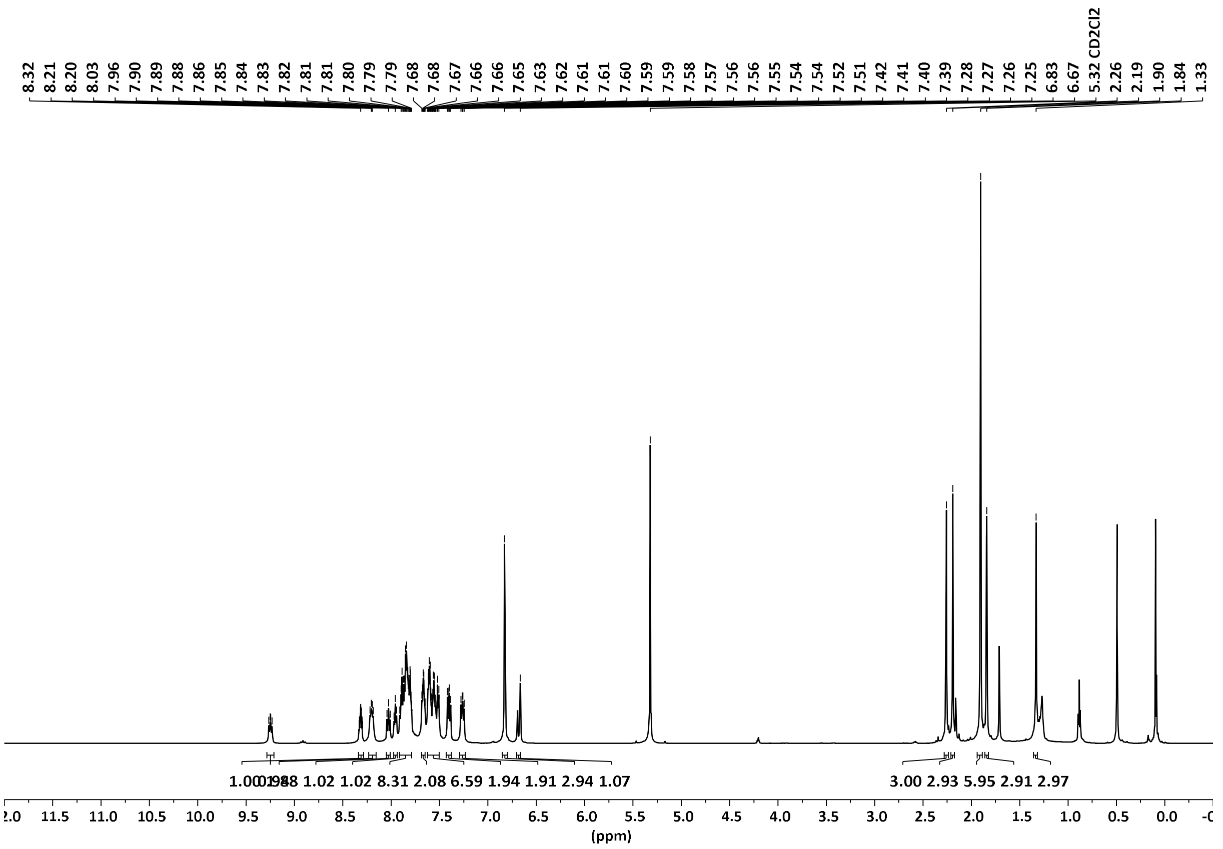

Figure S34: ^{1}H NMR spectrum (CD_2Cl_2, 600 MHz) of [**4Sb**]OTf.

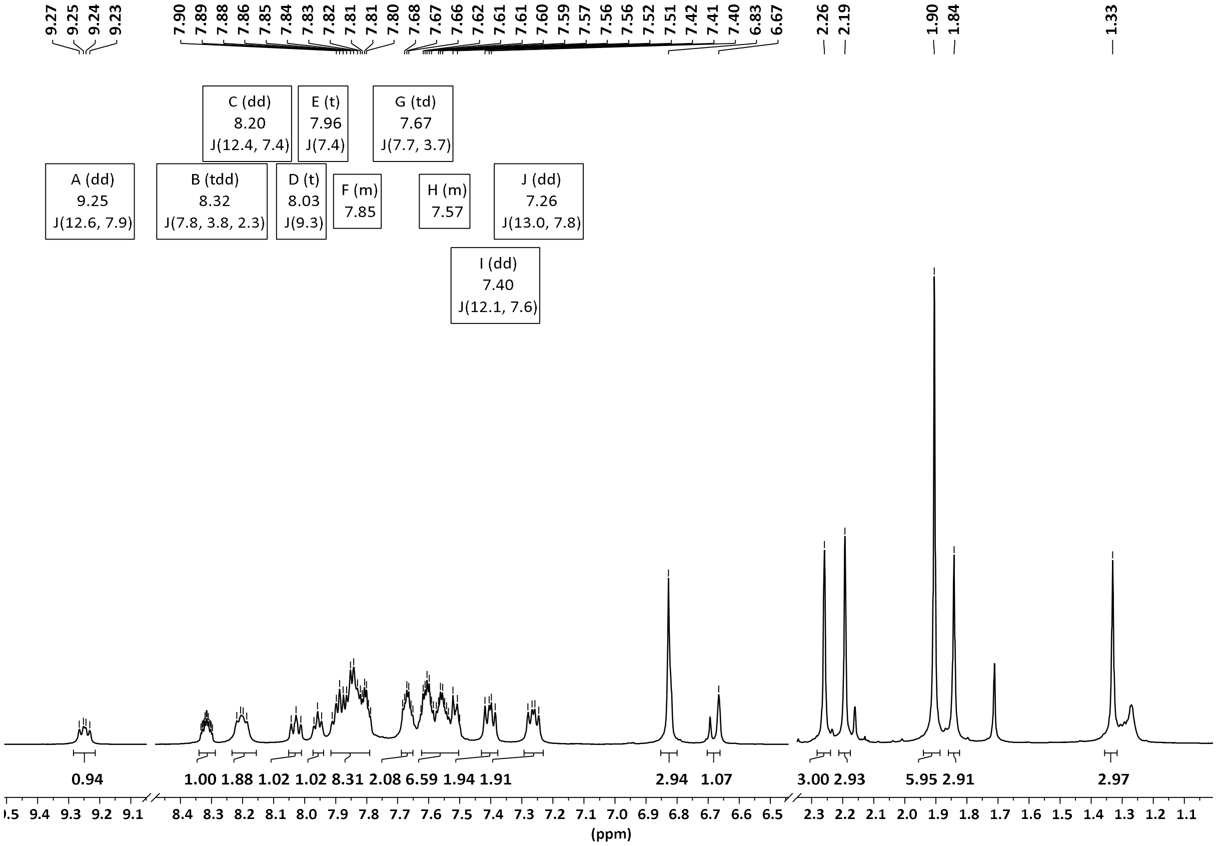

Figure S35: Detailed ^{1}H NMR spectrum (CD_2Cl_2, 600 MHz) of [**4Sb**]OTf.

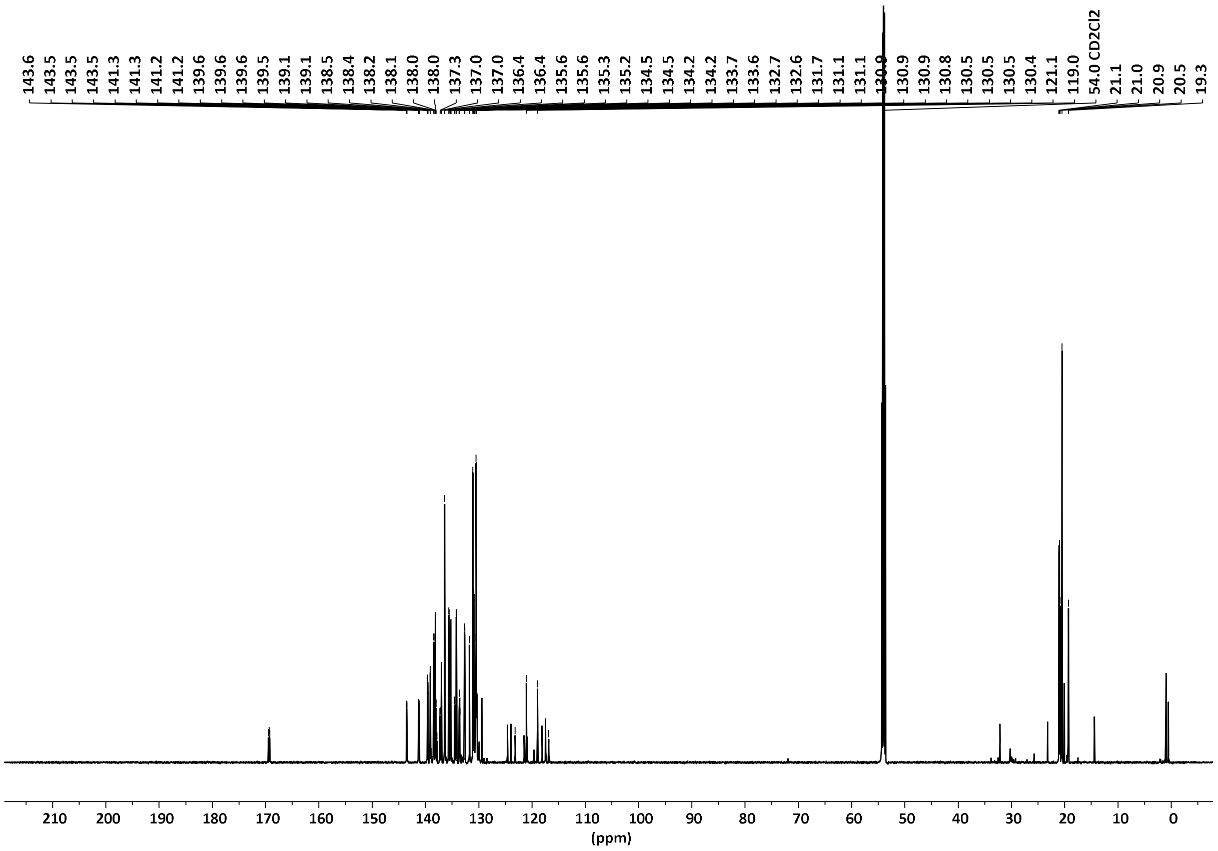

Figure S36: $^{13}C\{^{1}H\}$ NMR spectrum (CD_2Cl_2, 151 MHz) of [**4Sb**]OTf.

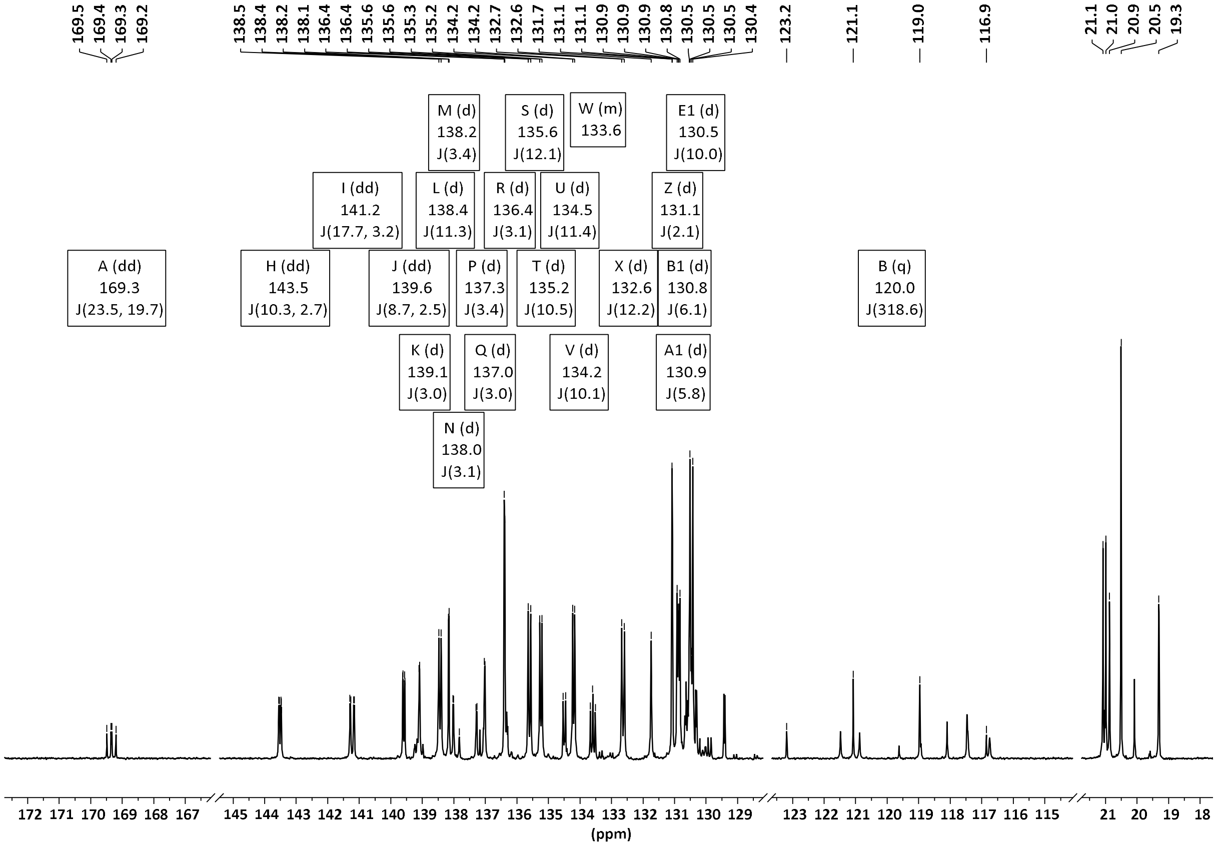

Figure S37: Detailed $^{13}C\{^{1}H\}$ NMR spectrum (CD_2Cl_2, 151 MHz) of [**4Sb**]OTf.

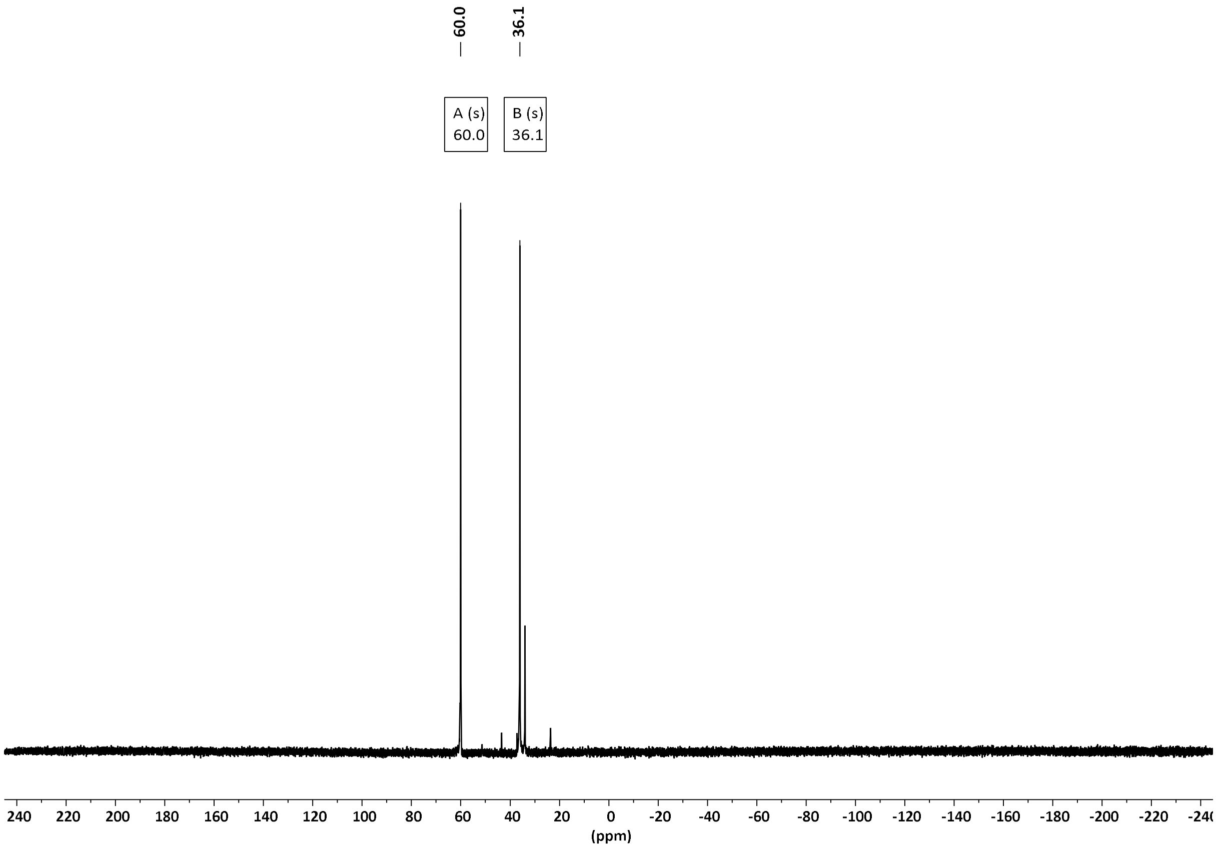

Figure S38: $^{31}P\{^1H\}$ NMR spectrum (CD_2Cl_2, 151 MHz) of [**4Sb**]OTf.

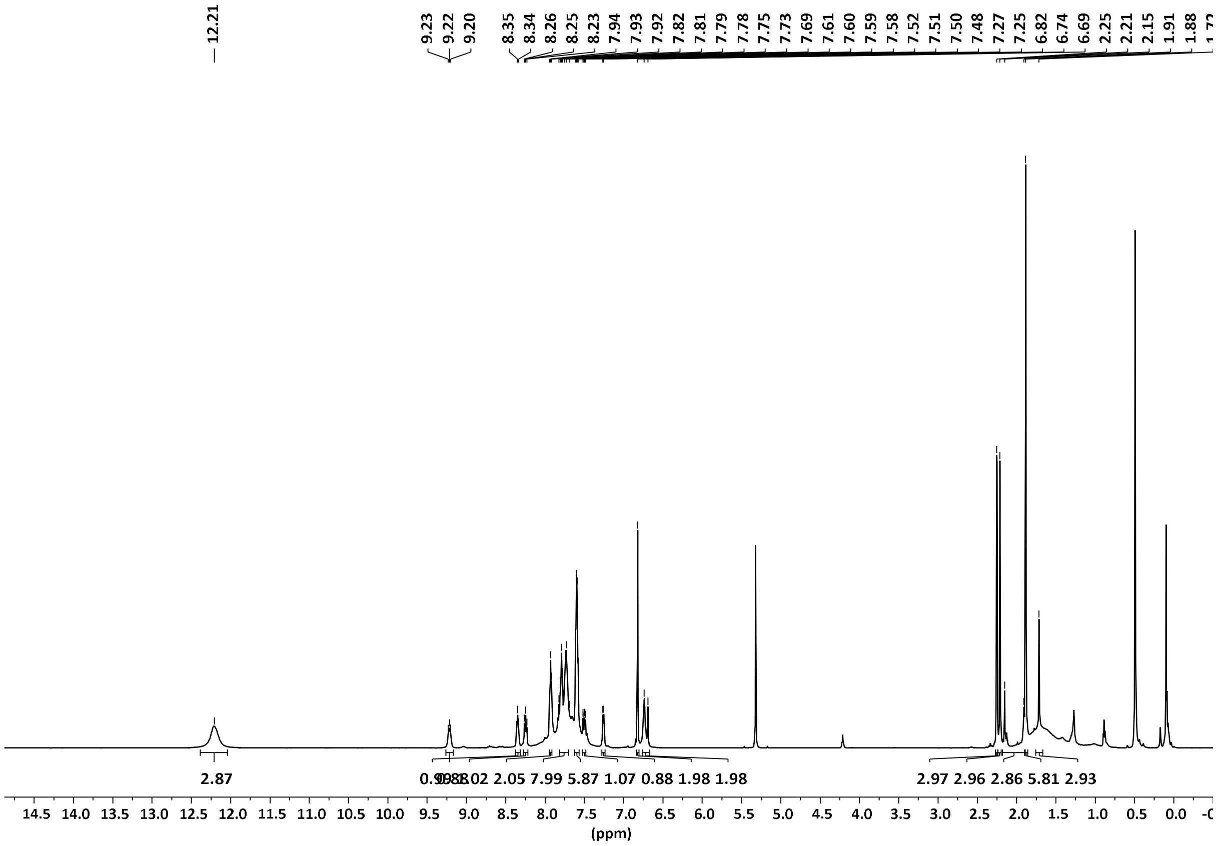

Figure S39: ^{1}H NMR spectrum (CD_2Cl_2, 600 MHz) of [**4Bi**]OTf.

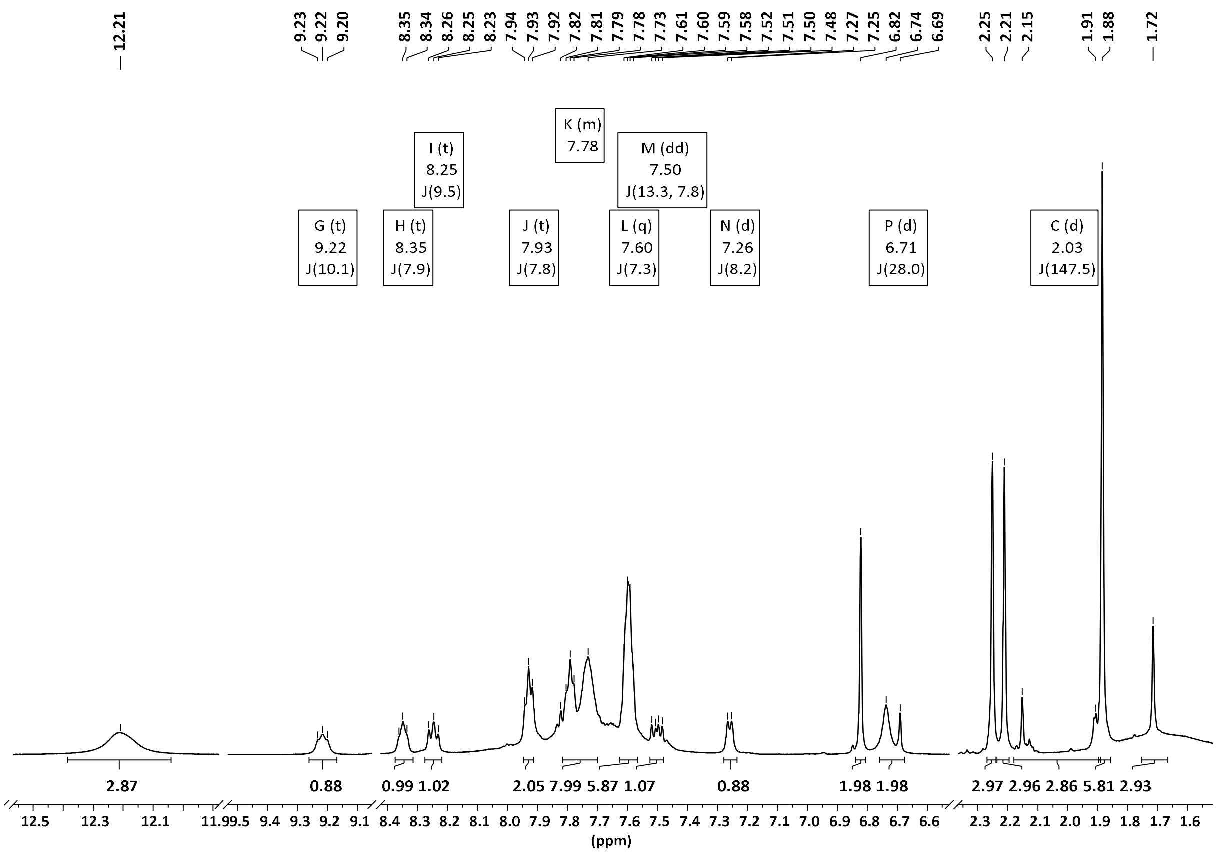

Figure S40: Detailed ^{1}H NMR spectrum (CD_2Cl_2, 600 MHz) of [**4Bi**]OTf.

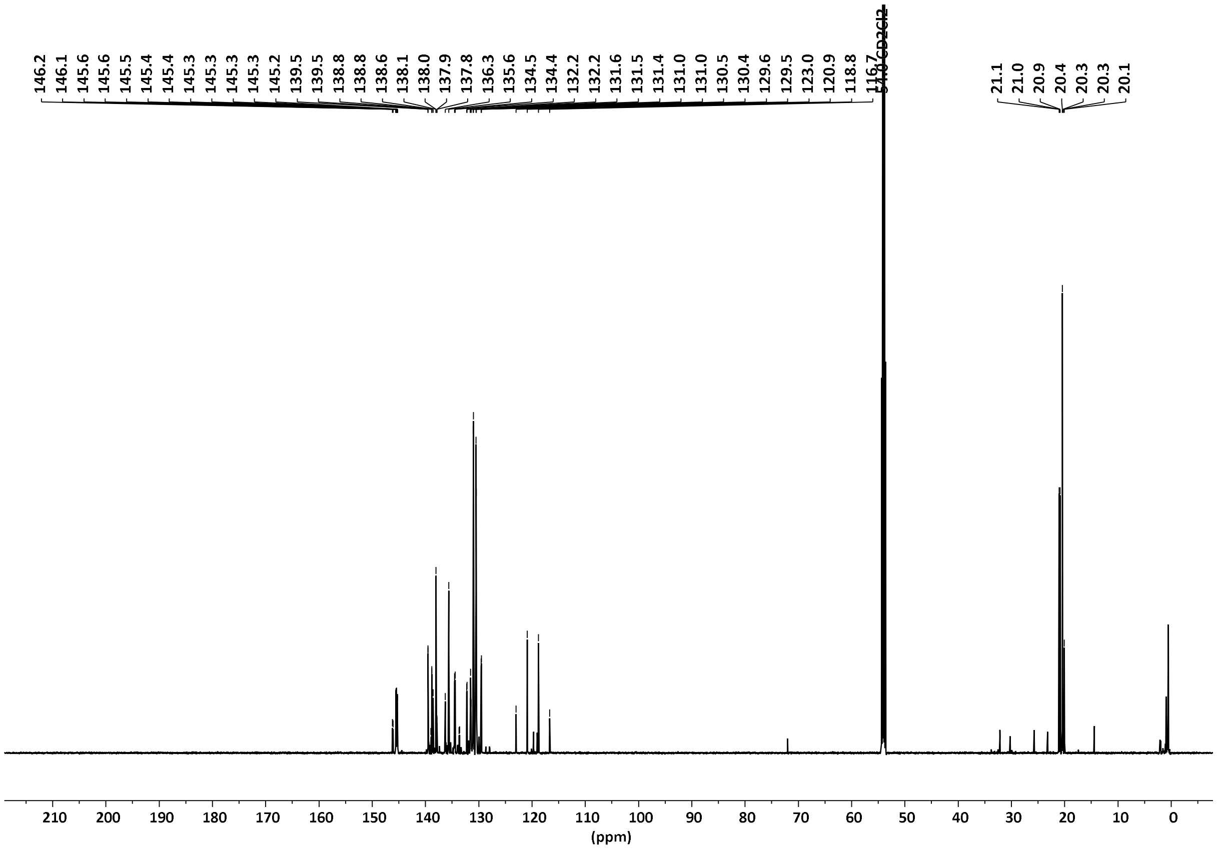

Figure S41: $^{13}C\{^{1}H\}$ NMR spectrum (CD_2Cl_2, 151 MHz) of [**4Bi**]OTf.

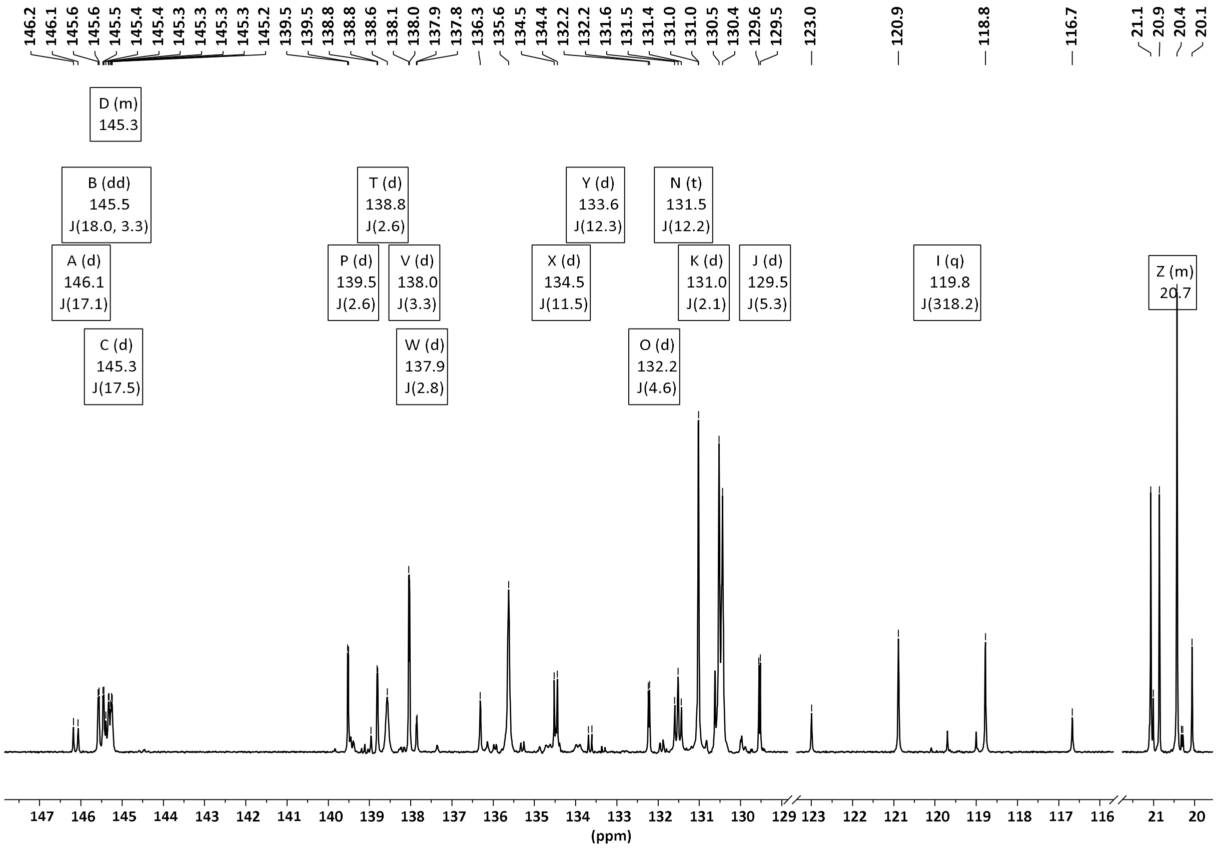

Figure S42: Detailed $^{13}C\{^1H\}$ NMR spectrum (CD_2Cl_2, 151 MHz) of [**4Bi**]OTf.

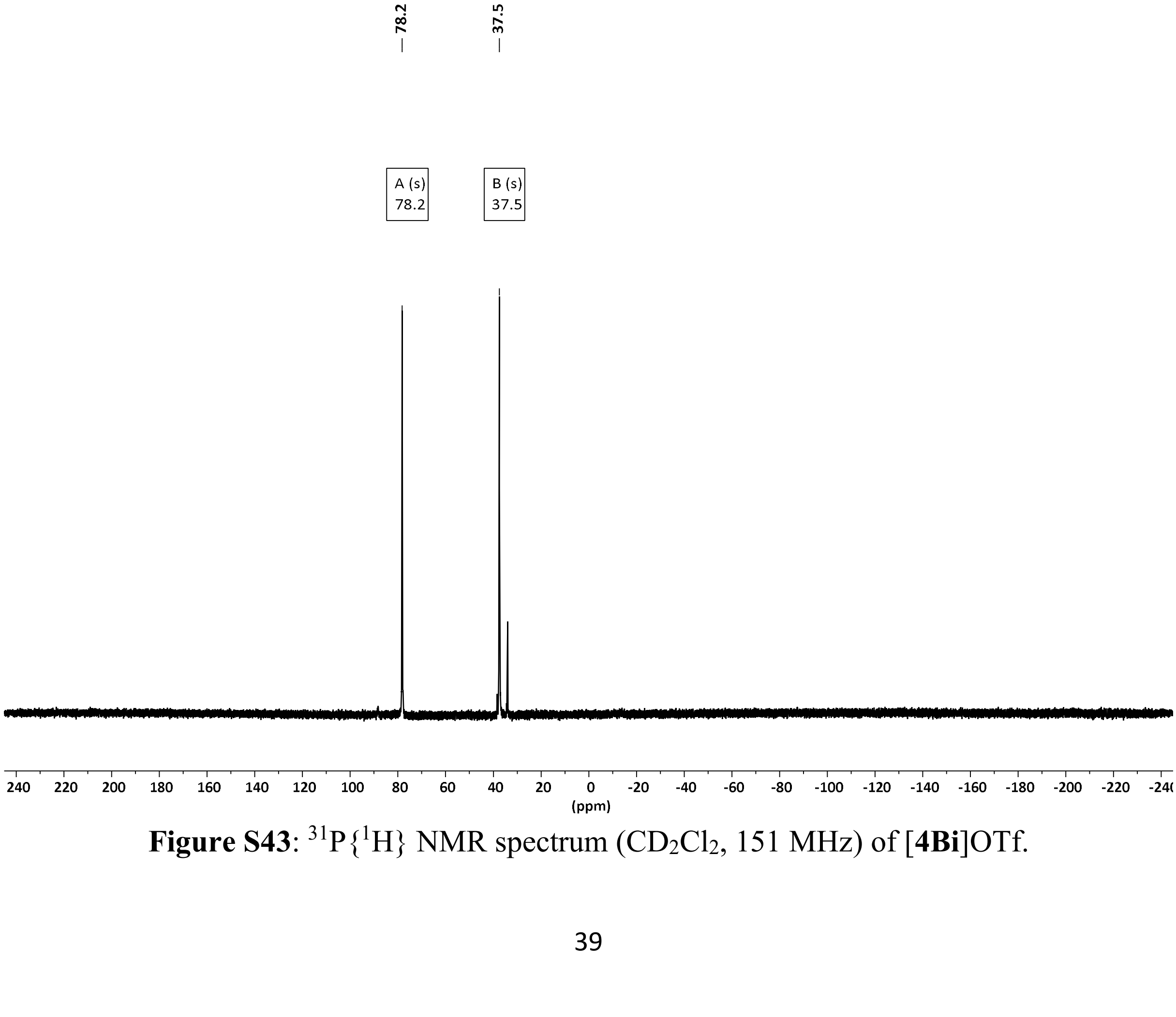

Figure S43: $^{31}P\{^1H\}$ NMR spectrum (CD_2Cl_2, 151 MHz) of [**4Bi**]OTf.

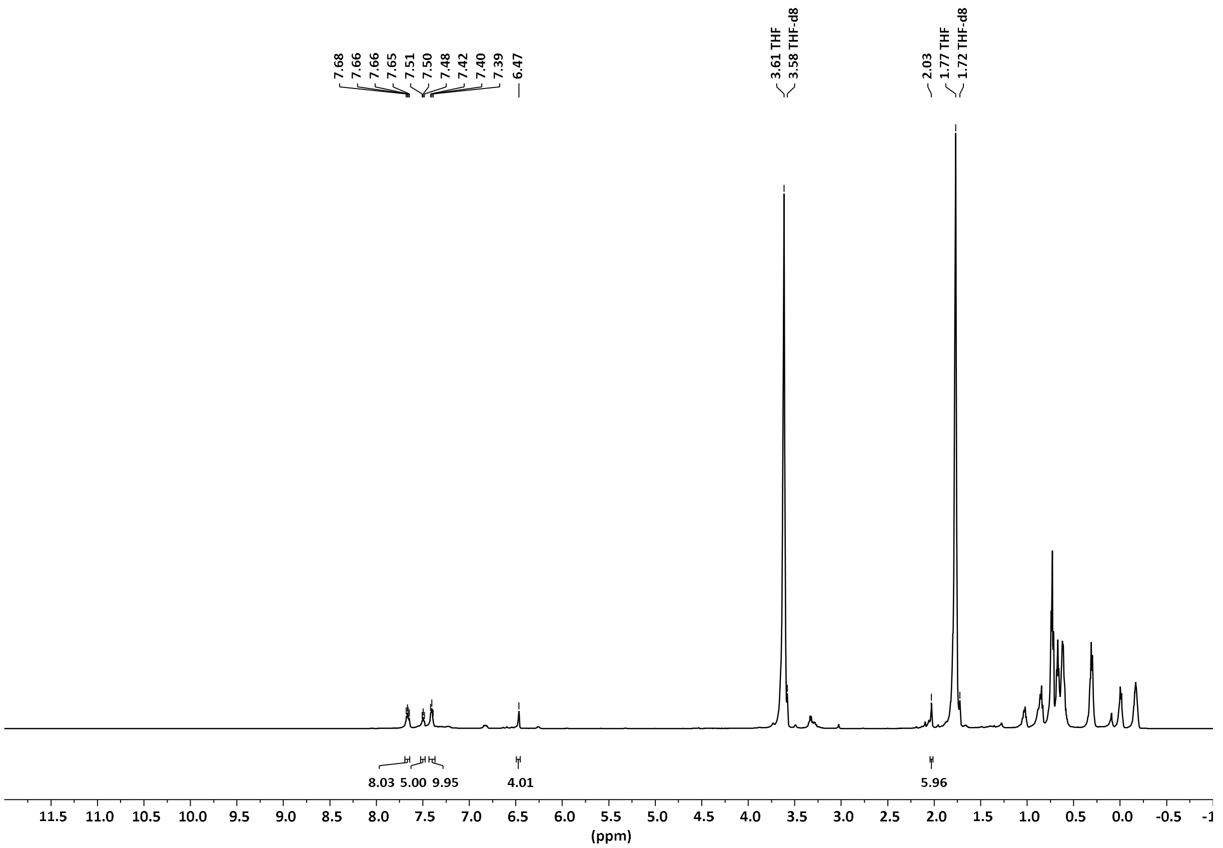

Figure S44: ^{1}H NMR spectrum (THF-d_8, 600 MHz) of **5As**.

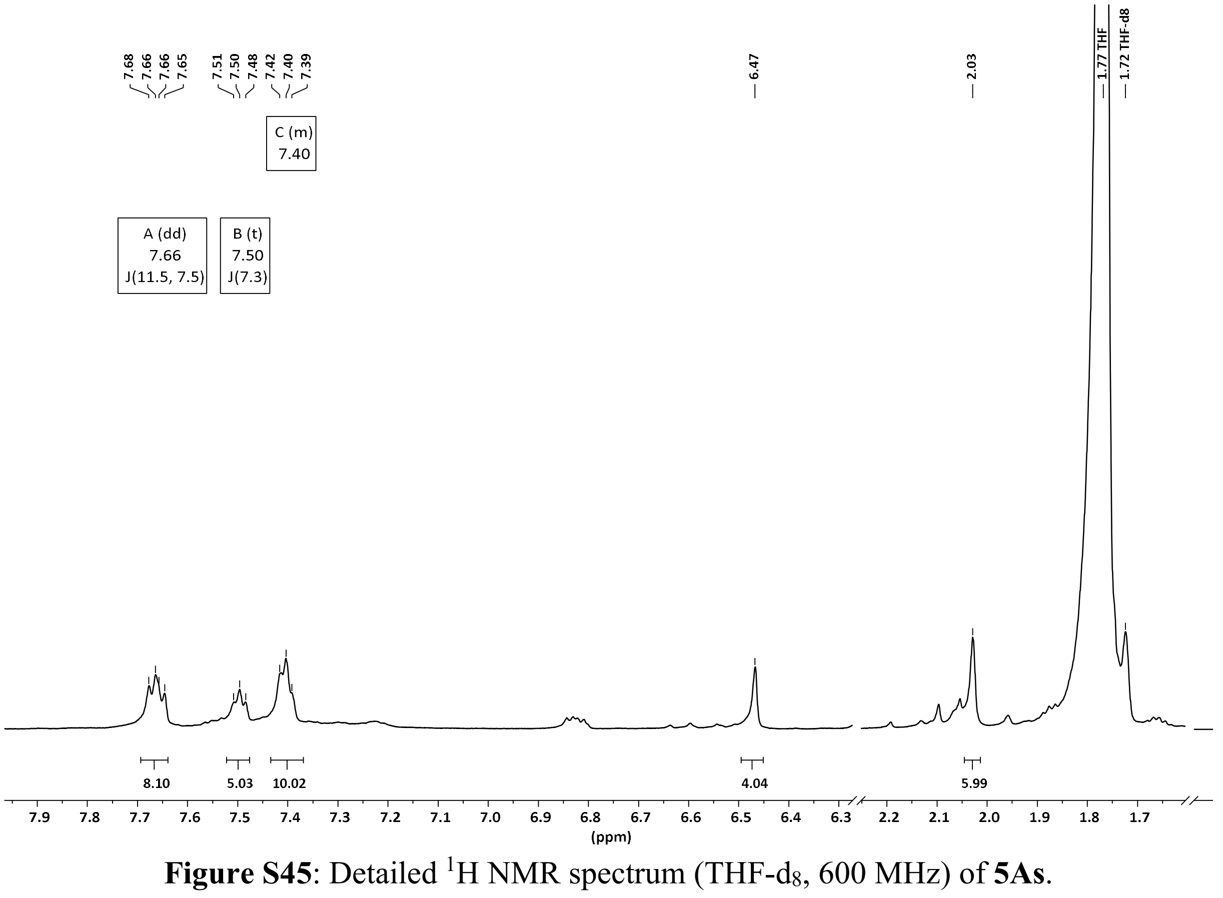

Figure S45: Detailed ^{1}H NMR spectrum (THF-d_8, 600 MHz) of **5As**.

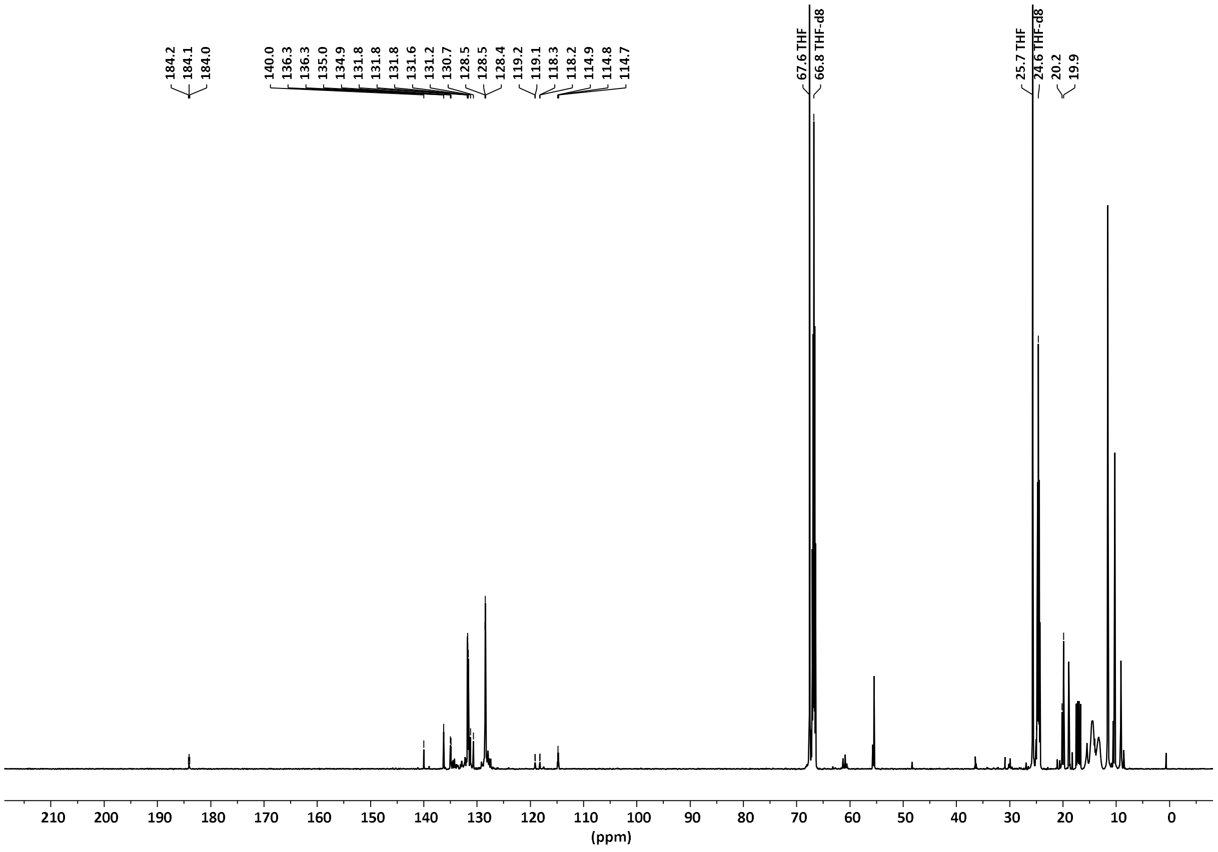

Figure S46: $^{13}C\{^{1}H\}$ NMR spectrum (THF-d$_8$, 151 MHz) of **5As**.

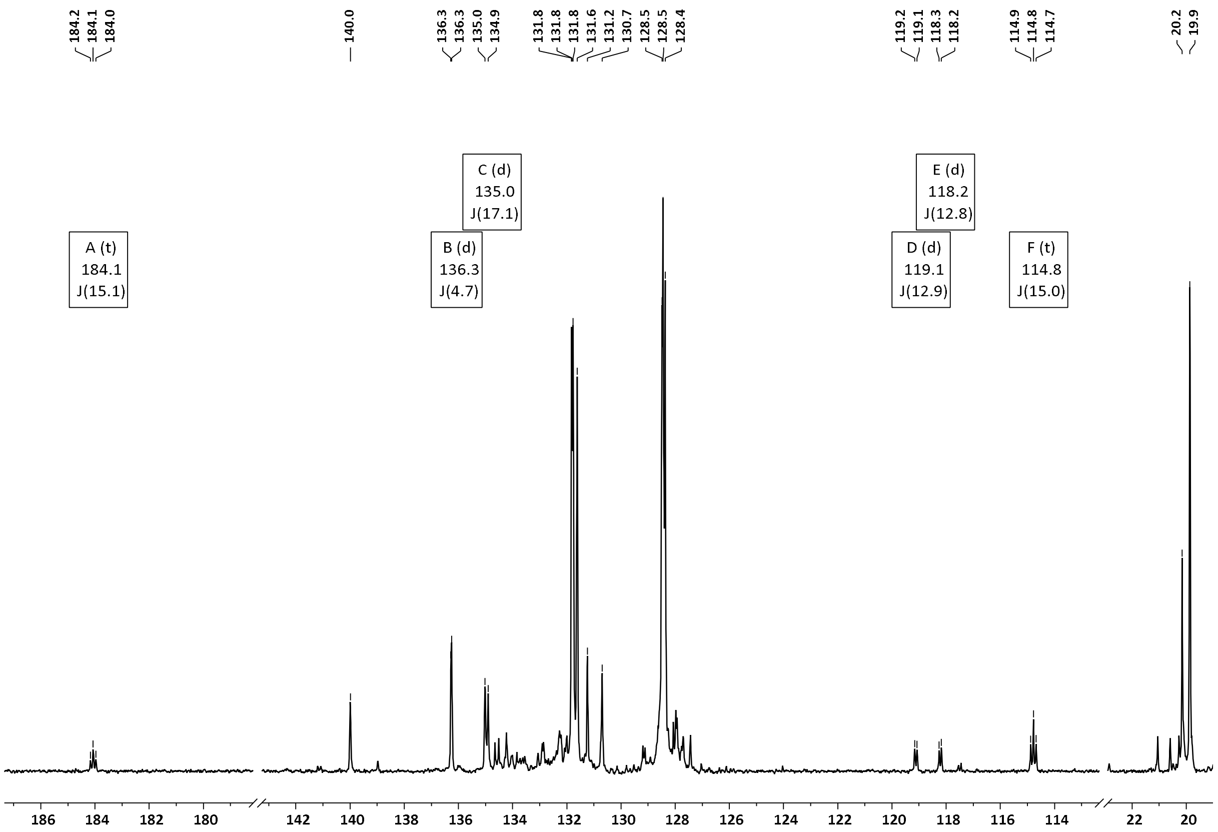

Figure S47: Detailed $^{13}C\{^{1}H\}$ NMR spectrum (THF-d$_8$, 151 MHz) of **5As**.

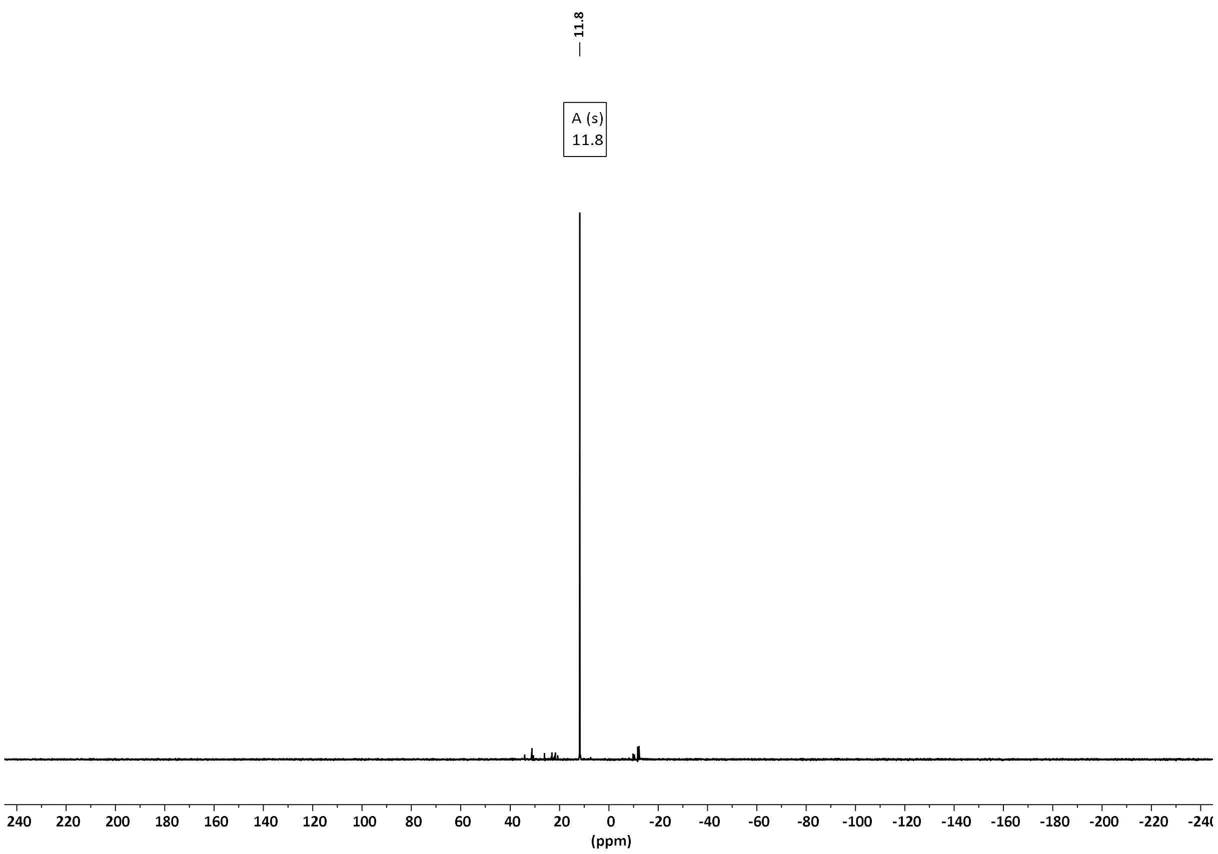

Figure S48: $^{31}P\{^{1}H\}$ NMR spectrum (THF-d$_8$,151 MHz) of **5As**.

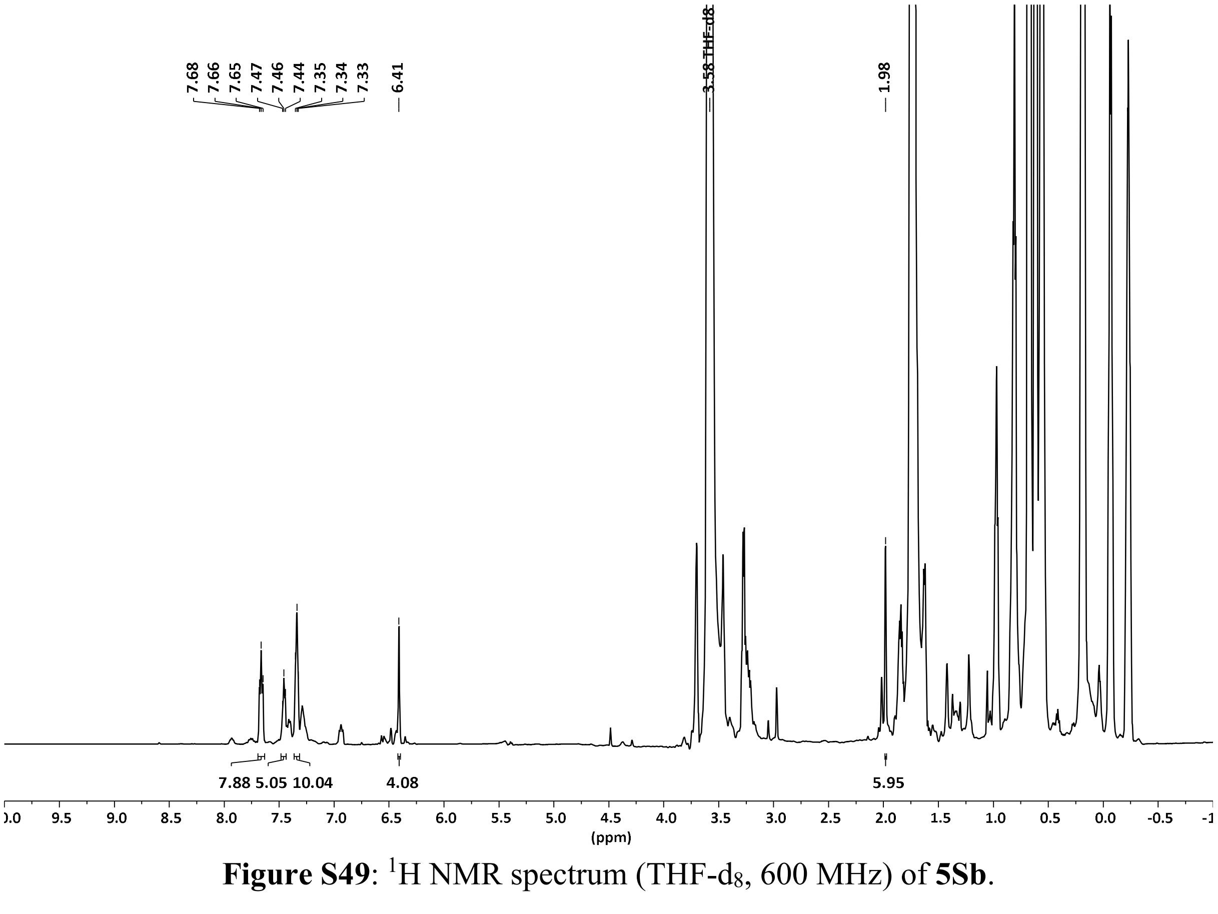

Figure S49: ^{1}H NMR spectrum (THF-d$_8$, 600 MHz) of **5Sb**.

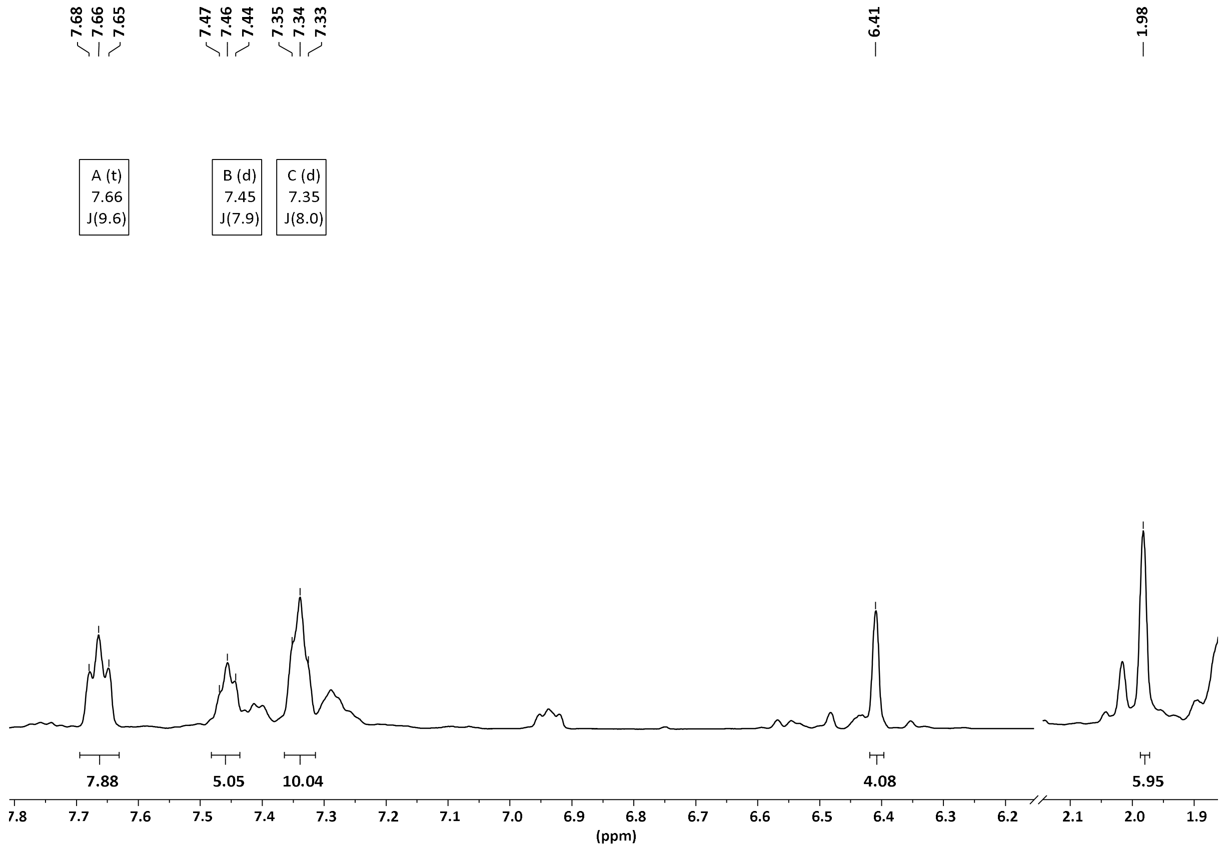

Figure S50: Detailed ^{1}H NMR spectrum (THF-d$_8$, 600 MHz) of **5Sb**.

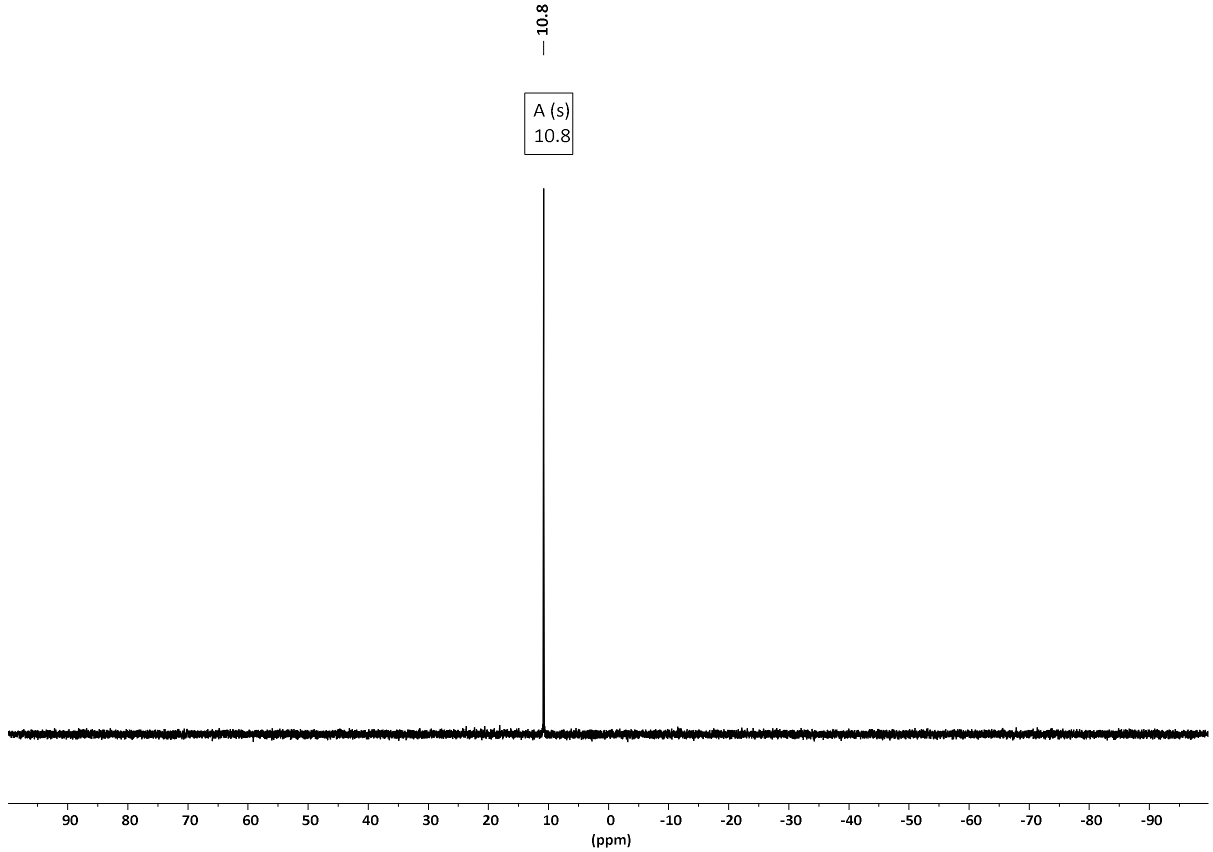

Figure S51: ^{31}P{^{1}H} NMR spectrum (THF-d$_8$,151 MHz) of **5Sb**.

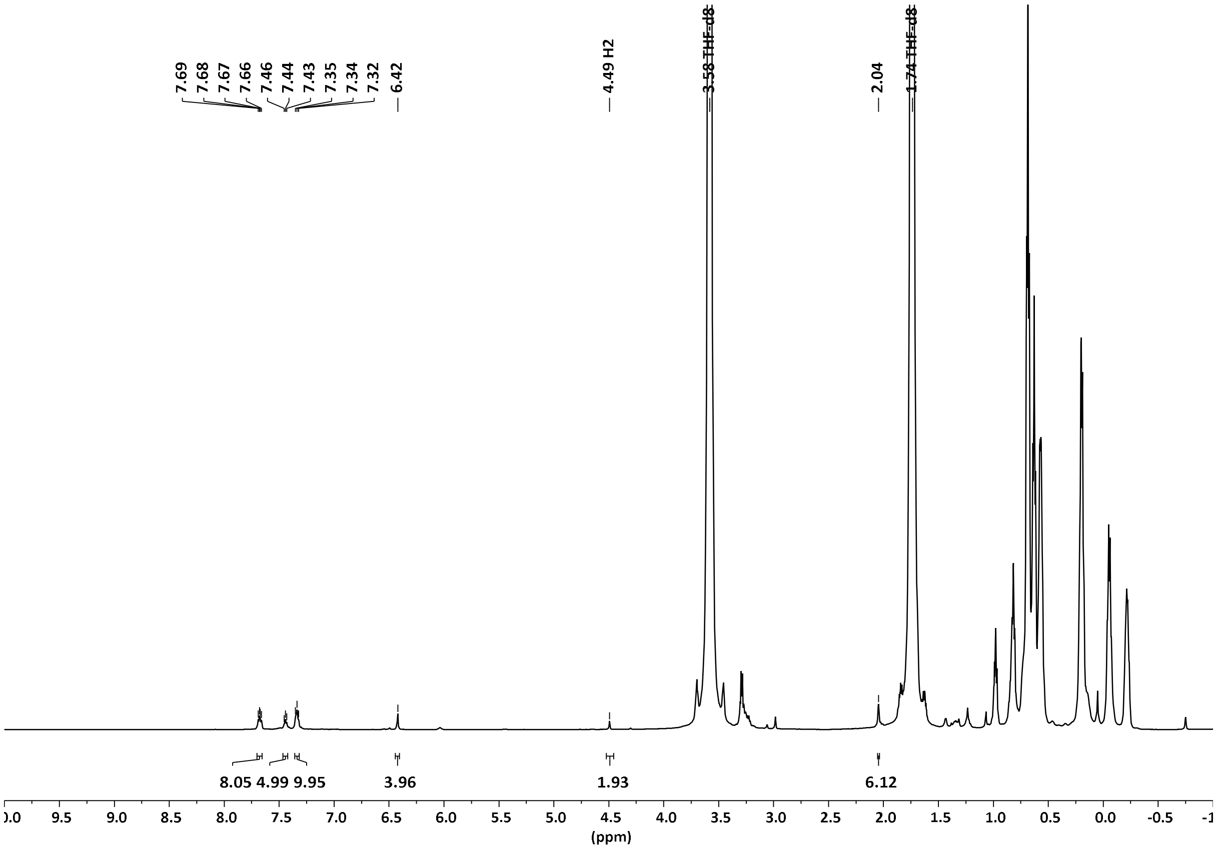

Figure S52: ^{1}H NMR spectrum (THF-d_8, 600 MHz) of **5Bi**.

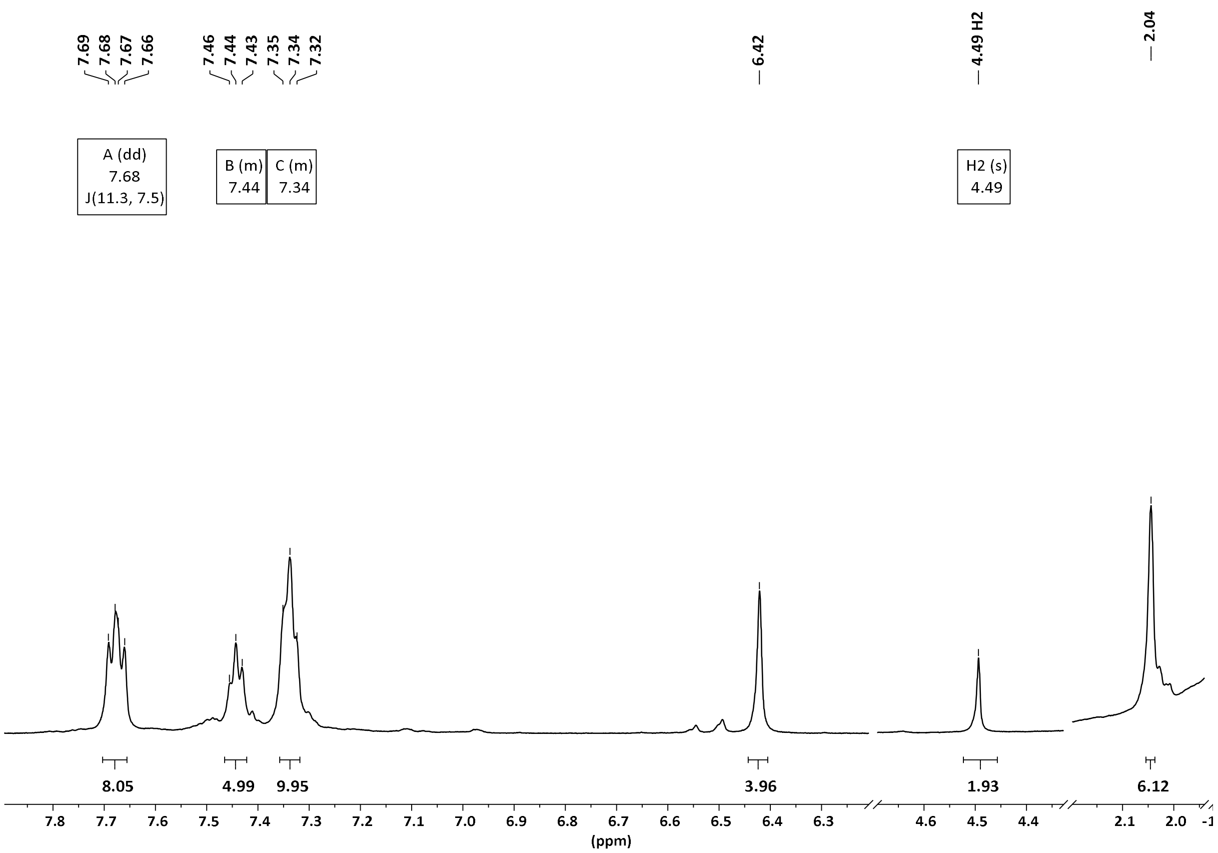

Figure S53: Detailed ^{1}H NMR spectrum (THF-d_8, 600 MHz) of **5Bi**.

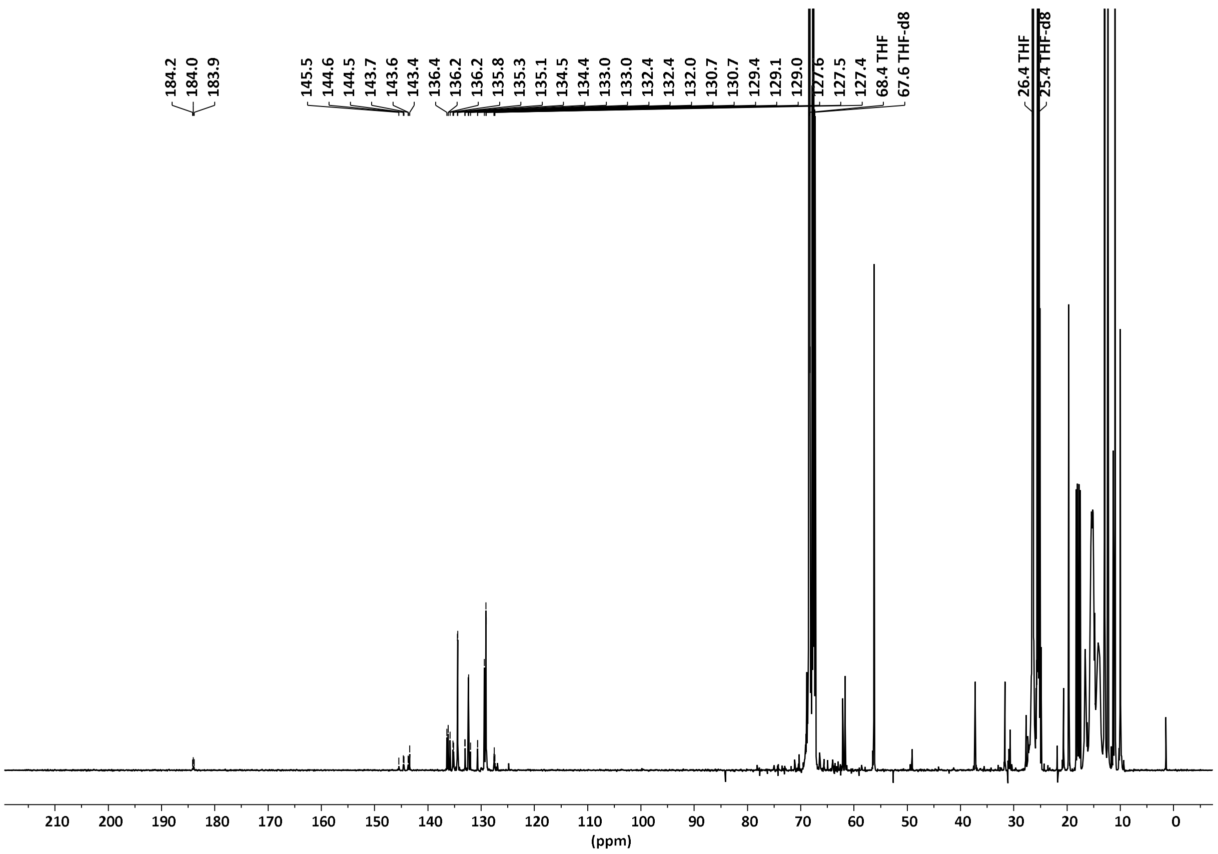

Figure S54: $^{13}C\{^{1}H\}$ NMR spectrum (THF-d$_8$, 151 MHz) of **5Bi**.

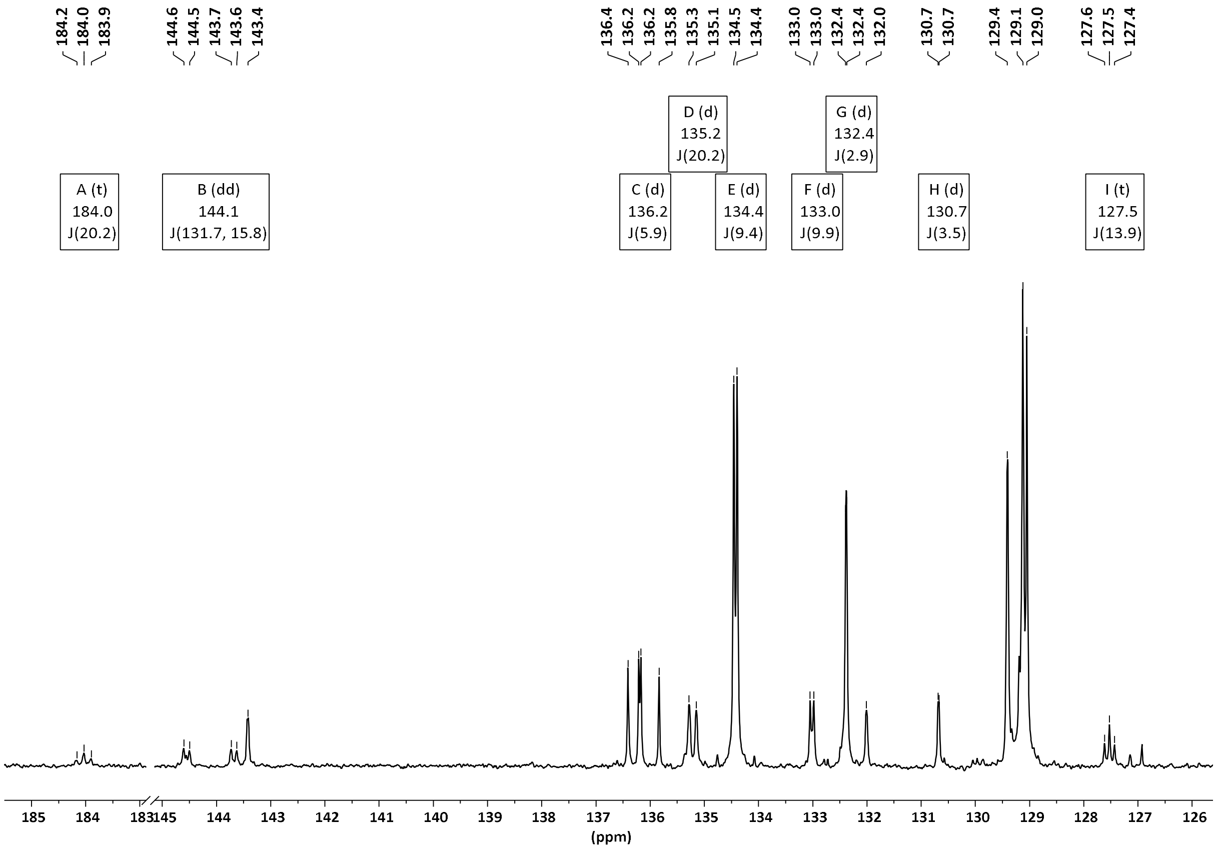

Figure S55: Detailed $^{13}C\{^{1}H\}$ NMR spectrum (THF-d$_8$, 151 MHz) of **5Bi**.

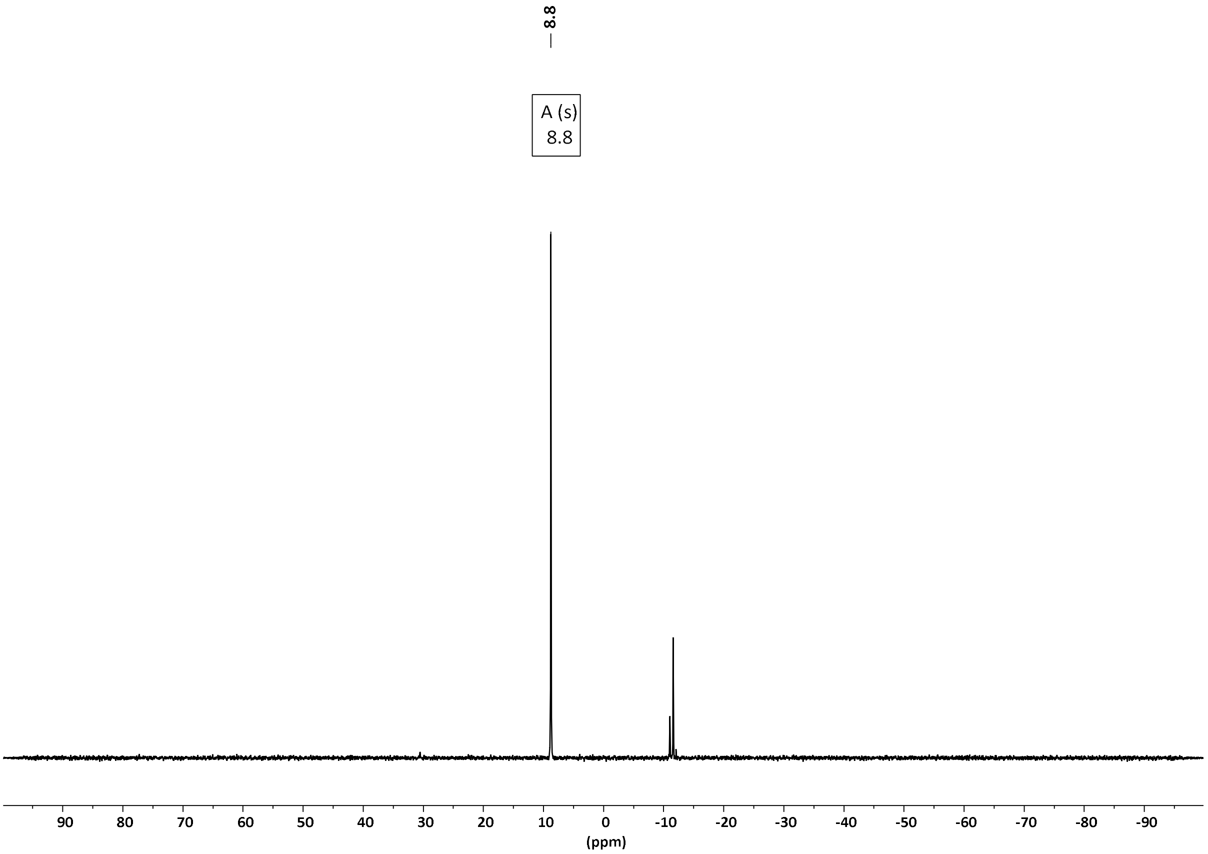

Figure S56: $^{31}P\{^1H\}$ NMR spectrum (THF-d_8, 151 MHz) of **5Bi**.

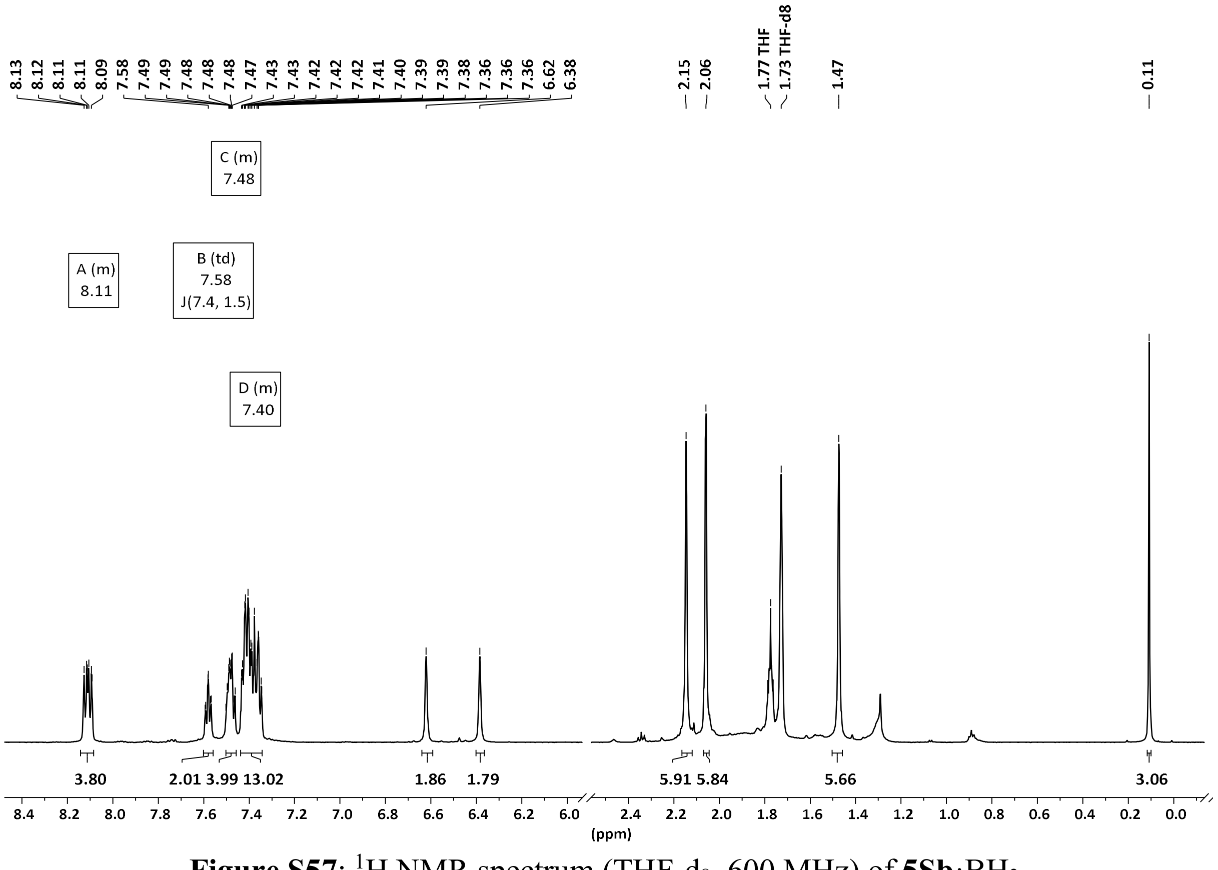

Figure S57: ^1H NMR spectrum (THF-d_8, 600 MHz) of **5Sb**·BH_3.

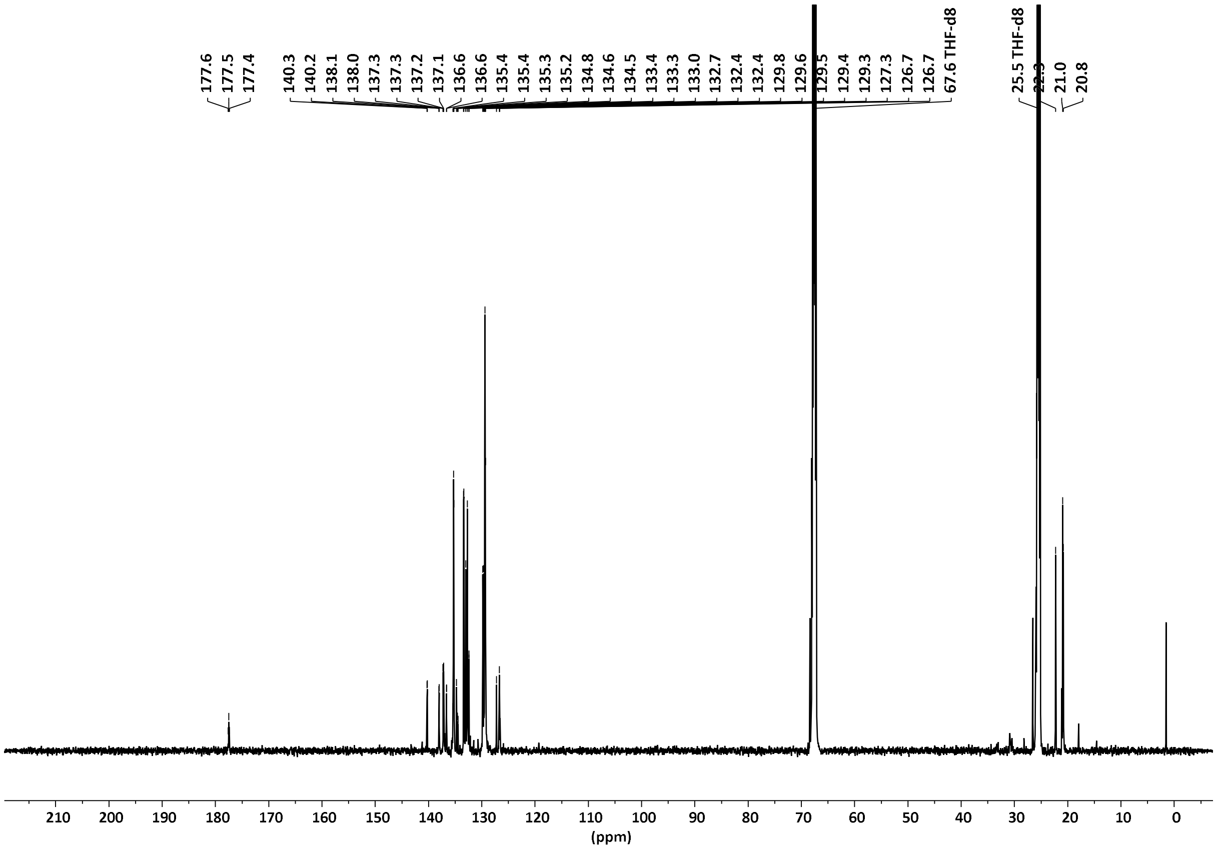

Figure S58: $^{13}C\{^{1}H\}$ NMR spectrum (THF-d_8, 151 MHz) of **5Sb**·BH_3.

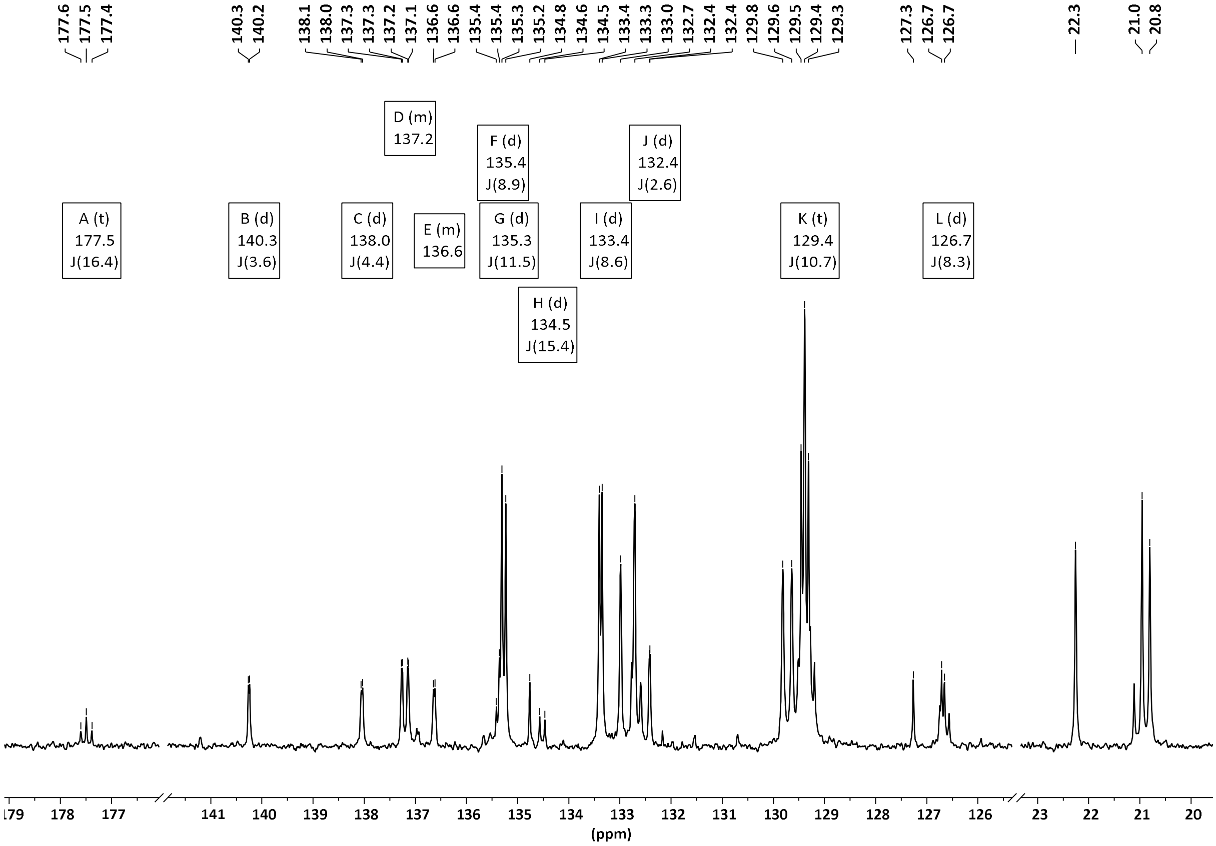

Figure S59: Detailed $^{13}C\{^{1}H\}$ NMR spectrum (THF-d_8, 151 MHz) of **5Sb**·BH_3.

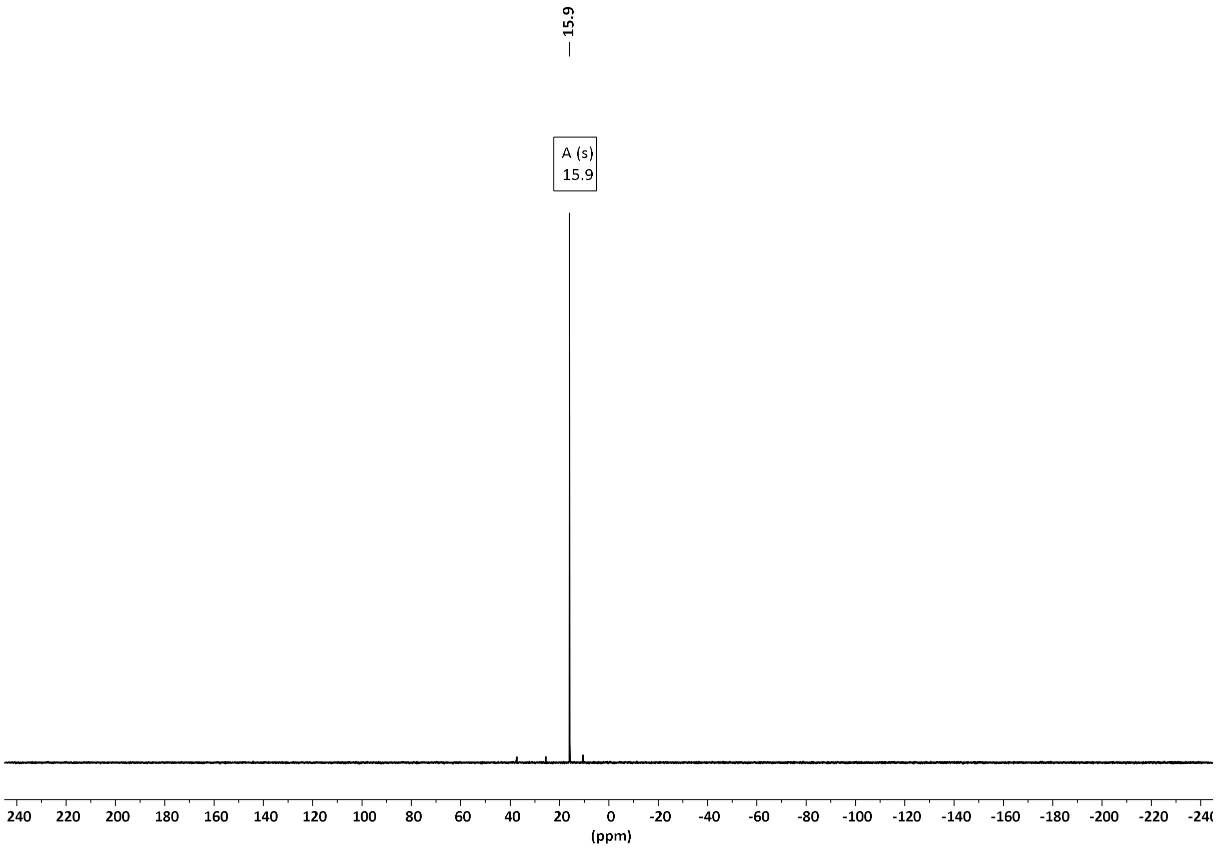

Figure S60: $^{31}P\{^1H\}$ NMR spectrum (THF-d$_8$, 151 MHz) of **5Sb**·BH$_3$.

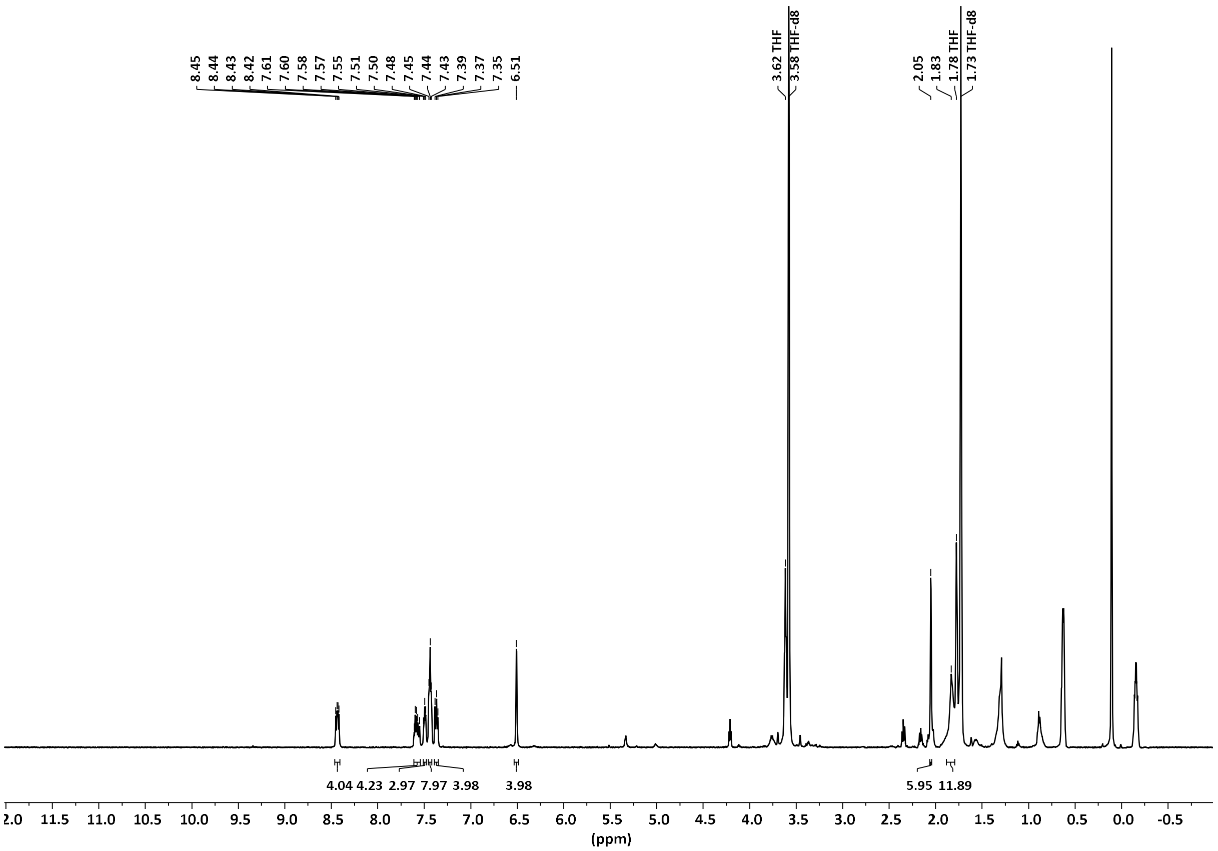

Figure S61: ^1H NMR spectrum (THF-d$_8$, 600 MHz) of **6AsS**.

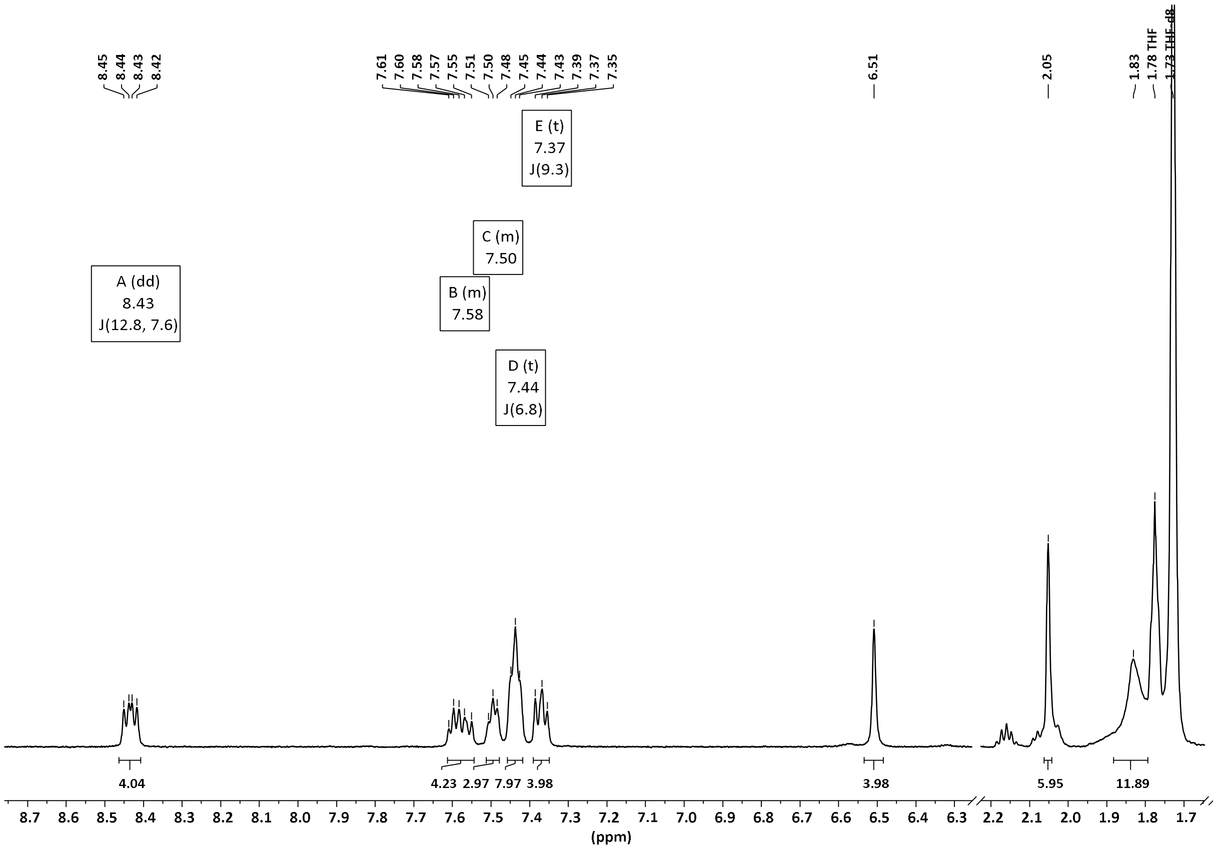

Figure S62: Detailed ^{1}H NMR spectrum (THF-d_8, 600 MHz) of **6AsS**.

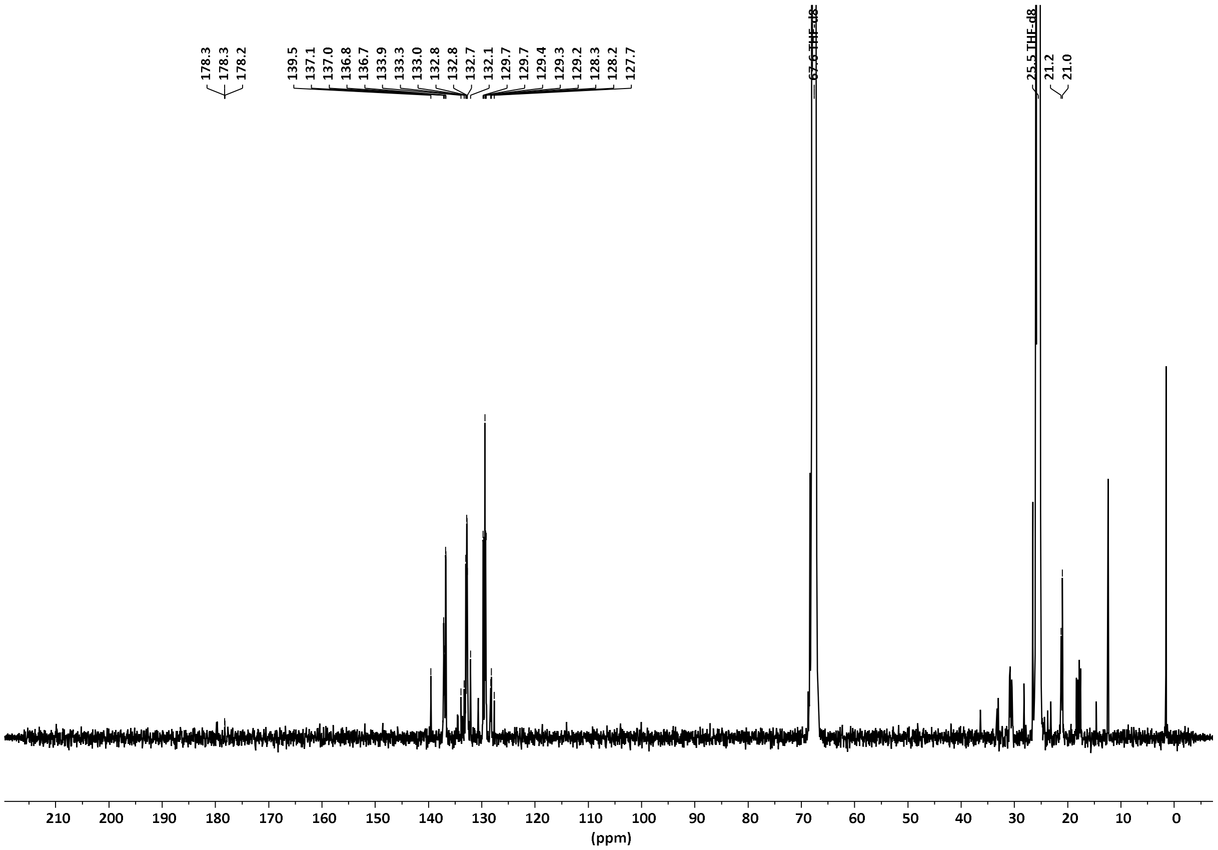

Figure S63: $^{13}C\{^{1}H\}$ NMR spectrum (THF-d_8, 151 MHz) of **6AsS**.

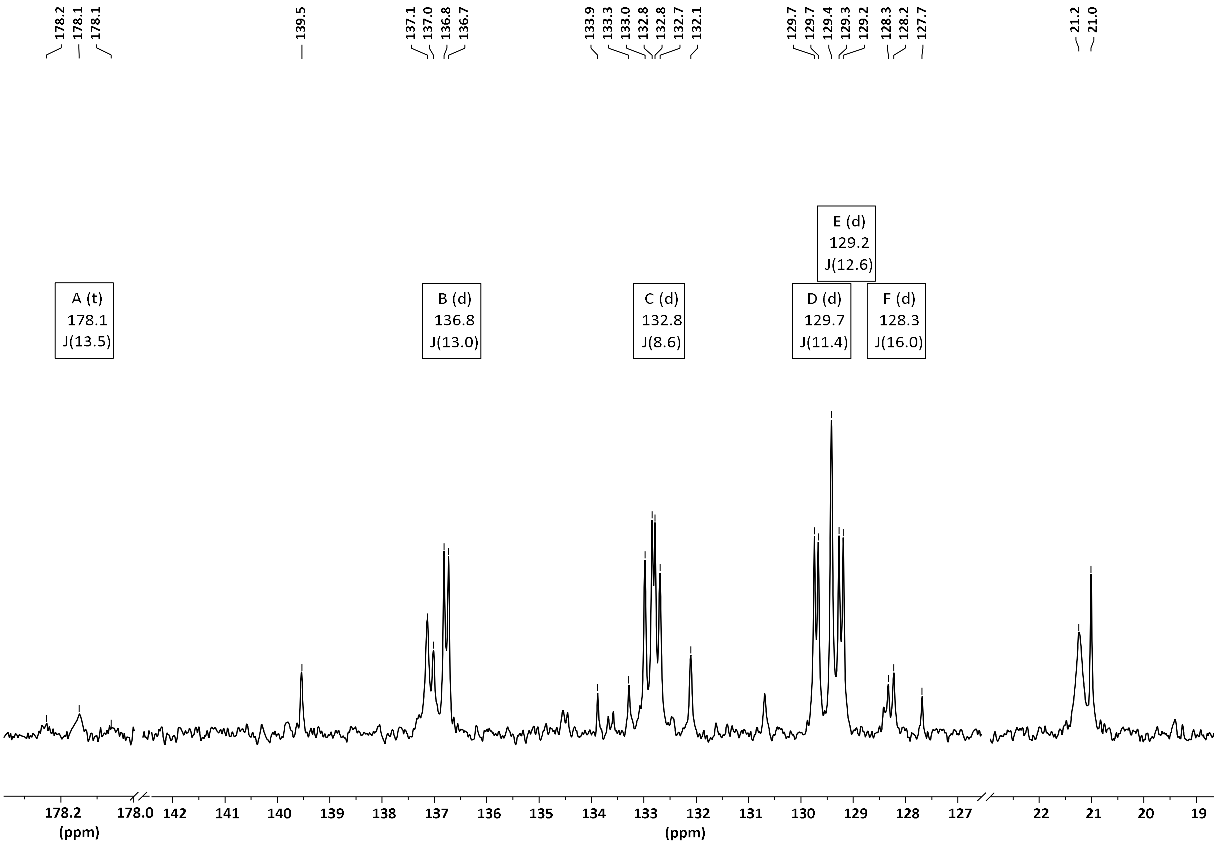

Figure S64: Detailed $^{13}C\{^1H\}$ NMR spectrum (THF-d$_8$, 151 MHz) of **6AsS**.

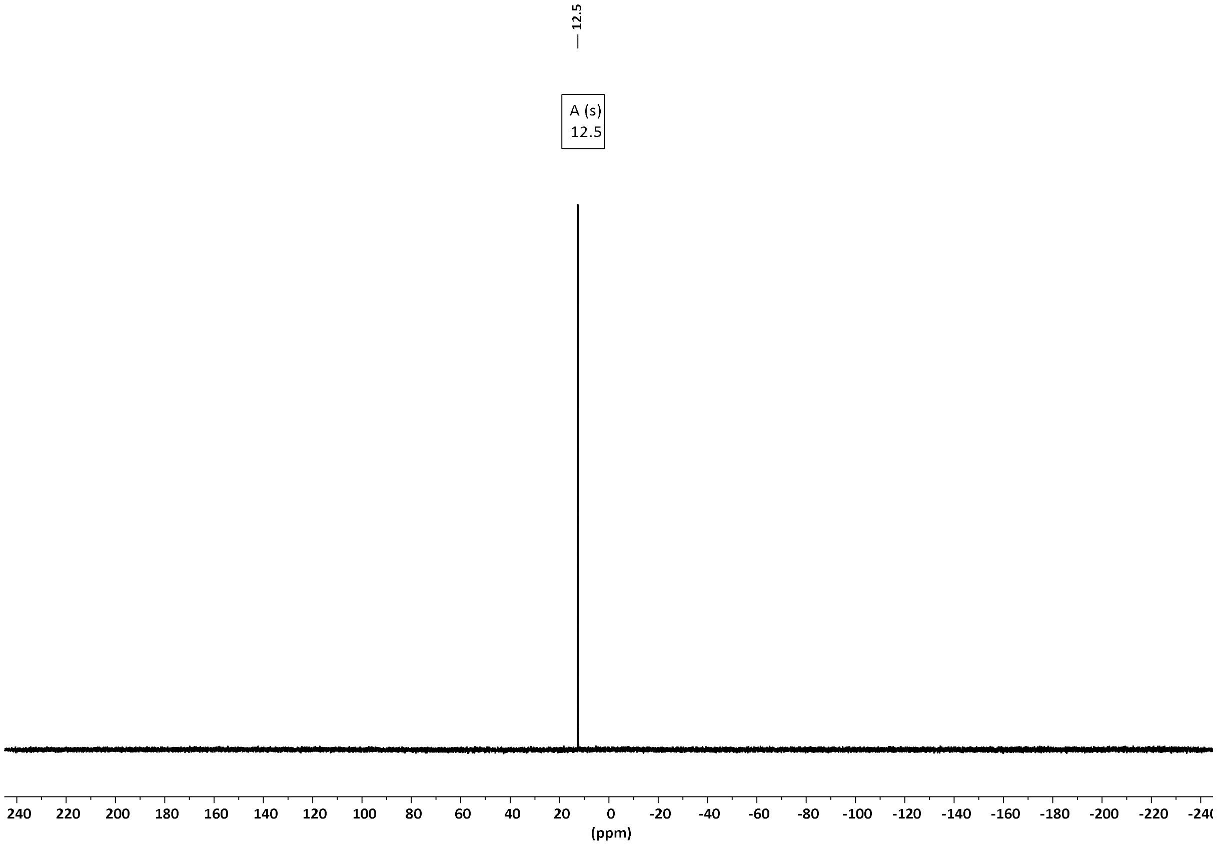

Figure S65: $^{31}P\{^1H\}$ NMR spectrum (THF-d$_8$, 151 MHz) of **6AsS**.

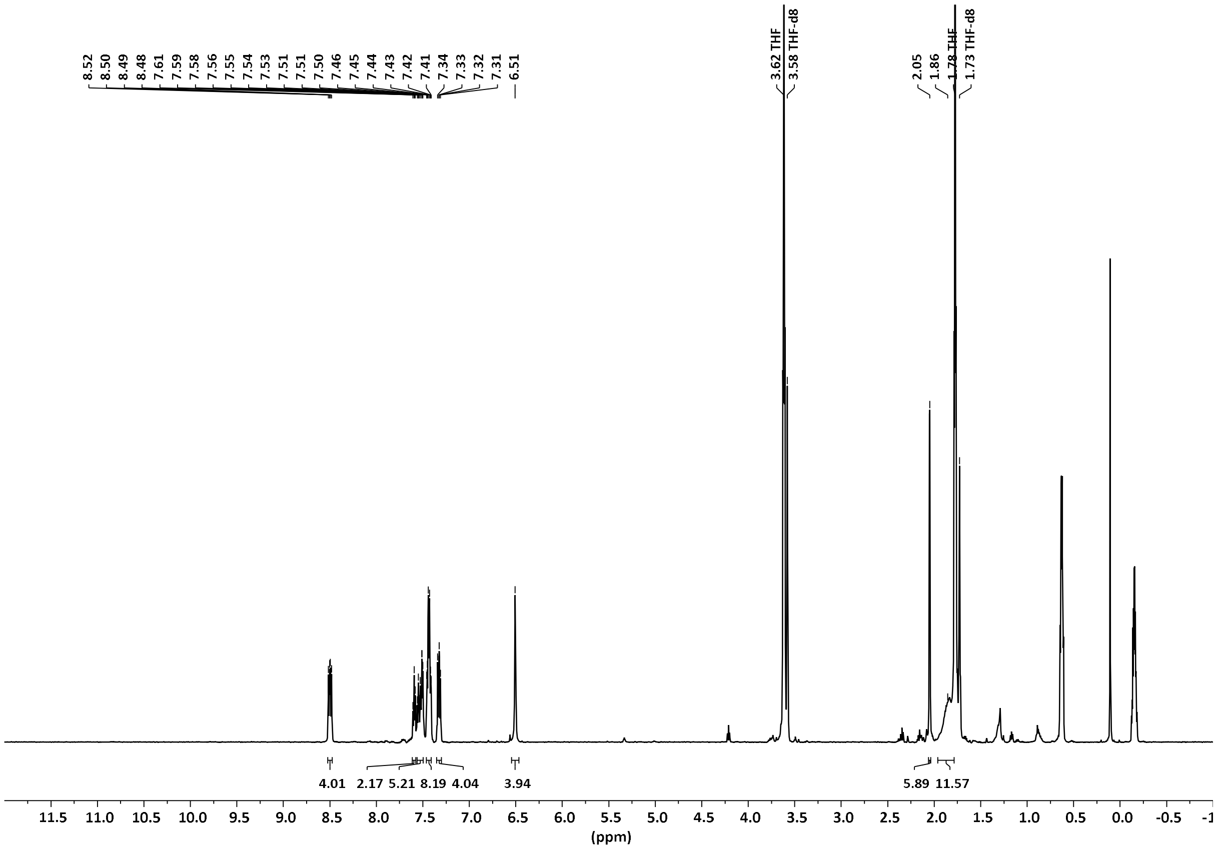

Figure S66: ^{1}H NMR spectrum (THF-d$_8$, 600 MHz) of **6AsSe**.

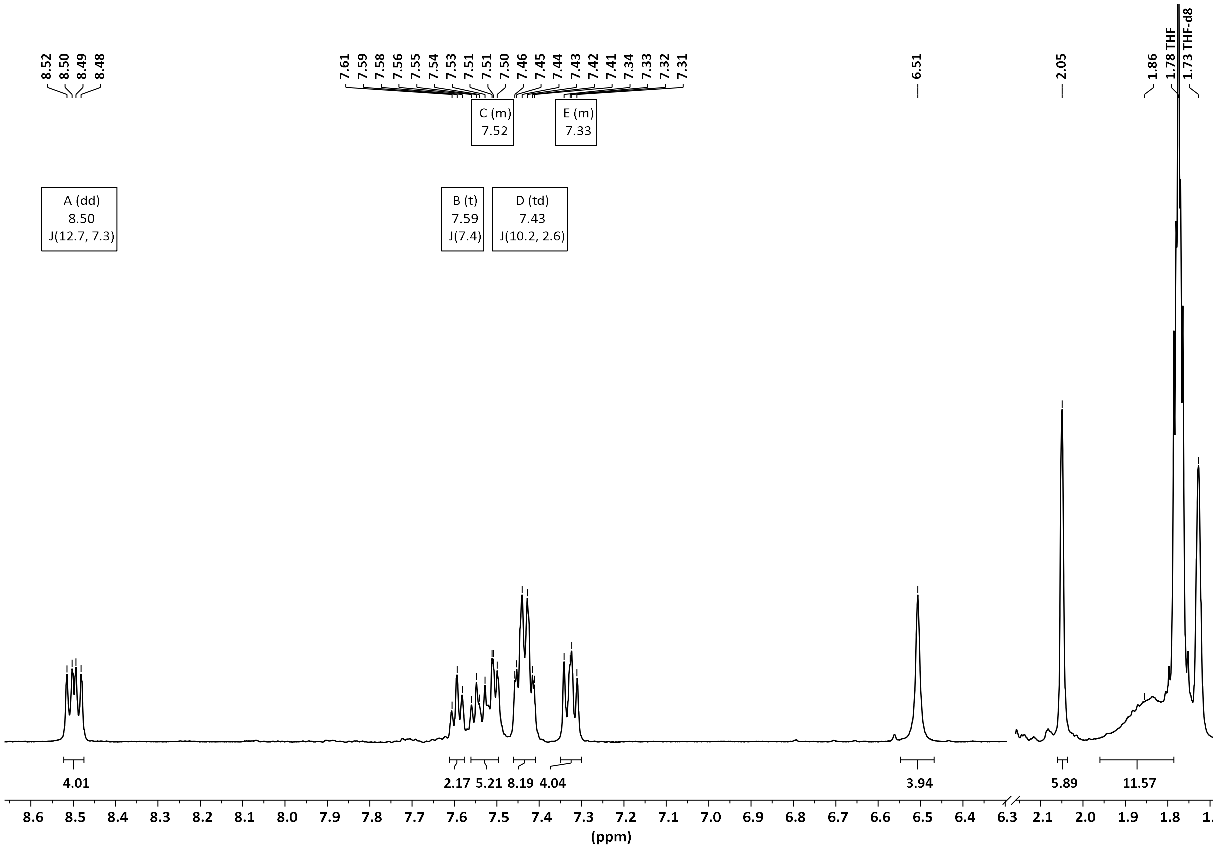

Figure S67: Detailed ^{1}H NMR spectrum (THF-d$_8$, 600 MHz) of **6AsSe**.

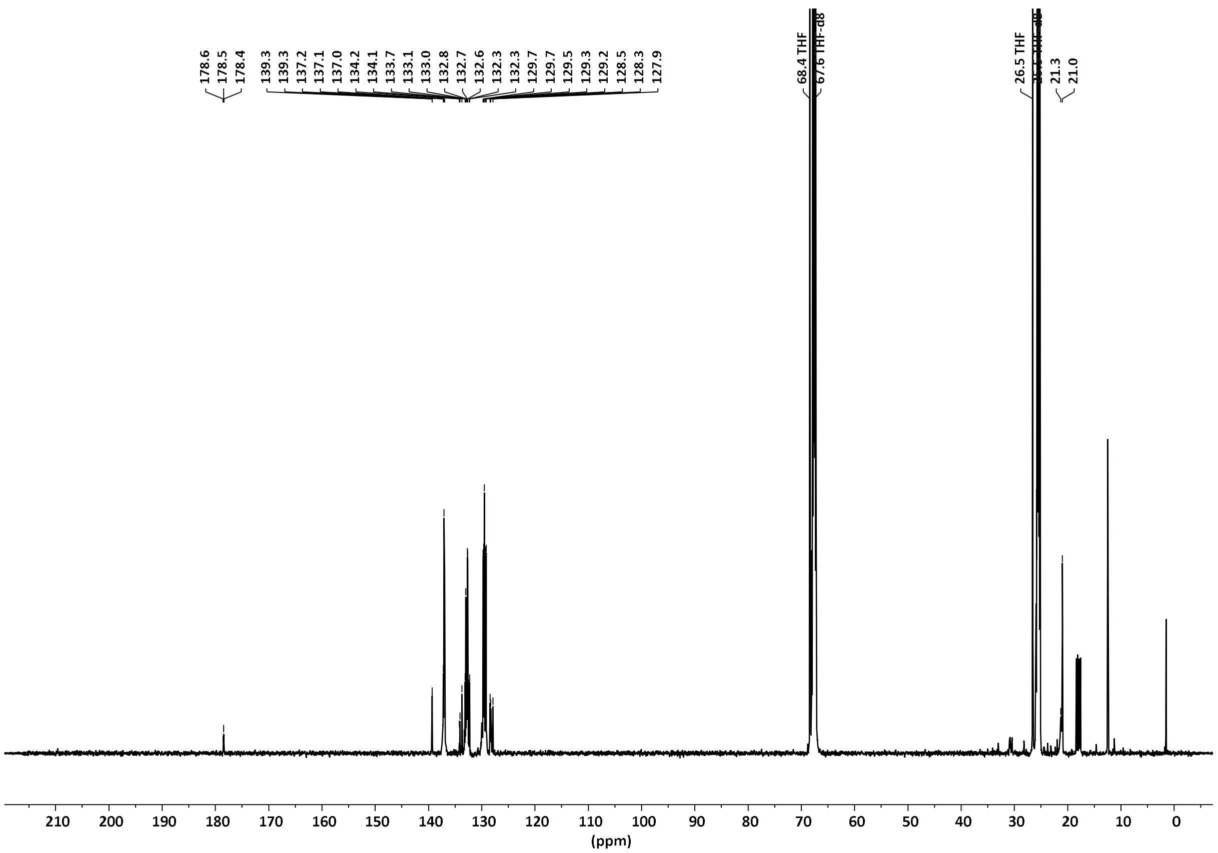

Figure S68: $^{13}C\{^{1}H\}$ NMR spectrum (THF-d$_8$, 151 MHz) of **6AsSe**.

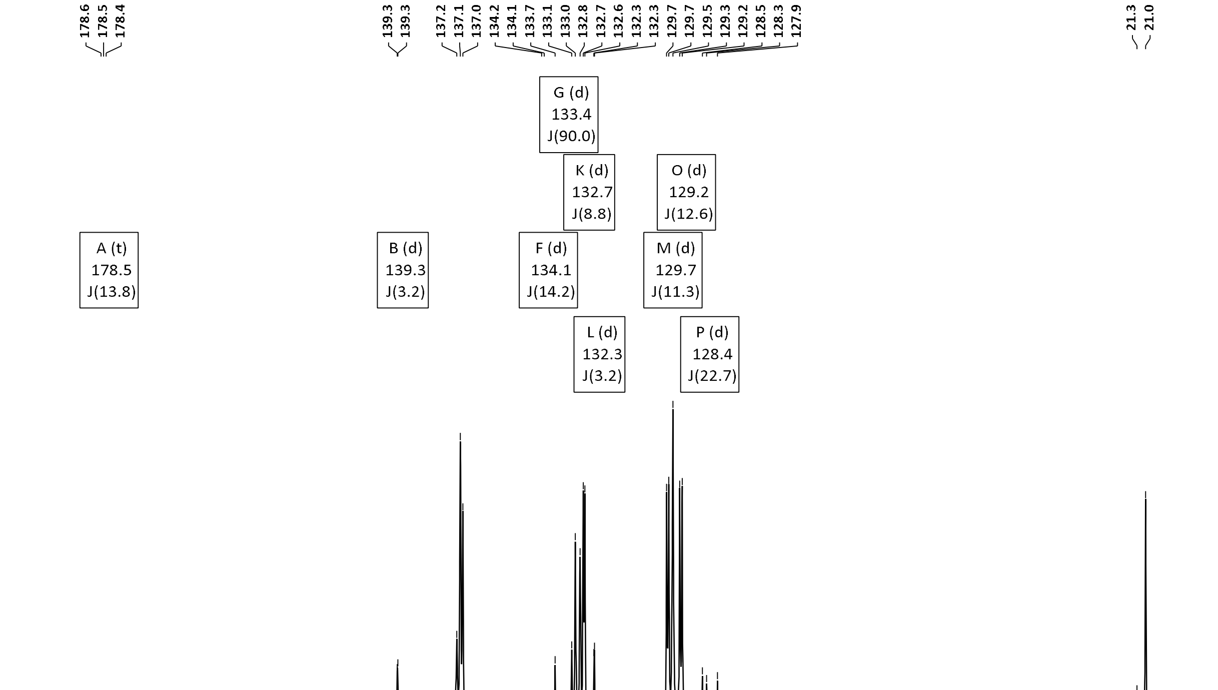

Figure S69: Detailed $^{13}C\{^{1}H\}$ NMR spectrum (THF-d$_8$, 151 MHz) of **6AsSe**.

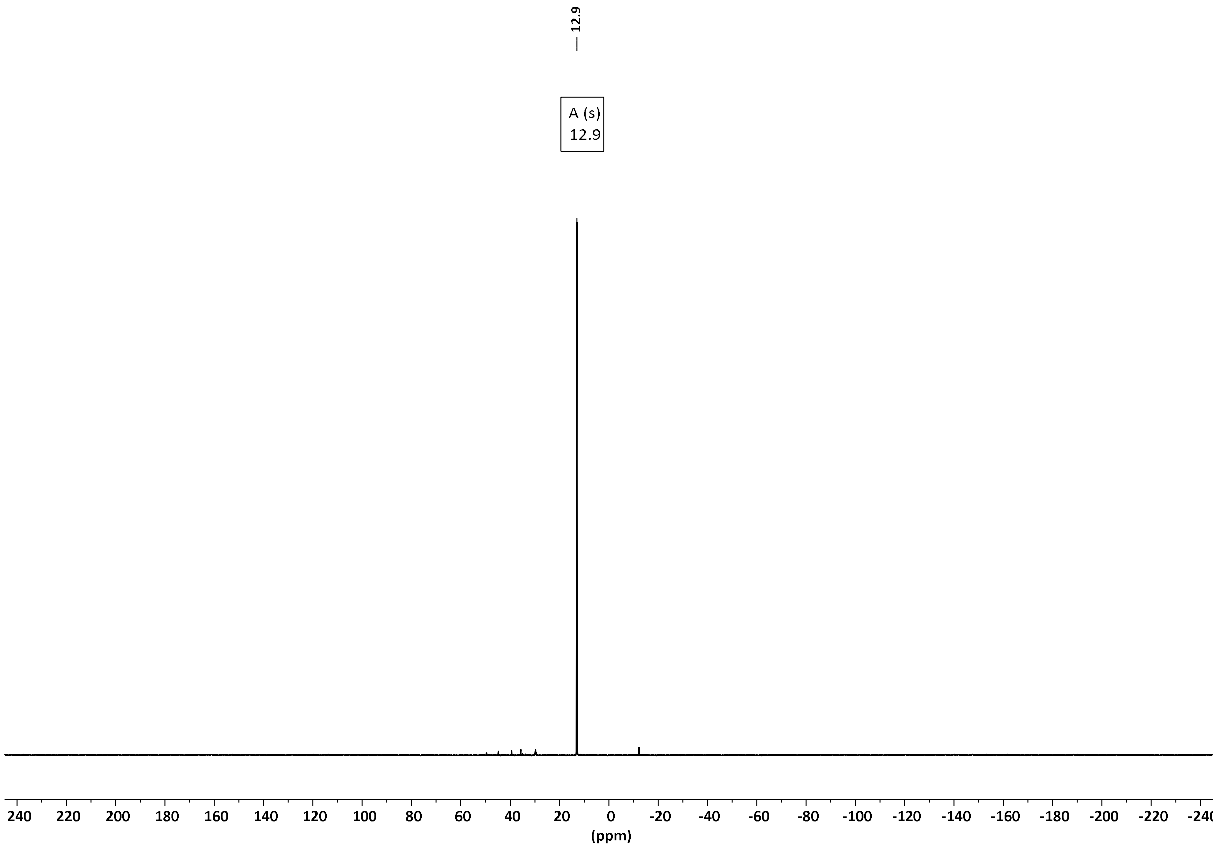

Figure S70: $^{31}P\{^{1}H\}$ NMR spectrum (THF-d_8, 151 MHz) of **6AsSe**.

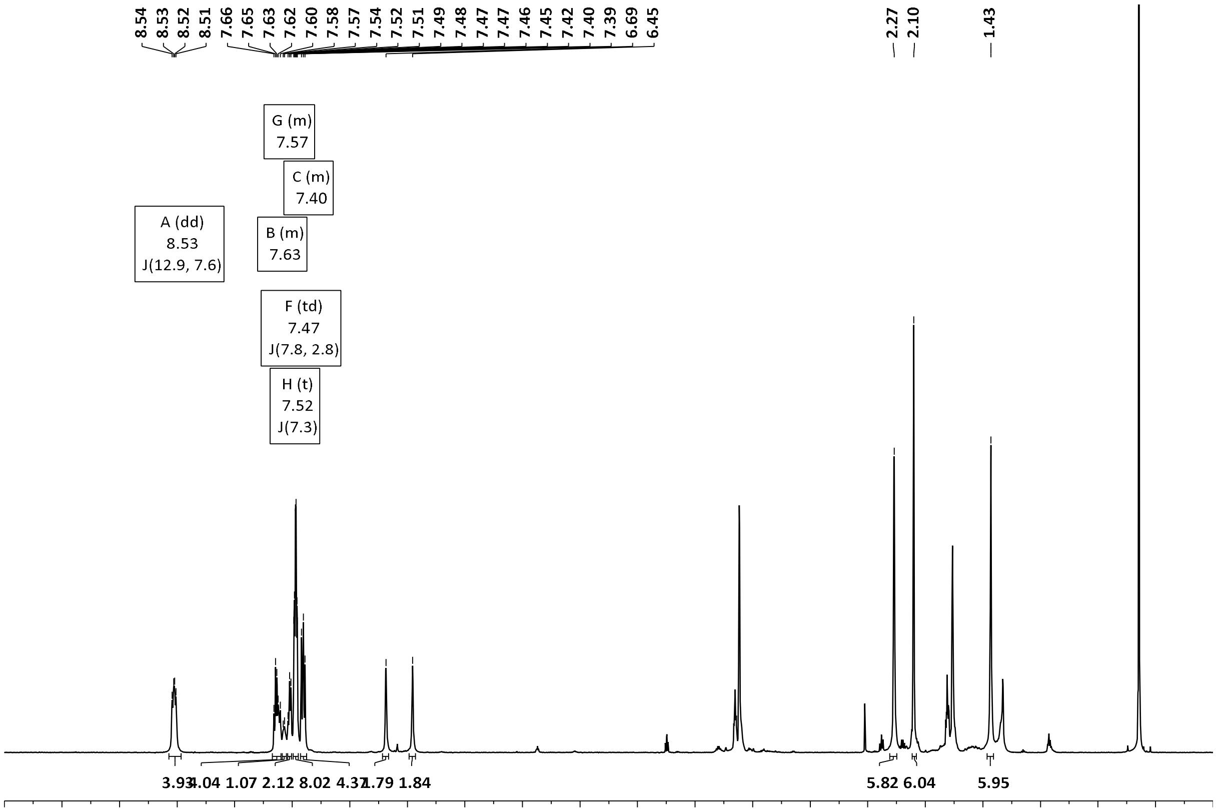

Figure S71: ^{1}H NMR spectrum (THF-d_8, 600 MHz) of **6SbS**.

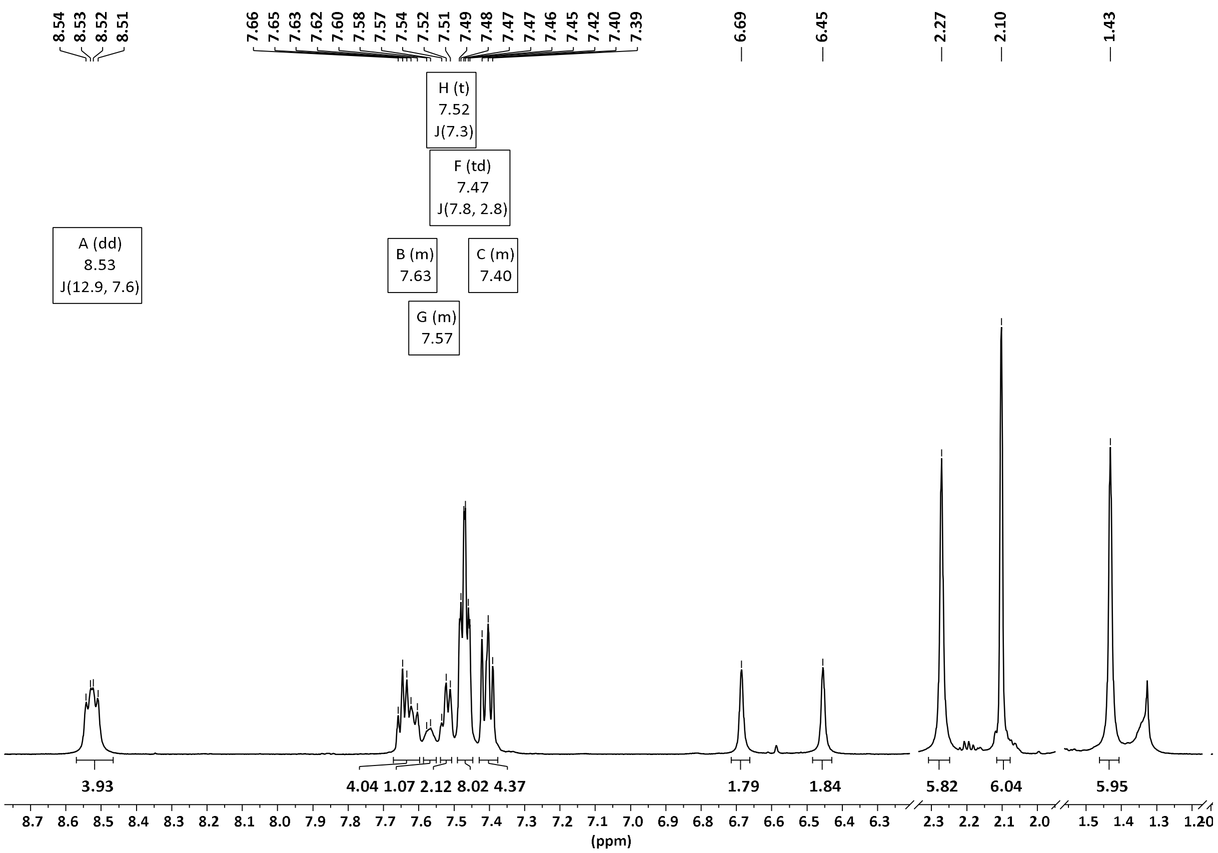

Figure S72: Detailed ^{1}H NMR spectrum (THF-d_8, 600 MHz) of **6SbS**.

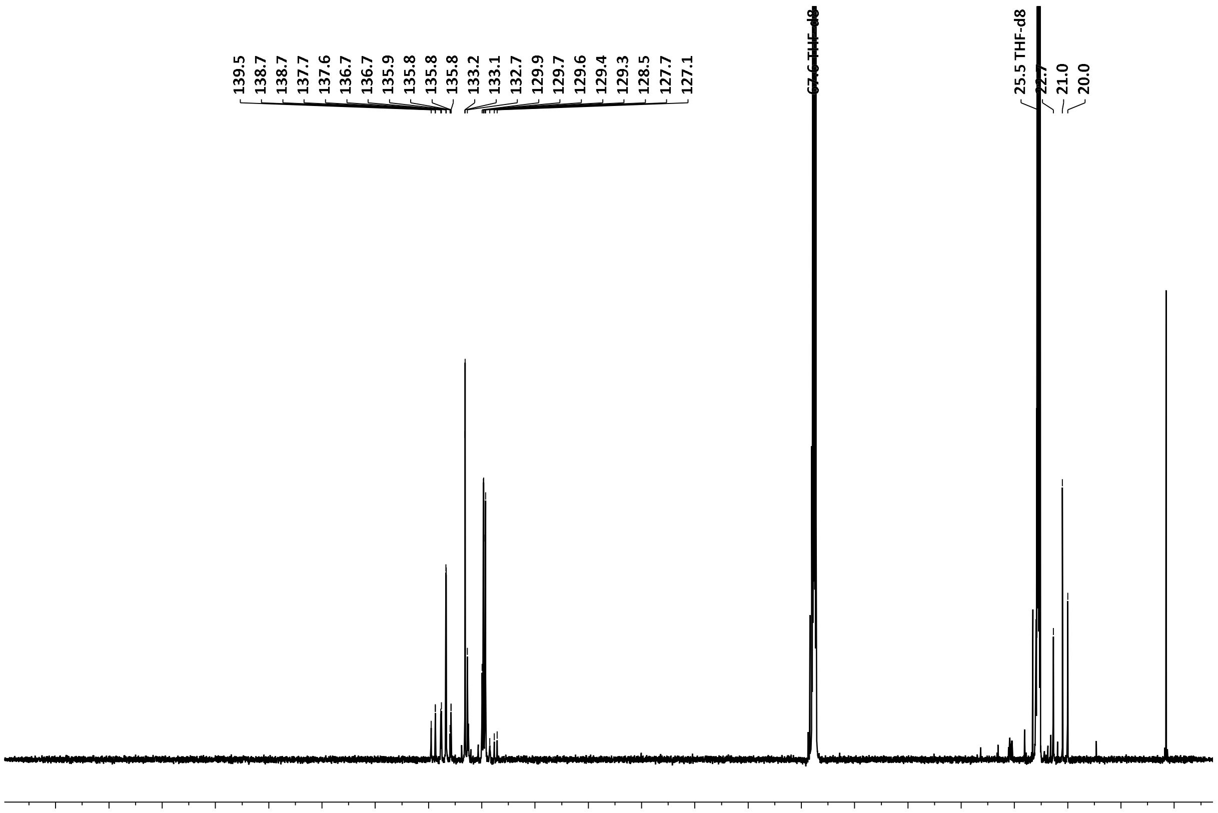

Figure S73: $^{13}C\{^{1}H\}$ NMR spectrum (THF-d_8, 151 MHz) of **6SbS**.

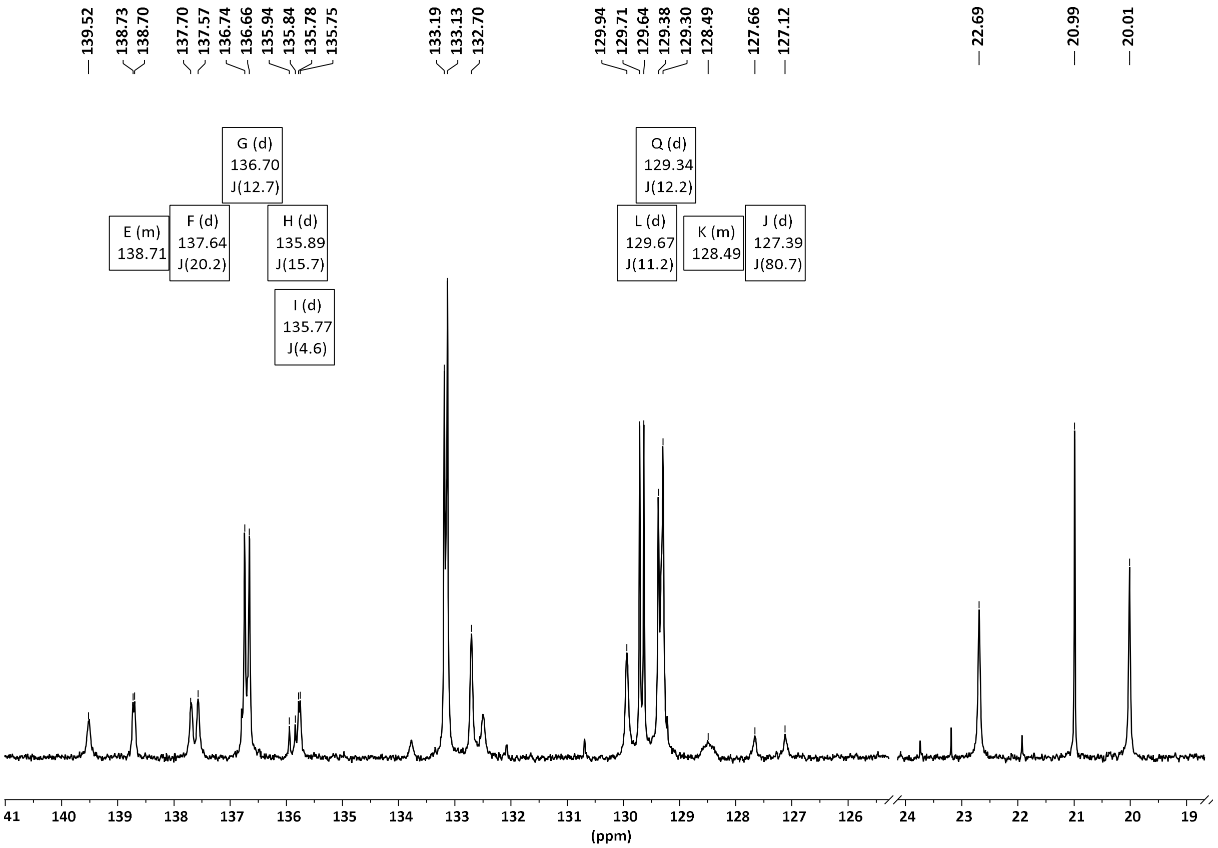

Figure S74: Detailed $^{13}C\{^1H\}$ NMR spectrum (THF-d$_8$, 151 MHz) of **6SbS**.

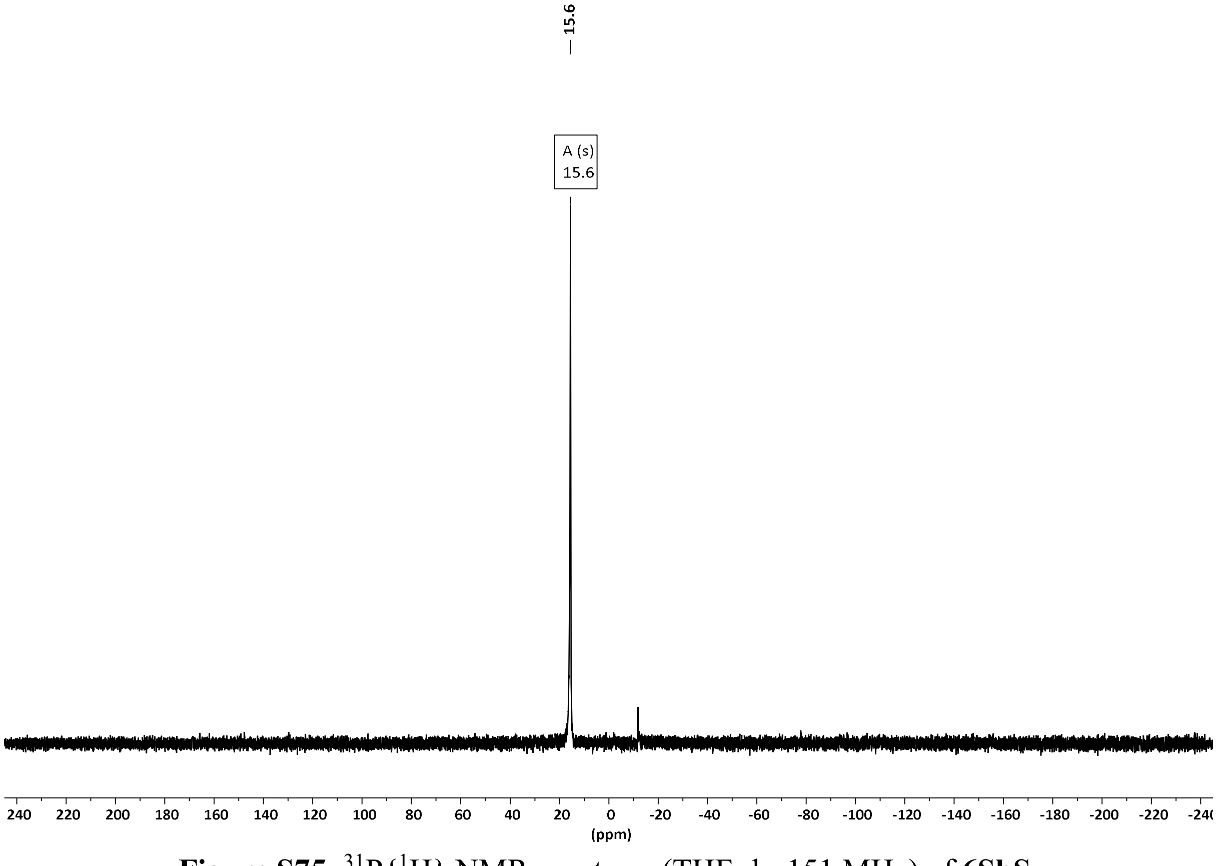

Figure S75: $^{31}P\{^1H\}$ NMR spectrum (THF-d$_8$, 151 MHz) of **6SbS**.

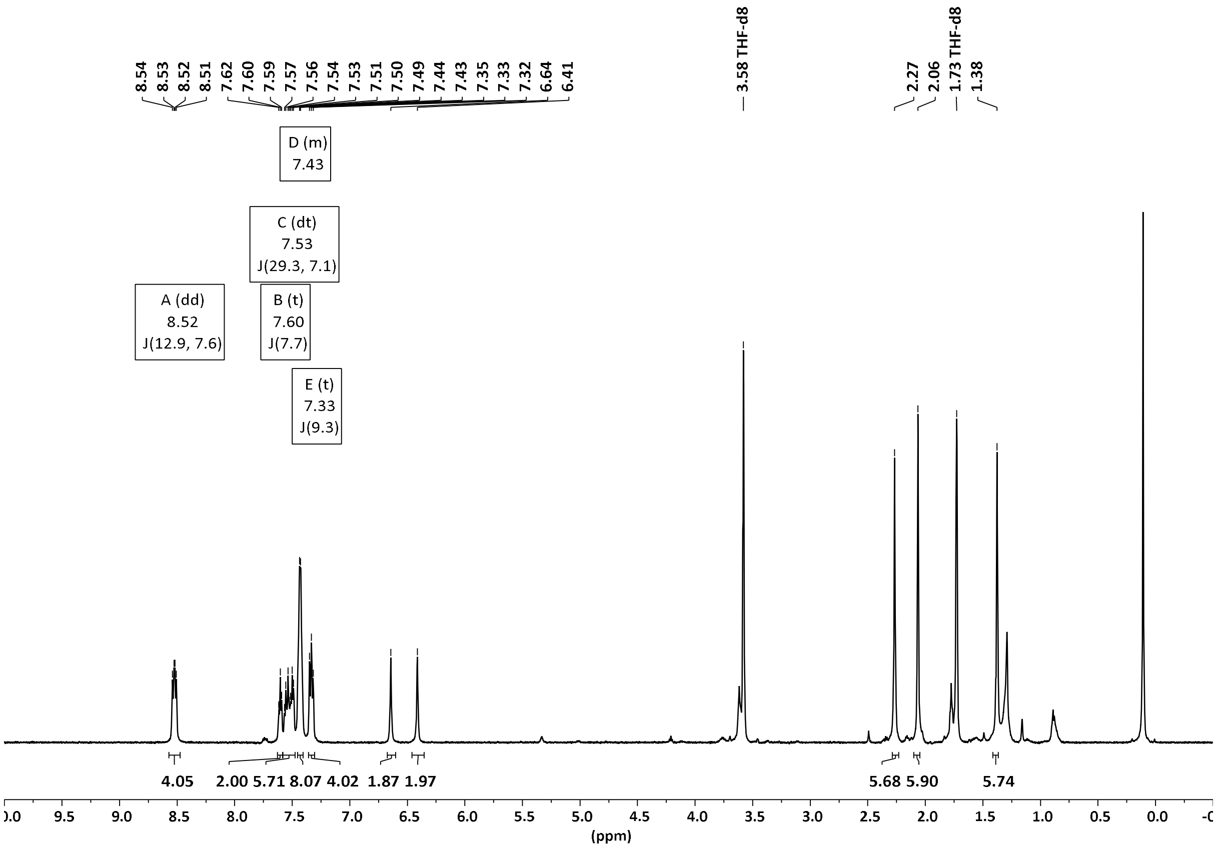

Figure S76: ^{1}H NMR spectrum (THF-d_8, 600 MHz) of **6SbSe**.

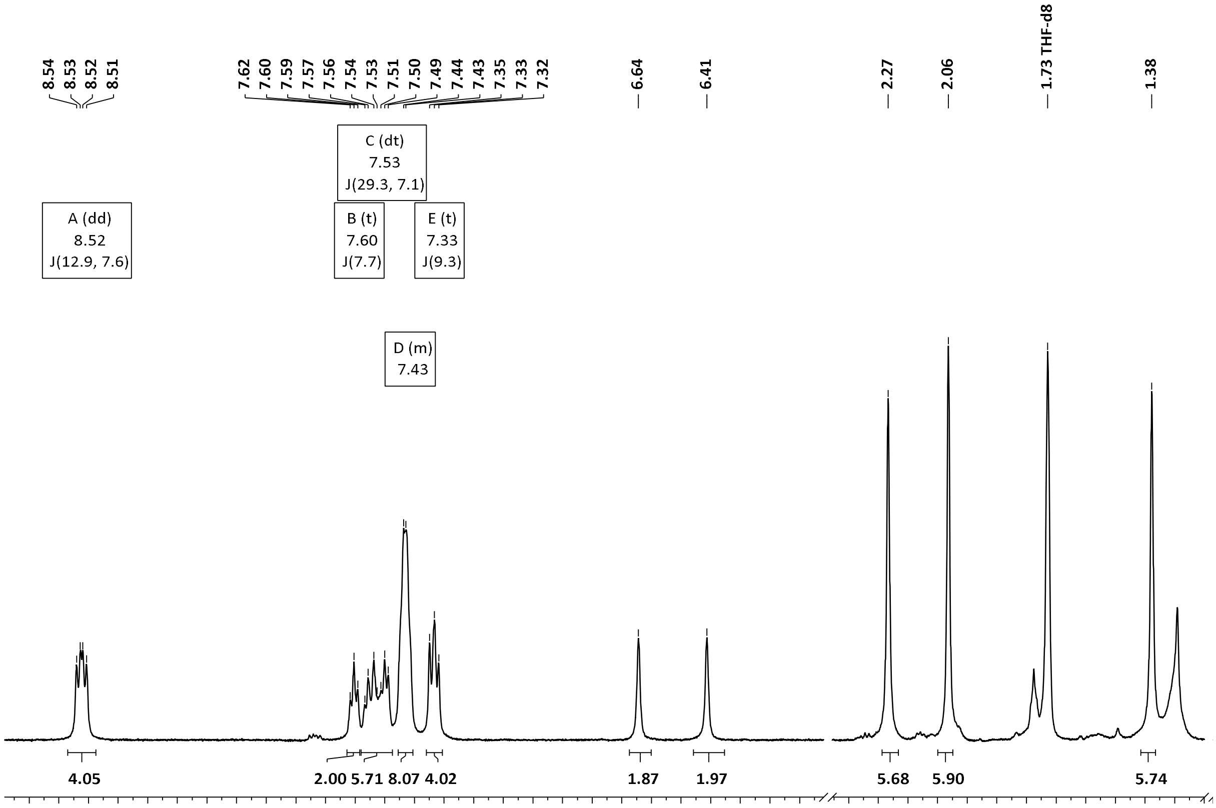

Figure S77: Detailed ^{1}H NMR spectrum (THF-d_8, 600 MHz) of **6SbSe**.

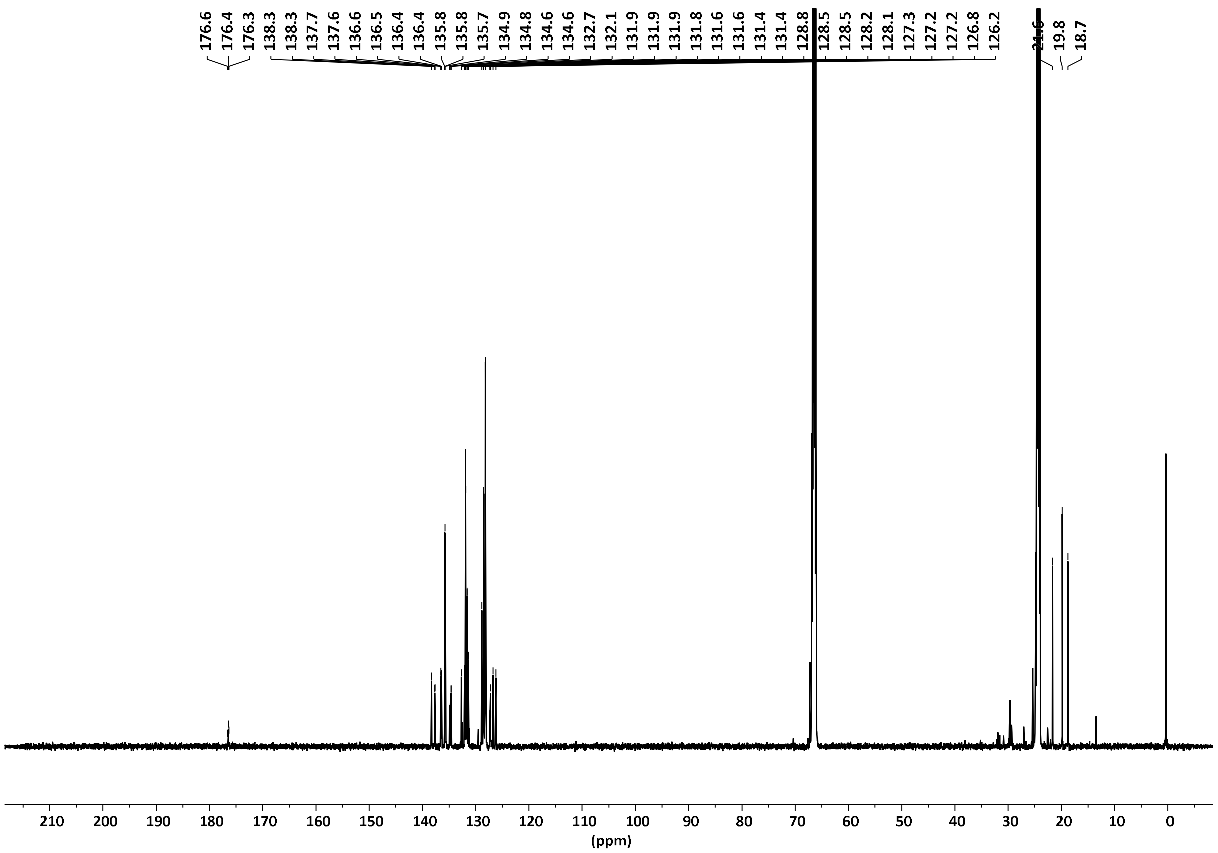

Figure S78: $^{13}C\{^{1}H\}$ NMR spectrum (THF-d$_8$, 151 MHz) of **6SbSe**.

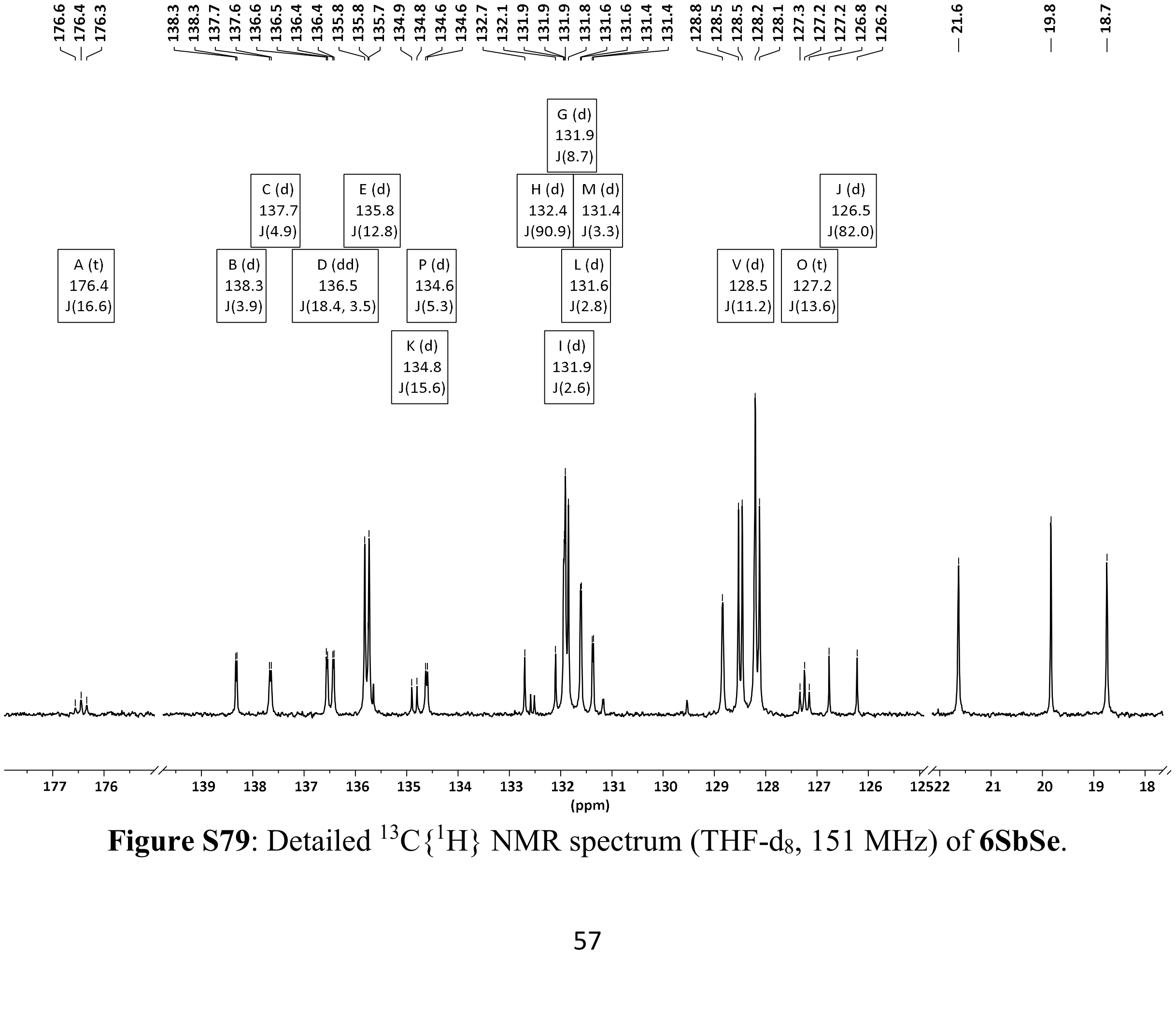

Figure S79: Detailed $^{13}C\{^{1}H\}$ NMR spectrum (THF-d$_8$, 151 MHz) of **6SbSe**.

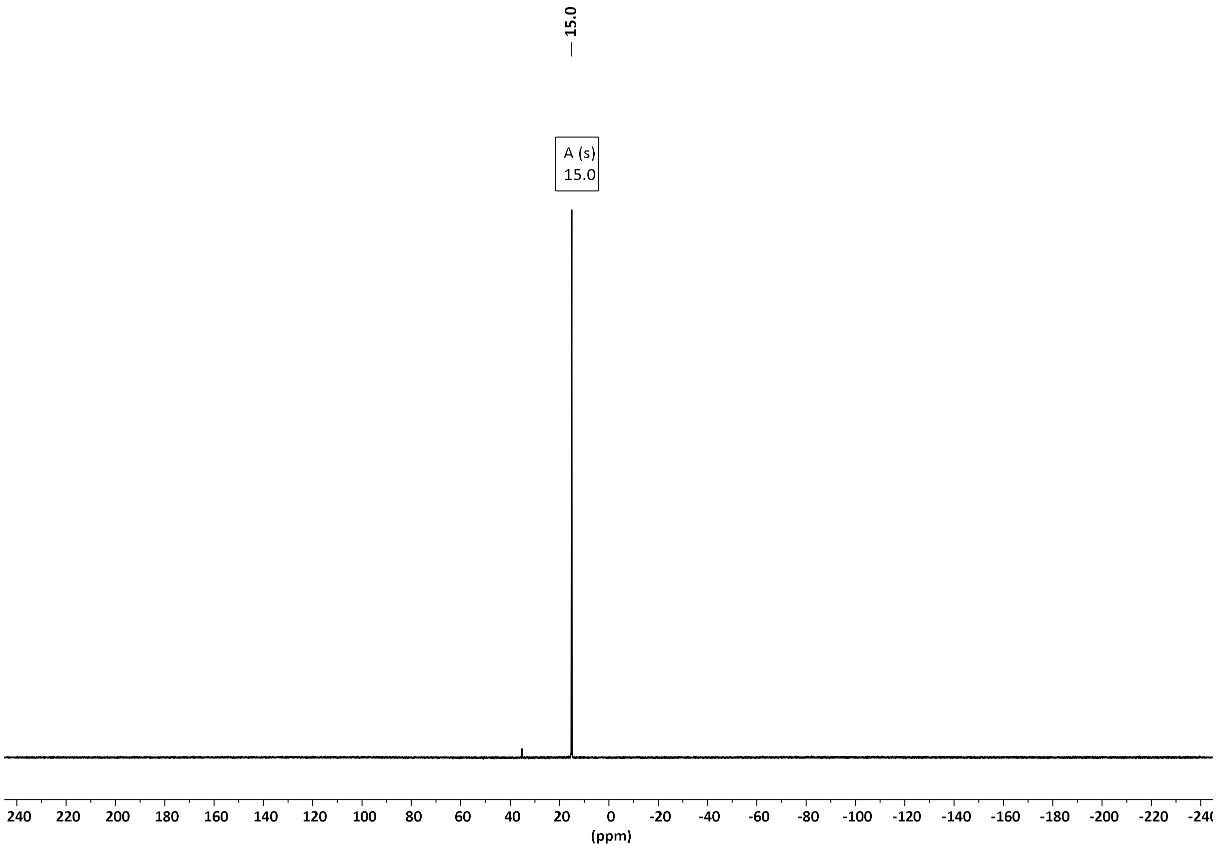

Figure S80: $^{31}P\{^{1}H\}$ NMR spectrum (THF-d$_8$, 151 MHz) of **6SbSe**.

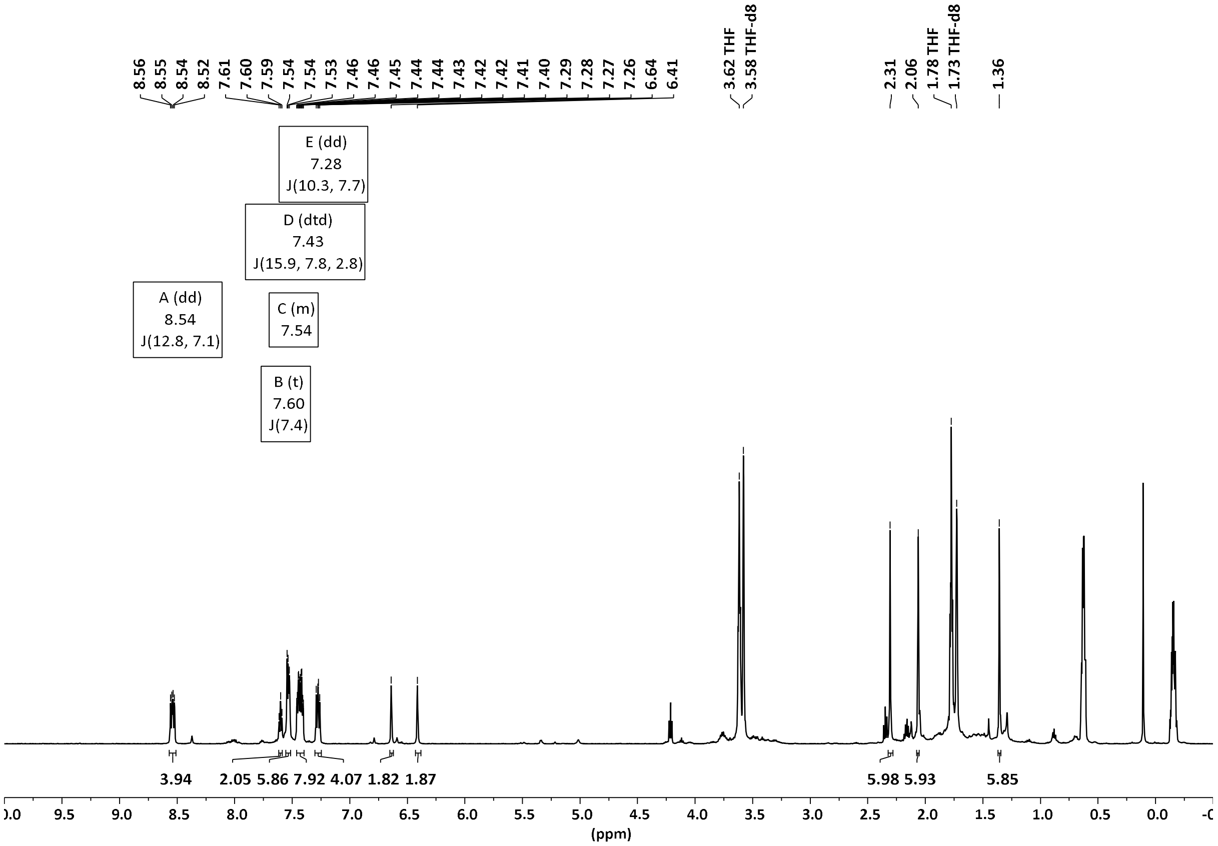

Figure S81: ^{1}H NMR spectrum (THF-d$_8$, 600 MHz) of **6SbTe**.

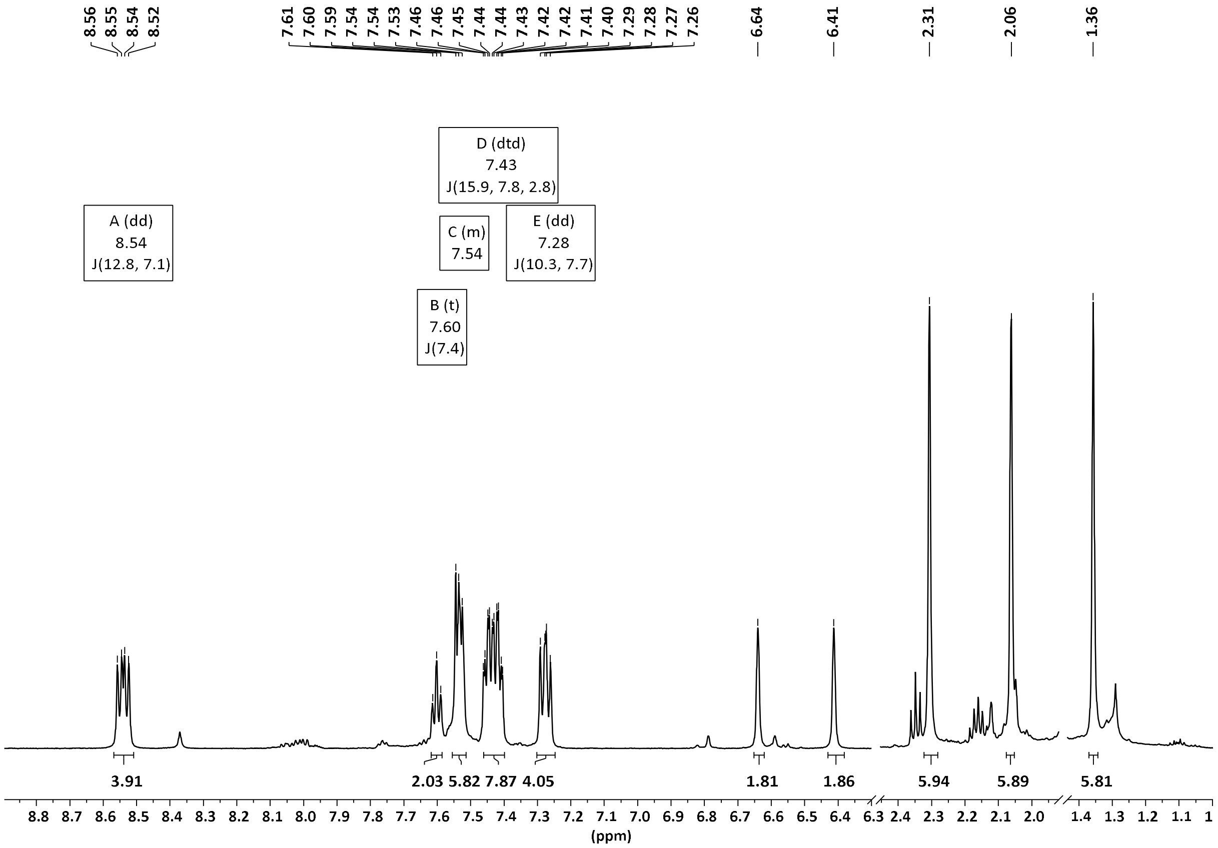

Figure S82: Detailed ^{1}H NMR spectrum (THF-d_8, 600 MHz) of **6SbTe**.

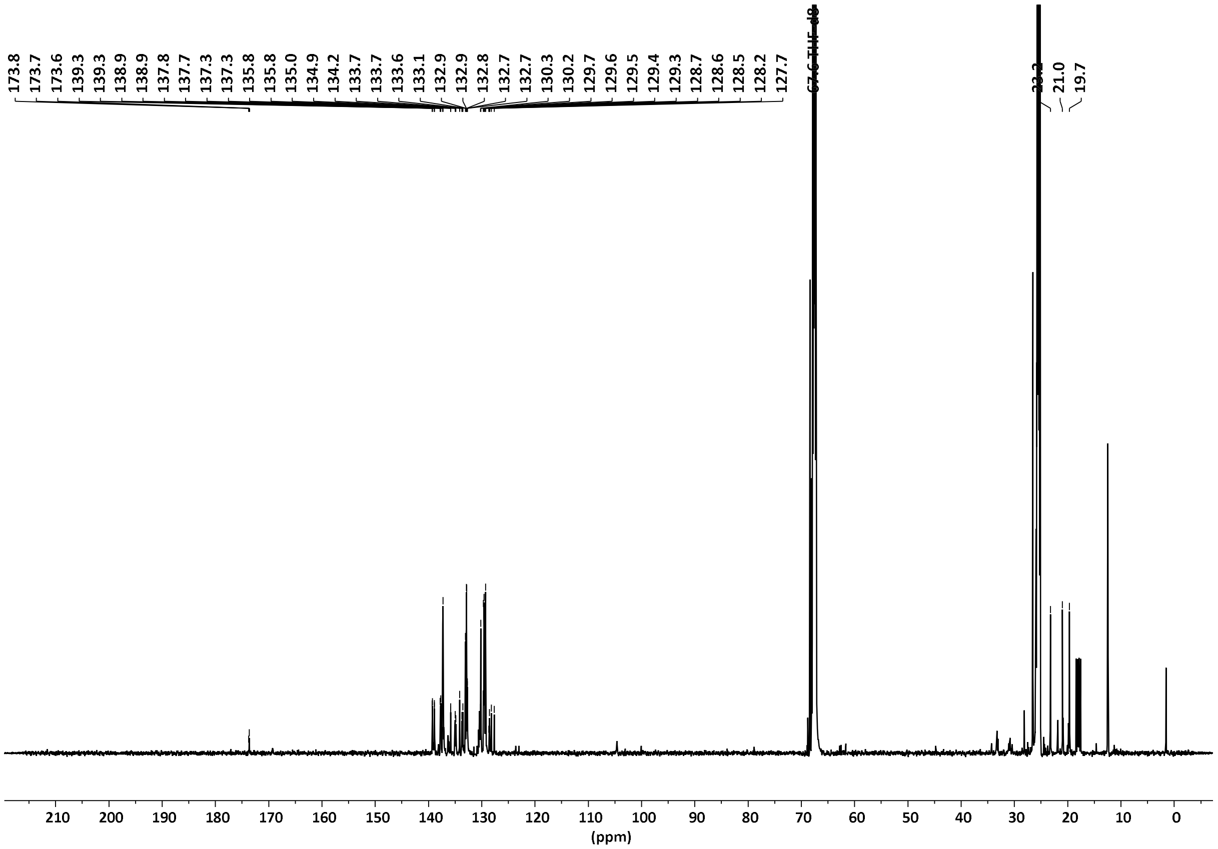

Figure S83: $^{13}C\{^{1}H\}$ NMR spectrum (THF-d_8, 151 MHz) of **6SbTe**.

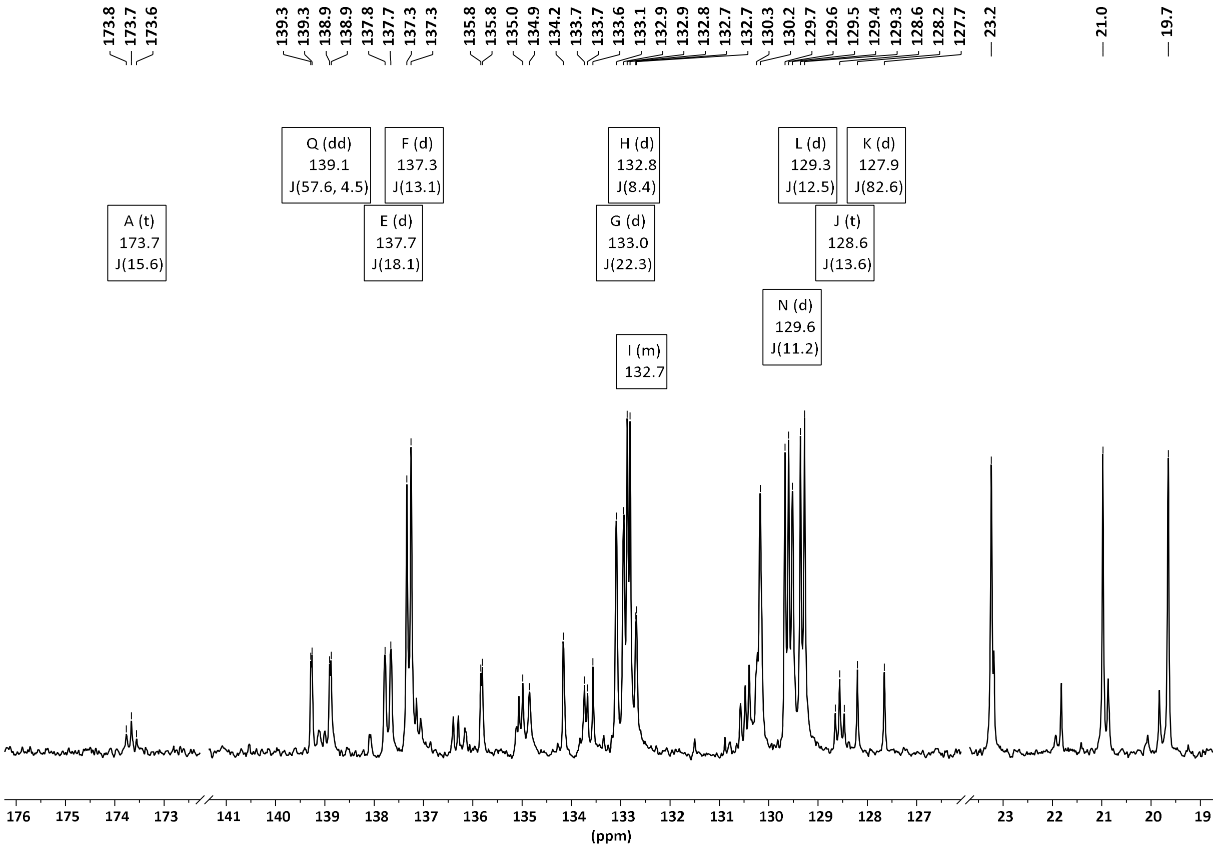

Figure S84: Detailed $^{13}C\{^{1}H\}$ NMR spectrum (THF-d$_8$, 151 MHz) of **6SbTe**.

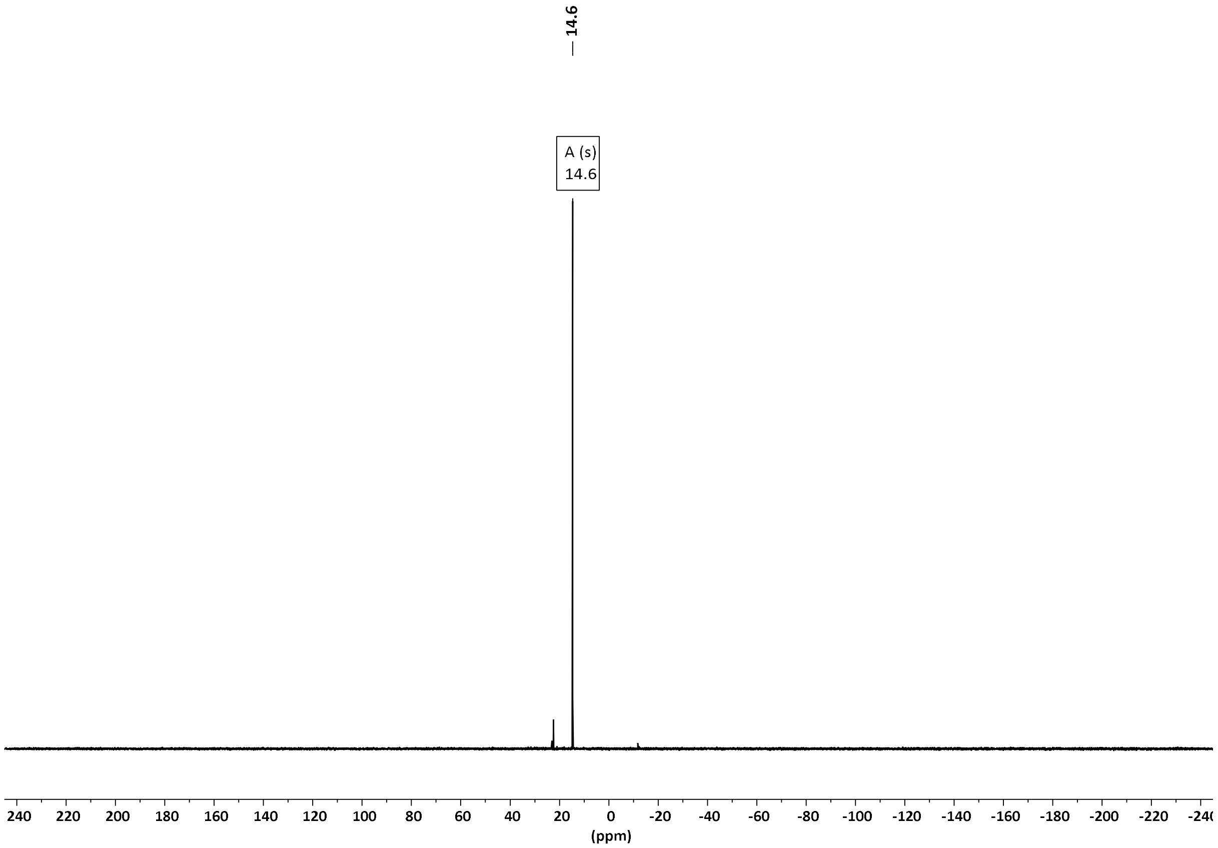

Figure S85: $^{31}P\{^{1}H\}$ NMR spectrum (THF-d$_8$, 151 MHz) of **6SbTe**.

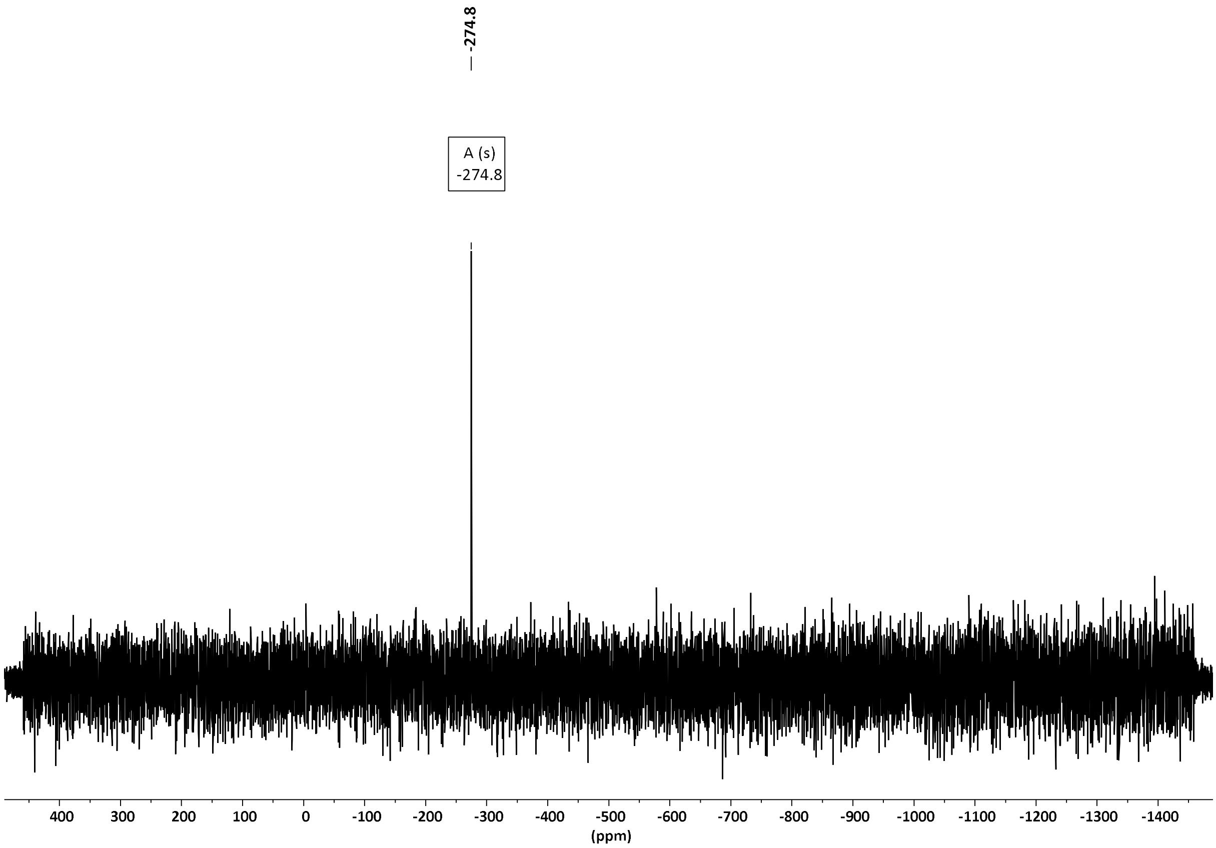

Figure S86: ^{125}Te NMR spectrum (THF-d_8, 189 MHz) of **6SbTe**.

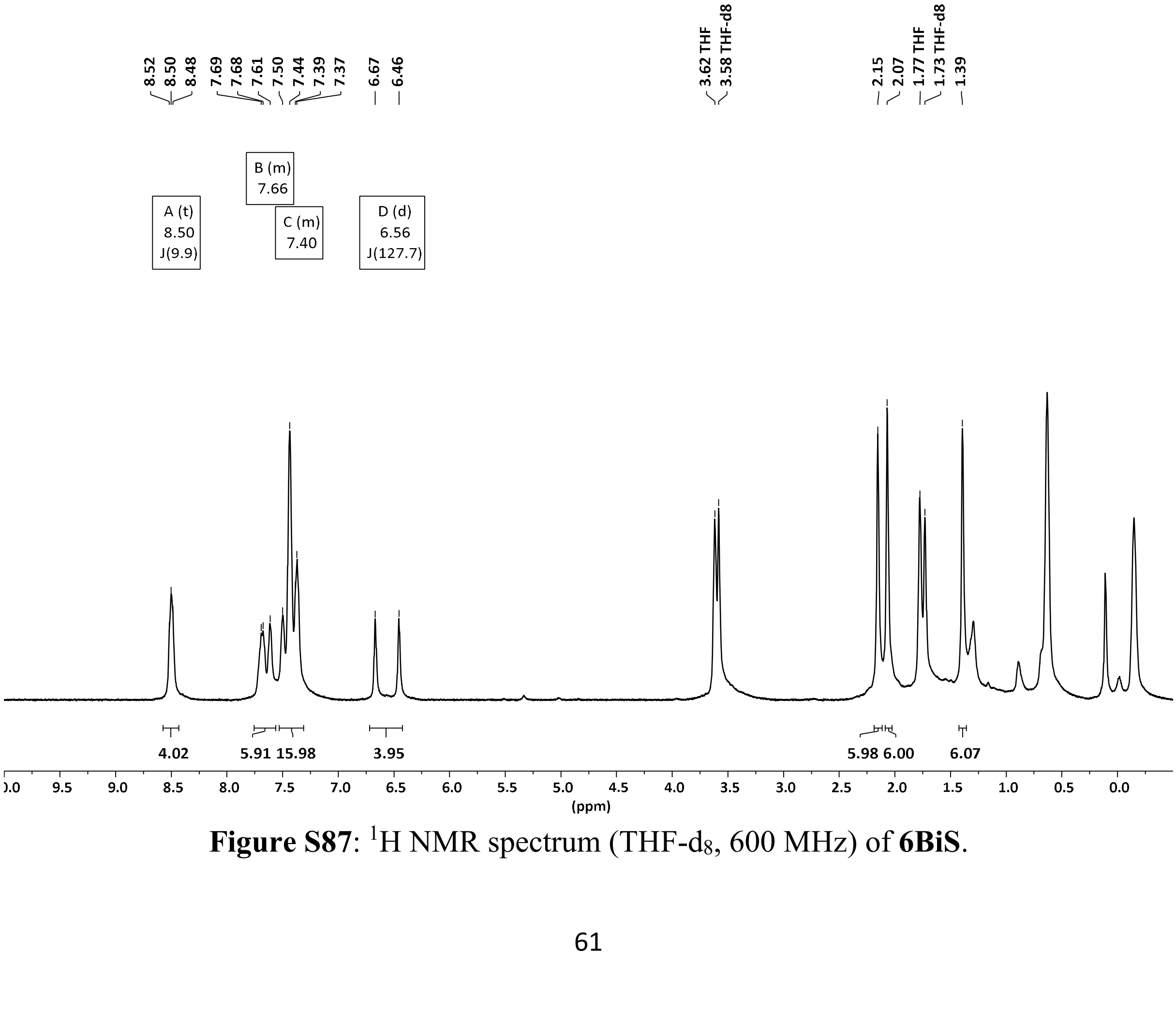

Figure S87: ^{1}H NMR spectrum (THF-d_8, 600 MHz) of **6BiS**.

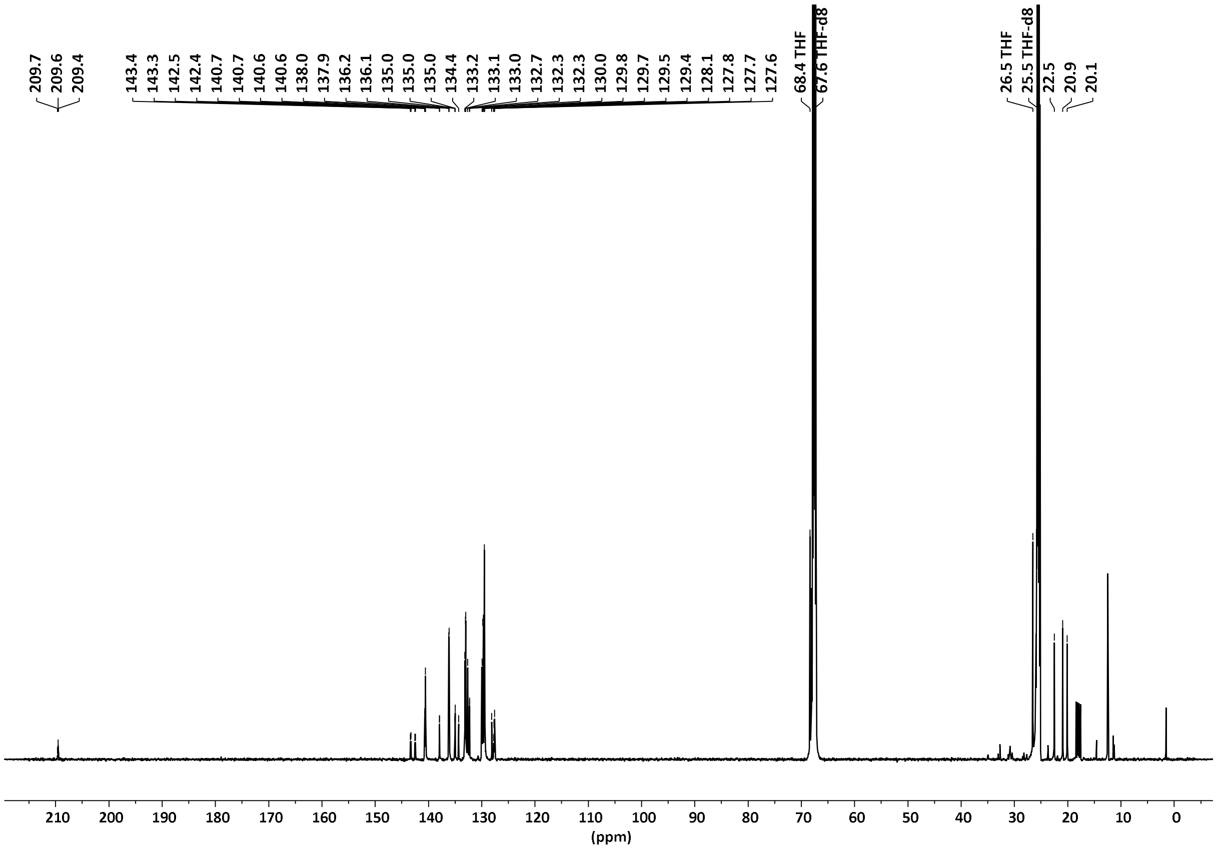

Figure S88: $^{13}C\{^1H\}$ NMR spectrum (THF-d$_8$, 151 MHz) of **6BiS**.

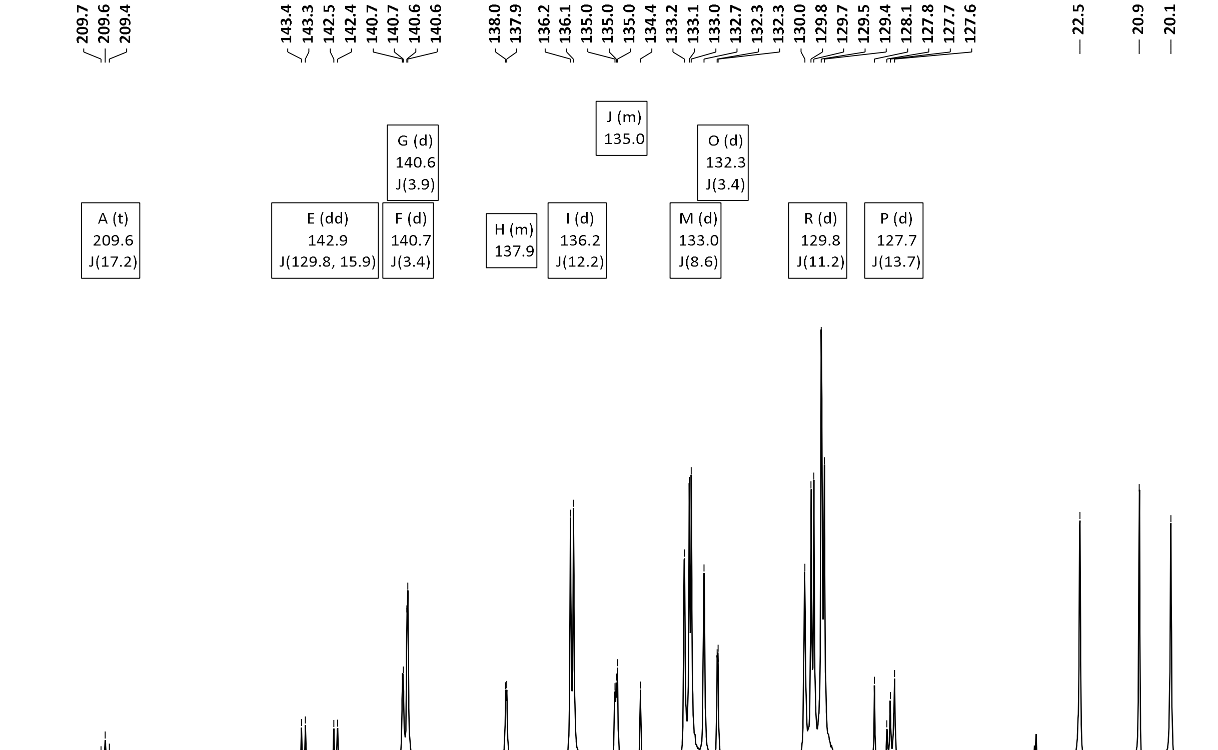

Figure S89: Detailed $^{13}C\{^1H\}$ NMR spectrum (THF-d$_8$, 151 MHz) of **6BiS**.

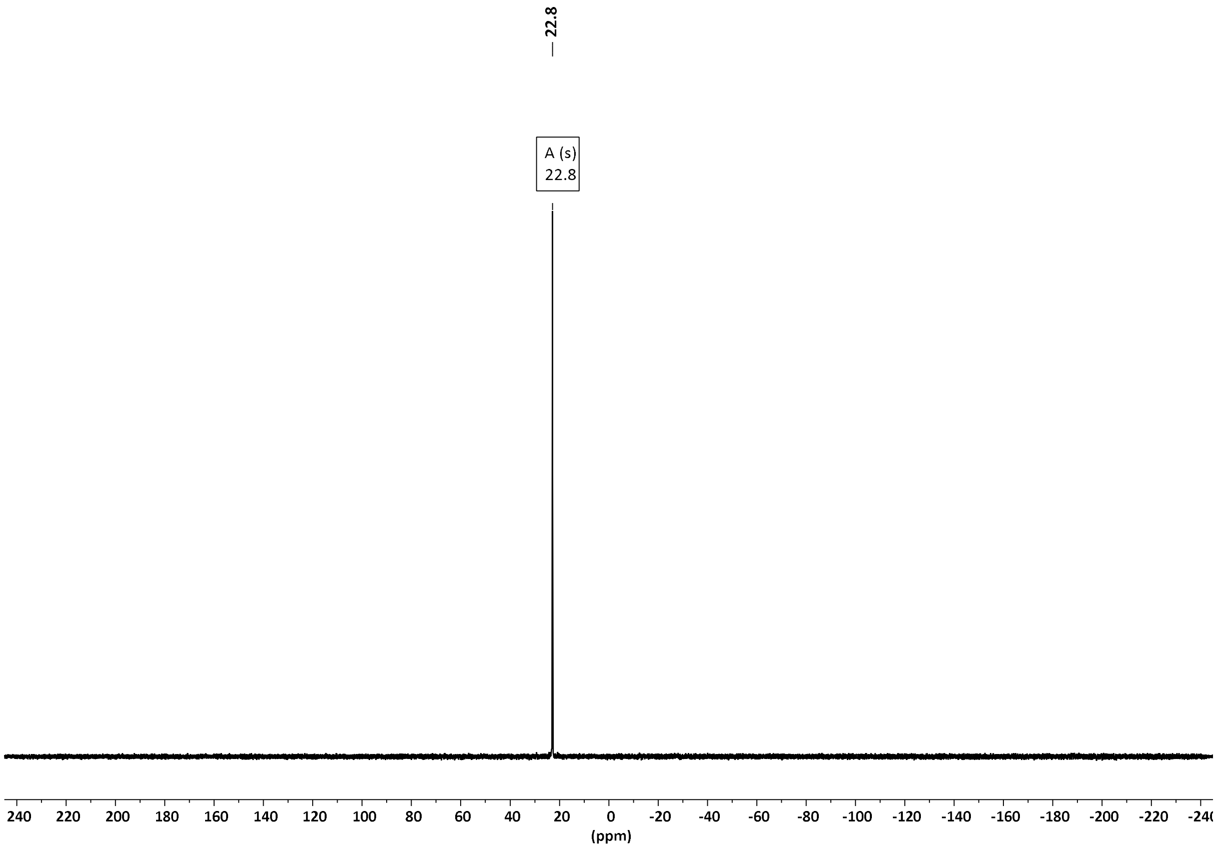

Figure S90: $^{31}P\{^{1}H\}$ NMR spectrum (THF-d_8, 151 MHz) of **6BiS**.

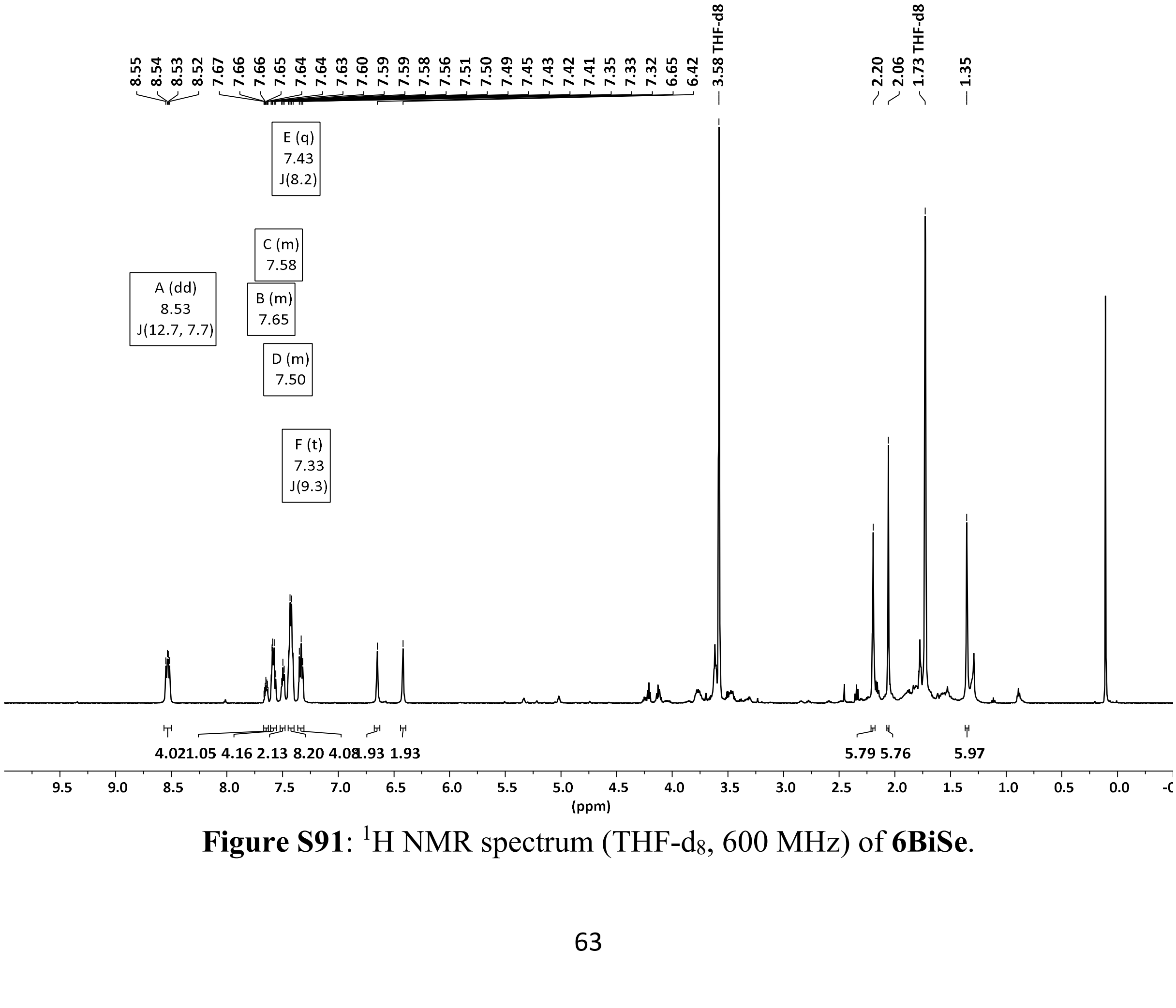

Figure S91: ^{1}H NMR spectrum (THF-d_8, 600 MHz) of **6BiSe**.

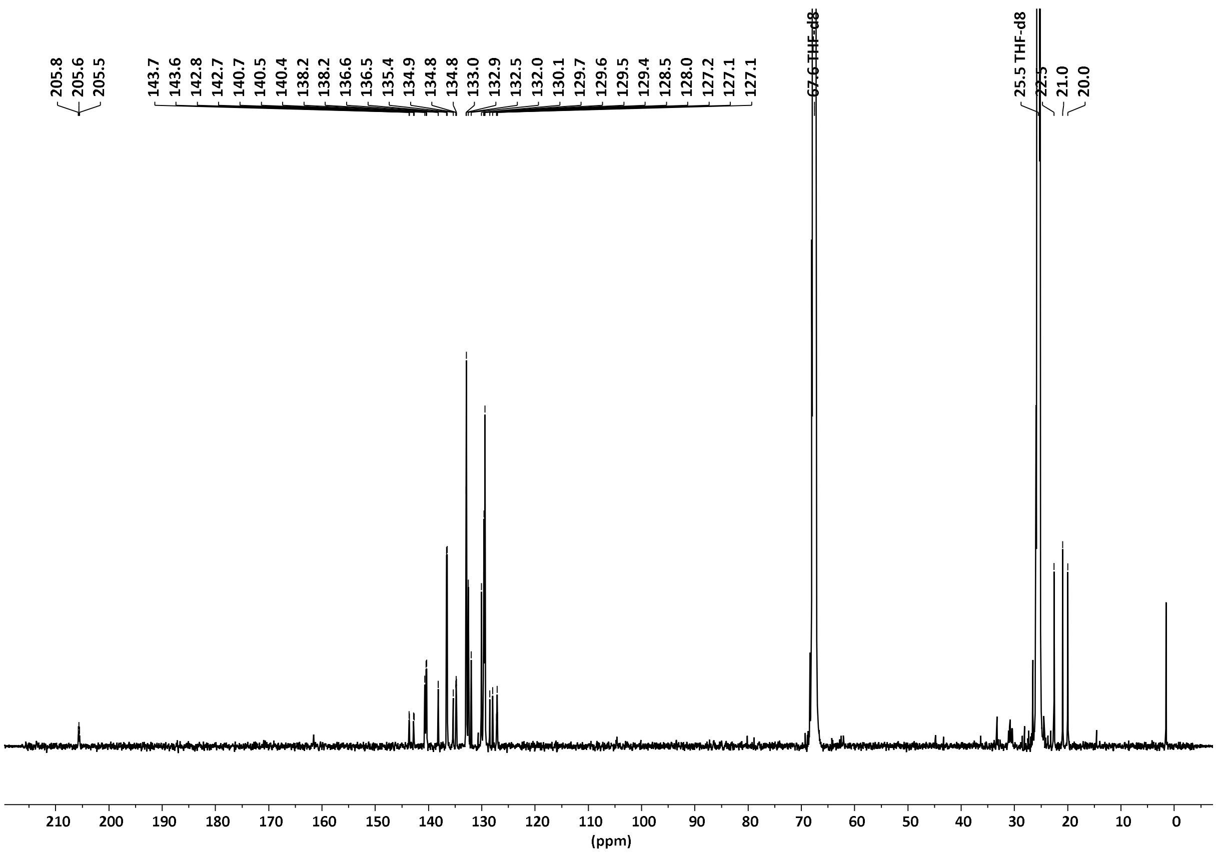

Figure S92: $^{13}C\{^{1}H\}$ NMR spectrum (THF-d_8, 151 MHz) of **6BiSe**.

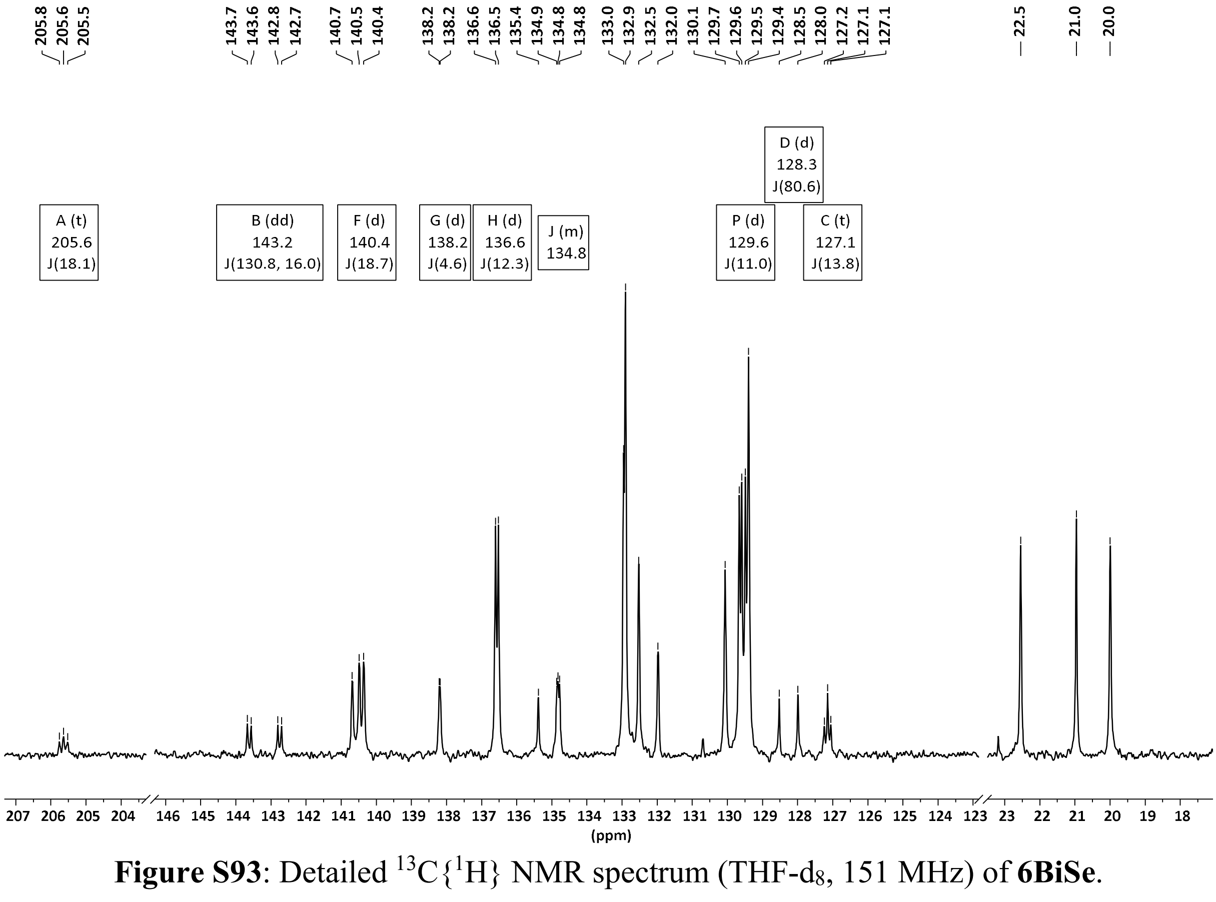

Figure S93: Detailed $^{13}C\{^{1}H\}$ NMR spectrum (THF-d_8, 151 MHz) of **6BiSe**.

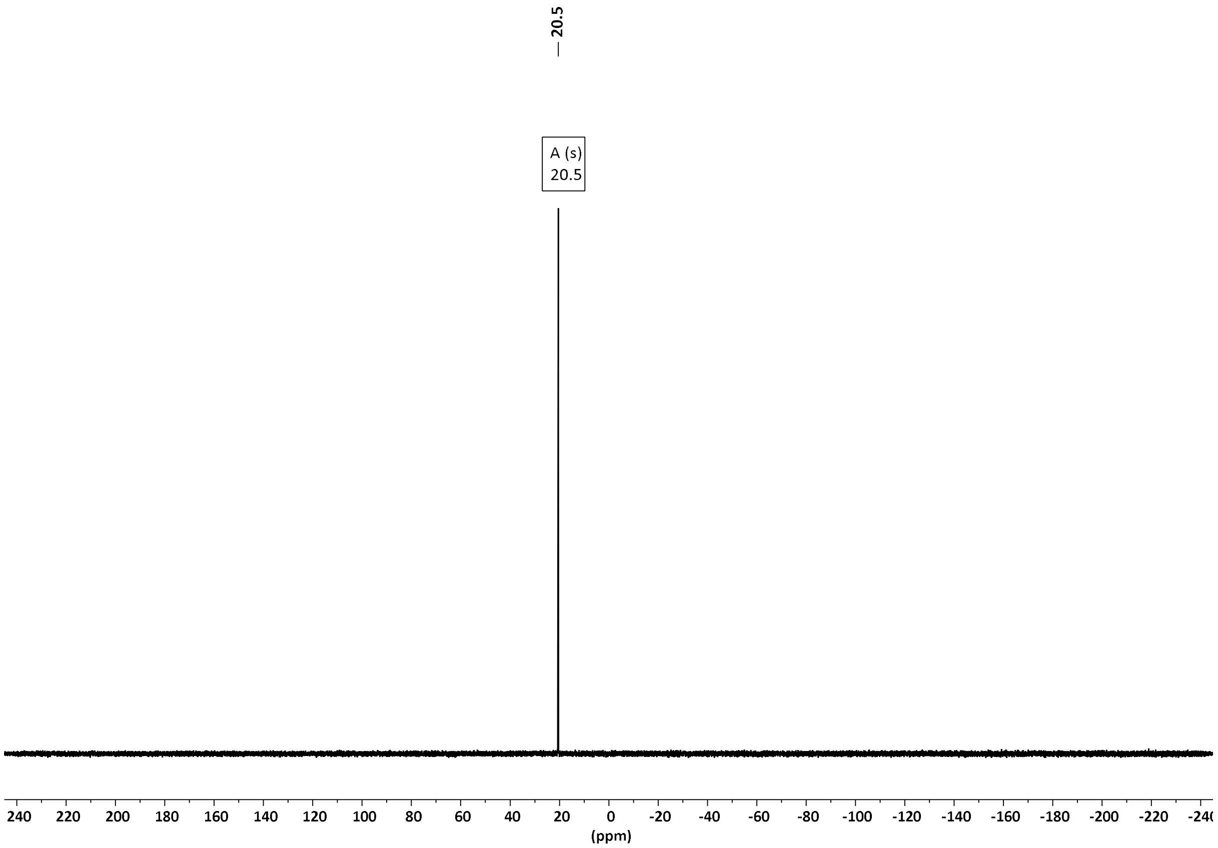

Figure S94: $^{31}P\{^{1}H\}$ NMR spectrum (THF-d$_8$, 151 MHz) of **6BiSe**.

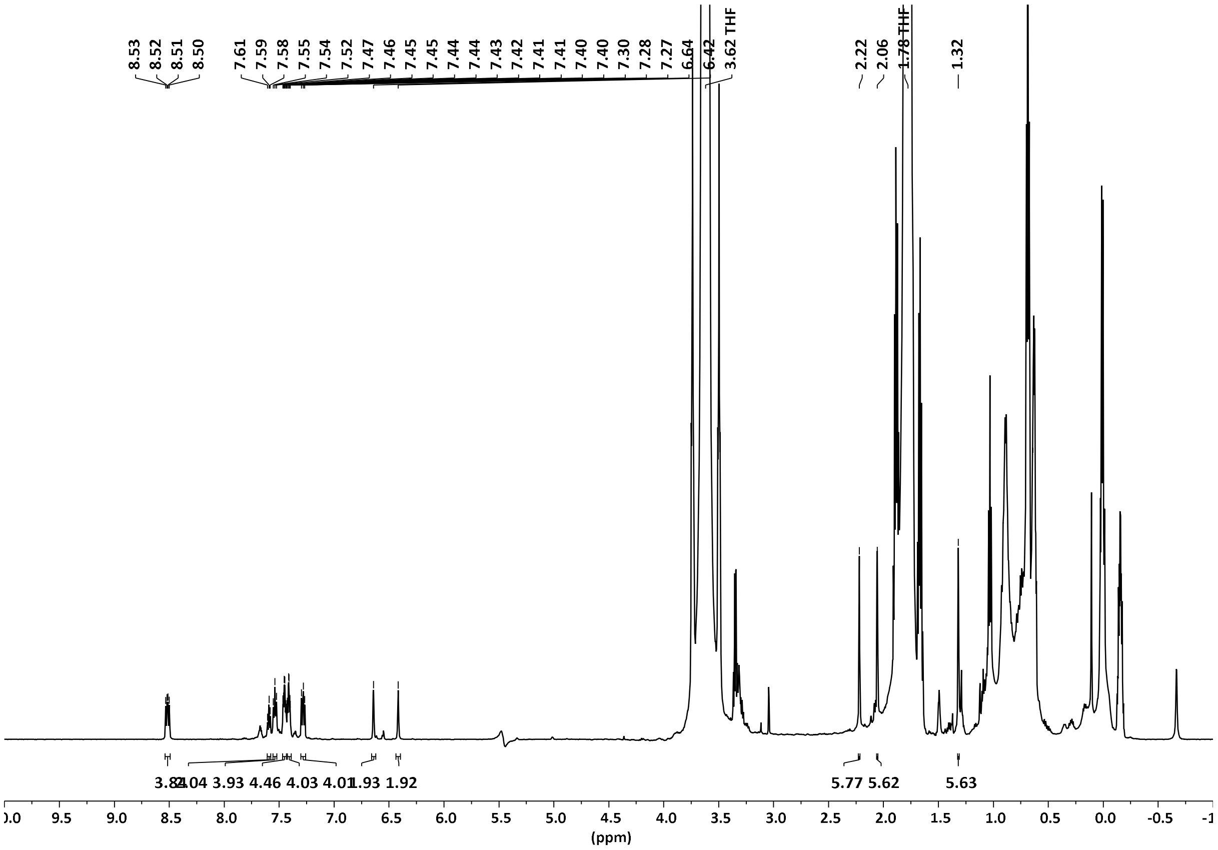

Figure S95: ^{1}H NMR spectrum (THF-d$_8$, 600 MHz) of **6BiTe**.

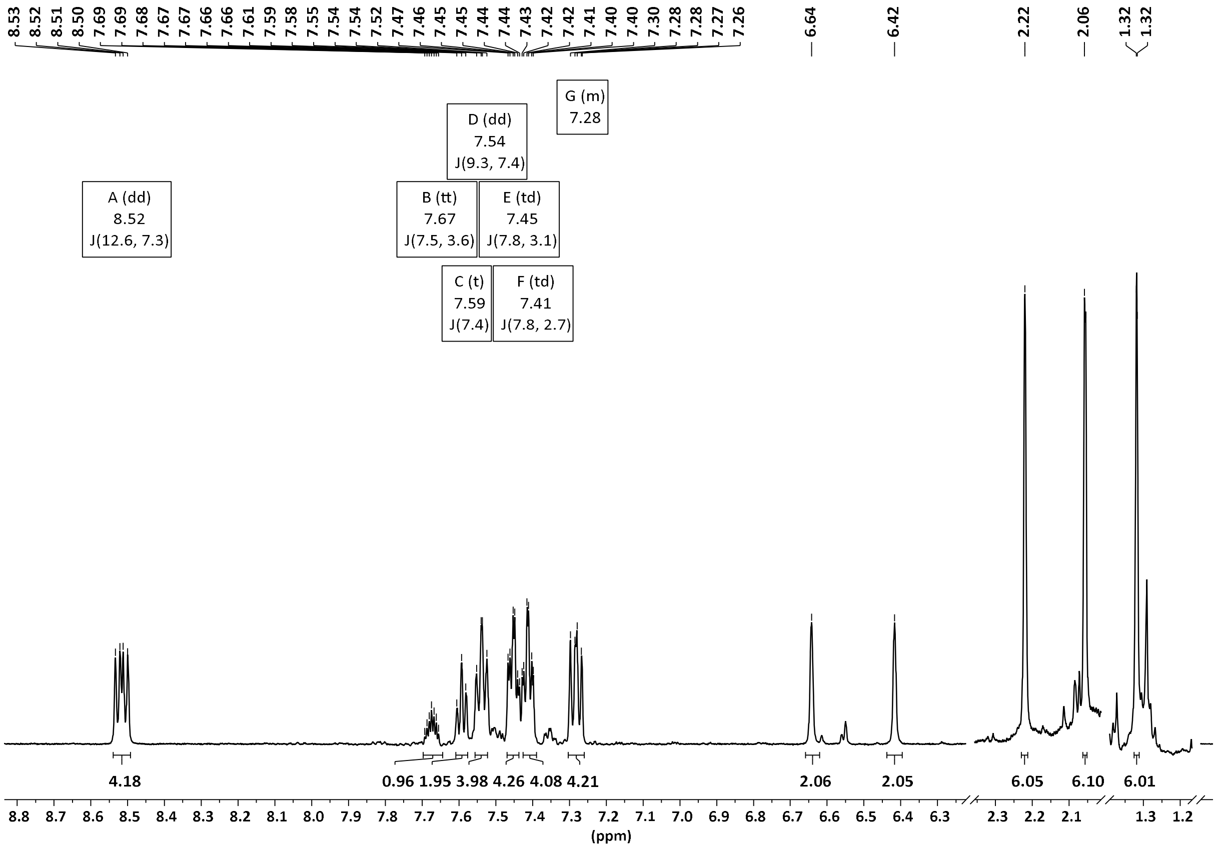

Figure S96: Detailed ^{1}H NMR spectrum (THF-d$_8$, 600 MHz) of **6BiTe**.

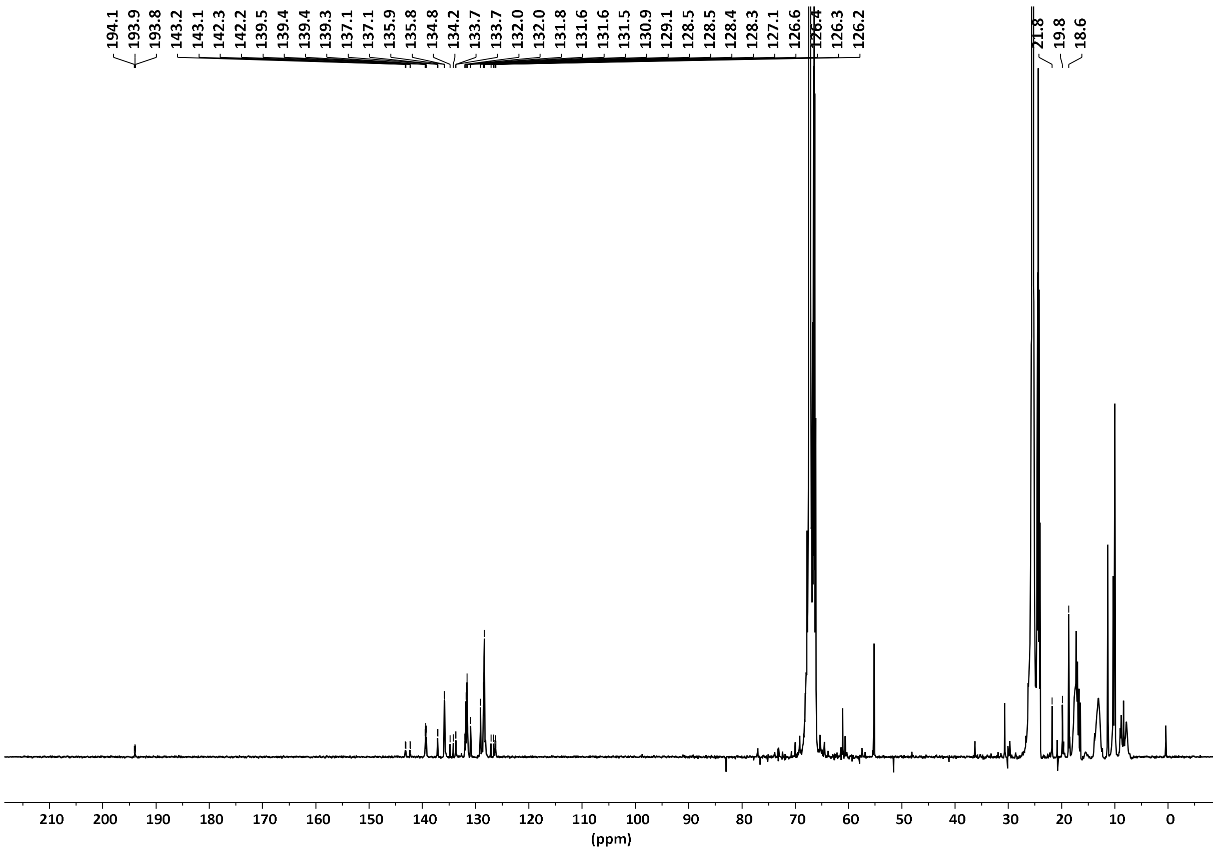

Figure S97: ^{13}C{^{1}H} NMR spectrum (THF-d$_8$, 151 MHz) of **6BiTe**.

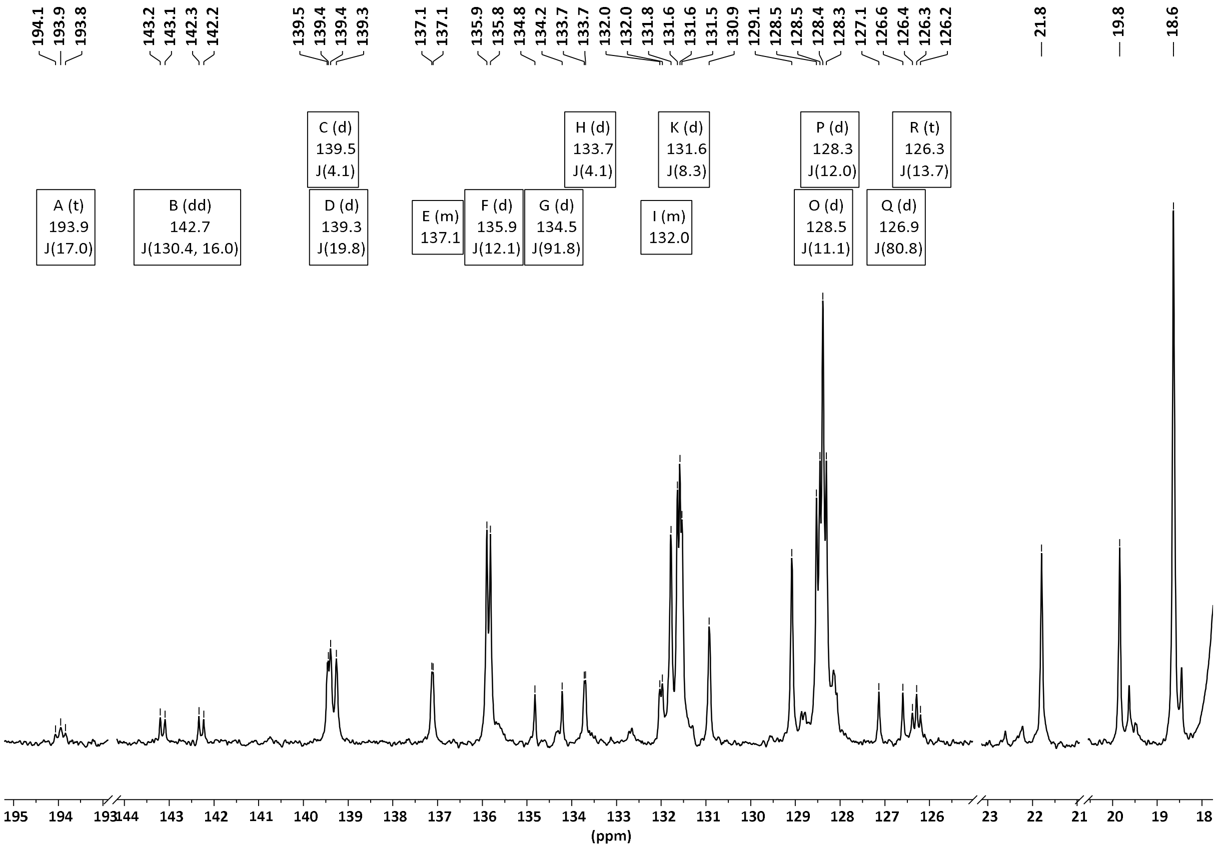

Figure S98: Detailed $^{13}C\{^{1}H\}$ NMR spectrum (THF-d$_8$, 151 MHz) of **6BiTe**.

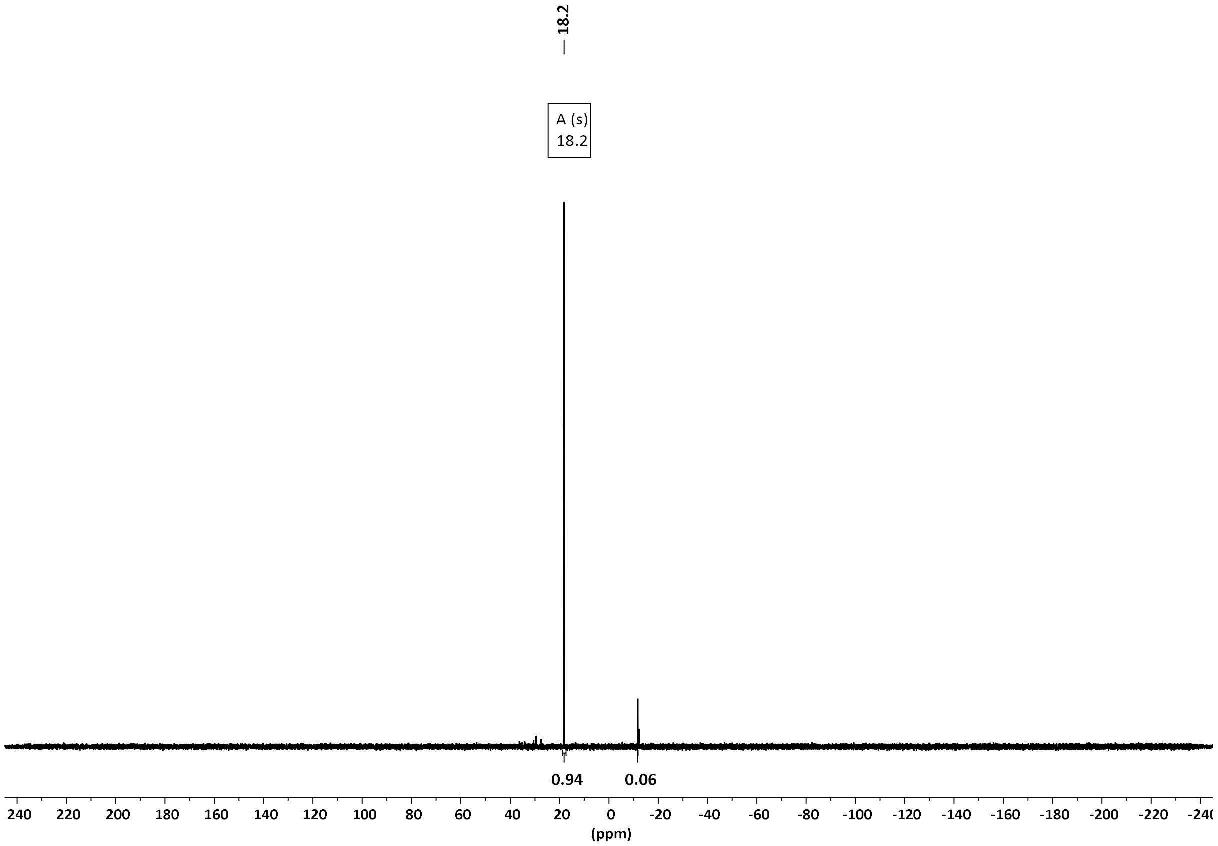

Figure S99: $^{31}P\{^{1}H\}$ NMR spectrum (THF-d$_8$, 151 MHz) of **6BiTe**.

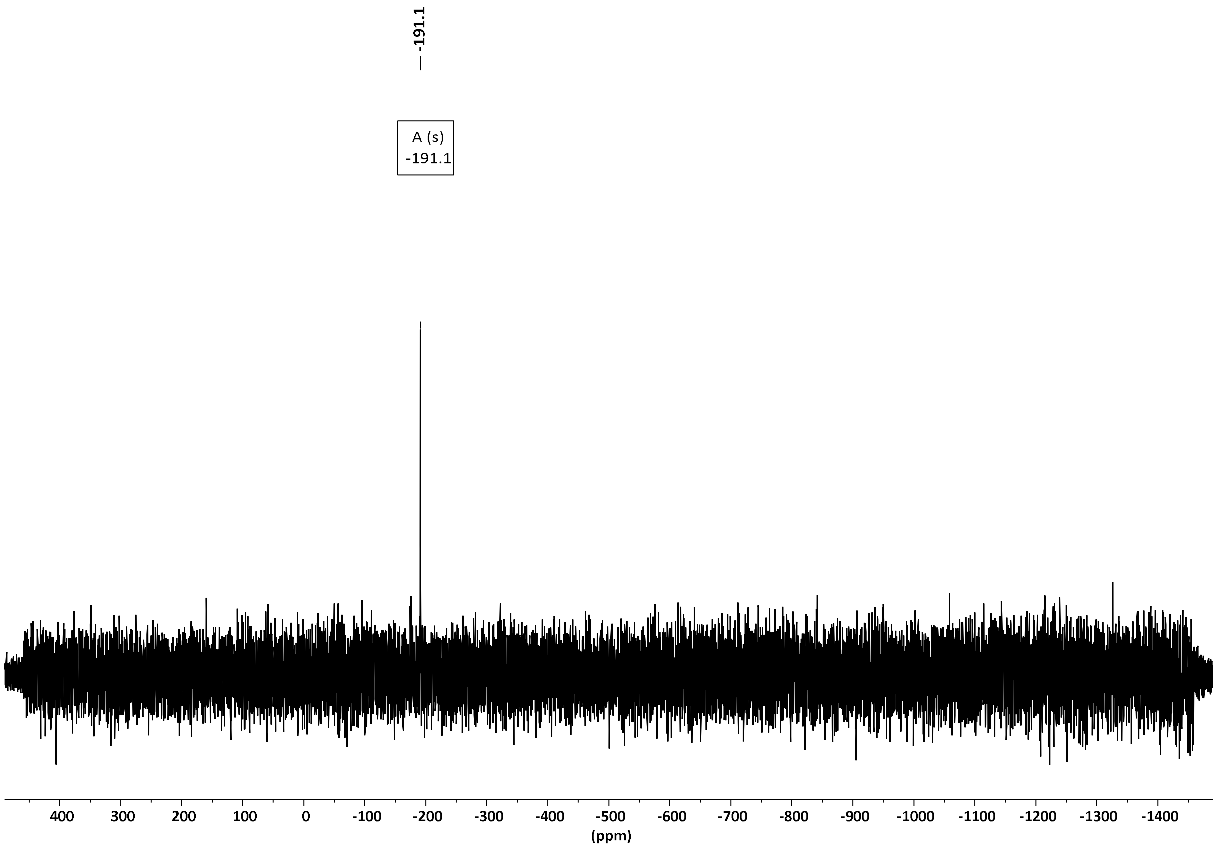

Figure S100: ^{125}Te NMR spectrum (THF-d_8, 189 MHz) of **6BiTe**.

UV-vis Spectra

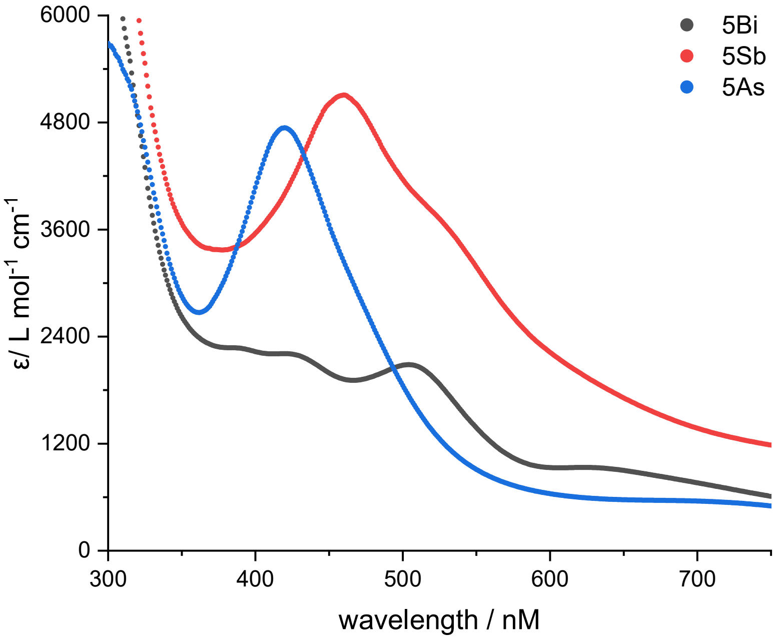

Figure S101: UV/Vis spectrum of **5As** (blue), **5Sb** (red) and **5Bi** (black) in THF as a direct comparison.

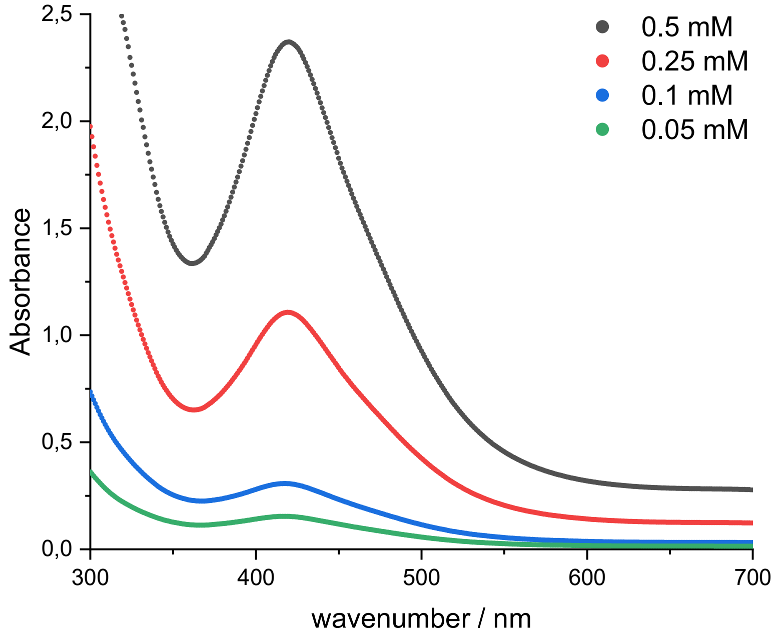

Figure S102: UV/Vis spectrum of **5As** in THF for determined concentrations.

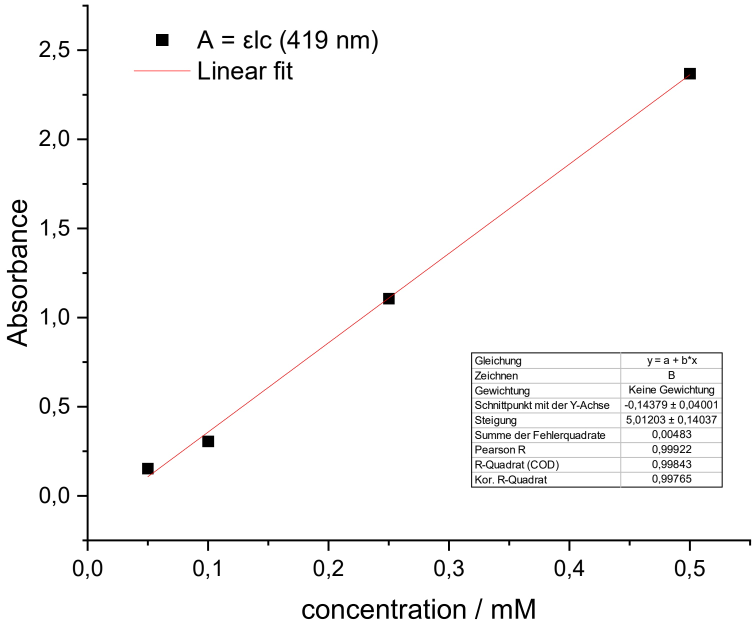

Figure S103: Concentration–absorbance dependence at 419 nm, absorption maximum of **5As** in THF. Molar absorptivity in the presented range (ε_{419}= 5.012 cm^{-1} mol^{-1}) was determined using the Lambert–Beer law.

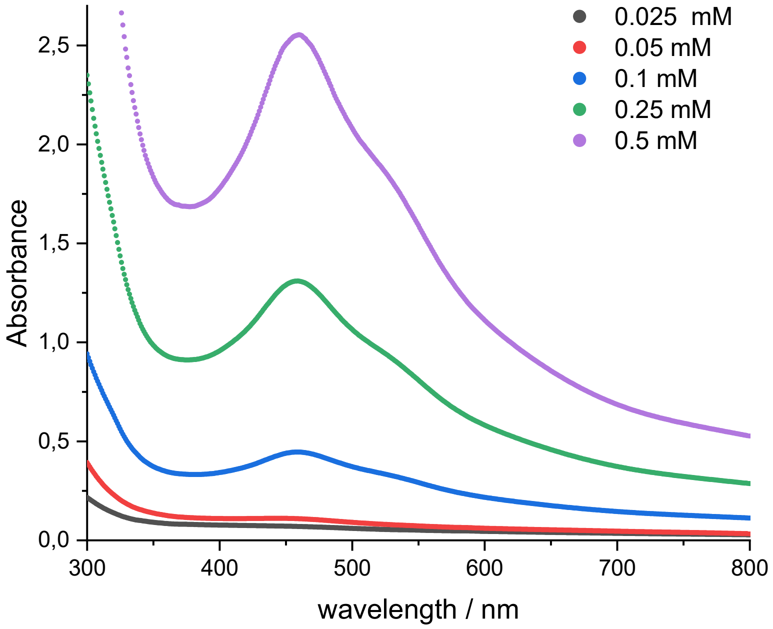

Figure S104: UV/Vis spectrum of **5Sb** in THF for determined concentrations.

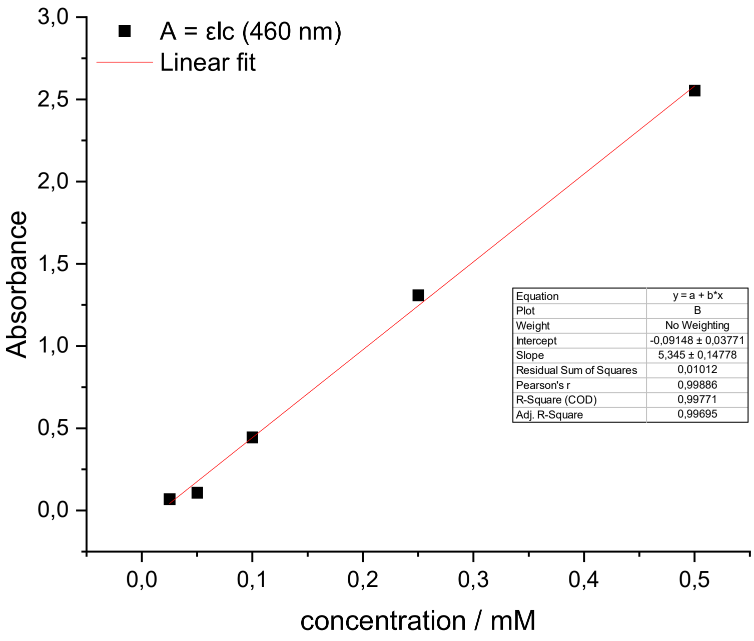

Figure S105: Concentration–absorbance dependence at 460 nm, absorption maximum of **5Sb** in THF. Molar absorptivity in the presented range (ε_{460}= 5.345 cm^{-1} mol^{-1}) was determined using the Lambert–Beer law.

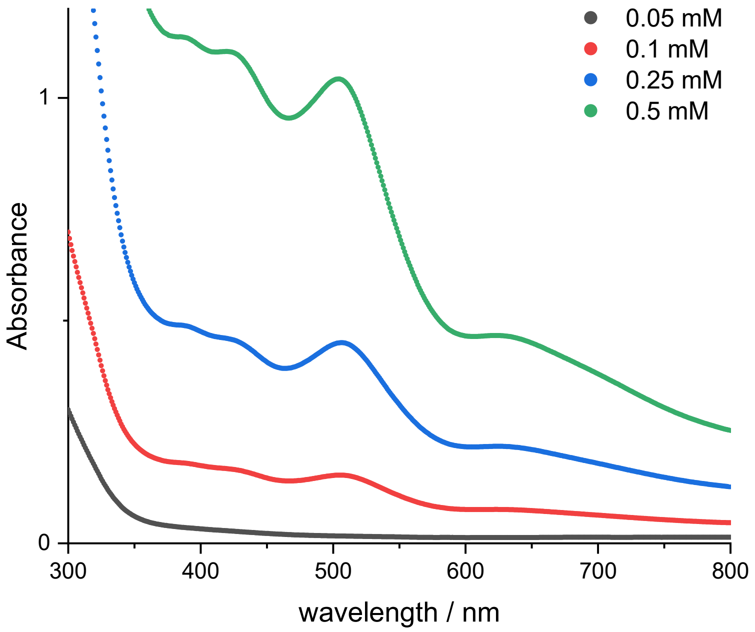

Figure S106: UV/Vis spectrum of **5Bi** in THF for determined concentrations.

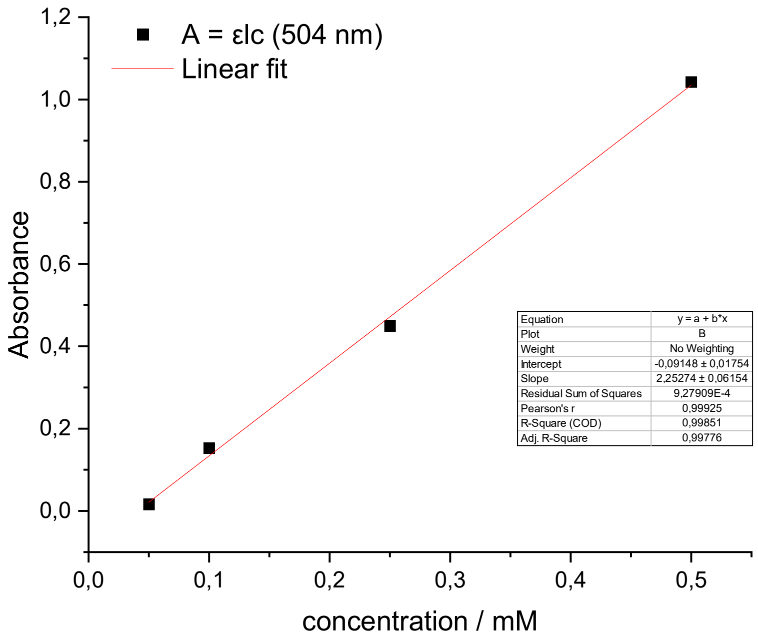

Figure S107: Concentration–absorbance dependence at 504 nm, absorption maximum of **5Bi** in THF. Molar absorptivity in the presented range (ε_{504}= 2.253 cm^{-1} mol^{-1}) was determined using the Lambert–Beer law.

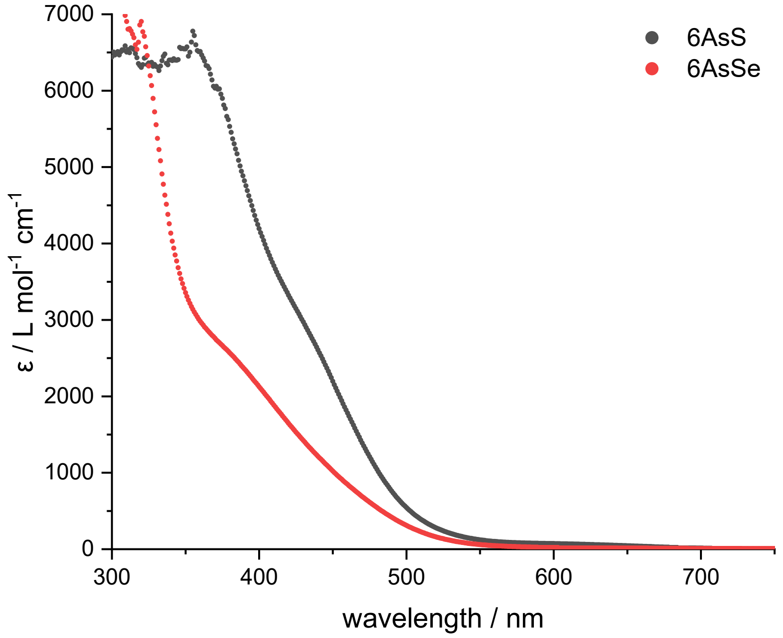

Figure S108: UV/Vis spectrum of **6AsS** (black) and **6AsSe** (red) in THF as a direct comparison**.**

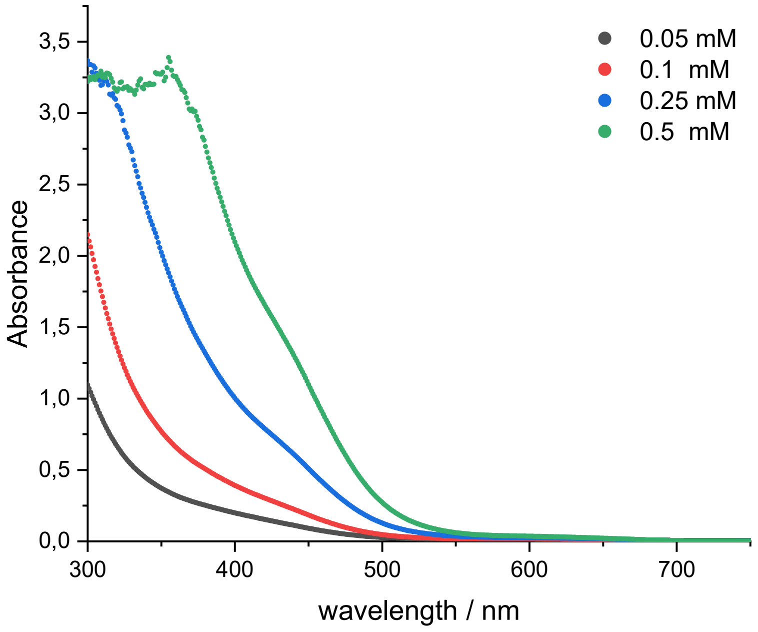

Figure S109: UV/Vis spectrum of **6AsS** in THF for determined concentrations.

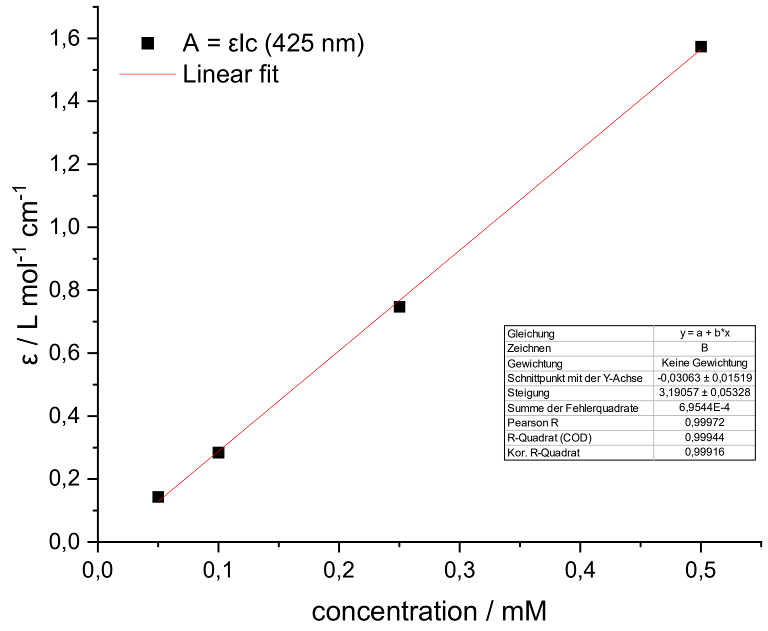

Figure S110: Concentration–absorbance dependence at 425 nm, absorption maximum of **6AsS** in THF. Molar absorptivity in the presented range (ε_{425}= 3.191 cm^{-1} mol^{-1}) was determined using the Lambert–Beer law.

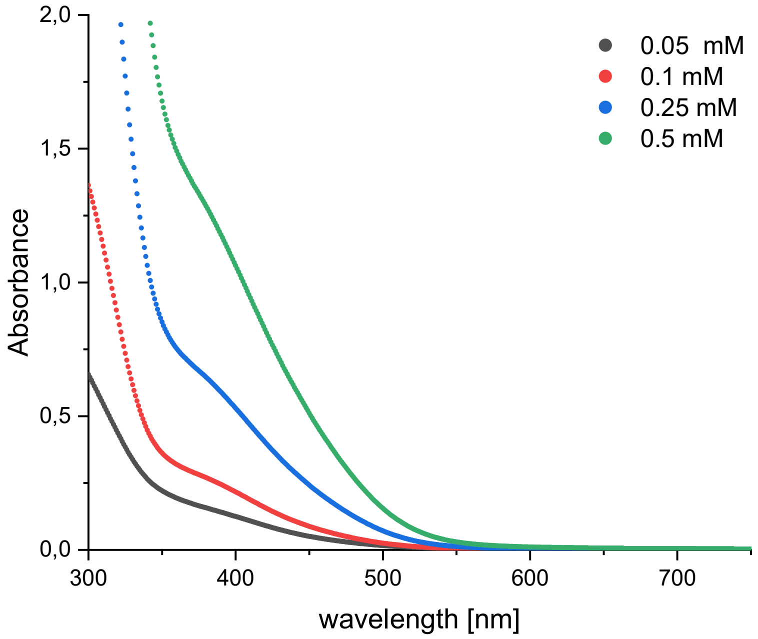

Figure S111: UV/Vis spectrum of **6AsSe** in THF for determined concentrations.

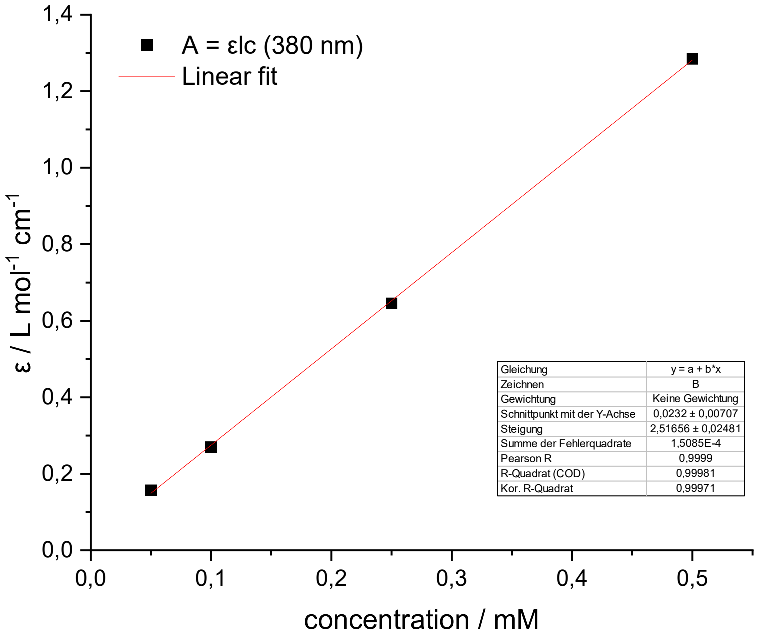

Figure S112: Concentration–absorbance dependence at 380 nm, absorption maximum of **6AsSe** in THF. Molar absorptivity in the presented range (ε_{380}= 2.517 cm^{-1} mol^{-1}) was determined using the Lambert–Beer law.

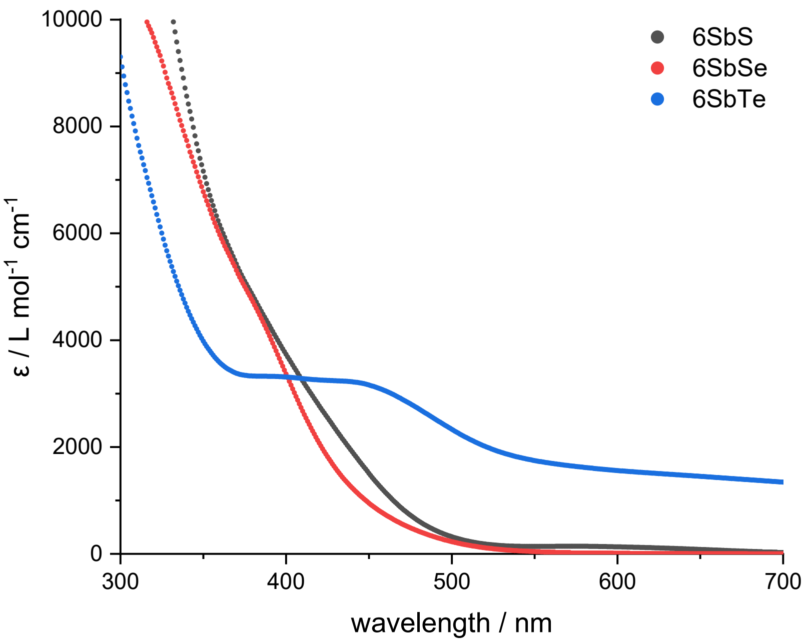

Figure S113: UV/Vis spectrum of **6SbS** (black), **6SbSe** (red) and **6SbTe** (blue) in THF as a direct comparison**.**

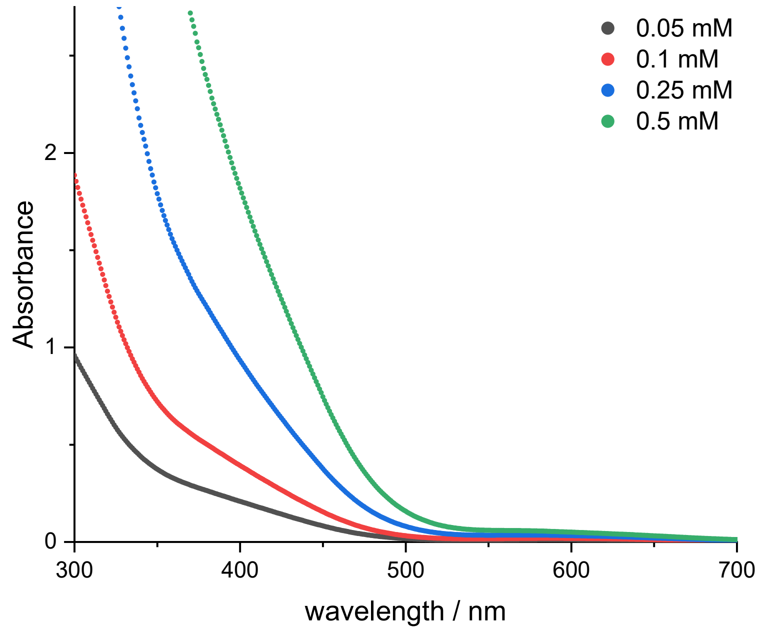

Figure S114: UV/Vis spectrum of **6SbS** in THF for determined concentrations.

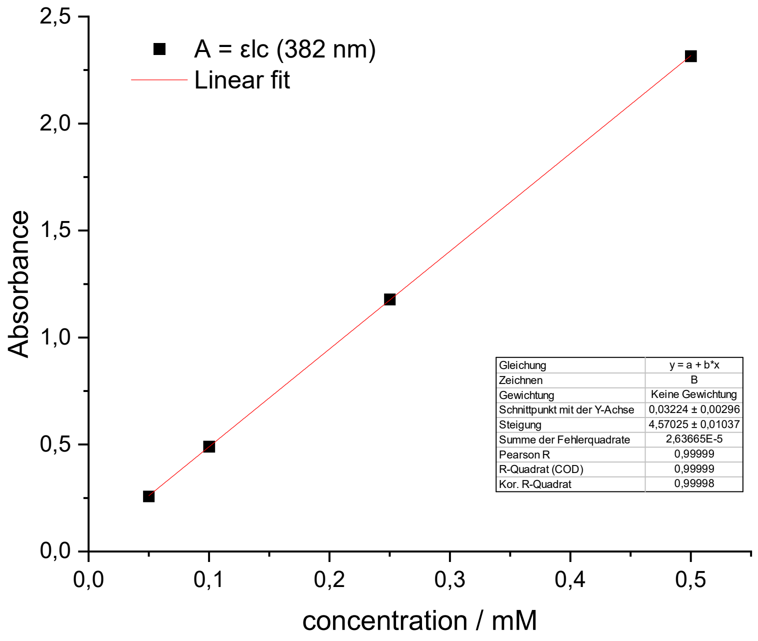

Figure S115: Concentration–absorbance dependence at 382 nm, absorption maximum of **6SbS** in THF. Molar absorptivity in the presented range (ε_{382}= 4.570 cm^{-1} mol^{-1}) was determined using the Lambert–Beer law.

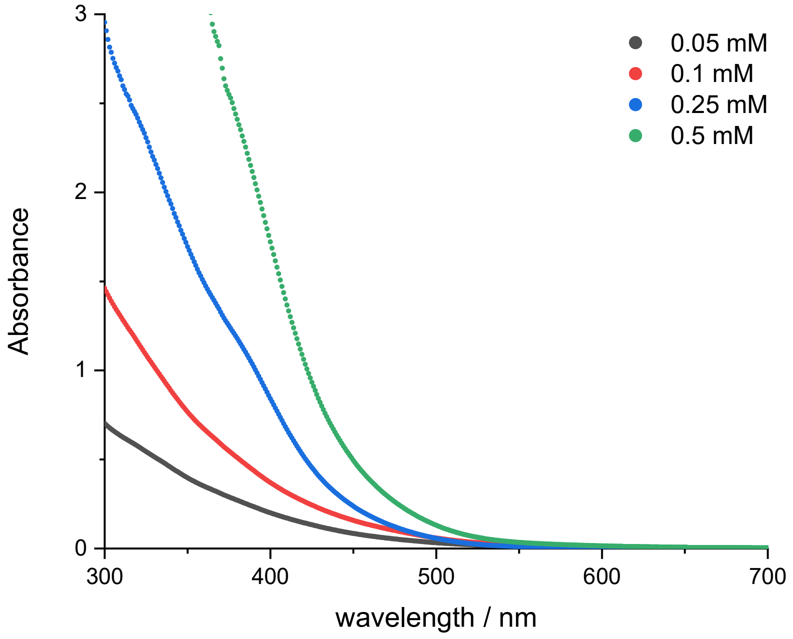

Figure S116: UV/Vis spectrum of **6SbSe** in THF for determined concentrations.

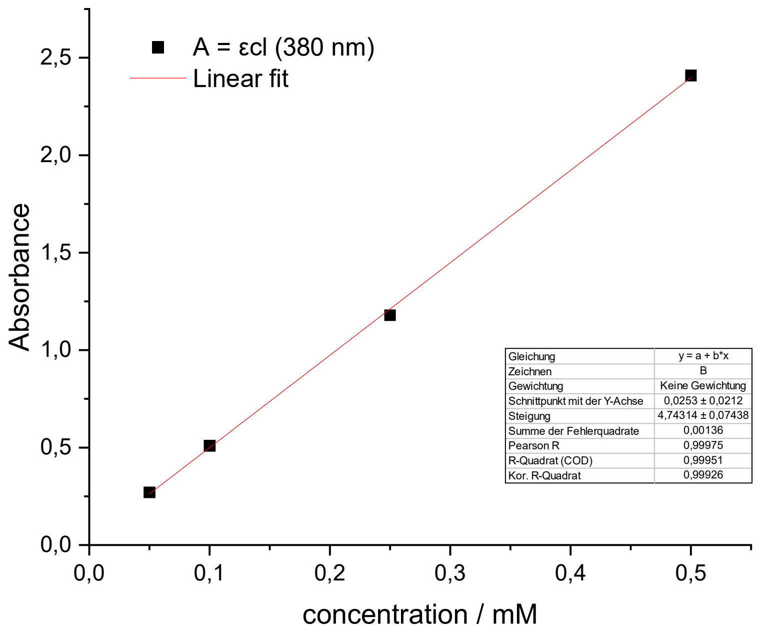

Figure S117: Concentration–absorbance dependence at 380 nm, absorption maximum of **6SbSe** in THF. Molar absorptivity in the presented range (ε_{380}= 4.743 cm^{-1} mol^{-1}) was determined using the Lambert–Beer law.

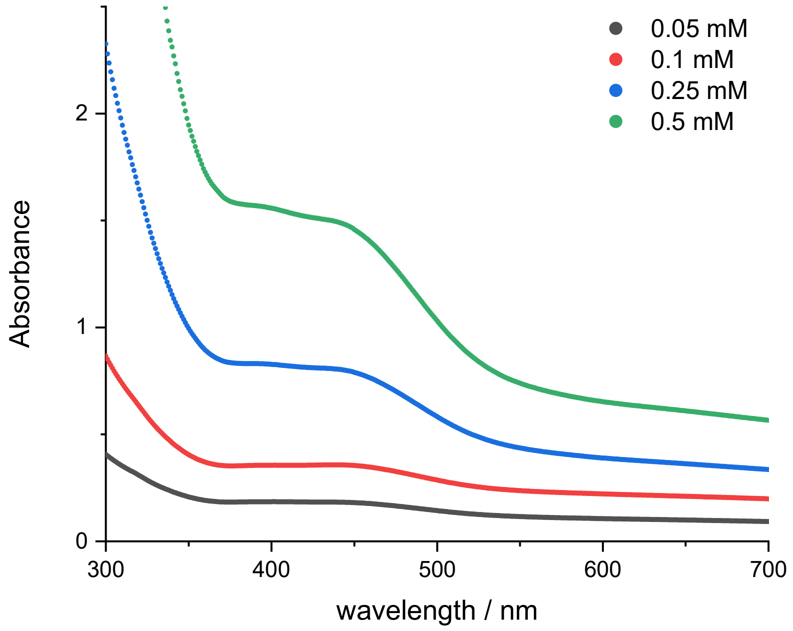

Figure S118: UV/Vis spectrum of **6SbTe** in THF for determined concentrations.

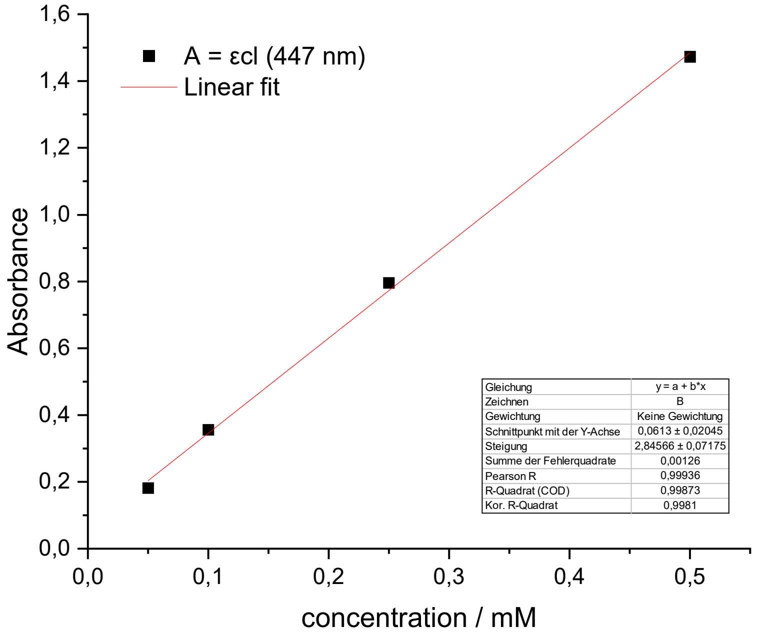

Figure S119: Concentration–absorbance dependence at 447 nm, absorption maximum of **6SbTe** in THF. Molar absorptivity in the presented range (ε_{447}= 2.846 cm^{-1} mol^{-1}) was determined using the Lambert–Beer law.

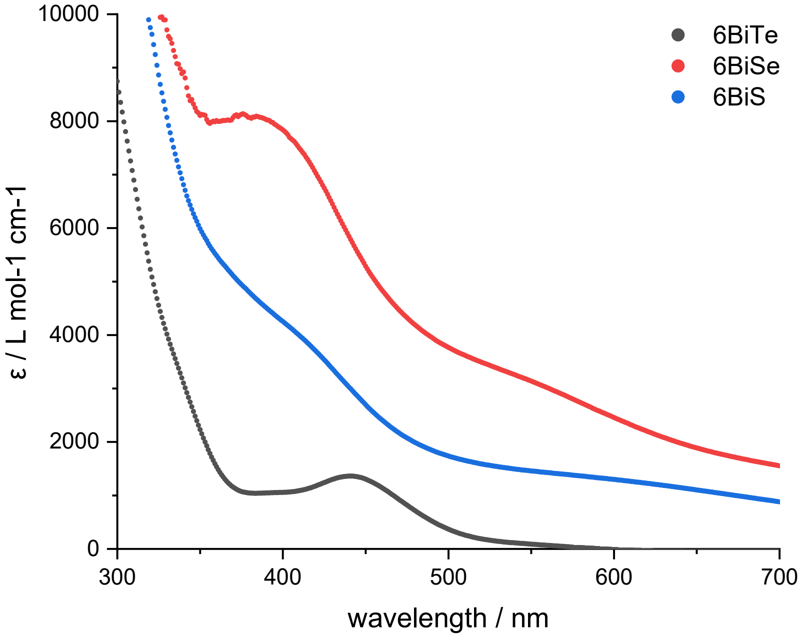

Figure S120: UV/Vis spectrum of **6BiS** (blue), **6BiSe** (red) and **6BiTe** (black) in THF as a direct comparison**.**

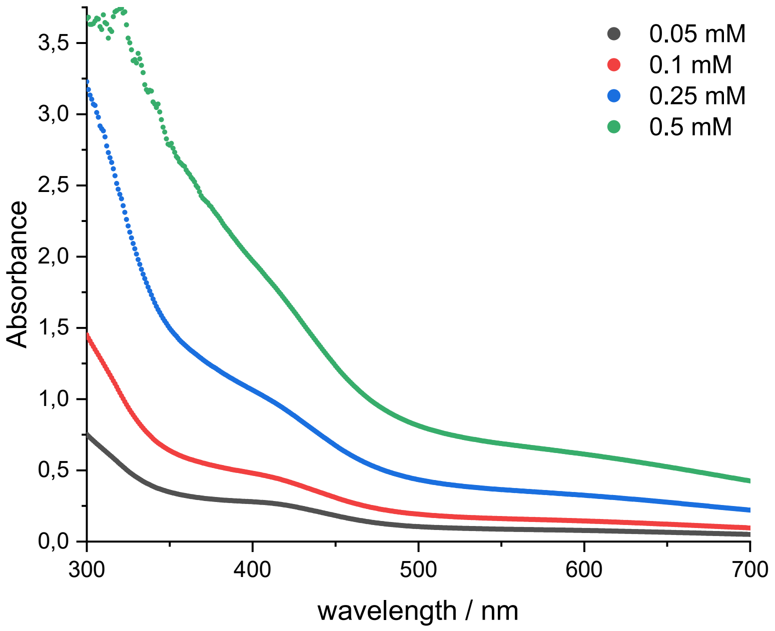

Figure S121: UV/Vis spectrum of **6BiS** in THF for determined concentrations.

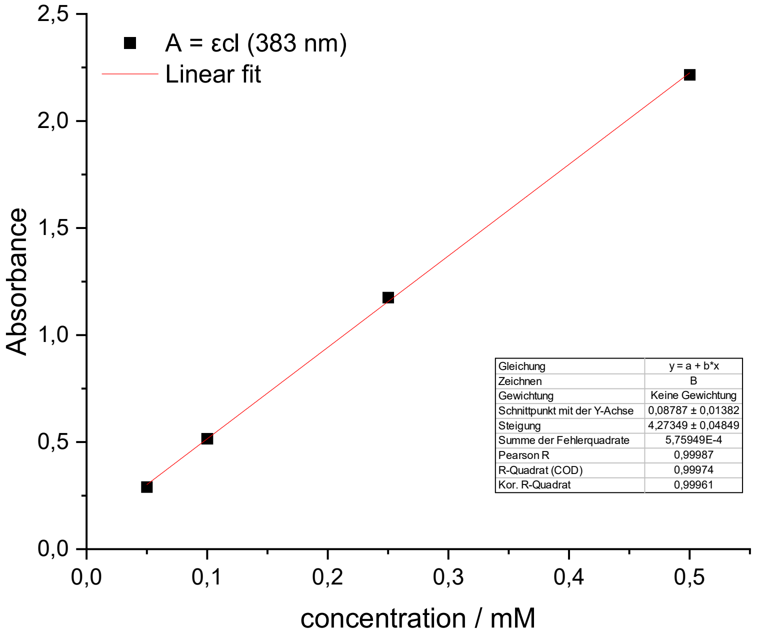

Figure S122: Concentration–absorbance dependence at 383 nm, absorption maximum of **6BiS** in THF. Molar absorptivity in the presented range (ε_{383}= 4.273 cm^{-1} mol^{-1}) was determined using the Lambert–Beer law.

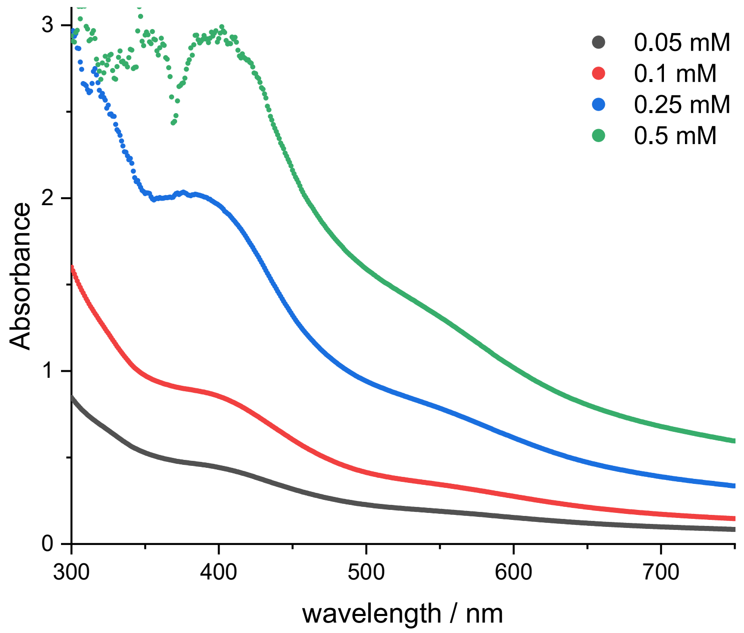

Figure S123: UV/Vis spectrum of **6BiSe** in THF for determined concentrations.

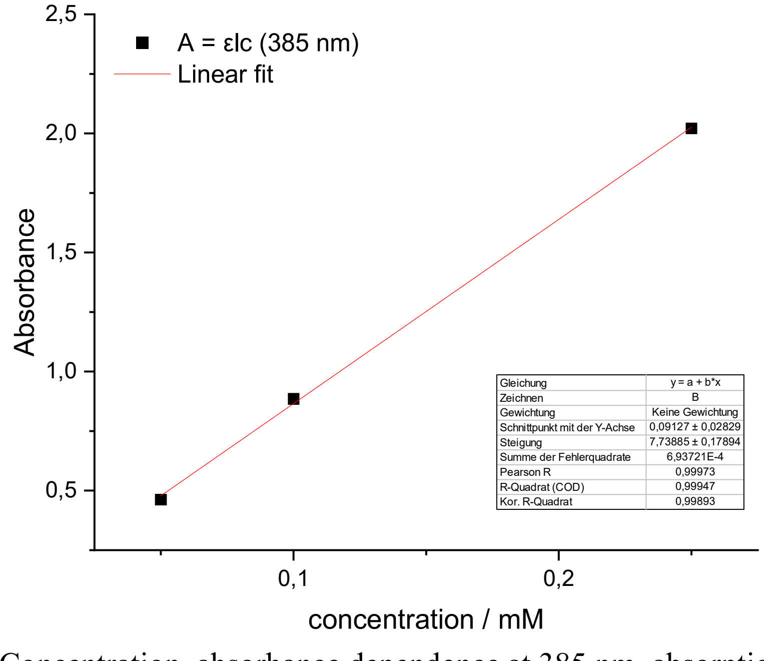

Figure S124: Concentration–absorbance dependence at 385 nm, absorption maximum of **6BiSe** in THF. Molar absorptivity in the presented range (ε_{385}= 7.739 cm^{-1} mol^{-1}) was determined using the Lambert–Beer law.

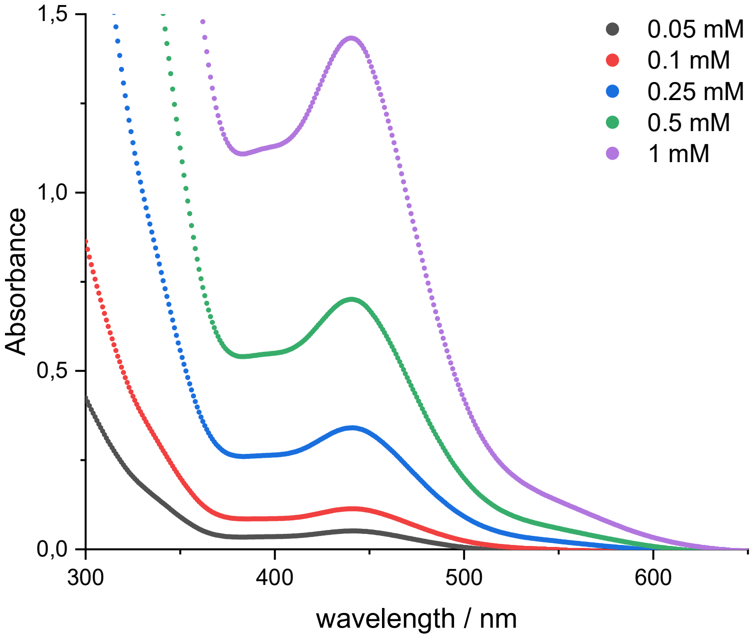

Figure S125: UV/Vis spectrum of **6BiTe** in THF for determined concentrations.

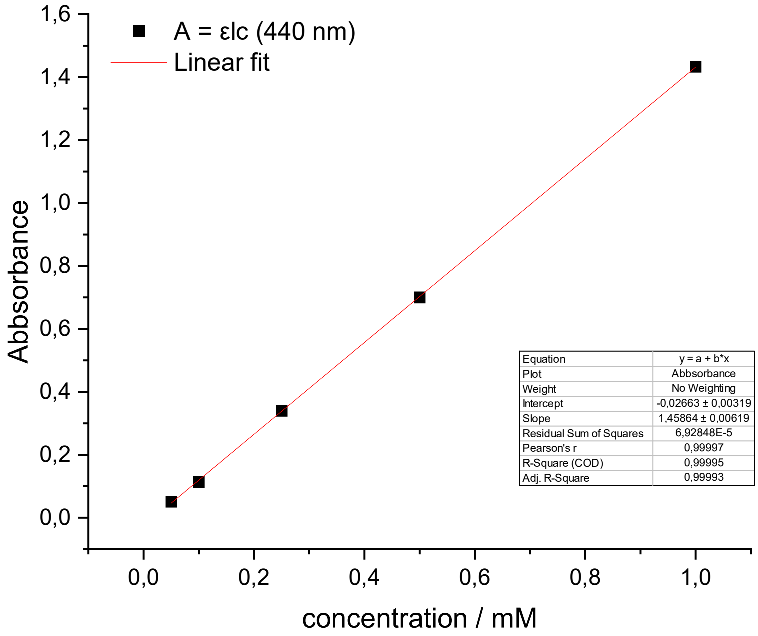

Figure S126: Concentration–absorbance dependence at 440 nm, absorption maximum of **6BiTe** in THF. Molar absorptivity in the presented range (ε_{440}= 1.458 cm^{-1} mol^{-1}) was determined using the Lambert–Beer law.

X-ray crystallography

Intensity data of **1**, **2**·Et_2O, **3Sb**·acetone, **3Bi**·0.5 CH_2Cl_2, [**4As**]$_2$[As_2OCl_5]·1.5 1,2-$C_6H_4F_2$, [**4As**][$AlCl_4$]·C_6H_{12}, [**4Sb**][$AlCl_4$]·THF, **5Sb**·$AlCl_3$·1.75 THF, **5As**·2.5 THF, **5Sb**·BH_3·2.5 THF, **6AsSe**·THF, **6SbSe**·THF, **6BiTe**·3 THF were collected at 100 K on a Bruker Venture D8 diffractometer with graphite-monochromated Mo-Kα (0.7107 Å) radiation. All structures were solved by direct methods and refined based on F^2 by use of the SHELX program package as implemented in OLEX 2 version 1.5.[58] All non-hydrogen atoms were refined using anisotropic displacement parameters. Hydrogen atoms attached to carbon atoms were included in geometrically calculated positions using a riding model. Crystal and refinement data are collected in Table S1. Figures were created using DIAMOND.[59] Crystallographic data for the structural analyses have been deposited with the Cambridge Crystallographic Data Centre, nos. 2446710 – 2446720, 2457652 and 2457653. Copies of this information may be obtained free of charge from The Director, CCDC, 12 Union Road, Cambridge CB2 1EZ, UK (Fax: +44-1223-336033; e-mail: deposit@ccdc.cam.ac.uk or http://www.ccdc.cam.ac.

Table S1. Crystals and refinement data.

	**1**	**2**·Et_2O	**3Sb**·acetone
Formula	$C_{55}H_{61}BrN_2P_2$	$C_{56}H_{63}ClMgN_2O_2P_2$	$C_{51}H_{51}Cl_2N_2OP_2Sb$
Formula weight, g mol^{-1}	891.90	917.78	962.53
Crystal system	monoclinic	triclinic	monoclinic
Crystal size, mm	0.20 × 0.20 × 0.18	0.35 × 0.28 × 0.090	0.12 × 0.08 × 0.08
Space group	$P2_1/n$	$P\bar{1}$	$P2_1/c$
a, Å	22.580(3)	12.1739(4)	10.682(4)
b, Å	12.2624(16)	12.6388(5)	18.781(9)
c, Å	16.199(2)	36.2781(12)	22.801(10)
α, °	90	88.6980(10)	90
β, °	94.048(5)	81.1000(10)	90.795(18)
γ, °	90	64.7300(10)	90
V, Å^3	4474.0(10)	4981.1(3)	4574(4)
Z	4	4	4
ρ_{calcd}, Mg m^{-3}	1.324	1.224	1.398
μ (Mo $K\alpha$), mm^{-1}	1.031	0.197	0.830
F(000)	1880	1952	1976
θ range, deg	2.09 to 25.01	1.87 to 25.01	1.94 to 29.63
Index ranges	$-26 \le h \le 26$	$-14 \le h \le 14$	$-17 \le h \le 17$
	$-14 \le k \le 14$	$-15 \le k \le 15$	$-30 \le k \le 30$
	$-19 \le l \le 19$	$-43 \le l \le 43$	$-37 \le l \le 37$
No. of reflns collected	271538	119273	131308
Completeness to θ_{max}	99.9%	99.9%	99.9%
No. indep. Reflns	7880	17525	21115
No. obsd reflns with($I>2\sigma(I)$)	7040	14068	17645
No. refined params	484	1187	540
GooF (F^2)	1.072	1.062	1.064
R_1 (F) ($I > 2\sigma(I)$)	0.0350	0.0472	0.0410
wR_2 (F^2) (all data)	0.1000	0.1312	0.0817
Largest diff peak/hole, e Å^{-3}	1.071 / –0.272	0.911 / –0.567	0.640 / –1.011
CCDC number	2446710	2446711	2446712

Table S1. cont.

**3Bi**·0.5 CH_2Cl_2	**4As**(As_2OCl_5)·1.5 $C_6H_4F_2$	**[4As]**($AlCl_4$)·C_6H_{12}	**[4Sb]**($AlCl_4$)·THF
$C_{48.5}H_{46}BiCl_3N_2P_2$	$C_{57}H_{51}As_3Cl_6F_3N_2OP_2$	$C_{54}H_{59}AlAsCl_5N_2P_2$	$C_{56}H_{61}AlCl_5N_2O_2P_2Sb$
1034.14	1336.40	1077.12	1181.98
monoclinic	triclinic	monoclinic	orthorhombic
0.16 × 0.13 × 0.11	0.19 × 0.18 × 0.17	0.21 × 0.18 × 0.16	0.41 × 0.15 × 0.15
$P2_1/n$	$P\bar{1}$	$P2_1/c$	$P2_12_12_1$
16.2659(14)	12.9688(9)	14.8002(7)	11.017(5)
19.0440(15)	18.3595(12)	19.5276(9)	18.037(8)
16.1671(14)	26.9379(16)	18.1091(8)	27.534(12)
90	106.082(2)	90	90
110.717(3)	93.732(2)	91.085(2)	90
90	105.763(2)	90	90
4684.2(7)	5862.5(7)	5232.8(4)	5472(4)
4	4	4	4
1.466	1.514	1.367	1.435
4.037	2.078	1.021	0.866
2060	2692	2232	2424
2.14 to 33.14	1.94 to 30.56	2.25 to 27.5	1.99 to 33.23
–25 ≤ h ≤ 25	–18 ≤ h ≤ 18	–19≤ h ≤ 19	–13 ≤ h ≤ 14
–29 ≤ k ≤ 29	–26 ≤ k ≤ 26	–25 ≤ k ≤ 25	–23 ≤ k ≤ 22
–24 ≤ l ≤ 24	–38 ≤ l ≤ 38	–23 ≤ l ≤ 23	–35 ≤ l ≤ 30
184813	218773	105461	37373
99.8%	99.9%	99.9%	99.9%
17844	35882	12019	11987
13978	23282	9934	10165
502	1201	538	523
1.053	1.022	1.061	1.031
0.0336	0.0451	0.0587	0.0527
0.0868	0.1150	0.1732	0.1021
1.040 / –0.872	1.635 / –0.647	1.986 / –1.367	1.275 / –0.889
2446713	2446714	2446715	2446716

Table S1. cont.

**5As**·2.5 THF	**5Sb**·$AlCl_3$·1.75 THF	**5Sb**·BH_3·2.5 THF	**6AsSe**·THF
$C_{58}H_{65}AsN_2O_{2.5}P_2$	$C_{55}H_{59}AlCl_3N_2O_{1.75}P_2Sb$	$C_{58}H_{68}BN_2O_{2.50}P_2Sb$	$C_{52}H_{53}AsN_2OP_2Se$
967.04	1093.06	1027.64	937.78
monoclinic	orthorhombic	monoclinic	monoclinic
0.16 × 0.13 × 0.11	0.16 × 0.13 × 0.11	0.45 × 0.35 × 0.29	0.20 × 0.17 × 0.14
C2/c	Pbca	C2/c	$P2_1/n$
27.619(5)	24.7633(6)	37.562(2)	11.0936(8)
18.001(3)	16.3853(4)	13.2376(6)	17.8887(15)
25.520(4)	26.2160(4)	20.6432(11)	22.944(2)
90	90	90	90
98.327(5)	90	104.313(2)	101.146(3)
90	90	90	90
12554(4)	10637.2(4)	9945.8(9)	4467.4(6)
8	8	8	4
1.023	1.365	1.373	1.394
0.629	0.787	0.666	1.688
4085	4496	4288	1936
2.02 to 25.00	2.06 to 27.50	1.94 to 30.56	2.14 to 27.50
–36 ≤ h ≤ 36	–32 ≤ h ≤ 28	–44≤ h ≤ 44	–14≤ h ≤ 14
–24 ≤ k ≤ 24	–21 ≤ k ≤ 19	–15 ≤ k ≤ 14	–23 ≤ k ≤ 23
–34 ≤ l ≤ 34	–33 ≤ l ≤ 34	–24 ≤ l ≤ 24	–29 ≤ l ≤ 29
150289	66916	113700	92526
99.6%	99.9%	99.9%	99.9%
11029	12226	8819	10267
8200	9395	7060	8149
484	620	505	538
1.004	1.084	1.039	1.047
0.0638	0.0573	0.0378	0.0390
0.1879	0.1379	0.1154	0.0925
0.929 / –0.643	1.216 / –0.887	0.467 / –0.542	0.643 / –0.540
2457652	2446717	2446718	2457653

Table S1. cont.

**6SbSe**·THF	**6BiTe**·3 THF
$C_{52}H_{53}N_2OP_2SbSe$	$C_{60}H_{69}BiN_2O_3P_2Te$
984.61	1264.69
monoclinic	monoclinic
0.21 × 0.18 × 0.16	0.35 × 0.35 × 0.05
$P2_1/n$	$P2_1/n$
11.0961(3)	10.1844(7)
18.1134(4)	15.2867(12)
22.9780(6)	34.707(2)
90	90
101.2820(10)	97.585(2)
90	90
4529.1(2)	5356.1(7)
4	4
1.444	1.568
1.524	3.932
2008	2528
2.23 to 28.52	2.03 to 25.03
$-14 \leq h \leq 14$	$-12 \leq h \leq 12$
$-24 \leq k \leq 24$	$-18 \leq k \leq 18$
$-30 \leq l \leq 30$	$-41 \leq l \leq 41$
127829	171563
99.8%	99.9%
11479	9450
10739	8574
538	637
1.027	1.133
0.0208	0.0340
0.0517	0.0716
0.386 / –0.411	0.884 / –1.406
2446719	2446720

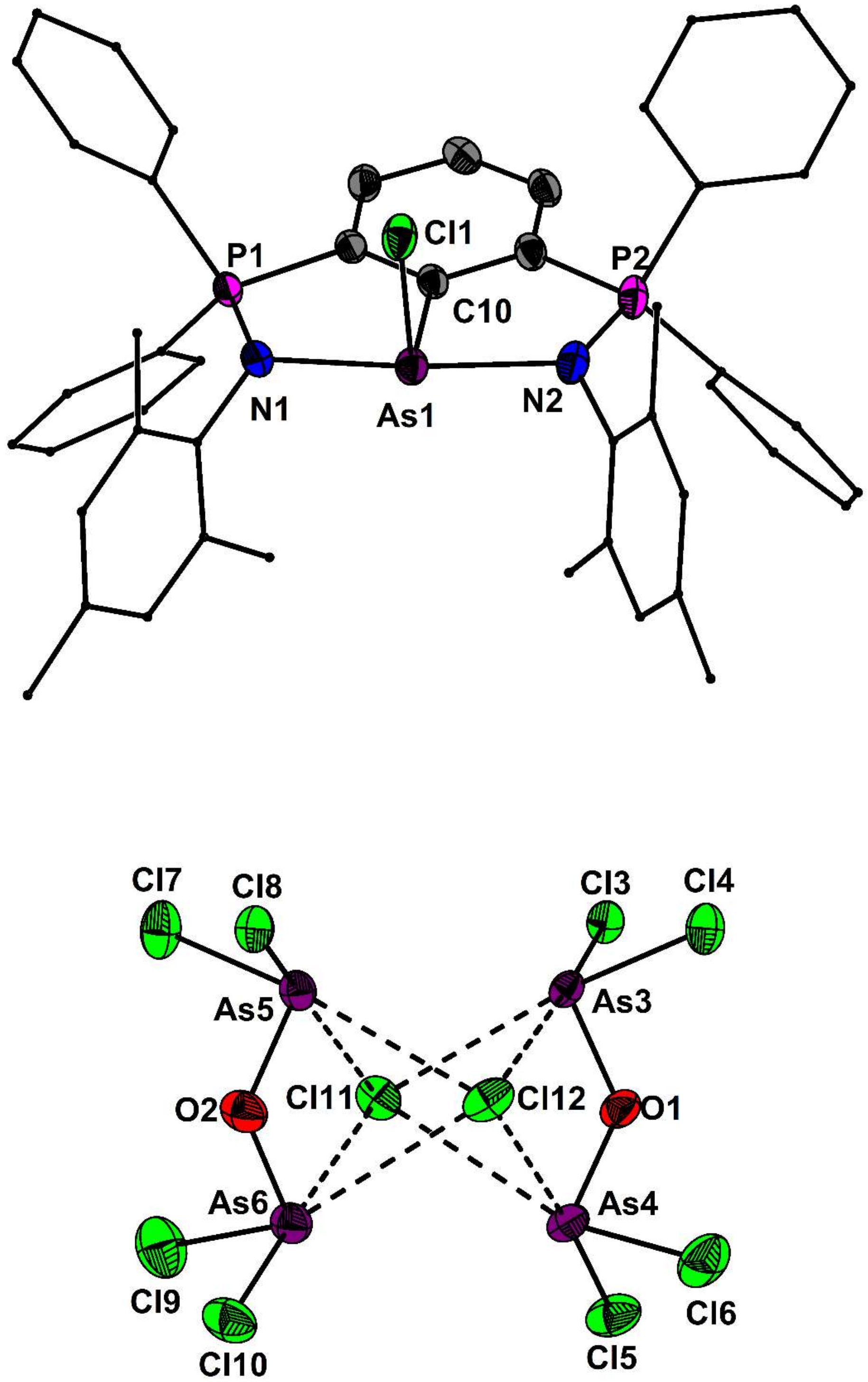

Figure S 127: Molecular structure of [**4As**][As_2OCl_5]·showing 50% probability ellipsoids and the atomic numbering scheme.

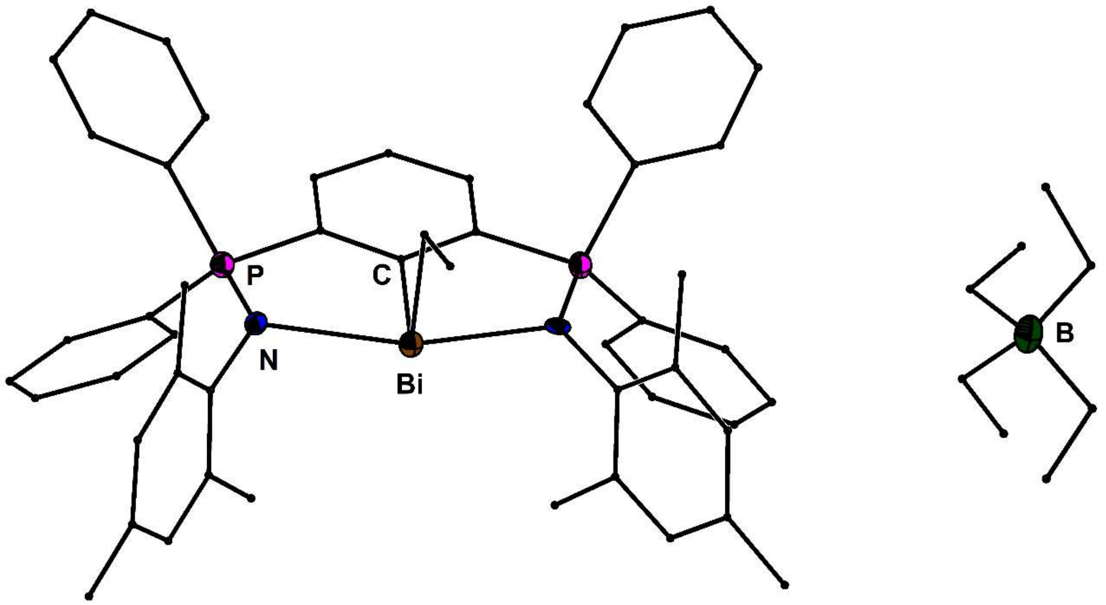

Figure S128: Preliminary molecular structure of [2,6-($Ph_2PNMes)_2C_6H_3BiEt$][Et_4B] showing 50% probability ellipsoids.

DFT Computations

Computational methodology. Geometry Optimizations of the isolated molecule structures were carried out using density functional theory (DFT) at the B3PW91/6-311+G(2df,p)[60,61] level of theory using the Gaussian16 software package.[62] For Se, Sb, Te and Bi atoms the cc-pVTZ-PP[63] basis set were used with effective core potentials accounting for 10 (Se), 28 (Sb and Te) and 60 electrons (Bi), respectively. Basis sets were obtained from the Basis Set Exchange Library.[64] Dispersion effects were modelled using Grimme's GD3BJ parameters.[65] Subsequent frequency analysis confirmed all structures to be local minima on the potential energy surface. The wavefunction files of the optimized structures were used for a topological analysis of the electron density according to the Atoms-In-Molecules partitioning scheme[50] using AIMAll.[66] The NCI[52] grids were computed with NCIplot.[67] NBO/NLMO[55] analysis was performed using the NBO6 software.[68] Independent gradient model based on Hirshfeld partition (IGMH)[69] analysis were obtained with Multiwfn_3.8.[70] Figures are displayed using VMD.[71] EDA-NOCV[56] analysis were performed using the Amsterdam Modeling Suite ADF2020.102[72] using the B3PW91 functional along with a TZ2P[73] basis set and Grimme's D4 dispersion effects.[74] The percentage of buried volume have been obtained using the SambVca 2.1 A web application.[75] For the analysis of the localized orbital locator of the π-orbitals (LOL-π)[57] an orbital localization analysis was performed according to the Pipek-Mezey method[76] using Orca 6.0.1[77] with the B3WP91/def2-TZVP[78] level of theory and Grimme's GD3BJ dispersion. Then, Multiwfn was used to detect the π-orbitals and to set the occupation of all other orbitals, except the π-orbitals, to zero.

Table S2. Topological bond properties from AIM analysis of **6AsCh** (Ch = S, Se, Te).

Species	d [Å]	ρ(r) [eÅ^{-3}]	∇^2ρ(r) [eÅ^{-5}]	ε	G/ρ(r) [a.u.]	H/ρ(r) [a.u.]
**6AsS**						
As1-S2	2.147	0.86	–1.4	0.01	0.45	–0.56
As1-C47	2.008	0.87	–1.5	0.12	0.51	–0.62
As1-N6	2.242	0.45	2.2	0.06	0.61	–0.27
As1-N5	2.231	0.46	2.2	0.06	0.62	–0.28
As1-H34	2.904	0.06	0.6	0.82	0.58	0.09
**6AsSe**						
As1-Se2	2.300	0.72	–1.1	0.02	0.37	–0.48
As1-C47	1.997	0.89	–1.5	0.14	0.51	–0.63
As1-N6	2.230	0.46	2.2	0.07	0.62	–0.28
As1-N5	2.227	0.46	2.2	0.08	0.62	–0.29
As1-H34	2.847	0.07	0.6	0.57	0.57	0.08
**6AsTe**						
As1-Te2	2.536	0.56	–0.3	0.03	0.35	–0.40
As1-C47	1.984	0.91	–1.5	0.16	0.53	–0.64
As1-N6	2.217	0.47	2.2	0.09	0.62	–0.30
As1-N5	2.217	0.47	2.2	0.09	0.62	–0.30

Table S3. Topological bond properties from AIM analysis of **6SbCh** (Ch = S, Se, Te).

**Species**	**d** [Å]	**ρ(r)** [eÅ^{-3}]	**∇^{2}ρ(r)** [eÅ^{-5}]	**ε**	**G/ρ(r)** [a.u.]	**H/ρ(r)** [a.u.]
**6SbS**						
Sb1-S2	2.315	0.70	1.1	0.00	0.57	–0.46
Sb1-C47	2.214	0.67	1.7	0.10	0.62	–0.44
Sb1-N6	2.366	0.40	2.9	0.07	0.72	–0.21
Sb1-N5	2.366	0.40	2.9	0.07	0.72	–0.21
Sb1-H34	3.042	0.06	0.5	2.36	0.56	0.08
Sb1-H70	3.042	0.06	0.5	2.13	0.55	0.08
**6SbSe**						
Sb1-Se2	2.453	0.61	0.3	0.01	0.45	–0.42
Sb1-C47	2.208	0.68	1.8	0.11	0.63	–0.44
Sb1-N6	2.366	0.40	2.9	0.08	0.72	–0.21
Sb1-N5	2.364	0.40	2.9	0.08	0.72	–0.21
Sb1-H34	2.998	0.06	0.5	0.55	0.52	0.07
**6SbTe**						
Sb1-Te2	2.674	0.50	–0.2	0.01	0.35	–0.38
Sb1-C47	2.199	0.69	1.8	0.13	0.63	–0.45
Sb1-N6	2.362	0.40	2.9	0.09	0.72	–0.21
Sb1-N5	2.363	0.40	2.9	0.09	0.72	–0.21
Sb1-H34	2.933	0.07	0.5	0.33	0.51	0.05

Table S4. Topological bond properties from AIM analysis of **6BiCh** (Ch = S, Se, Te).

Species	d [Å]	ρ(r) [eÅ^{-3}]	$\nabla^2\rho(r)$ [eÅ^{-5}]	ε	G/ρ(r) [a.u.]	H/ρ(r) [a.u.]
**6BiS**						
Bi1-S2	2.395	0.65	1.9	0.01	0.59	–0.40
Bi1-C47	2.317	0.61	2.1	0.09	0.61	–0.37
Bi1-N6	2.463	0.36	3.3	0.08	0.77	–0.13
Bi1-N5	2.463	0.36	3.3	0.08	0.77	–0.13
**6BiSe**						
Bi1-Se2	2.527	0.57	1.1	0.01	0.50	–0.36
Bi1-C47	2.313	0.61	2.1	0.10	0.61	–0.37
Bi1-N6	2.462	0.36	3.3	0.09	0.77	–0.13
Bi1-N5	2.462	0.36	3.3	0.09	0.77	–0.13
Bi1-H34	3.089	0.05	0.5	2.45	0.57	0.09
**6BiTe**						
Bi1-Te2	2.739	0.47	0.4	0.00	0.40	–0.34
Bi1-C47	2.305	0.62	2.2	0.11	0.61	–0.37
Bi1-N6	2.459	0.36	3.3	0.09	0.77	–0.13
Bi1-N5	2.463	0.36	3.3	0.10	0.77	–0.13
Bi1-H34	3.014	0.06	0.5	0.61	0.53	0.07

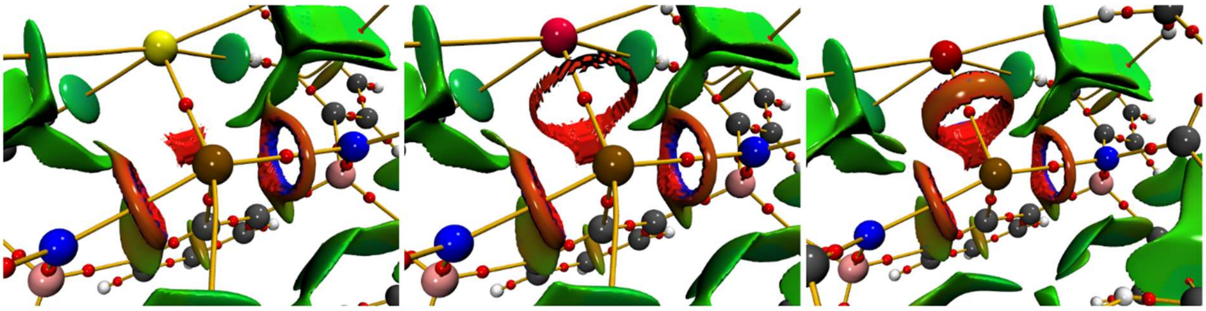

Figure S129: AIM molecular graphs of **6AsS** (left), **6AsSe** (middle) and **6AsTe** (right) with bond critical points as red spheres and bond paths in orange as well as NCI *iso*-surfaces at s(r) = 0.5 colour coded with sign(λ2)ρ in a. u. Blue surfaces refer to attractive forces and red to repulsive forces. Green indicates weak interactions.

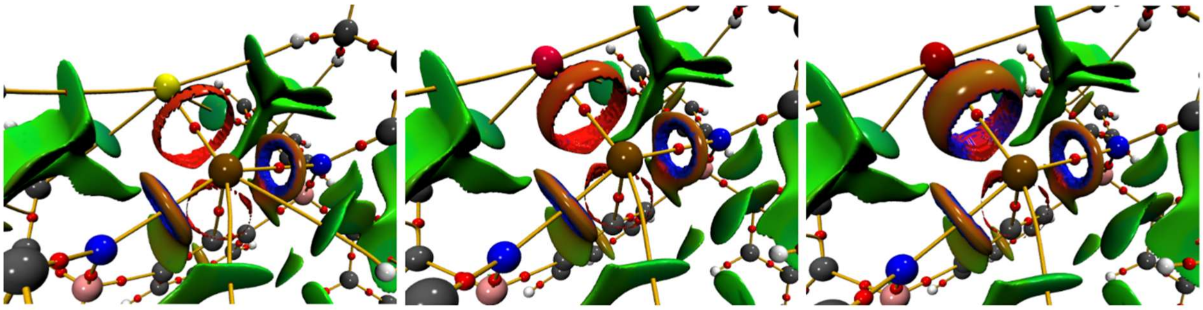

Figure S130: AIM molecular graphs of **6SbS** (left), **6SbSe** (middle) and **6SbTe** (right) with bond critical points as red spheres and bond paths in orange as well as NCI *iso*-surfaces at s(r) = 0.5 colour coded with sign(λ2)ρ in a. u. Blue surfaces refer to attractive forces and red to repulsive forces. Green indicates weak interactions.

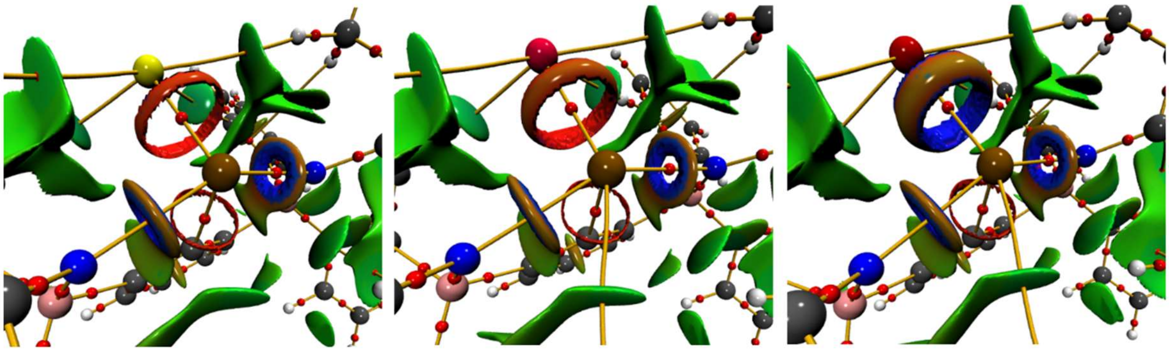

Figure S131: AIM molecular graphs of **6BiS** (left), **6BiSe** (middle) and **6BiTe** (right) with bond critical points as red spheres and bond paths in orange as well as NCI iso-surfaces at s(r) = 0.5 colour coded with sign(λ2)ρ in a. u. Blue surfaces refer to attractive forces and red to repulsive forces. Green indicates weak interactions.

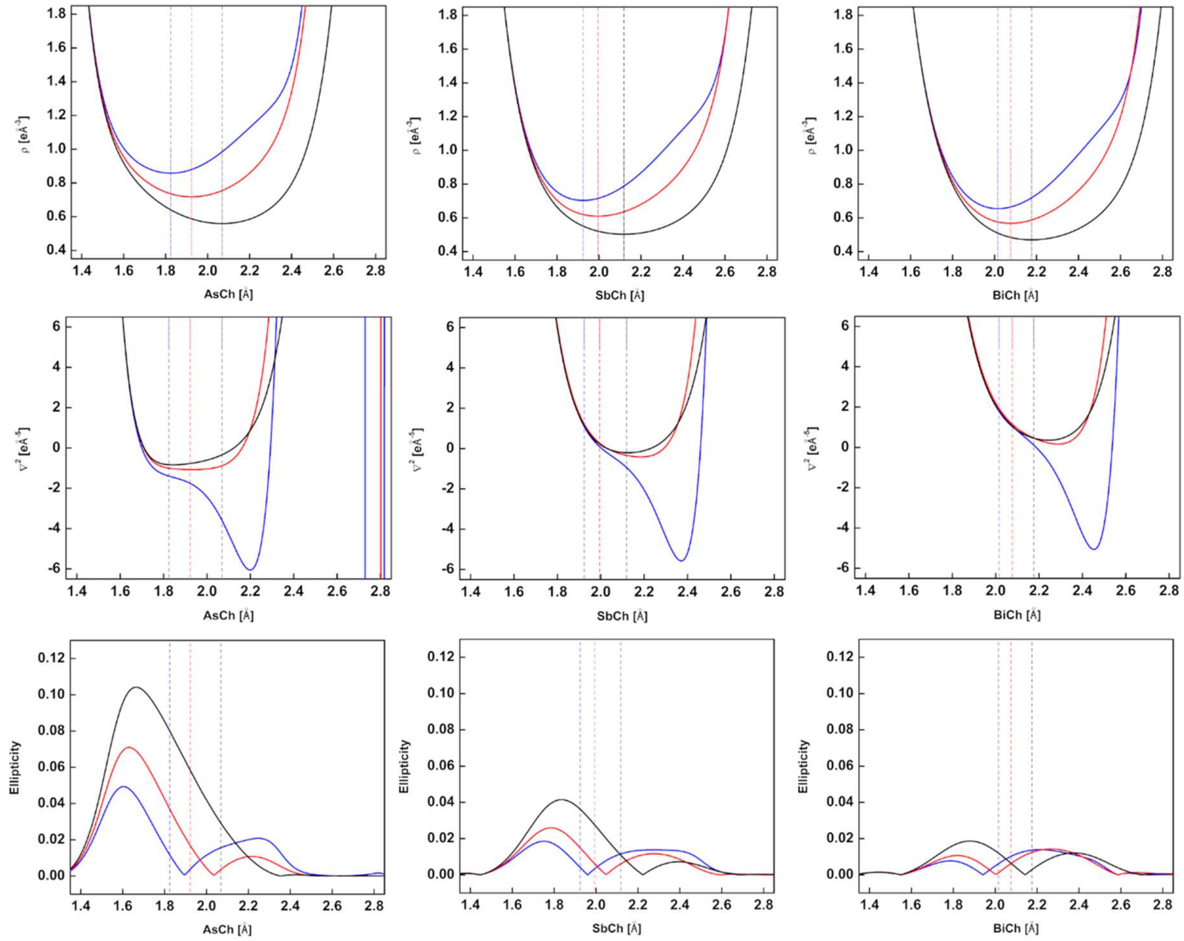

Figure S132. Electron density (top) and Laplacian of the electron density (middle) and ellipticity (bottom) of **6AsCh** (left), **6SbCh** (middle) and **6BiCh** (right) along the Pn–Ch bond axis with S represented in blue, Se in red and Te in black. The positions of the Pn–Ch bond critical points are indicated by the dashed vertical lines in the respective color. The pnictogen is located at 0.794 Å (1.5 bohr).

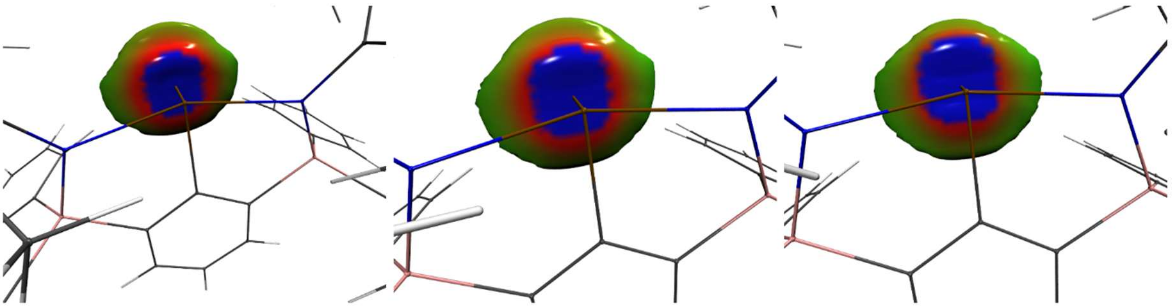

Figure S133: IGM based on a Hirshfeld partition of the molecular density of **6AsS** (left), **6AsSe** (middle) and **6AsTe** (right). Fragment 1 is the Sb-atom and fragment 2 the respective chalcogen. IGMH *iso*-surfaces at s(r) = 0.005 colour coded with sign(λ2)ρ in a. u. Blue surfaces refer to attractive forces and red to repulsive forces. Green indicates weak interactions.

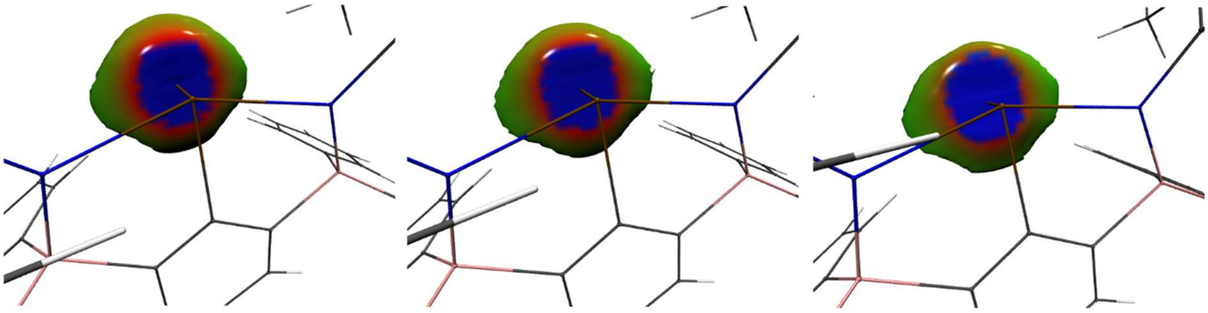

Figure S134: IGM based on a Hirshfeld partition of the molecular density of **6SbS** (left), **6SbSe** (middle) and **6SbTe** (right). Fragment 1 is the Sb-atom and fragment 2 the respective chalcogen. IGMH *iso*-surfaces at s(r) = 0.005 colour coded with sign(λ2)ρ in a. u. Blue surfaces refer to attractive forces and red to repulsive forces. Green indicates weak interactions.

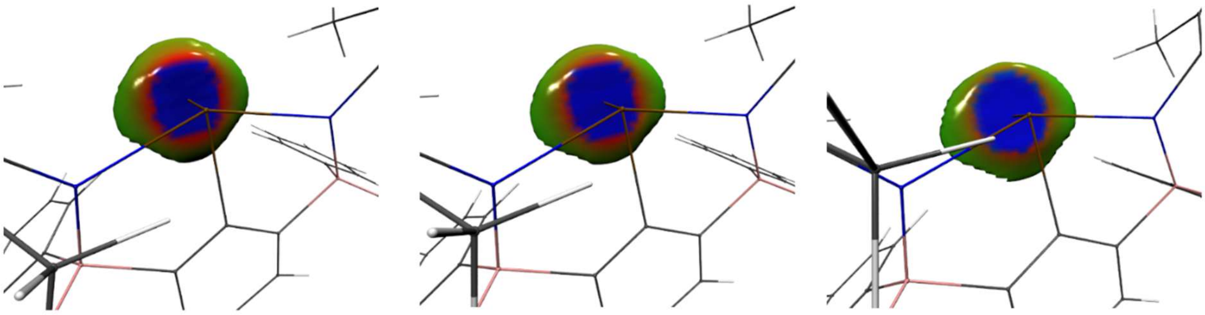

Figure S135: IGM based on a Hirshfeld partition of the molecular density of **6BiS** (left), **6BiSe** (middle) and **6BiTe** (right). Fragment 1 is the Bi-atom and fragment 2 the respective chalcogen. IGMH *iso*-surfaces at s(r) = 0.005 colour coded with sign(λ2)ρ in a. u. Blue surfaces refer to attractive forces and red to repulsive forces. Green indicates weak interactions.

Table S5. Selected delocalization indices (δ), Wiberg Bond Index (WBI) and NLMO/NPA bond orders of **6AsCh**, **6SbCh** and **6BiCh** (Ch = S, Se, Te).

Species	δ	WBI	NLMO/ NPA	δ	WBI	NLMO/ NPA	δ	WBI	NLMO /NPA
		**6AsS**			**6SbS**			**6BiS**	
Ch1-S2	1.28	1.23	1.01	1.24	1.19	0.89	1.29	1.21	0.90
Ch1-C47	0.78	0.75	0.56	0.69	0.63	0.45	0.69	0.61	0.44
Ch1-N6	0.44	0.29	0.16	0.42	0.26	0.14	0.40	0.23	0.12
Ch1-N5	0.45	0.30	0.19	0.42	0.26	0.15	0.40	0.23	0.12
		**6AsSe**			**6SbSe**			**6BiSe**	
Ch1-Se2	1.26	1.19	1.07	1.26	1.21	0.96	1.30	1.24	0.97
Ch1-C47	0.80	0.77	0.57	0.71	0.64	0.45	0.70	0.62	0.44
Ch1-N6	0.46	0.30	0.17	0.43	0.26	0.15	0.40	0.23	0.12
Ch1-N5	0.46	0.31	0.19	0.43	0.26	0.15	0.40	0.23	0.12
		**6AsTe**			**6SbTe**			**6BiTe**	
Ch1-Te2	1.19	1.11	0.97	1.25	1.20	1.06	1.29	1.25	1.07
Ch1-C47	0.84	0.80	0.60	0.73	0.66	0.46	0.72	0.63	0.45
Ch1-N6	0.48	0.32	0.19	0.44	0.27	0.15	0.41	0.24	0.13
Ch1-N5	0.48	0.32	0.19	0.44	0.27	0.15	0.41	0.24	0.14

Table S6. Selected AIM and NPA derived atomic charges of **6SbCh** and **6BiCh** (Ch = S, Se, Te).

q	Pn	Ch	N/N	Pn	Ch	N/N	Pn	Ch	N/N
		**6AsS**			**6SbS**			**6BiS**	
q_{AIM}	1.07	–0.84	–1.67/ –1.68	1.32	–0.96	–1.70	1.25	–0.92	–1.69
q_{NPA}	0.98	–0.78	–1.14	1.26	–0.93	–1.18	1.31	–0.95	–1.17
		**6AsSe**			**6SbSe**			**6BiSe**	
q_{AIM}	0.95	–0.70	–1.67	1.21	–0.84	–1.69/ –1.70	1.16	–0.82	–1.69
q_{NPA}	0.91	–0.70	–1.14	1.19	–0.85	–1.18	1.23	–0.87	–1.17
		**6AsTe**			**6SbTe**			**6BiTe**	
q_{AIM}	0.81	–0.54	–1.67	1.08	–0.69	–1.69	1.05	–0.70	–1.69
q_{NPA}	0.80	–0.58	–1.14	1.08	–0.72	–1.18/ –1.17	1.11	–0.75	–1.17

Table S7. NBO analysis of selected bonds of **6AsCh** (Ch = S, Se, Te) with respective hybrids.

**Model**	**Occupation**	**Model**	**Occupation**	**Model**	**Occupation**
**6AsS**		**6AsSe**		**6AsTe**	
**LP(1) As**	1.96	**LP(1) As**	1.95	**LP(1) As**	1.95
**LP(1) S**	1.98	**LP(1) Se**	1.98	**LP(1) Te**	1.98
**LP(2) S**	1.87	**LP(2) Se**	1.87	**LP(2) Te**	1.88
**LP(3) S**	1.80	**LP(3) Se**	1.81	**LP(3) Te**	1.83

**Model**	**Occupation**	**Atom 1**	**Hybrid**	**Atom 2**	**Hybrid**
**BD(1) As-S**	1.95	As (43.25 %)	$sp^{7.11}d^{0.02}$	S (56.75 %)	$sp^{6.28}d^{0.08}$
**BD(1) As-C**	1.96	As (29.45 %)	$sp^{7.82}d^{0.04}$	C (70.55 %)	$sp^{2.54}$
**BD(1) As-Se**	1.94	As (47.95 %)	$sp^{8.05}d^{0.03}$	Se (52.05 %)	$sp^{9.69}d^{0.07}$
**BD(1) As-C**	1.96	As (29.57 %)	$sp^{7.60}d^{0.04}$	C (70.43 %)	$sp^{2.51}$
**BD(1) As-Te**	1.92	As (54.66 %)	$sp^{9.47}d^{0.05}$	Te (45.34 %)	$sp^{13.44}d^{0.09}$
**BD(1) As-C**	1.96	As (29.77 %)	$sp^{7.28}d^{0.05}$	C (70.23 %)	$sp^{2.47}$

**Donor NBO**	**Acceptor NBO**	**E2 [kcal/mol]**
LP(3) S	LV(1) As	47.73
LP(2) S	LV(1) C	16.03
LP(2) S	BD*(1) As-C	9.44
LP(3) Se	LV(1) As	39.41
LP(2) Se	LV(1) C	20.10
LP(2) Se	BD*(1) As-C	7.30
LP(3) Te	LV(1) As	28.77
LP(2) Te	LV(1) C	29.13
LP(2) Te	BD*(1) As-C	5.26

Table S8. Natural Localized Molecular Orbital (NLMO) Analysis of **6AsS**.

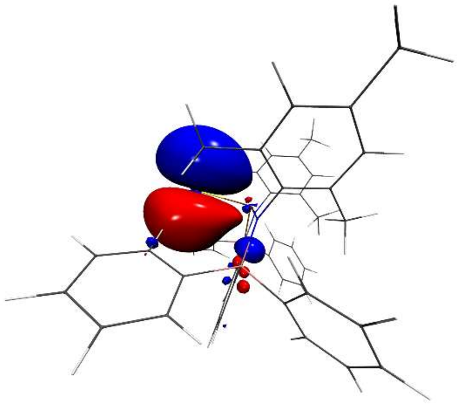
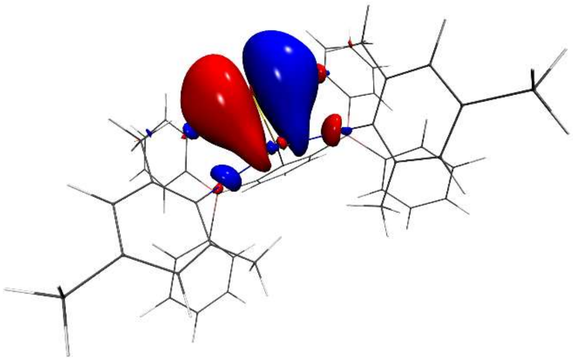

**NLMO 82**	**NLMO 83**
93.28 % of LP(2) S	90.06 % of LP(3) S
**Atomic Hybrid Contribution (with contributions > 1.00 %)**	
2.81 % Sb	5.93 % Sb
93.31 % S	90.06 % S
1.93 % C47	
**NLMO/NPA Bonder (% of total NLMO/NPA Bond order of 1.0095)**	
0.0561 (5.6 %)	0.1187 (11.8 %)

Table S8 cont. Natural Localized Molecular Orbital (NLMO) Analysis of **6AsSe**.

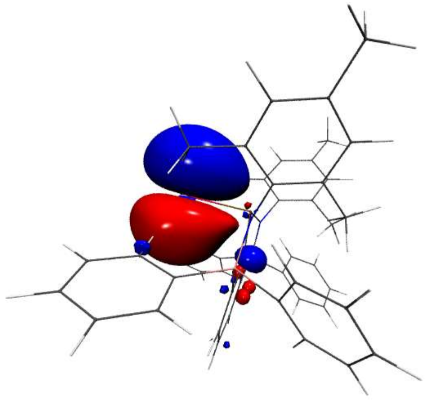
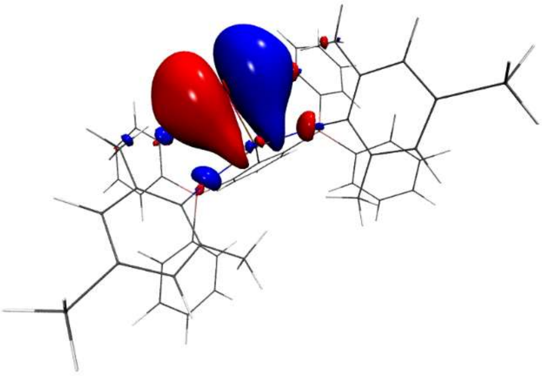

**NLMO 86**	**NLMO 87**
93.61 % of LP(2) Se	90.57 % of LP(3) Se
**Atomic Hybrid Contribution (with contributions > 1.00 %)**	
2.44 % As	5.14 % Sb
93.61 % Se	90.57 % Se
1.85 % C47	
**NLMO/NPA Bonder (% of total NLMO/NPA Bond order of 1.0706)**	
0.0489 (4.6 %)	0.1028 (9.6 %)

Table S8 cont. Natural Localized Molecular Orbital (NLMO) Analysis of **6AsTe**.

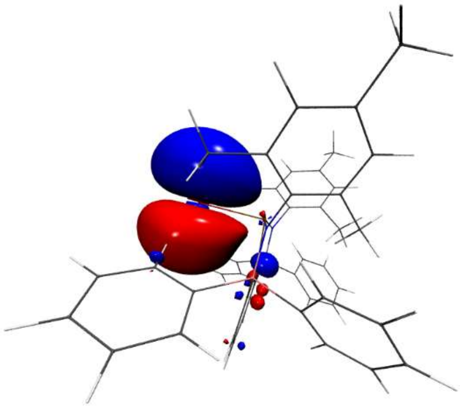

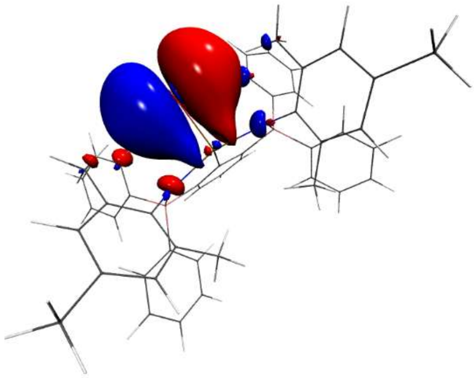

**NLMO 86**	**NLMO 87**
93.98 % of LP(2) Te	91.17 % of LP(3) Te
**Atomic Hybrid Contribution (with contributions > 1.00 %)**	
2.07 % Sb	4.04 % Sb
93.98 % Te	91.17 % Te
1.74 % C47	
**NLMO/NPA Bonder (% of total NLMO/NPA Bond order of 0.9748)**	
0.0413 (4.1 %)	0.0807 (8.3 %)

Table S9. NBO analysis of selected bonds of **6SbCh** (Ch = S, Se, Te) with respective hybrids.

Model	Occupation	Model	Occupation	Model	Occupation
**6SbS**		**6SbSe**		**6SbTe**	
**LP(1) Sb**	1.97	**LP(1) Sb**	1.97	**LP(1) Sb**	1.96
**LP(1) S**	1.98	**LP(1) Se**	1.99	**LP(1) Te**	1.99
**LP(2) S**	1.87	**LP(2) Se**	1.87	**LP(2) Te**	1.87
**LP(3) S**	1.82	**LP(3) Se**	1.82	**LP(3) Te**	1.82

Model	Occupation	Atom 1	Hybrid	Atom 2	Hybrid
**BD(1) Sb-S**	1.96	Sb (36.81 %)	$sp^{8.47}d^{0.02}$	S (63.19 %)	$sp^{6.06}d^{0.06}$
**BD(1) Sb-C**	1.95	Sb (24.47 %)	$sp^{9.89}d^{0.03}$	C (75.53 %)	$sp^{2.64}$
**BD(1) Sb-Se**	1.95	Sb (40.82 %)	$sp^{9.11}d^{0.02}$	Se (59.18 %)	$sp^{8.63}d^{0.07}$
**BD(1) Sb-C**	1.95	Sb (24.58 %)	$sp^{9.83}d^{0.03}$	C (75.42 %)	$sp^{2.63}$
**BD(1) Sb-Te**	1.96	Sb (46.89 %)	$sp^{10.09}d^{0.03}$	Te (53.11 %)	$sp^{10.81}d^{0.07}$
**BD(1) Sb-C**	1.95	Sb (24.79 %)	$sp^{9.58}d^{0.03}$	C (75.21 %)	$sp^{2.59}$

Donor NBO	Acceptor NBO	E2 [kcal/mol]
LP(3) S	LV(1) Sb	34.62
LP(2) S	LV(1) C	8.84
LP(2) S	BD*(1) Sb-C	9.51
LP(3) Se	LV(1) Sb	31.97
LP(2) Se	LV(1) C	10.47
LP(2) Se	BD*(1) Sb-C	8.42
LP(3) Te	LV(1) Sb	27.38
LP(2) Te	LV(1) C	13.69
LP(2) Te	BD*(1) Sb-C	7.06

Table S10. Natural Localized Molecular Orbital (NLMO) Analysis of **6SbS**.

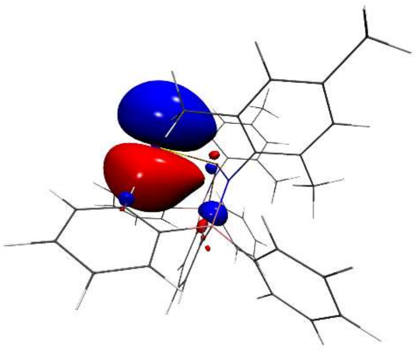

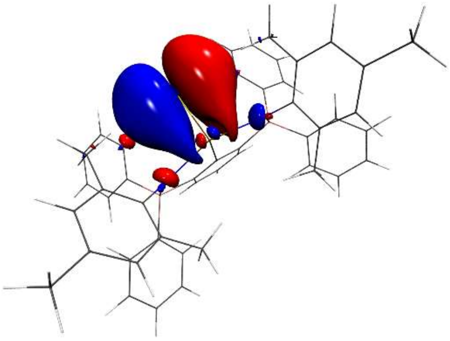

NLMO 77	NLMO 78
93.47 % of LP(2) S	90.98 % of LP(3) S
**Atomic Hybrid Contribution (with contributions > 1.00 %)**	
3.30 % Sb	5.65 % Sb
93.48 % S	90.98 % S
1.47 % C47	
**NLMO/NPA Bonder (% of total NLMO/NPA Bond order of 0.8887)**	
0.0661 (7.4 %)	0.1130 (12.7 %)

Table S10 cont. Natural Localized Molecular Orbital (NLMO) Analysis of **6SbSe**.

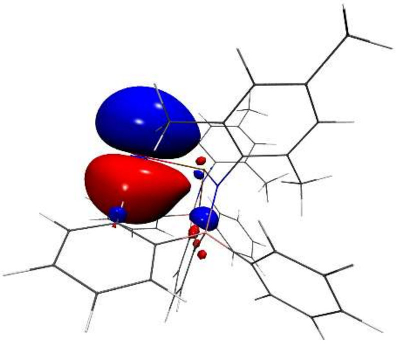

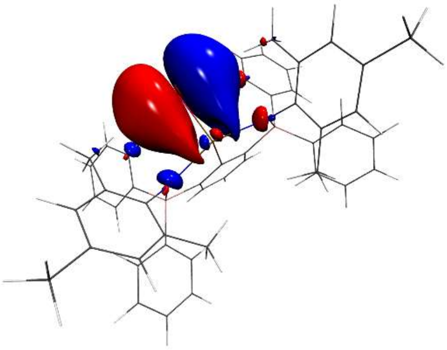

NLMO 81	NLMO 82
93.53 % of LP(2) Se	90.87 % of LP(3) Se
**Atomic Hybrid Contribution (with contributions > 1.00 %)**	
3.14 % Sb	5.52 % Sb
93.53 % Se	90.87 % Se
1.52 % C47	
**NLMO/NPA Bonder (% of total NLMO/NPA Bond order of 0.9559)**	
0.0628 (6.6 %)	0.1104 (11.5 %)

Table S10 cont. Natural Localized Molecular Orbital (NLMO) Analysis of **6SbTe**.

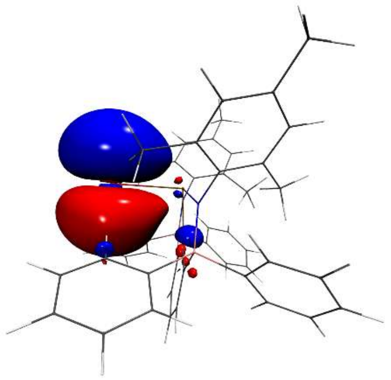	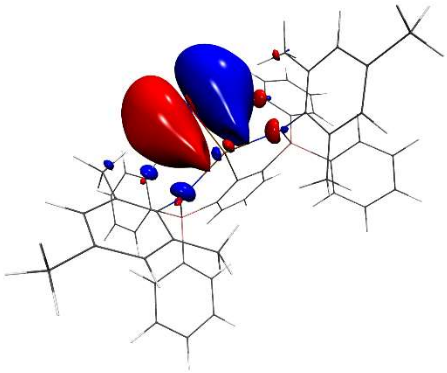
**NLMO 81**	**NLMO 82**
93.55 % of LP(2) Te	90.75 % of LP(3) Te
**Atomic Hybrid Contribution (with contributions > 1.00 %)**	
2.96 % Sb	5.17 % Sb
93.56 % Te	90.75 % Te
1.53 % C47	
**NLMO/NPA Bonder (% of total NLMO/NPA Bond order of 1.0591)**	
0.0592 (5.6 %)	0.1034 (9.8 %)

Table S11. NBO analysis of selected bonds of **6BiCh** (Ch = S, Se, Te) with respective hybrids.

**Model**	**Occupation**	**Model**	**Occupation**	**Model**	**Occupation**
**6BiS**		**6BiSe**		**6BiTe**	
**LP(1) Bi**	1.98	**LP(1) Bi**	1.98	**LP(1) Bi**	1.98
**LP(1) S**	1.98	**LP(1) Se**	1.99	**LP(1) Te**	1.99
**LP(2) S**	1.87	**LP(2) Se**	1.87	**LP(2) Te**	1.87
**LP(3) S**	1.82	**LP(3) Se**	1.81	**LP(3) Te**	1.80

**Model**	**Occupation**	**Atom 1**	**Hybrid**	**Atom 2**	**Hybrid**
**BD(1) Bi-S**	1.96	Bi (35.71 %)	$sp^{13.42}d^{0.02}$	S (64.29 %)	$sp^{6.85}d^{0.05}$
**BD(1) Bi-C**	1.94	Bi (23.58 %)	$sp^{16.50}d^{0.03}$	C (76.42 %)	$sp^{2.77}$
**BD(1) Bi-Se**	1.96	Bi (39.59 %)	$sp^{14.29}d^{0.02}$	Se (60.41 %)	$sp^{9.51}d^{0.06}$
**BD(1) Bi-C**	1.94	Bi (23.75 %)	$sp^{16.41}d^{0.03}$	C (76.25 %)	$sp^{2.77}$
**BD(1) Bi-Te**	1.95	Bi (45.33 %)	$sp^{15.47}d^{0.03}$	Te (54.67 %)	$sp^{11.48}d^{0.07}$
**BD(1) Bi-C**	1.95	Bi (24.10 %)	$sp^{16.01}d^{0.03}$	C (75.90 %)	$sp^{2.73}$

**Donor NBO**	**Acceptor NBO**	**E2 [kcal/mol]**
LP(3) S	LV(1) Bi	31.34
LP(2) S	LV(1) C	5.73
LP(2) S	BD*(1) Bi-C	9.20
LP(3) Se	LV(1) Bi	29.77
LP(2) Se	LV(1) C	6.55
LP(2) Se	BD*(1) Bi-C	8.17
LP(3) Te	LV(1) Bi	26.29
LP(2) Te	LV(1) C	7.94
LP(2) Te	BD*(1) Bi-C	7.18

Table S12. Natural Localized Molecular Orbital (NLMO) Analysis of **6BiS**.

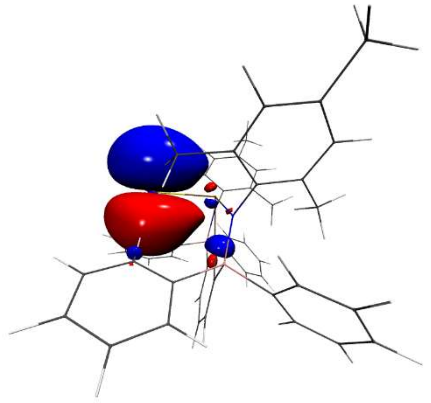

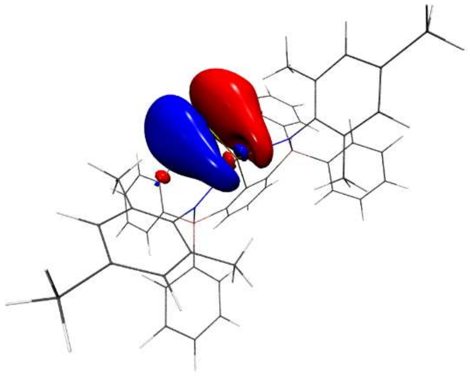

NLMO 77	NLMO 78
93.78 % of LP(2) S	89.84 % of LP(3) S
**Atomic Hybrid Contribution (with contributions > 1.00 %)**	
3.54 % Bi	7.96 % Bi
93.58 % S	89.84 % S
1.34 % C47	
**NLMO/NPA Bonder (% of total NLMO/NPA Bond order of 0.9015)**	
0.0707 (7.8 %)	0.1592 (17.7 %)

Table S12 cont. Natural Localized Molecular Orbital (NLMO) Analysis of **6BiSe**.

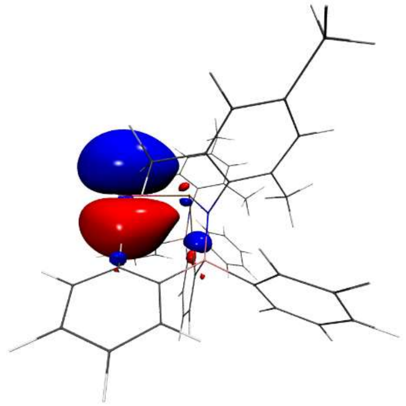

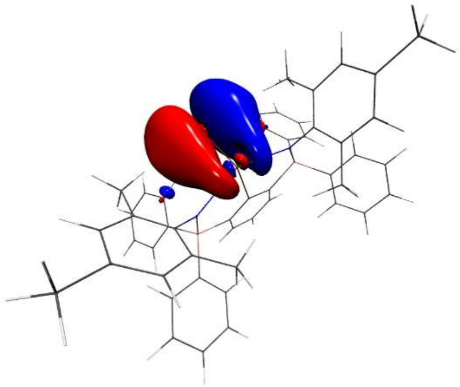

NLMO 81	NLMO 82
93.54 % of LP(2) Se	89.54 % of LP(3) Se
**Atomic Hybrid Contribution (with contributions > 1.00 %)**	
3.44 % Bi	8.02 % Bi
93.55 % Se	89.54 % Se
1.41 % C47	
**NLMO/NPA Bonder (% of total NLMO/NPA Bond order of 0.9729)**	
0.0688 (7.1 %)	0.1605 (16.5 %)

Table S12 cont. Natural Localized Molecular Orbital (NLMO) Analysis of **6BiTe**.

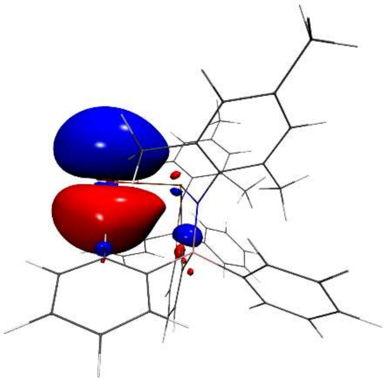	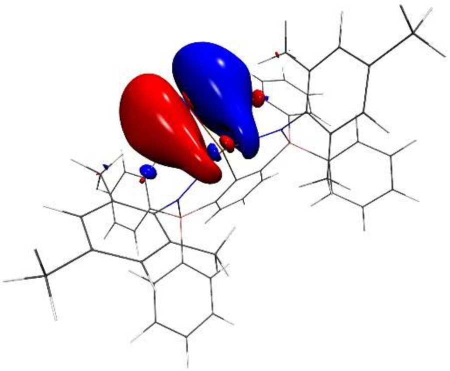
**NLMO 81**	**NLMO 82**
93.49 % of LP(2) Te	89.30 % of LP(3) Te
**Atomic Hybrid Contribution (with contributions > 1.00 %)**	
3.34 % Bi	7.87 % Bi
93.49 % Te	89.30 % Te
1.45 % C47	
**NLMO/NPA Bonder (% of total NLMO/NPA Bond order of 1.0743)**	
0.0668 (6.2 %)	0.1573 (14.6 %)

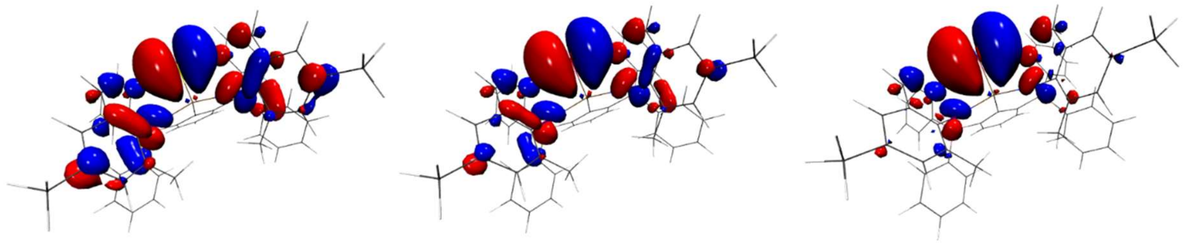

Figure S136: Respective HOMO–1 of **6AsS** (left), **6AsSe** (middle) and **6AsTe** (right) at *iso*-surfaces at s(r) = +/– 0.02 (blue/red) representing the π-bonding contribution.

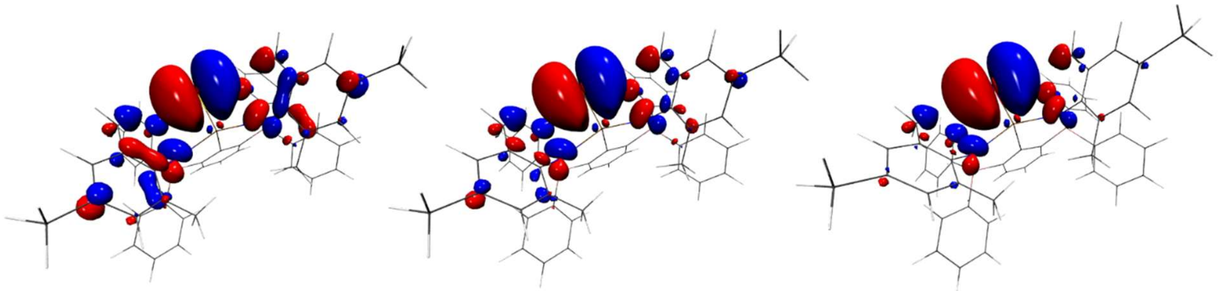

Figure S137: Respective HOMO–1 of **6SbS** (left), **6SbSe** (middle) and **6SbTe** (right) at *iso*-surfaces at s(r) = +/– 0.02 (blue/red) representing the π-bonding contribution.

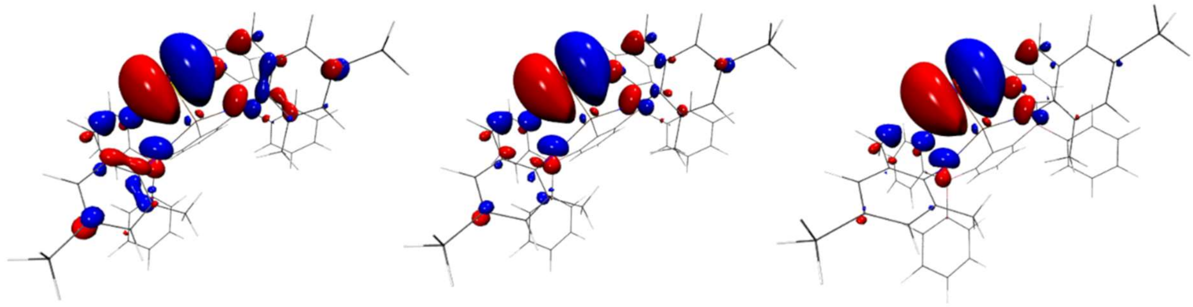

Figure S138: Respective HOMO–1 of **6BiS** (left), **6BiSe** (middle) and **6BiTe** (right) at *iso*-surfaces at s(r) = +/– 0.02 (blue/red) representing the π-bonding contribution.

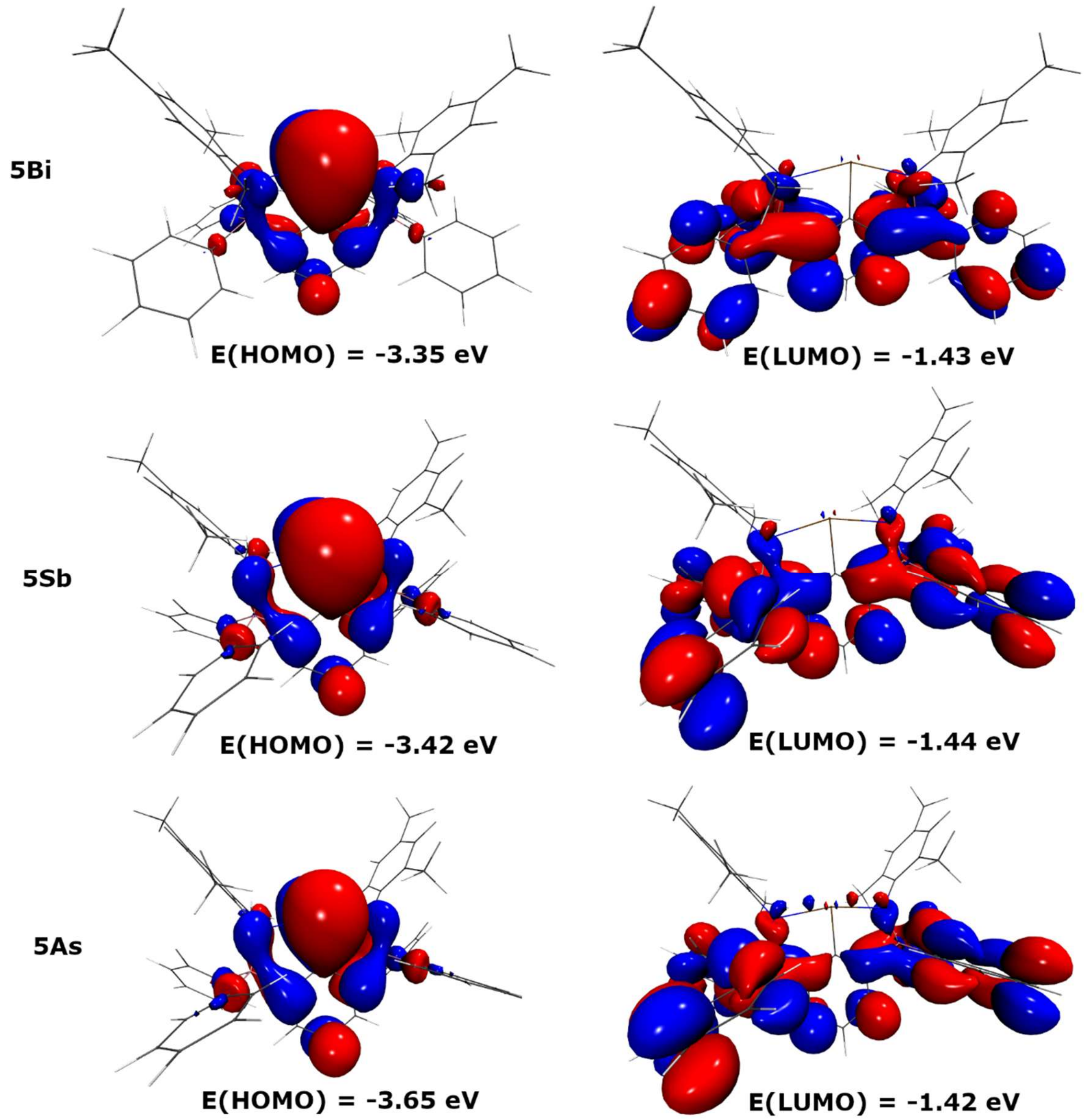

Figure S139. HOMOs (left) and LUMOs (right) of **5As** (top), **5Sb** (middle) and **5Bi** (bottom) at *iso*-surfaces at s(r) = +/– 0.02 (blue/red).

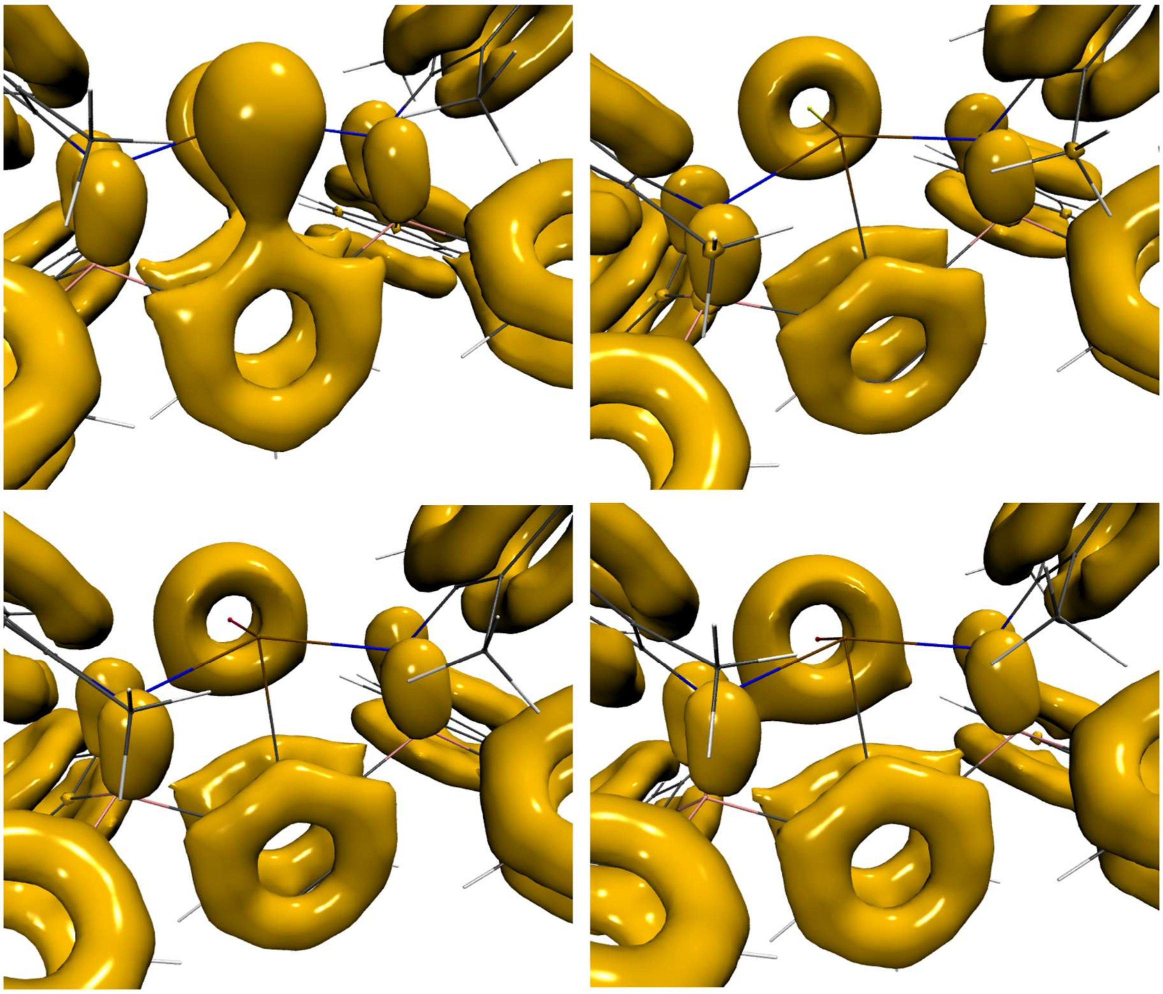

Figure S140. LOL-π of **5Bi** (top left), **6BiS** (top right), **6BiSe** (bottom left) and **6BiTe** (bottom right) at *iso*-surfaces of 0.35.

Table S13. EDA-NOCV derived energy terms for **6SbSe** and **6BiTe**, values given in kcal/mol.

Energy term	6SbSe	6BiTe
**Double bond Pn=Ch (with ^{3}Pn and ^{3}Ch fragments)**		
ΔE_{int}	–133.49	–119.30
ΔE_{Pauli}	334.20	279.42
ΔV_{elstat}	–131.58	–114.01
ΔE_{orb}	–324.89	–271.90
ΔE_{disp}	–11.22	–12.80
**Single bond $^+$Pn–Ch$^-$ (with ^{2}Pn$^+$ and ^{2}Ch$^-$ fragments)**		
ΔE_{int}	–161.29	–145.65
ΔE_{Pauli}	366.66	312.95
ΔV_{elstat}	–253.86	–229.72
ΔE_{orb}	–260.71	–213.94
ΔE_{disp}	–13.38	–14.94

Table S14. Percentage buried volume for **5Pn** (Pn = As, Sb, Bi) and **6AsCh**, **6SbCh** and **6BiCh** and related reported compounds, for which the percentage buried volume have been determined based on the reported optimized coordinates within Reference 14 of the main manuscript.

**5As**	**6AsS**	**6AsSe**	**6AsTe**
64.0	62.9	62.9	63.0
**5Sb**	**6SbS**	**6SbSe**	**6SbTe**
57.9	57.4	57.2	57.1
**5Bi**	**6BiS**	**6BiSe**	**6BiTe**
54.3	54.1	54.0	53.9

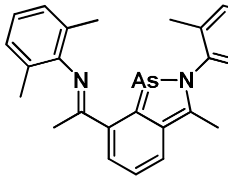

61.5

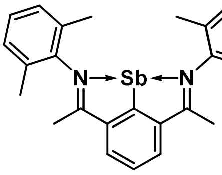

53.9

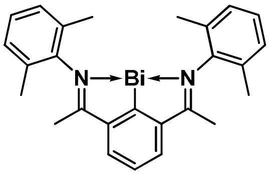

48.5

References:

[58] O. V. Dolomanov, L. J. Bourhis, R. J. Gildea, J. A. K. Howard, H. Puschmann, *J. Appl. Crystallogr.* 2009, **42**, 339−341.

[59] H. Putz, K. Brandenburg GbR, Diamond–Crystal and Molecular Structure Visualization, Crystal Impact: Kreuzherrenstr. 102, 53227 Bonn, Germany, 2023.

[60] (a) A. D. Becke, *J. Chem. Phys.*, 1993, **98**, 5648−5652; (b) J. P. Perdew, J. A. Chevary, S. H. Vosko, K. A. Jackson, M. R. Pederson, D. J. Singh and C. Fiolhais, *Phys. Rev. B: Condens. Matter Mater. Phys.*, 1992, **46**, 6671−6687.

[61] (a) R. Krishnan, J. S. Binkley, R. Seeger and J. A. Pople, *J. Chem. Phys.*, 1980, **72**, 650−654; (b) A. D. McLean and G. S. Chandler, *J. Chem. Phys.*, 1980, **72**, 5639−5648.

[62] Gaussian16 (RevisionC.01), M. J. Frisch, G. W. Trucks, H. B. Schlegel, G. E. Scuseria, M. A. Robb, J. R. Cheeseman, G. Scalmani, V. Barone, G. A. Petersson, H. Nakatsuji, X. Li, M. Caricato, A. V. Marenich, J. Bloino, B. G. Janesko, R. Gomperts, B. Mennucci, H. P. Hratchian, J. V. Ortiz, A. F. Izmaylov, J. L. Sonnenberg, D. Williams-Young, F. Ding, F. Lipparini, F. Egidi, J. Goings, B. Peng, A. Petrone, T. Henderson, D. Ranasinghe, V. G. Zakrzewski, J. Gao, N. Rega, G. Zheng, W. Liang, M. Hada, M.Ehara, K. Toyota, R. Fukuda, J. Hasegawa, M. Ishida, T. Nakajima, Y. Honda, O. Kitao, H. Nakai, T. Vreven, K. Throssell, J. A. Montgomery, Jr., J. E.Peralta, F. Ogliaro, M. J. Bearpark, J. J. Heyd, E. N. Brothers, K. N. Kudin, V. N. Staroverov, T. A. Keith, R. Kobayashi, J. Normand, K. Raghavachari, A. P. Rendell, J. C. Burant, S. S. Iyengar, J. Tomasi, M. Cossi, J. M. Millam, M. Klene, C. Adamo, R. Cammi, J. W. Ochterski, R. L. Martin, K. Morokuma, O. Farkas, J. B. Foresman and D. J. Fox, Gaussian, Inc., Wallingford CT, 2019.

[63] (a) B. Metz, H. Stoll and M. Dolg, *J. Chem. Phys.*, 2000, **113**, 2563-2569; (b) K. A. Peterson, *J. Chem. Phys.*, 2003, **119**, 11099−11112. (c) K. A. Peterson, D. Figgen, E. Goll, H. Stoll and M. Dolg, *J. Chem. Phys*., 2003, **119**, 11113-11123.

[64] (a) D. Feller, *J. Comput. Chem.*, 1996, **17**, 1571−1586. (b) K. L. Schuchardt, B. T. Didier, T. Elsethagen, L. Sun, V. Gurumoorthi, J. Chase, J. Li and T. L. Windus, *J. Chem. Inf. Model.*, 2007, **47**, 1045−1052. (c) B. P. Pritchard, D. Altarawy, B. Didier, T. D. Gibson and T. L. Windus, *J. Chem. Inf. Model.*, 2019, **59**, 4814-4820.

[65] S. Grimme, S. Ehrlich and L. Goerigk, *J. Comp. Chem.*, 2011, **32**, 1456-1465.

[66] AIMAll (Version 15.09.27), Todd A. Keith, TK Gristmill Software, Overland Park KS, USA, 2015 (aim.tkgristmill.com)

[67] J. Contreras-García, E. Johnson, S. Keinan, R. Chaudret, J.-P. Piquemal, D. Beratan and W. Yang, *J. Chem. Theory Comput.*, 2011, **7**, 625-632.

[68] NBO 6.0., E. D. Glendening, J. K. Badenhoop, A. E. Reed, J. E. Carpenter, J. A. Bohmann,C. M. Morales, C. R. Landis and F. Weinhold, Theoretical Chemistry Institute, University of Wisconsin, Madison, 2013.

[69] (a) IGM: C. Lefebvre, G. Rubez, H. Khartabil, J.-C. Boisson, J. Contreras-García and E. Hénon, *Phys. Chem. Chem. Phys*., 2017, **19**, 17928–17936. (b) IGMH: T. Lu, *J. Comput. Chem.*, 2022, **43**, 539–555.

[70] T. Lu and F. Chen, *J. Comput. Chem.*, 2012, **33**, 580−592.

[71] W. Humphrey, A. Dalke and K. Schulten, *J. Mol. Graph.*, 1996, **14**, 33-38.

[72] ADF 2025.1, SCM, Theoretical Chemistry, Vrije Universiteit, Amsterdam, The Netherlands, http://www.scm.com, E. J. Baerends, T. Ziegler, A. J. Atkins, J. Autschbach, O. Baseggio, D. Bashford, A. Bérces, F. M. Bickelhaupt, C. Bo, P. M. Boerrigter, C. Cappelli, L. Cavallo, C. Daul, D. P. Chong, D. V. Chulhai, L. Deng, R. M. Dickson, J. M. Dieterich, F. Egidi, D. E. Ellis, M. van Faassen, L. Fan, T. H. Fischer, A. Förster, C. Fonseca Guerra, M. Franchini, A. Ghysels, A. Giammona, S. J. A. van Gisbergen, A. Goez, A. W. Götz, J. A. Groeneveld, O. V. Gritsenko, M. Grüning, S. Gusarov, F. E. Harris, P. van den Hoek, Z. Hu, C. R. Jacob, H. Jacobsen, L. Jensen, L. Joubert, J. W. Kaminski, G. van Kessel, C. König, F. Kootstra, A. Kovalenko, M. V. Krykunov, P. Lafiosca, E. van Lenthe, D.A. McCormack, M. Medves, A. Michalak, M. Mitoraj, S. M. Morton, J. Neugebauer, V. P. Nicu, L. Noodleman, V. P. Osinga, S. Patchkovskii, M. Pavanello, C. A. Peeples, P. H. T. Philipsen, D. Post, C. C. Pye, H. Ramanantoanina, P. Ramos, W. Ravenek, M. Reimann, J. I. Rodríguez, P. Ros, R. Rüger, P. R. T. Schipper, D. Schlüns, H. van Schoot, G. Schreckenbach, J. S. Seldenthuis, M. Seth, J.G. Snijders, M. Solà, M. Stener, M. Swart, D. Swerhone, V. Tognetti, G. te Velde, P. Vernooijs, L. Versluis, L. Visscher, O. Visser, F. Wang, T. A. Wesolowski, E.M. van Wezenbeek, G. Wiesenekker, S. K. Wolff, T. K. Woo, A. L. Yakovlev.

[73] E. Van Lenthe and E. J. Baerends, *J. Comput. Chem.*, 2003, **24**, 1142-1156.

[74] E. Caldeweyher, S. Ehlert, A. Hansen, H. Neugebauer, S. Spicher, C. Bannwarth and S. Grimme, *J. Chem. Phys.*, 2019, **150**, 154122.

[75] L. Falivene, Z. Cao, A. Petta, L. Serra, A. Poater, R. Oliva, V. Scarano and L. Cavallo, *Nat. Chem.* 2019, **11**, 872-879.

[76] J. Pipik and P. G. Mezey *J. Chem. Phys.* 1989, **90**, 4916-4926.

[77] F. Neese *Wiley Interdiscip. Rev. Comput. Mol. Sci.* 2012, **2**, 73–78; b) for Version 6.0 and above also: F. Neese *Wiley Interdiscip. Rev. Comput. Mol. Sci.* 2025, **15**, e70019; c) F. Neese, F. Wennmohs, U. Becker and C. Riplinger, *J. Chem. Phys.* 2020, **152**, Art.Nr. L224108.

[78] F. Weigend and R. Ahlrichs, *Phys. Chem. Chem. Phys.* 2005, **7**, 3297–3305.
